# Comprehensive evaluation of structural variation detection algorithms for whole genome sequencing

**DOI:** 10.1186/s13059-019-1720-5

**Published:** 2019-06-03

**Authors:** Shunichi Kosugi, Yukihide Momozawa, Xiaoxi Liu, Chikashi Terao, Michiaki Kubo, Yoichiro Kamatani

**Affiliations:** 1 Laboratory for Statistical Analysis, RIKEN Center for Integrative Medical Sciences, 1-7-22 Suehiro-cho, Tsurumi-ku, Yokohama, 230-0045 Japan; 2 Laboratory for Statistical and Translational Genetics, RIKEN Center for Integrative Medical Sciences, 1-7-22 Suehiro-cho, Tsurumi-ku, Yokohama, 230-0045 Japan; 3 Laboratory for Genotyping Development, RIKEN Center for Integrative Medical Sciences, 1-7-22 Suehiro-cho, Tsurumi-ku, Yokohama, 230-0045 Japan; 4 RIKEN Center for Integrative Medical Sciences, 1-7-22 Suehiro-cho, Tsurumi-ku, Yokohama, 230-0045 Japan

**Keywords:** Structural variation, SV, Copy number variation, CNV, Next generation sequencing, WGS

## Abstract

**Background:**

Structural variations (SVs) or copy number variations (CNVs) greatly impact the functions of the genes encoded in the genome and are responsible for diverse human diseases. Although a number of existing SV detection algorithms can detect many types of SVs using whole genome sequencing (WGS) data, no single algorithm can call every type of SVs with high precision and high recall.

**Results:**

We comprehensively evaluate the performance of 69 existing SV detection algorithms using multiple simulated and real WGS datasets. The results highlight a subset of algorithms that accurately call SVs depending on specific types and size ranges of the SVs and that accurately determine breakpoints, sizes, and genotypes of the SVs. We enumerate potential good algorithms for each SV category, among which GRIDSS, Lumpy, SVseq2, SoftSV, Manta, and Wham are better algorithms in deletion or duplication categories. To improve the accuracy of SV calling, we systematically evaluate the accuracy of overlapping calls between possible combinations of algorithms for every type and size range of SVs. The results demonstrate that both the precision and recall for overlapping calls vary depending on the combinations of specific algorithms rather than the combinations of methods used in the algorithms.

**Conclusion:**

These results suggest that careful selection of the algorithms for each type and size range of SVs is required for accurate calling of SVs. The selection of specific pairs of algorithms for overlapping calls promises to effectively improve the SV detection accuracy.

**Electronic supplementary material:**

The online version of this article (10.1186/s13059-019-1720-5) contains supplementary material, which is available to authorized users.

## Background

Genomic structural variations (SVs) are generally defined as deletions (DELs), insertions (INSs), duplications (DUPs), inversions (INVs), and translocations (TRAs) of at least 50 bp in size. SVs are often considered separately from small variants, including single nucleotide variants (SNVs) and short insertions, and deletions (indels), as these are often formed by distinct mechanisms [1]. INVs and TRAs are balanced forms, with no net change in a genome, and the remaining SVs are imbalanced forms. Imbalanced deletions (DELs) and duplications (DUPs) are also referred to as copy number variations (CNVs), with DUPs comprising tandem and interspersed types depending on the distance between the duplicated copies [2, 3]. INSs are categorized into several classes based on the insertion sequences: mobile element insertions (MEIs), nuclear insertions of mitochondrial genome (NUMTs), viral element insertions (VEIs; referred to in this study), and insertions of unspecified sequence.

SVs are largely responsible for the diversity and evolution of human genomes at both individual and population level [3–6]. The genomic difference between individuals caused by SVs has been estimated to be 3–10 times higher than that by SNVs [2, 6, 7]. Consequently, SVs could have higher impacts on gene functions and phenotypic changes than do SNVs and short indels. Accordingly, SVs are associated with a number of human diseases, including neurodevelopmental disorders and cancers [3, 8–11].

Two types of methods have been used to detect SVs: (1) array-based detection, including microarray comparative genome hybridization (array CGH), and (2) sequencing-based computational methods [2, 12]. Array-based methods are advantageous for high-throughput analysis, but they only detect certain types of SVs, have a lower sensitivity for small SVs, and have a lower resolution for determining breakpoints (BPs) than the sequencing-based methods. Although sequencing requires more time and money than the array-based method, it would be necessary for detecting a broad range of SVs to adopt the sequencing-based methods, as in recent projects aimed at identifying SVs on a population scale [6, 13–15].

Sequencing-based methods take several conceptual approaches to derive information about SVs from short read sequencing data [2, 9, 16–18]. Read pairs (RP) and read depth (RD) approaches utilize the discordant alignment features and depth features of paired-end reads that encompass or overlap an SV, respectively. The split read (SR) approach uses split (soft-clipped) alignment features of single-end or paired-end reads that span a BP of a SV. The assembly (AS) approach detects SVs by aligning the contigs, assembled with the entire or unmapped sequencing reads, to the reference sequence. A number of recently developed SV detection algorithms use a combination (CB) of the above four methods (here, we refer to these five basic SV detection methods as “methods” and each specific SV detection tool as an “algorithm”). Irrespective of the strategy, sequencing-based methods suffer from a high rate of miscalling of SVs because they involve errors in base call, alignment, or de novo assembly, especially in repetitive regions unable to be spanned with short reads. To overcome the shortcomings of short read sequencing, long reads generated using single-molecule sequencing technology have recently been used to detect SVs in a human sample using the AS and/or SR approach [19–22]. However, the high cost and the low throughput of this strategy currently limits its general use.

Although the sequencing-based methods can in theory detect any type of SV, no single computational algorithm can accurately and sensitively detect all types and all sizes of SVs [23]. Therefore, most projects use multiple algorithms to call SVs, then merge the outputs to increase the precision and/or the recall [6, 13–15, 17, 24–29]. Many projects use popular SV detection algorithms, including BreakDancer [30], CNVnator [31], DELLY [32], GenomeSTRiP [33], Pindel [34], and Lumpy [35], which give calls with relatively high accuracy. Although one study has investigated for the performances of 13 SV detection algorithms [36], there has been no systematic investigation of which algorithms can accurately detect which types of SVs. Importantly, while it is common practice to do so, there has been no systematic investigation into optimal strategies to combine the results of multiple algorithms to come to the most complete characterization of SVs in a genome. In this study, we evaluated 69 algorithms for their precision and recall for both single and overlapping SV callings, using multiple simulated and real datasets of WGS datasets.

## Results

### Evaluation of SV detection algorithms using simulated and real WGS data

We accessed 79 publicly available SV detection algorithms that can handle the human WGS data but do not require multiple samples such as matched datasets (e.g., control and tumor samples). We excluded 10 algorithms that did not work in our computational environment. Completed results were obtained with 69 algorithms using simulated and real human WGS data (Additional file 1: Tables S1 and S2, please see Additional file 1: Table S1 for the reference for each algorithm described below and Additional file 1: Table S2 for the list of unworked algorithms) to calculate the precision and recall. A simulated short read dataset was generated using the VarSim simulator [37]: first, a simulated GRCh37 human diploid genome into which known SVs had been introduced at the known sites was generated, then this was used to generate simulated paired-end short reads (125 bp) with 500 bp insert size averaging 30× coverage of the simulated genome (Sim-A). The number of simulated SVs of each type was slightly larger than the mean numbers detected for an individual human genome in the 1000 Genome project [6] (e.g., 1.3-fold higher for DELs, Additional file 1: Table S4-A and S4-C). Four sets of the NA12878 Illumina short read data (data1, data2, data3, and data4) and three sets of PacBio long read data (PacBio-data1, PacBio-data2, and PacBio-data3) were used as real datasets and were acquired from different sources with different read lengths and/or insert sizes (Additional file 1: Table S3). A reference SV dataset for the real data was generated by merging the DGV dataset corresponding to NA12878 and the INS, DEL, and INV data detected from NA12878 long read assemblies (Additional file 1: Table S4; see the “Methods” section for details).

These datasets, including the simulated data and four or three NA12878 datasets, were aligned with the GRCh37d5 reference genome using bwa [38] or other specific alignment tools (see the “Methods” section). The alignment data or read data were then used for calling DELs, DUPs, INSs, and INVs in all but the Y chromosome for the real data. Translocations were not evaluated because there are few known translocations in the databases and VarSim cannot simulate translocations. For DELs and DUPs, SVs were divided into four and three categories, respectively, depending on their sizes (DEL-SS: 50–100 bp; DEL-S and DUP-S, 100 bp to 1 kb; DEL-M and DUP-M, 1–100 kb; DEL-L and DUP-L, 100 kb to 1 Mb). We defined true called SVs as the called SVs that significantly overlap with the reference SVs by proportions (≧ 50% [or ≧ 80% for the simulated data] reciprocal overlap for DELs, DUPs, and INVs; overlap with a BP ± 200 bp for INSs). The outline of the entire evaluation processes is presented in Figure S1 in Additional file 1.

We observed changes in precision and recall by using different filtering thresholds; the minimum number of reads supporting the called SVs, termed “RSS” (Reads Supporting SV) in this study (see Additional file 1: Figure S2 for representative examples). Thus, to compare the performance of each algorithm as objectively as possible, we selected an RSS for each call set at which the numbers of calls for an SV type approximates the simulated reference data or the expected number of SVs in an individual (see the “Methods” section for detail). Both precision and recall were calculated for each size range of DELs (Additional file 1: Figure S3), DUPs (Additional file 1: Figure S4), INSs, and INVs (Additional file 1: Figure S5); for the real data, the mean precision and recall from the four short read datasets are presented. The numerical data for all the results for the Sim-A and multiple NA12878 real datasets are presented in Tables S5-S9 in Additional file 3. The precision and recall values at the selected RSSs for the four NA12878 real datasets and the mean and the standard deviation (SD) are presented in Table S10 in Additional file 3.

**Fig. 1:**
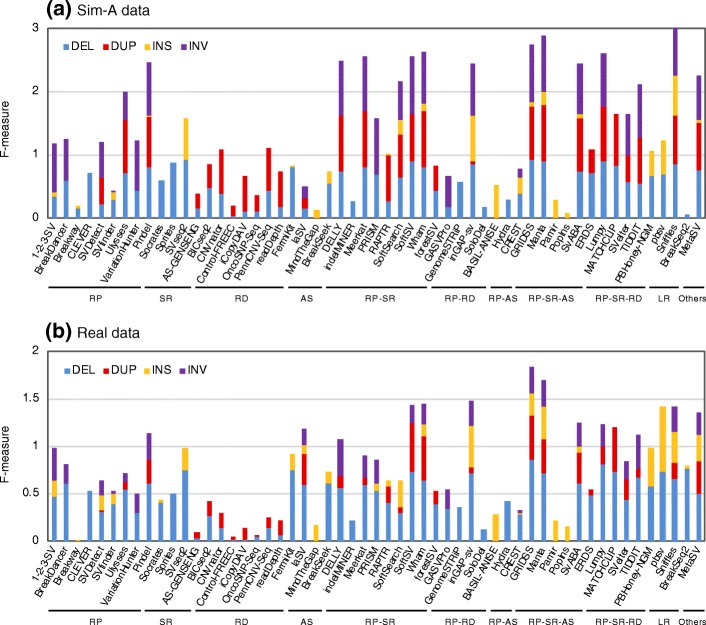
SV type specificity of SV detection algorithms. Precision and recall of DELs, DUPs, INSs, and INVs were determined with the simulated (**a**) and the NA12878 real data (**b**). Modified *F*-measures (the combined statistics for precision and recall (see the “Methods” section for details)) are shown for the algorithms indicated with blue (for DEL), red (for DUP), orange (for INS), and purple (for INV) bars. The mean values of the results obtained with the four NA12878 real datasets (three PacBio datasets for long reads) are indicated. The algorithms were categorized according to the methods used to detect SV signals (RP, read pairs; SR, split reads; RD, read depth; AS, assembly; LR, long reads) and their combined methods (RP-SR, RP-RD, RP-AS, RP-SR-AS, and RP-SR-RD)

**Fig. 2:**
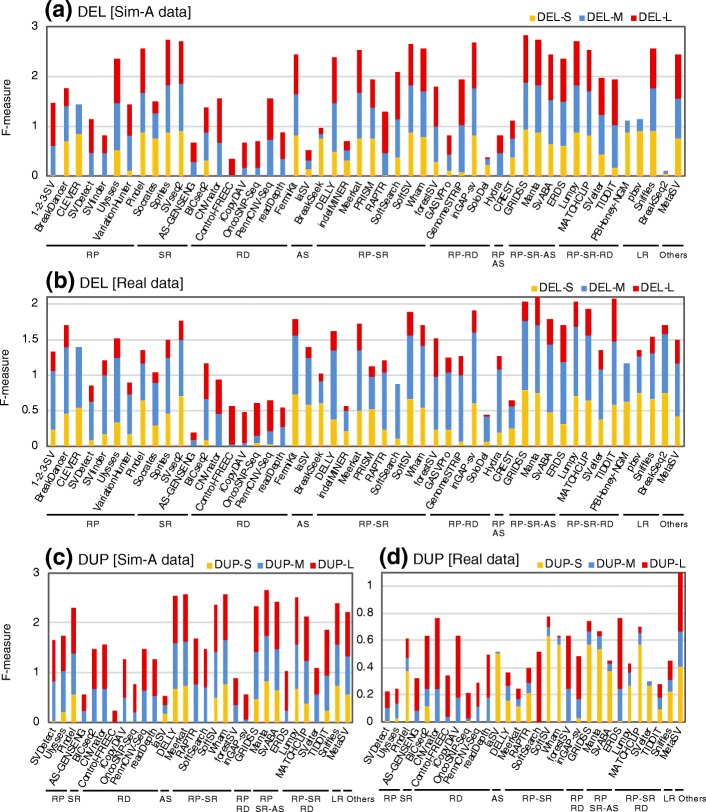
Size range specificity of SV detection algorithms for DELs and DUPs. Precision and recall of each size range of DELs (**a**, **b**) and DUPs (**c**, **d**) were determined with the simulated (**a**, **c**) and the NA12878 real data (**b**, **d**). Modified *F*-measures (the combined statistics for precision and recall) are shown for the algorithms indicated with orange (for S, 100 bp to 1 kb), blue (for M, 1 to 100 kb), and red (for L, 100 kb to 1 Mb) bars. The mean values of the results obtained with the four (or three) NA12878 real datasets are indicated. The algorithms were categorized according to the methods used to detect SV signals, as in Fig. 1

The precision and recall for calling SVs varied greatly depending on the algorithm, the SV type, and the size of the SV. Figures 1 and 2 highlight a number of algorithms that specifically and/or sensitively detected SVs for each type of SV and for each size range of SV (also see Additional file 1: Figures S3–S5 for precision–recall plots). Figure 1 shows the combined statistics (*F*-measure) for the precision and recall of each algorithm for calling each SV type and highlights a subset of algorithms that can call many SVs with a high level of precision and recall for both simulated and real datasets, which include 1-2-3-SV [39], DELLY [32], GRIDSS [40], inGAP-sv [41], Lumpy [35], Manta [42], MetaSV [43], Pindel [34], SoftSV [44], SvABA [45], and Wham [46]. Although many of the algorithms that call DELs or DUPs covered all the size ranges (S, M, and L) for both the simulated and real datasets, a subset of algorithms exhibited a limited performance in a specific size range (Fig. 2). For example, CLEVER [47] less effectively detected large DELs, and depth-based algorithms (e.g., AS-GENESENG [48], Control-FREEC [49], CNVnator, OncoSNP-Seq [50], readDepth [51], and GenomeSTRiP [33]) less effectively detected small DELs and/or DUPs.

The algorithms benchmarked in this study are based on one of the 10 method classes, including RP, RD, SR, AS, or LR alone, or one of five combined methods (RP-RD, RP-SR, RP-AS, RP-RD-S, and RP-SR-AS) (Additional file 1: Table S1). For calling DEL and DUP, the SR, LR, and RP-SR-AS methods achieved relatively good performance both with the simulated and the real data as shown in the precision–recall plots for the 10 categorized SV detection methods (Additional file 1: Figure S6).

In addition, we determined potential false-positive calls for each algorithm using NA12878 pedigree data, NA12878 for child and NA12891 and NA12892 for parents (Additional file 1: Table S3). The variants present only in child but not in both parents are attributable to Mendelian inheritance errors or de novo variants. Because the occurrence of de novo SVs is quite low and is thus negligible [28], the SV calls from only child are derived from Mendelian inheritance errors or false-negative call in parents. We determined Mendelian inheritance error rate (MIER; the percentage of Mendelian inheritance errors in the total calls) for each algorithm in each SV type. We observed a weak correlation between “100 − MIER” and precision for each algorithm in each SV type (the Spearman rank correlation coefficients, 0.31~0.46 for each SV type) (Additional file 1: Figure S7 and Additional file 3: Tables S6–S10 for numerical data). The weak correlation may be due to false-negative calls in parents and/or the presence of false positives that are called commonly between parents and child.

### Evaluation with HG00514 WGS data

We further evaluated SV detection algorithm using another WGS real data of a Han Chinese individual HG00514 (Additional file 1: Table S3), which is one of the data used in the Human Genome Structural Variation Consortium (HGSV). In HGSV, a HG00514 SV set had been generated using 13 short read-based SV detection algorithms and using an approach with long read-based assemblies [36]. We used this SV set as a reference SV set, although it was devoid of INVs (Additional file 1: Table S4; see the “Methods” section for detail). We showed the performance of each algorithm for each type of SV and for each size range of SV using *F*-measure (Additional file 1: Figures S8 and S9) and using precision–recall plots (Additional file 1: Figures S10 and S11, and Additional file 3: Table S11 for numerical data), as demonstrated for the NA12878 datasets in the previous section. Although the tendency of precision and recall between algorithms was similar to that of the NA12878 results, the overall precision values especially for DELs were lower than those of NA12878 (mean precision in HG00514: 53.6 for DEL, 22.5 for DUP, 42.9 for INS; mean precision in NA12878: 62.0 for DEL, 27.9 for DUP, 47.7 for INS).

We examined the correlation in the SV calling accuracies between the six datasets (the four NA12878 real datasets, one HG00514 real dataset, and one simulation dataset), by comparing the accuracy ranks of algorithms between SV types and/or datasets with the Spearman rank correlation coefficients (Additional file 1: Figure S12). The rank correlation coefficients for these algorithms were high (> 0.7 for almost all cases) for all types of SV between the five real datasets, suggesting that the determined SV calling accuracies for the tested algorithms were robust at least among the NA12878 and HG00514 datasets. The accuracy ranks between the simulated and NA12878 real datasets correlated reasonably well for DELs (0.72) and INSs (0.61) but weakly correlated for INVs (0.57) and DUPs (0.48). This result suggests that the simulated data fails to accurately model the mechanisms of SV formation, especially the properties of the real DUPs and INVs, which often involve complex SVs in which other types of SVs are integrated [24]. Alternatively, DUPs and INVs for NA12878 may be insufficiently represented in the reference databases. Exceptionally, the accuracy ranks for DUPs between the simulated and HG00514 real datasets (0.72) were considerably higher than those between the simulated and NA12878 real datasets (0.49). This high correlation is probably because HG00514 DUPs reported in HGSV have been detected mainly with short read-based SV detection algorithms [36], in contrast with NA12878 DUPs that are derived mainly from array-based detection. On the other hand, the high correlation between all the datasets observed for DELs was probably because the NA12878 reference DELs were covered with the datasets derived from both array-based and assembly-based SV detection.

### Evaluation of algorithms that call MEIs, NUMTs, and VEIs

**Fig. 3:**
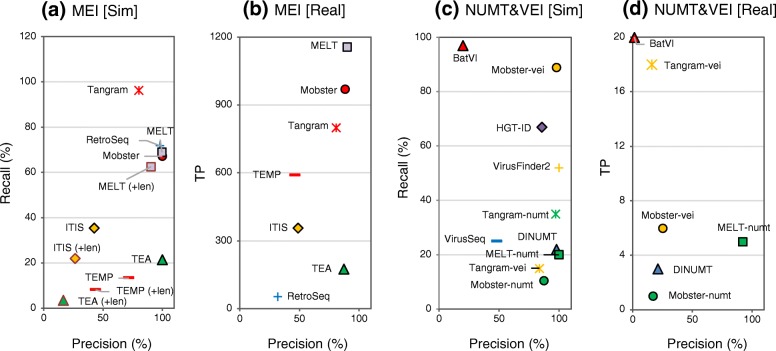
Precision and recall of MEIs, NUMTs, and VEIs called using existing algorithms. MEI (**a**, **b**), NUMT, and VEI (**c**, **d**) insertions were called using the indicated algorithms and simulated data (**a**, **c**) and the real data (**b**, **d**). NUMTs and VEIs were called using algorithms including modified versions of Mobster, MELT, and Tangram (Mobster-numt, Mobster-vei, MELT-numt, Tangram-numt, and Tangram-vei). For the real data, the mean values of the results obtained with the four NA12878 real datasets (data1 to data4) are indicated. VirusFinder and HGT-ID could not be applied to accomplish the runs for the real data due to unresolvable errors. The precision and recall percentages (or the number of true positives for the real data) determined for the respective call sets are indicated on the *x*-axis and *y*-axis, respectively. The data labeled with (+len) were determined considering insertion length in addition to breakpoints in (**a**). In this case, called sites were judged as true when the ratio of the called MEI lengths and the matched reference MEI length was ≧ 0.5 and ≦ 2.0. The algorithms without the label do not output the defined length of insertions

Based on the identity of the inserted sequence, some INSs can be classified into special classes including MEIs, NUMTs, and VEIs. Thus, we next evaluated the subset of computational algorithms that detect specific classes of INSs. We used three different simulated datasets (Sim-MEI, Sim-NUMT, and Sim-VEI, generated using only the chr17 sequence; see the “Methods” section) and the four NA12878 real datasets to evaluate the performances of 12 algorithms and an additional five derivatives of three algorithms (Fig. 3, and see Additional file 3: Tables S5–S10 for the numerical data). For the real data, the numbers of true positives (TPs) was determined in place of recall, because MEI, NUMT, and VEI have not been defined for the NA12878 INS reference. We added NUMT-compatible versions of Mobster [52], MELT [53], and Tangram [54] (Mobster-numt, MELT-numt, and Tangram-numt) and VEI-compatible versions of Mobster and Tangram (Mobster-vei, Tangram-vei) to NUMT- and VEI-detection algorithms, respectively (see Additional file 4: Supplementary methods for detail).

For MEI calling, MELT and Mobster achieved higher performances with both the simulated and real data than the other algorithms (> 88% in precision and > 50% in recall [> 900 TPs], Fig. 3a and b). Although MELT had the highest recall for MEI calling, RetroSeq, Tangram, and Mobster exhibited higher recall metrics in calling simulated LINE1 than MELT (Additional file 3: Table S5). For NUMT, MELT-numt exhibited the highest precision (> 92%) both with the simulated and the real data but exhibited only 20% recall with the simulated data (Fig. 3c and d). A more increased recall for NUMT calling may be achieved by a combination with Tangram-numt or DINUMT, because MELT-numt calls exhibited only 67% overlap with the Tangram-numt or DINUMT calls. For VEI, Mobster-vei had the highest precision (100%) and recall (~ 90%) in the simulated data (Fig. 3c).

### Evaluation of algorithms with long read data

**Fig. 4:**
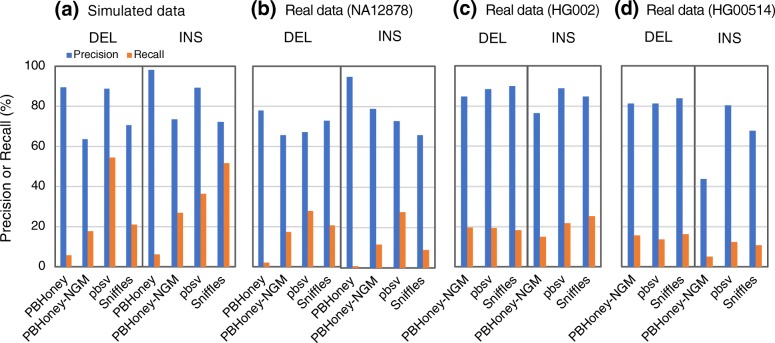
Precision and recall of SV detection algorithms with long read data. Precision and recall determined with the Sim-A-PacBio simulated data (**a**), the NA12878 real datasets (**b**), the PacBio-HG002 real data (**c**), and the PacBio-HG00514 real data (**d**). For the NA12878 data, the mean values of the results obtained with the three NA12878 long read datasets (PacBio-data1 to PacBio-data3) are indicated

We evaluated the performances of three SV detection algorithms with long read data, including PBHoney [22], Sniffles [55], and pbsv [56]. We also added a modified PBHoney algorithm (PBHoney-NGM), which used NGM-LR as alignment tool (see the “Methods” section). To generate a simulated dataset of long reads, PacBio long reads (average 7.5–20 kb) aimed at 10× coverage were simulated with Sim-A using the PBSIM simulator [57] (Fig. 4, Additional file 1: Table S3). For real data, we used long read datasets from three individuals: NA12878 (PacBio-data1 to PacBio-data3), HG002 (PacBio-HG002), and HG00524 (PacBio-HG00524) to determine precision and recall (Additional file 1: Table S3). pbsv achieved the highest precision and recall in DEL calling with the simulated data (Fig. 4, Additional file 3: Tables S5-S10 for the numerical data). Overall, however, the three algorithms exhibited similar accuracy in the real data, especially in the HG002 data. Although the input datasets used for evaluation of short read-based and long read-based algorithms were different, we compared the evaluation results of these three detection algorithms with those of short read-based ones (Figs. 1 and 2, Additional file 1: Figures S3–S5 and S8–S11). The long read-based algorithms exhibited good performances in calling short DELs (DEL-SS and DEL-S) and INSs despite the lower coverage of the long read data (10×) than that of the short read data (30×).

### Effect of different properties of read data on detection accuracy

We examined how read and library characteristics affect the precision and recall of SV calling among algorithms with relatively high precision and/or recall for each type and each size range. We generated datasets with different read lengths (100 bp, 125 bp, and 150 bp), read coverage (10×, 20×, 30×, and 60×), and library insert size (400 bp, 500 bp, and 600 bp) and evaluated the SV calling accuracies of the algorithms with these datasets (Additional file 2: Figure S13).

Changes in read coverage prominently affected recall and precision (see Additional file 1: Tables S12 and S13 for the summarized and statistical results). Data with higher coverage exhibited higher recall due to an increased number of signals including discordant reads and split reads. Interestingly, for many algorithms data with higher coverage resulted in lower precision than data with lower coverage when compared at the same threshold of RSS (as representative examples, see Additional file 2: Figure S13-A, S13-N, S13-X, S13-Z, S13-AJ, S13-AN, S13-AS, and S13-AU). In many cases, the precision using high-coverage data was comparable to that with lower coverage when the threshold values of RSS were increased (Additional file 2: Figure S13-M, S13-T, S13-X, S13-Y, S13-AB, S13-AD, S13-AH, S13-AL, S13-AN, S13-AP, S13-AR, and S13-AU). These results suggest that increasing the read coverage results in an increased number of spuriously aligned reads that lead to miscalling of SVs. In contrast to read coverage, neither read length nor insert size greatly affected recall and precision. We noted overall moderate effects on recall and precision for INS calling, while larger insert sizes led to greater than 10% decreased recall for DEL calling for several algorithms including BreakDancer [30], DELLY, inGAP-sv, Meerkat [58], and RAPTR-SV [59] (Additional file 1: Tables S12 and S13).

### Accuracy for calling breakpoints, sizes, and genotypes of SVs

We evaluated the accuracy with which each algorithm called breakpoints (BPs) and SV length (both calculated in root mean squared errors, RMSEs) using the Sim-A data (Additional file 3: Table S14; also see the “Methods” section for RMSEs). BreakSeek [60], BreakSeq2 [61], CREST [62], DELLY, GRIDSS, PBHoney-NGM, pbsv, SvABA, SVseq2 [63], and Wham achieved the highest accuracy (< 60-bp RMSE) for calling BPs for all size ranges of the DELs and/or DUPs. CREST, Manta, FermiKit [64], Pamir [65], pbsv, SVseq2, SoftSearch [66], Wham, and the specific INS detection algorithms (MEI and NUMT algorithms) exhibited the highest accuracy (< 10-bp RMSE) for calling INS BPs. Most algorithms that called BPs accurately used the split reads-based or assembly-based methods whereas algorithms only using the read depth-based alone approach exhibited poor BP resolution. BreakSeek, BreakSeq2, CLEVER, CREST, DELLY, FermiKit, GASVPro [67], GRIDSS, inGAP-sv, laSV [68], Lumpy, Manta, PBHoney-NGM, pbsv, PRISM [69], SvABA, SVseq2, and Wham provided higher accuracy (< 100-bp RMSV) for lengths of called DELs and/or DUPs, and most of these algorithms used the read pair-based or assembly-based method. These results suggest that the basic method used in SV detection algorithms affects the resolution of the called BPs and sizes.

Twenty-two algorithms used in this study call the genotypes or copy number associated with the detected SVs. We determined the precision and recall of the SV genotypes called with these algorithms using the Sim-A and NA12878 real datasets (Additional file 1: Figure S14 and Table S15). In the real datasets, only 335 DELs and 120 DUPs with specified genotype information were available. For the real DEL data, most algorithms exhibited > 95% precision. In contrast, most of the called DUPs did not match the 120 reference DUPs, limiting interpretation (Additional file 1: Table S15). For the simulated DEL data, Manta, Lumpy, Pindel, and ERDS [70] exhibited top performance in terms of both precision (> 90%) and recall (> 1900 TPs). PennCNV-Seq, CNVnator, BICseq2 [71], and readDepth exhibited high precision (> 89%) and recall (> 800 TPs) for the DUP data. For the INS data, Manta achieved the best performance, with > 97% precision. We note that algorithms with high performance genotype calling are also algorithms with good SV detection precision and recall.

### Run time and memory consumption

**Fig. 5:**
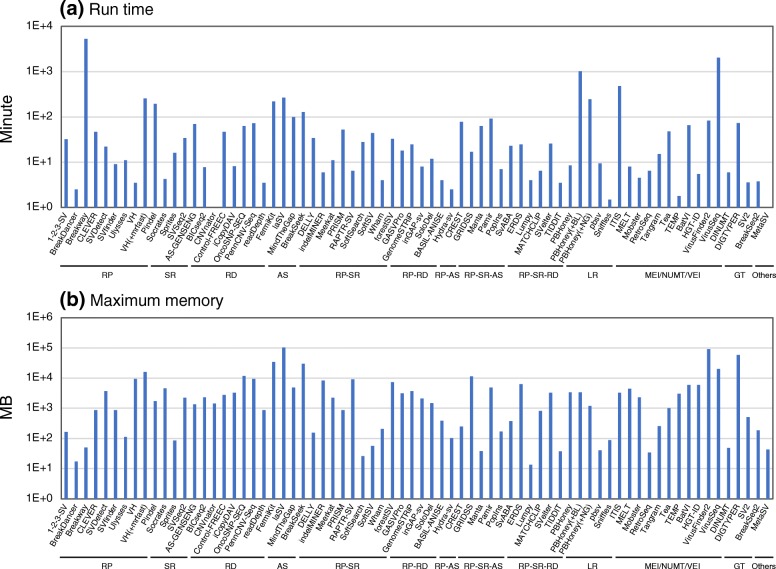
**a**, **b** Run time and memory consumption for SV detection algorithms. A bam or fastq files of the reads aligned to the NA12878 chromosome 8 (NA12878 data1 or PacBio-data1) was used as input data, and GRCh37 chr8 fasta file was used as reference. Each of the indicated algorithms was run using a single CPU. For VH (VariationHunter) and PBHoney, the data obtained together with the run of the indicated alignment tools (BL, BLASR; NG, NGM-LR) are also shown. For MetaSV, run time and maximum memory without those spent on Pindel and the other required tools are indicated. The algorithms were categorized according to the methods used to detect SV signals (RP, SR, RD, AS, LR, MEI/NUMT/VEI, and others) and their combined methods (RP-SR, RP-RD, RP-AS, RP-SR-AS, and RP-SR-RD)

Figure 5 shows run time and maximum memory per CPU for each SV detection algorithm, which were determined with 30× short read data (10× for long reads) of the NA12878 data1 that were aligned to the NA12878 chromosome 8 (146 Mb). SV detection algorithms directly using fastq read files (FermiKit, laSV, MinTheGap, Pamir, ITIS, and VirusSeq), many of which use the assembly method, exhibited long run time and large memory consumption. Algorithms requiring specific alignment tools, including VariationHunter [72] and long read-based algorithms, took longer run time than the standard algorithms using BWA. Pindel, known as a popular algorithm, also took longer run time although it exhibited good SV calling accuracy. Many of algorithms using the read depth method or detecting viral element insertions consumed larger memory than the others.

### Systematic identification of pairs of algorithms showing high accuracy in their overlapping, called SVs

**Fig. 6:**
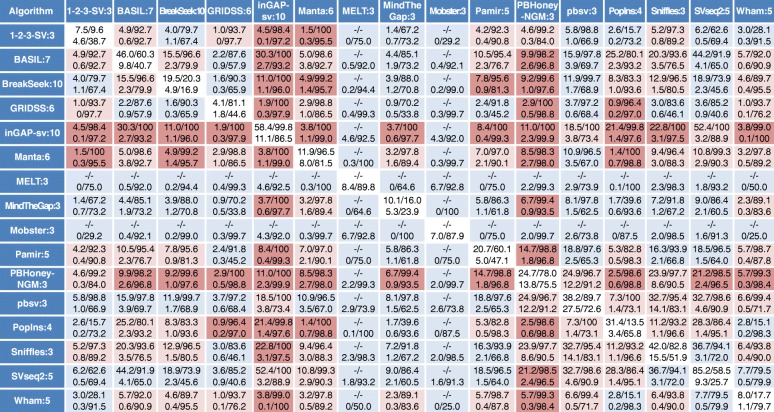
Recall and precision of SVs commonly called between a pair of SV detection algorithms for the INS category. INSs, called from the indicated algorithms, were filtered with the minimum number of reads supporting the called SVs, indicated with the suffix number of the algorithm name. The INSs overlapping between the filtered SV sets from a pair of the indicated algorithms were selected, and the recall and precision of the selected INSs were determined. Recall and precision percentages are presented with an intervening slash, and the recall/precision values for the simulated and real data are indicated in the upper and lower lines of each cell, respectively. Results for the real data represent the mean values of the values determined with four different NA12878 datasets (three PacBio datasets for long reads). The recall/precision values for the individual algorithm are indicated with blue letters and a white background. The data contained in the top 20th percentile of the combined precision scores (see the “Methods” section for details) for the simulated and real data are highlighted with a red background, and the next data contained in the top 21st to 50th percentile of the combined precision scores are shown with a pale red background. “–” indicates undetermined data

The above results revealed that the precision and recall with which a given algorithm calls SVs varies widely and depends on the types and size ranges of the SVs. However, few algorithms could call SVs with high precision, especially for DUP, INS, and INV of the real data, although the real dataset is likely to be incomplete (i.e., there are unidentified true SVs not present in our reference SV set). Several studies have taken the strategy of selecting SVs that are commonly called by multiple algorithms to increase the precision of the called SVs [13, 14, 24–29]. However, there has been no systematic investigation into optimal strategies to combine the results of multiple algorithms. We selected a total of 51 algorithms (12–38 algorithms for each SV type and size range) that exhibited relatively high precision and recall [the sum of recall (or precision) of the simulated and the NA12878 real data is > 10 for INS and INV or > 30 for the other types of SVs] for each type and each size range, and determined the precision and recall of the SVs that were commonly called for each combination of pairs of algorithms (Fig. 6 for INS and Additional file 1: Figures S15–S22 for DEL, DUP, and INV, also see Additional file 3: Table S16). The set of SVs called in common by two algorithms was more precise than the SVs called with either algorithm alone, as expected, yet this came at the cost of decreased recall. The degree of increased precision and decreased recall was varied depending on the algorithm combination. Combinations of algorithms that yielded more precise calls for a given type and size range of SV in both the simulated and real data are highlighted (Fig. 6 and Additional file 1: Figures S15–S22). We calculated the mean precision and recall values of overlapped calls between pairs of algorithms for each SV category (Additional file 1: Figure S23, Additional file 3: Table S17). As expected, high precision in the overlapped calls was often observed in pairs containing an algorithm exhibiting high precision by itself. Interestingly, however, several algorithms with a moderate level of precision in an SV category yielded higher precision in their overlapped calls. Examples of such good “team players” include CREST and VariationHunter in the DEL category and BASIL-ANISE [73] and BreakSeek in the INS category, each of which showed over twofold increase in combination with another algorithm.

**Fig. 7:**
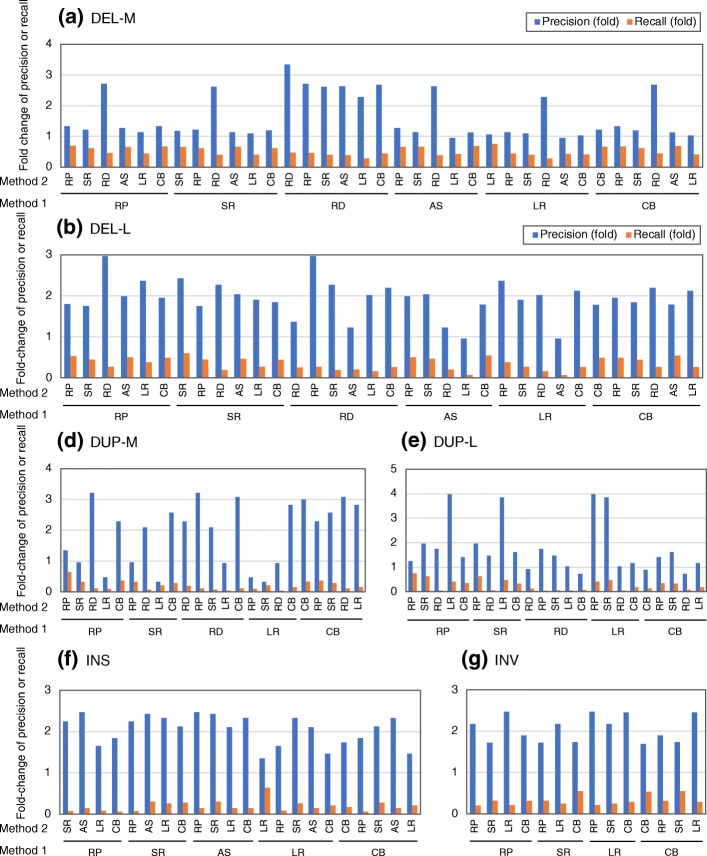
Increased or decreased rates of precision and recall of overlapped calls between various SV detection methods. Precision and recall values of overlapped calls between pairs of algorithms based on the indicated six different methods were determined for different SV categories (DEL-M (**a**), DEL-L (**b**), DUP-S (**c**), DUP-M (**d**), DUP-L (**e**), INS (**f**), and INV (**g**)) using four sets of NA12878 real data. The mean values (presented in Additional file 3: Table S18 in detail) were summarized based on pairs of methods (method 1 and method 2) by calculating the fold increase of precision or recall of overlapped calls relative to those for method 1 alone. RP, method using read pairs-based signal; RD, method using read depth-based signal; SR, method using split (soft-clipped) reads-based signal; AS, assembly-based approach; LR, method using long reads, CB; combined method using two or more methods out of RP, SR, RD, and AS

We then examined how precision and recall change when combining algorithms across the six SV detection methods, including RP, SR, RD, AS, LR, and CB (Fig. 7 and Additional file 3: Table S18). The DEL-calling precision increased less than the other types of SV because precision was already high. In general, combinations of algorithms from two different method class led to higher precision but lower recall than two algorithms using the same methods (mean fold change of precision: 1.63× for the same method and 1.82× for different methods; mean fold change of recall, 0.5× for the same method and 0.33× for different methods) (Fig. 7). These results suggest that combining algorithms from two different methods is a better strategy for obtaining an accurate representation of SV than using two algorithms of the same class. However, the results also suggest that the importance of obtaining overlapping SV calls with high precision and high recall to select good pairs of algorithms, irrespective of the combination of methods used in the algorithms.

## Discussion

No previous study has comprehensively compared the accuracies of existing SV detection algorithms. While papers describing new SV detection algorithms often include some benchmarking, they have done so using only a limited number of comparator algorithms. One recent study has compared the performances of existing seven MEI detection algorithms [74], and the results are well correlated with our evaluation results of MEI detection algorithms. Despite the overall consistency in accuracy rank of algorithms between the datasets (Additional file 1: Figure S12), the recall values for the real data were overall low relative to those for the simulated data. This would be in part due to the presence of overlapping redundant SVs in the NA12878 reference SV data, because the DGV data is derived from multiple sources of studies. Alternatively, several falsely detected SVs might be included in the reference set. In addition, lower levels of precision observed in the real data, especially for DUP and INV calls, would in part be due to a number of unidentified DUPs/INVs absent from the NA12878 reference SV dataset. More elaborate refinement, involving experimental validation, of the NA12878 SV reference data should be made in the future. Despite these shortcomings, the recall and precision values for the real data can be considered as relative values for ranking the relative performances of the algorithms.

Based on our evaluation results, we list the algorithms exhibiting higher precision and recall values for both the simulated and NA12878 real datasets (Table 1, see also Additional file 1: Table S19 for an extended list), although this list can be changed depending on what level of precision or recall is required. It shows the top 2–7 (the top 30% for Table S19) algorithms for each category exhibiting high values of the sum of the normalized *F*-measures of the simulated and real data and exhibiting short run time (< 200 min in Fig. 5). Overall, GRIDSS, Lumpy, SVseq2, SoftSV, and Manta show good performances in calling DELs of diverse sizes. TIDDIT [75], forestSV [76], ERDS, and CNVnator call large DELs well whereas SV detection algorithms using long reads, including pbsv, Sniffles, and PBHoney, are good at detecting small DELs. For DUP detection, good choices include Wham, SoftSV, MATCHCLIP, and GRIDSS. CNVnator, ERDS, and iCopyDAV [77] achieve good performances in calling large sizes of DUPs. For INSs, MELT, Mobster, inGAP-sv, and SV detection algorithms with long read data would effectively call reliable variants. AS-GENESENG, Control-FREEC, OncoSNP-Seq, and GenomeSTRiP may more accurately detect SVs in other types of applications, such as somatic SV detection or SV calling with whole exome sequencing data or multiple sample data because these algorithms have been more intensively designed for such applications. We also listed the poor performing algorithms in Table S20 in Additional file 1.

**Table 1:** List of tools providing good SV calling results for both the simulated and NA12878 real datasets

SV type	Tools	Simulated data		Real data		nF^*1^
		Precision	Recall	Precision	Recall	
DEL	GRIDSS	98.9 (5)	86.6 (2)	87.6 (7)	28.9 (2)	3.57 (1)
	Lumpy	99.1 (4)	81.4 (6)	87.1 (8)	26.1 (4)	3.41 (2)
	SVseq2	96.2 (11)	86.1 (3)	75.7 (17)	24.9 (5)	3.28 (3)
	SoftSV	96.8 (10)	83.6 (4)	80.2 (13)	23.2 (8)	3.25 (7)
	Manta	95.9 (12)	83.1 (5)	74.2 (20)	24.3 (6)	3.21 (5)
	MATCHCLIP	99.4 (2)	71.7 (10)	91.6 (4)	20.9 (11)	3.12 (6)
	inGAP-sv	91.1 (18)	78.6 (7)	78.3 (14)	22.5 (8)	3.10 (7)
DUP	Wham	96.9 (4)	81.7 (4)	57.1 (4)	10.2 (5)	3.92 (1)
	SoftSV	84.2 (14)	67.8 (13)	47.3 (6)	14.3 (3)	3.91 (2)
	MATCHCLIP	87.6 (11)	77.5 (8)	58.0 (3)	9.9 (6)	3.79 (3)
	GRIDSS	91.1 (9)	77.9 (7)	58.4 (2)	9.6 (7)	3.78 (4)
	Manta	99.0 (1)	83.2 (1)	40.4 (9)	6.5 (11)	3.35 (5)
	SvABA	82.6 (15)	69.6 (11)	42.7 (8)	7.2 (9)	3.02 (6)
INS [Unspecified]	pbsv	89.7 (3)	38.2 (5)	72.7 (8)	27.5 (2)	6.68 (1)
	inGAP-sv	99.7 (1)	58.5 (2)	85.5 (2)	11.8 (3)	6.27 (2)
	Sniffles	74.8 (5)	52.5 (3)	65.9 (10)	9.0 (5)	5.08 (3)
	SVseq2	70.4 (8)	64.2 (1)	38.5 (19)	7.1 (9)	4.87 (4)
INS [MEI]	MELT	99.7 (3)	68.9 (3)	88.9 (1)	85.6 ^*2^ (1)	3.21 (1)
	Mobster	100 (1)	67.1 (4)	88.3 (2)	71.9 ^*2^ (2)	3.04 (2)
INV	DELLY	94.7 (8)	81.8 (4)	38.9 (4)	15.6 (2)	3.07 (1)
	TIDDIT	89.2 (14)	77.9 (8)	49.1 (1)	11.7 (5)	2.89 (2)
	1–2-3-SV	70.7 (19)	81.2 (5)	31.8 (9)	14.8 (3)	2.67 (3)
	GRIDSS	96.6 (6)	84.7 (3)	34.2 (8)	10.4 (7)	2.67 (4)

^*1^Sum of normalized *F*-measures of the simulated and the real data. Normalized *F*-measure = *F*-measure/the mean *F*-measure for the corresponding category

^*2^Provisional recall value: the number of true positives was calculated by dividing by the provisional number of reference MEIs (1350), which was estimated using the data from the 1000 Genome project

Ranks of tools for each result (precision, recall, or *F*-measure) are indicated within parentheses

In almost all cases, SVs called in common between multiple algorithms exhibit higher precision and lower recall than those called with a single algorithm, but the degree of the increased precision and the decreased recall varies based on the specific combination of algorithms, including both short read- and long read-based algorithms. Mills et al. examined the accuracy of overlapping calls between five methods and demonstrated that combining algorithms based on the same method increased precision, but the increase was lower than when combining algorithms based on different methods [14]. This is consistent with our observations. However, combining algorithms based on same methods gives a moderate increase in precision and less decrease in recall. Previous studies have selected SV calls overlapping between at least two sets from multiple SV call sets in order to increase precision [13, 14, 24–28]. However, this strategy could take overlapping calls from “bad” pairs of algorithms whose overlapping calls give only a small increase in precision with a considerable decrease in recall. It is promising, therefore, to iteratively merge the overlapping calls from the selected pairs of algorithms, giving high quality of overlapping calls, thereby generating an SV call set with high accuracy and recovery. Furthermore, the use of overlapped calls should also improve the accuracies of the BPs, sizes, and genotypes of the SVs because we can select the BPs/sizes/genotypes from algorithms providing higher accuracy for these SV properties, shown in this study.

## Conclusion

We evaluated the SV detection accuracy, including the precision of BPs, sizes, and genotypes of called SVs, of 69 existing computational algorithms using simulated and real data in terms of both precision and recall. This is the largest benchmarking study for genomic variant discovery performed to date. Our evaluation tests reveal that most algorithms exhibit their best performance for specific types of SV and, in several cases, for specific size ranges. These findings indicate that specific algorithms suitable for each type of and each size range of SV should be selected to obtain the desired results. Furthermore, systematic evaluation for overlapping calls from each combination of algorithm pairs demonstrates that several specific pairs of algorithms give a higher precision and recall for specific SV types and size ranges compared with other pairs.

## Methods

### WGS datasets

The simulated dataset Sim-A was generated with the VarSim simulator [37] and the GRCh37d5 reference, which contains 41.8 Mb of extra decoy sequences comprising of 61 sequences. VarSim introduced a total of 8310 SVs (3526 DELs, 1656 DUPs, 2819 INSs, and 309 INVs) with sizes ranging from 50 bp to 1 Mb, in addition to SNPs and short indels corresponding to 0.1% and 0.02% of the genome size, respectively, into simulated paternal and maternal haploid genomes, containing approximately 67% heterozygous alleles (Additional file 1: Table S4). The number of introduced SVs was larger and smaller than the number of SVs detected for an individual human genome in the 1000 Genome project [6] and the numbers of SVs identified from the NA12878 assembly generated with long reads [20], respectively. Eighty percent of the introduced SVs were derived from known SVs, and the remaining were derived from artificial novel SVs automatically generated by the VarSim simulator. The introduced known SVs in the Sim-A genome were derived from the DGV variant data contained in the VarSim package, and the sizes and chromosomal positions of the introduced SVs faithfully reproduced the corresponding DGV variants. The Sim-A read set generated from both the paternal and maternal genomes consisted of 125 bp of paired-end reads with 30× coverage and with 500 bp insert size with 100 bp standard deviation (Additional file 1: Table S3). A variety of read sets of Sim-A with different statics in read length (100 bp, 125 bp, and 150 bp), insert size (400 bp, 500 bp, and 600 bp), and coverage (10×, 20×, 30×, and 60×) were generated with the simulated paternal and maternal genomes of Sim-A using the ART simulator [78]. The simulated PacBio reads (Sim-A-PacBio) were generated with the simulated paternal and maternal genomes of Sim-A using PBSIM [57], which was conducted using the model-based mode with the following options: --depth = 10, --length-mean = 75,000, and --length-sd = 8000. The other simulated datasets (Sim-MEI, Sim-NUMT, and Sim-VEI) were generated with in-house scripts. The NUMT sequences (766 NumtS sequences) to be introduced were obtained from the UCSC Genome Browser site (https://genome.ucsc.edu), and the genome sequences of 669 human-infectious viruses, including herpes simplex virus and adenovirus, were obtained from NCBI (https://www.ncbi.nlm.nih.gov/genome/viruses/). The MEI sequences were obtained by similarity searches (minimum identity 90%, minimum coverage 10%) for Alu, LINE1, SVA, and HERVK mobile elements against human chromosome 1 with BLAST. The number of identified sequences from Alu, LINE1, SVA, and HERVK were 9548, 1663, 123, and 10, respectively. For Sim-MEI, 651 randomly selected sequences, in addition to SNPs and short indels corresponding to 0.1% and 0.02% of the genome size, respectively, were introduced into chromosome 17 from the GRCh37d5 reference (Additional file 1: Table S4). Similarly, 200 randomly selected NUMT sequences at least 100 bp long and 100 randomly selected VEI sequences were introduced into chromosome 17 to generate Sim-NUMT and Sim-VEI, respectively. To diversify the VEI sequences, 500 bp to 10 kb fragments were extracted from randomly selected regions of the virus sequences, and random artificial substitutions were made for 0–5% of the VEI nucleotide bases to be introduced. Using the simulated paternal and maternal chromosome 17 containing VEIs, NUMTs, or VEIs, simulated paired-end reads were generated with the ART simulator, as with VarSim. The read length, insert size, and coverage of the Sim-MEI, Sim-NUMT, and Sim-VEI read sets were the same as the Sim-A data (Additional file 1: Table S3).

The real datasets of NA12878, including Illumina HiSeq and PacBio RS data, were downloaded from DDBJ (http://www.ddbj.nig.ac.jp) and DNAnexus (https://platform.dnanexus.com/login). The NA12878 short and long read sets included four (data1 to data4) and three (PacBio-data1 to PacBio-data3) datasets from different sources or libraries, respectively (Additional file 1: Table S3). To determine Mendelian inheritance errors for SV calling, Illumina HiSeq WGS datasets of NA12891 and NA12892, which correspond to father and mother of NA12878, were also downloaded from DDBJ. The real datasets of HG00514, including Illumina HiSeq and PacBio RS data [36], and HG002 PacBio RS dataset from the Genome in a Bottle (GIAB) Consortium [79] were downloaded from DDBJ.

### Reference SV dataset for real data

A reference SV dataset corresponding to NA12878 was generated by combining the DGV variant data (the 2016-05-15 version for GRCh37) obtained from the Database of Genomic Variants (http://dgv.tcag.ca/dgv/app/home) with the PacBio SV data identified from the NA12878 assembly generated with long reads [20]. The DGV data contained 1127 DELs (28% of the total DELs) with < 1 kb and 3730 INSs (79% of the total INSs) with < 1 kb or undefined length. We removed these short DELs and INSs from the DGV data because the long read-/assembly-based data covers a higher number of these size ranges of DELs (6550) and INSs (13,131) and is likely to be more reliable than the DGV data. We further removed DELs, DUPs, and INVs with ≧ 95% reciprocal overlap (≧ 90% reciprocal overlap for > 1 kb variants) in the DGV and long read/assembly data, resulting in the removal of 450 variants in total. The merge of both the datasets was conducted by removing shorter ones of overlapped DELs with ≧ 70% reciprocal overlap, resulting in the inclusion of 1671 DELs, 979 INSs, 2611 DUPs, and 233 INVs specific to the DGV SV data. Although there were still many overlaps within this SV data, they were not removed, because we were unable to judge which sites were inaccurately defined SVs. All the SVs < 50 bp, except for INSs, were removed. In addition, a high confidence NA12878 SV set (2676 DELs and 68 INSs) of the svclassify study [80], which has been deposited in GIAB (ftp://ftp-trace.ncbi.nlm.nih.gov//giab/ftp/technical/svclassify_Manuscript/Supplementary_Information), was merged, resulting in inclusion of 248 DELs (7%) and 4 INSs (6%) as nonoverlapping variants. Furthermore, 72 experimentally verified nonredundant INV dataset from the studies with the long reads [20, 81] and the InvFEST database (http://invfestdb.uab.cat) was merged, resulting in inclusion of 41 unique INVs. For the HG00514 SV reference, a minimal 30 bp of HG00514 variants was extracted from nstd152.GRCh37.variant_call.vcf.gz, which was obtained at the NCBI dbVar site (ftp://ftp-trace.ncbi.nlm.nih.gov//pub/dbVar/data/Homo_sapiens/by_study/vcf) (Additional file 1: Table S4). Variants specified as “BND” type were removed, and variants specified as “CNV” were reassigned to both DEL and DUP as SV type. For the HG002 SV reference, a minimal 30 bp of variants was extracted from HG002_SVs_Tier1_v0.6.vcf, which was obtained at the GIAB download site (ftp://ftp-trace.ncbi.nlm.nih.gov//giab/ftp/data/AshkenazimTrio/analysis/NIST_SVs_Integration_v0.6) (Additional file 1: Table S4).

### SV calling with simulated and real datasets

The simulated and real datasets were each aligned with the GRCh37d5 reference using bwa mem to generate bam files. For Meerkat and Mobster, bam files were modified by adding XA tags and with removing hard-clipped reads to mimic bam files generated with bwa aln although later versions of these algorithms can use bam files generated using bwa mem. For Tangram, bam files were generated by aligning the read set with a reference containing a subset of mobile element sequences using Mosaik [82]. For VariationHunter, reads were aligned using mrfast [8] to generate divet files. PacBio long reads were aligned with blasr [83] for PBHoney and using NGM-LR [55] for PBHoney-NGM, Sniffles, and pbsv. These alignment data were used for calling SVs with all the algorithms, except for FermiKit, laSV, BatVI, MindTheGap, Pamir, and VirusSeq, for which read data was directly used. PBHoney-NGM was conducted with a custom PBHoney setting, obtained from Dr. Aaron Wenger at Pacific Biosciences (http://www.pacb.com/blog/identifying-structural-variants-na12878-low-fold-coverage-sequencing-pacbio-sequel-system/). For calling NUMTs and VEIs, we enabled Mobster, MELT, and Tangram to call NUMTs or VEIs by modifying their reference or input files, although these algorithms were originally designed to detect only MEIs (see Additional file 4: Supplementary methods for detail). Detailed explanations for calling SVs with each algorithm are provided in Supplementary methods in Additional file 4.

### Evaluation of the SV detection accuracy of SV algorithms

For DELs and DUPs, called SVs were divided into four and three fractions, respectively, depending on their size, and precision and recall were calculated for each SV-type and for each size range. Precision was calculated by dividing the number of truly called sites with the total number of called sites, and recall was calculated by dividing the number of truly called sites with the total number of corresponding reference SVs. The true positive (TP) calls were judged when the called DELs, DUPs, and INVs exhibited ≧ 80% reciprocal (60% reciprocal for ≦ 1 kb) and ≧ 50% reciprocal overlaps with the reference SVs for the simulated and real data, respectively, or when the BPs of the called INSs were placed within 200 bp of those of the reference INSs. We further determined the SV calls exhibiting Mendelian inheritance errors with the WGS datasets of NA12878, NA12891, and NA12892 trio. When the SV calls of the child NA12878 overlap with neither from the parent SV call sets (≦ 200 bp distance for INSs and ≧ 50% overlaps for the others), the corresponding sites were regarded as Mendelian inheritance errors. Because these sites could attribute to false negatives in parents, we used 1.7-fold coverage of parent WGS datasets relative to the child data to minimize false negatives in parents. Called DELs or DUPs were divided into size ranges and searched against the total DEL or DUP reference sets but not against the divided reference set for the corresponding size range, because the overlap-based search sometimes hits sites with out of the size range. When size-ranged DEL/DUP calls matched the reference, the matched calls were used as true calls for calculating precision for the corresponding size range; in contrast, for the calculation of recall, the matched calls were used for the size range of the matched reference site. INSs and DUPs are sometimes complementary [84] and could be confusedly called by several types of algorithms. Thus, to judge whether the called INSs are true, we also searched them against the reference DUPs when the called INSs had no matched INS references. When INS calls were matched with the DUP references, the number of hit was added to both the TP calls and the INS reference to calculate precision and recall, respectively. Similarly, called DUPs were also searched against the reference INSs. The precision and recall values for many algorithms varied depending on the RSS threshold values. For several algorithms (e.g., CNVnator, readDepth), information on RSS values was lacking and thus other information, such as read depth or scores, was converted to a provisional number of RSS value (see Additional file 4: Supplemental methods). To determine the best precision/recall points for each algorithm and for each SV category, we selected an RSS threshold at which the numbers of calls for an SV type approximates but does not exceed 90% of the corresponding simulated reference data or the expected SV number in an individual (DEL: 3500, DUP: 550, INS: 3000, and INV: 100, estimated from the previous studies).

### Evaluation of accuracy for BP, SV length, and genotype calls

To determine the accuracies of the called BPs and the called SV lengths for each algorithm and for each SV category, we calculated the root mean squared errors (RMSEs) using the results obtained with the Sim-A data (the formula used to calculate RMSEs is presented below). The genotyping accuracy (i.e., homozygous or heterozygous) of called SVs was determined with the Sim-A and the NA12878 real datasets. The reference data (Real-GT, Additional file 1: Table S4) for NA12878 were generated by merging the array-based CNV data (estd195, nstd22, and nest6) from the dbVar database (https://www.ncbi.nlm.nih.gov/dbvar). Genotyping of DELs/DUPs called with the depth-based SV detection algorithms, including AS-GENSENG, CNVnator, Control-FREEC, and readDepth, is described in detail in Supplementary methods in Additional file 4 in detail. Precision was calculated by dividing the number of correctly called genotypes with the number of truly called sites (Precision1) or with the number of truly called sites with genotyped information (Precision2), and recall was calculated by dividing the number of correctly called genotypes by the total number of the corresponding reference SVs.

### Evaluation of overlapped calls between pairs of algorithms

Based on the evaluation results for SV detection algorithms, we selected 51 algorithms (12–38 algorithms for each SV type and size range) that exhibited relatively high precision and/or recall [the sum of recall (or precision) of the simulated and the real data is > 10 for INSs and INVs or > 30 for the other types of SVs] for each type and each size range. First, we determined the optimal RSSs at which the sum of the precision and recall values was highest for each algorithm and for each category. Next, to increase recall, we selected specific test RSSs that were lower by a few points than the determined optimal RSSs. We expected that this setting of RSS could achieve higher accuracy in precision and recall for the overlapped calls and would be helpful for practical use. For every combination of algorithm pairs for each SV category, we selected overlapped calls with ≧ 60% reciprocal overlap between the call sets from the two algorithms (filtered with the specified RSS thresholds). Both the mean precision and mean recall values for the overlapped calls were calculated with the TP calls determined for each of the algorithm pair. The tested algorithms, except for MetaSV, were categorized into six groups based on SV detection methods (RP, SR, RD, AS, long-read (LR) and combined (CB)) that involved any combinations of RP, SR, RD, and AS, and the method-based results of the overlapped calls were summarized by determining the mean values.

### Statistical analysis for SV detection accuracy

Precision (Pr) and recall (Rc) were calculated as follows:

$$ \Pr =\frac{\mathrm{TP}}{\mathrm{Call}}\times 100 $$

$$ \mathrm{Rc}=\frac{\mathrm{TP}}{\mathrm{Ref}}\times 100 $$

where TP, Call, and Ref are the numbers of true positives, called SVs, and the corresponding reference SVs, respectively.

To determine the degree of variance in both precision and recall between the different library properties (e.g., different ranges in read length), the coefficient of variation (CV; the ratio of the standard deviation to the mean) in precision and recall was determined for each algorithm for each SV category. The determined CVs were further summarized for each SV category by taking the mean of the CVs of 6–18 algorithms belonging to the same SV category.

To determine the rank of precision of overlapped calls for each SV category, a combined precision score (cPr), in which the precision values both for the simulated and real data were integrated, was calculated as follows:

$$ \mathrm{cPr}=\frac{\Pr \left(\mathrm{sim}\right)\times \Pr \left(\mathrm{real}\right)}{\mathrm{mPr}\left(\mathrm{sim}\right)\times \mathrm{mPr}\left(\mathrm{real}\right)} $$

where Pr(sim) and Pr(real) are precision (%) of overlapped calls for the simulated and real data, respectively, and mPr(sim) and mPr(real) are the mean precision values (%) for the simulated and real data, respectively. These values were calculated using all the overlapped calls in each SV category.

To examine the consistency of the determined SV calling accuracies between the simulated and the five real datasets, the accuracy ranks of the algorithms were compared between SV types and/or datasets using the Spearman rank correlation coefficients. The accuracy of algorithms within a dataset was ranked with a modified *F*-measure (*F*) using the following equations:

$$ F=\frac{2\Pr \times \mathrm{Rc}\times \mathrm{Nrc}}{\left(\Pr +\mathrm{Rc}\times \mathrm{Nrc}\right)}\times 0.01 $$

where Pr, Rc, and Nrc are precision (%), recall (%), and the normalization index for an algorithm, respectively. Because the recall values for the real datasets were considerably lower than those for the simulated dataset due to an excess of overlapped reference SVs for the real data, we normalized the recall values between the simulated and real datasets with the normalization index. The normalization index is a constant value specific to the SV type to normalize recall values for the real data; its value were 2.9, 4.0, 2.4, and 2.4 for DEL, DUP, INS, and INV, respectively.

When the accuracies of the algorithms were ranked using the *F*-measures for two datasets, the Spearman rank correlation coefficients (*r*_*s*_) between the two datasets were determined as follows:

$$ {r}_s=1-\frac{6\sum {d_i}^2}{n^3-n} $$

where *d*_*i*_ is the difference between the *i*th algorithm’s ranks of each dataset, and *n* is the number of algorithms for either dataset.

The root mean squared errors (RMSEs) were calculated according to the following formula to determine the statistical errors of the called BPs and SV lengths for each algorithm:

$$ \mathrm{RMSE}=\sqrt{\frac{1}{N}\sum \limits_{i=1}^N{\left( Ci- Ri\right)}^2} $$

where *N* is the number of truly called SVs, *Ci* is a breakpoint (or SV length) of the *i*th truly called SV, and *Ri* is a breakpoint (or SV length) of the corresponding reference SV.

## Additional files

Additional file 1: **Figures S1-S12**, **Figures S14-S23**, and **Tables S1-S4, S12, S13, S15, S19, S20.** (PDF 1464 kb)

Additional file 2: **Figure S13.** Effect of read length, read coverage, and insert size on recall and precision for various SV algorithms. (PDF 2174 kb)

Additional file 3: **Table S5.** Recall and precision of SV-calling results with the simulated data (Sim-A, Sim-MEI, Sim-NUMT, Sim-VEI). **Table S6.** Recall and precision of SV-calling results with the real data (NA12978 data1 or PacBio-data1).** Table S7.** Recall and precision of SV-calling results with the real data (NA12978 data2 or PacBio-data2). **Table S8.** Recall and precision of SV-calling results with the real data (NA12978 data3 or PacBio-data3). **Table S9.** Recall and precision of SV-calling results with the real data (NA12978 data4 or PacBio-HG002). **Table S10.** SV calling results (recall, precision, Mendelian inheritance error, mean, and standard error) obtained with the four (or three) NA12878 real datasets. (including the numerical data of Fig. 3 and Additional file 1: Figures S3–S5). **Table S11.** Recall and precision of SV-calling results with the HG00514 real data. **Table S14.** Root mean squared errors of breakpoints (BPs) and lengths of called SVs for SV detection algorithms. **Table S16.** Recall and precision of SVs commonly called between a pair of SV detection algorithms with the simulated and the NA12878 real datasets. **Table S17.** Mean precision and recall of overlapped calls for each algorithm and for each SV category. **Table S18.** Fold change of precision and recall of overlapped calls between algorithm pair for the four (or three) sets of NA12878 real data. (XLSX 1162 kb)

Additional file 4: Supplementary methods. SV calling processes for 69 SV detection algorithms used in this study. (PDF 430 kb)

## Data Availability

The scripts used for the evaluation of algorithms’ performance and the simulated data (genome sequences and reference SV set) are available at https://github.com/stat-lab/EvalSVcallers [85]. All the sequence data used in this study were downloaded from DDBJ (http://www.ddbj.nig.ac.jp/) and DNAnexus (https://platform.dnanexus.com/login), with accession numbers shown in the supplemental information (Additional file 1: Table S3). The reference SV sets of NA12878 were constructed with the datasets downloaded from the Database of Genomic Variants (http://dgv.tcag.ca/dgv/app/home) and the long read-derived SV data [20]. The reference SV datasets of HG00514 and HG002 were downloaded from the NCBI dbVar site (ftp://ftp-trace.ncbi.nlm.nih.gov//pub/dbVar/data/Homo_sapiens/by_study/vcf) [36] and the GIAB download site (ftp://ftp-trace.ncbi.nlm.nih.gov//giab/ftp/data/AshkenazimTrio/analysis/NIST_SVs_Integration_v0.6) [79], respectively. The SV detection algorithms used in this study were obtained from the reference list in the supplemental information (Additional file 1: Table S1).

## Abbreviations

AS: Assembly
bp: Base pair
BP: Breakpoint
CB: Combined method
CNV: Copy number variation
DEL: Deletion
DGV: Database of genome variants
DUP: Duplication
GIAB: The Genome in a Bottle Consortium
HGSV: The Human Genome Structural Variation Consortium
indel: Short insertion and deletion
INS: Insertion
INV: Inversion
kb: Kilobase pair
LR: Long read
Mb: Megabase pair
MEI: Mobile element insertion
MIER: Mendelian inheritance error rate
NUMT: Nuclear insertion of mitochondrial genome
RD: Read depth
RMSE: Root mean squared error
RP: Read pairs
RSS: Reads supporting the called SVs
SNV: Single nucleotide variant
SR: Split read
SRA: Sequence read archive
SV: Structural variation
VEI: Viral genome insertion
WGS: Whole genome sequencing
