# Supplementary material for: Comprehensive evaluation of structural variation detection algorithms for whole genome sequencing
Source: Genome Biol. 2019 Jun 3;20:117. doi: 10.1186/s13059-019-1720-5 (PMC6547561; doi:10.1186/s13059-019-1720-5)
Supplement: Supplementary file 1 — Figures S1-S12, Figures S14-S23, and Tables S1-S4, S12, S13, S15, S19, S20. (PDF 1464 kb) [file 13059_2019_1720_MOESM1_ESM.pdf]

Table of contents in Additional file 1

Figure S1 – Outline of evaluation process for the performances of SV detection algorithms.
Figure S2 – Changes of precision and recall depending on the threshold number of reads supporting the called SVs for filtering.
Figure S3 – Precision and recall of DELs called using existing algorithms with the simulated and the NA12878 real data.
Figure S4 – Precision and recall of DUPs called using existing algorithms with the simulated and the NA12878 real data.
Figure S5 – Precision and recall of INSs and INVs called using existing algorithms with the simulated and the NA12878 real data.
Figure S6 – Precision and recall of called SVs based on SV calling methods.
Figure S7 – Mean precision and Mendelian inheritance error rate for SV detection algorithms.
Figure S8 – SV type-specificity of SV detection algorithms, determined with the HG00514 real data.
Figure S9 – Size range-specificity of SV detection algorithms for DELs and DUPs, determined with the HG00514 real data.
Figure S10 – Precision and recall of DELs called using existing algorithms with the HG00514 real data.
Figure S11 – Precision and recall of DUPs and INSs called using existing algorithms with the HG00514 real data.
Figure S12 – Spearman's rank correlation of SV detection accuracy between the datasets.
Figure S14 – Precision and recall of genotypes called with the simulated data.
Figure S15 – Recall and precision of SVs commonly called between a pair of SV detection algorithms for the DEL-SS category.
Figure S16 – Recall and precision of SVs commonly called between a pair of SV detection algorithms for the DEL-S category.
Figure S17 – Recall and precision of SVs commonly called between a pair of SV detection algorithms for the DEL-M category.
Figure S18 – Recall and precision of SVs commonly called between a pair of SV detection algorithms for the DEL-L category.

Figure S19 – Recall and precision of SVs commonly called between a pair of SV detection algorithms for the DUP-S category.

Figure S20 – Recall and precision of SVs commonly called between a pair of SV detection algorithms for the DUP-M category.

Figure S21 – Recall and precision of SVs commonly called between a pair of SV detection algorithms for the DUP-L category.

Figure S22 – Recall and precision of SVs commonly called between a pair of SV detection algorithms for the INV category.

Figure S23 – Mean precision and recall of overlapped calls between SV detection algorithms.

Table S1 – SV detection algorithms used in this study.

Table S2 – List of algorithms that did not work in our computational environment.

Table S3 – Whole genome sequencing reads used in this study.

Table S4 – Components of reference SVs for real and simulated datasets.

Table S12 – Effect of the length, insert size, and coverage of reads on precision and recall of SV detection algorithms (summarized data of Additional file 2: Figure S13).

Table S13 – Mean coefficient of variation of SV detection accuracy for each category of SV type and read property

Table S15 – Genotyping precision of SV detection algorithms.

Table S19 – Extended list of algorithms exhibiting good SV calling results in both the simulated and the NA12878 real datasets.

Table S20 – List of poor performing algorithms that cannot be recommended for SV detection with human WGS data.

Supplementary references – References cited in Tables S1, S2, and S4.

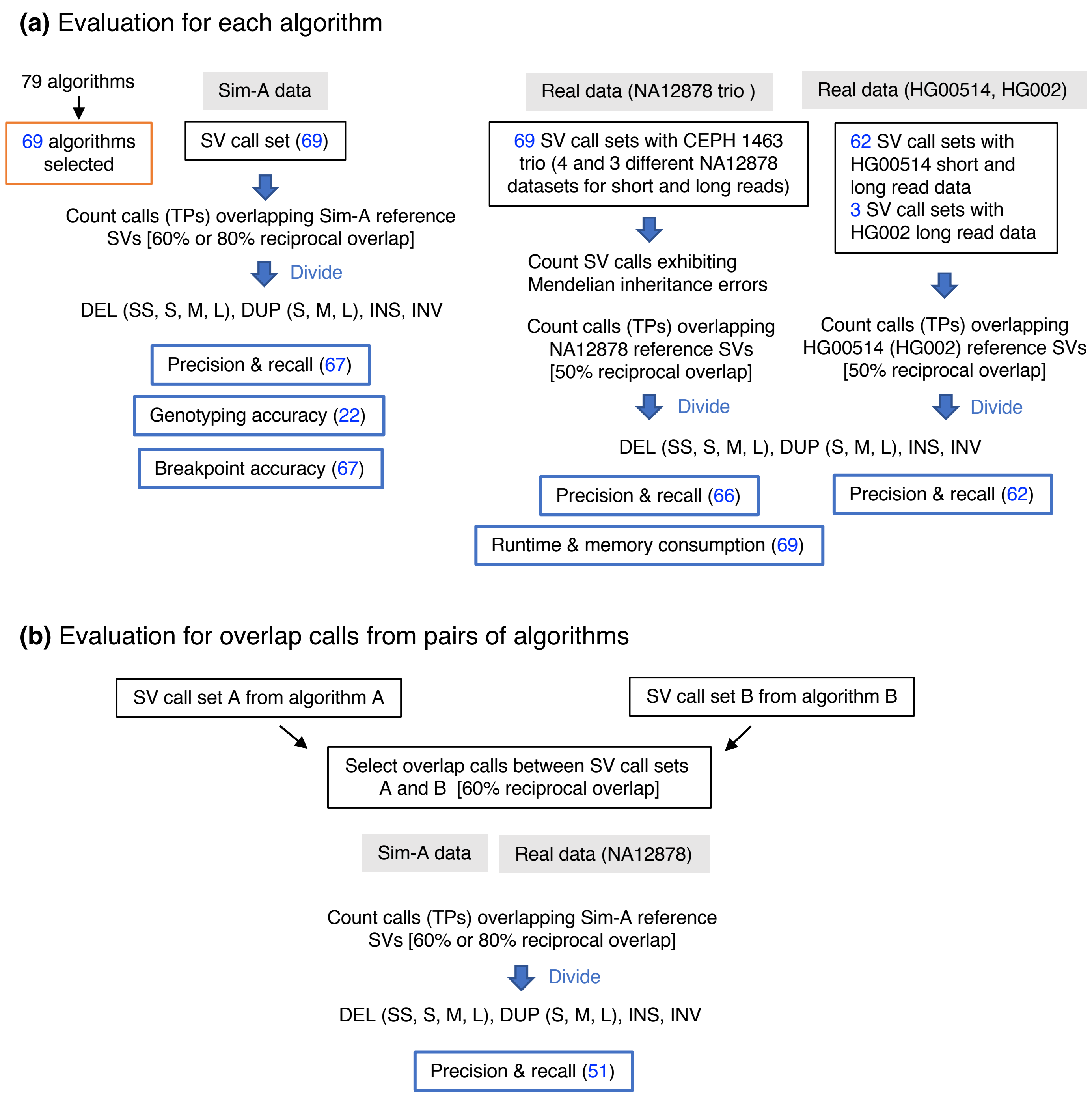

Figure S1. Outline of evaluation process for the performances of SV detection algorithms. Simulated data (Sim-A) and real datasets consisting of NA12878 trio, HG00514, and HG002 datasets were used for evaluation of the performance of single algorithm (**a**) and overlap calls from pairs of algorithms (**b**). For real data, Mendelian inheritance errors that were called only for the child NA12878 but not for the parents NA12891 and NA12892 were counted for each SV type for each algorithm. The mean precision, recall, and Mendelian error rates from four distinct NA12878 child datasets derived from different sequencing libraries were determined. For long read-based algorithms, the mean precision and recall values from three distinct NA12878 long read datasets were determined. In addition, each SV detection algorithm was also evaluated with HG00514 real datasets of short and long reads. The number of algorithms used in each analysis is indicated in blue. For evaluation for single algorithm, 69 algorithms, containing 2 genotype calling algorithms, were selected from available 79 algorithms. For evaluation of overlap calls, 51 algorithms out of the 69 algorithms were selected.

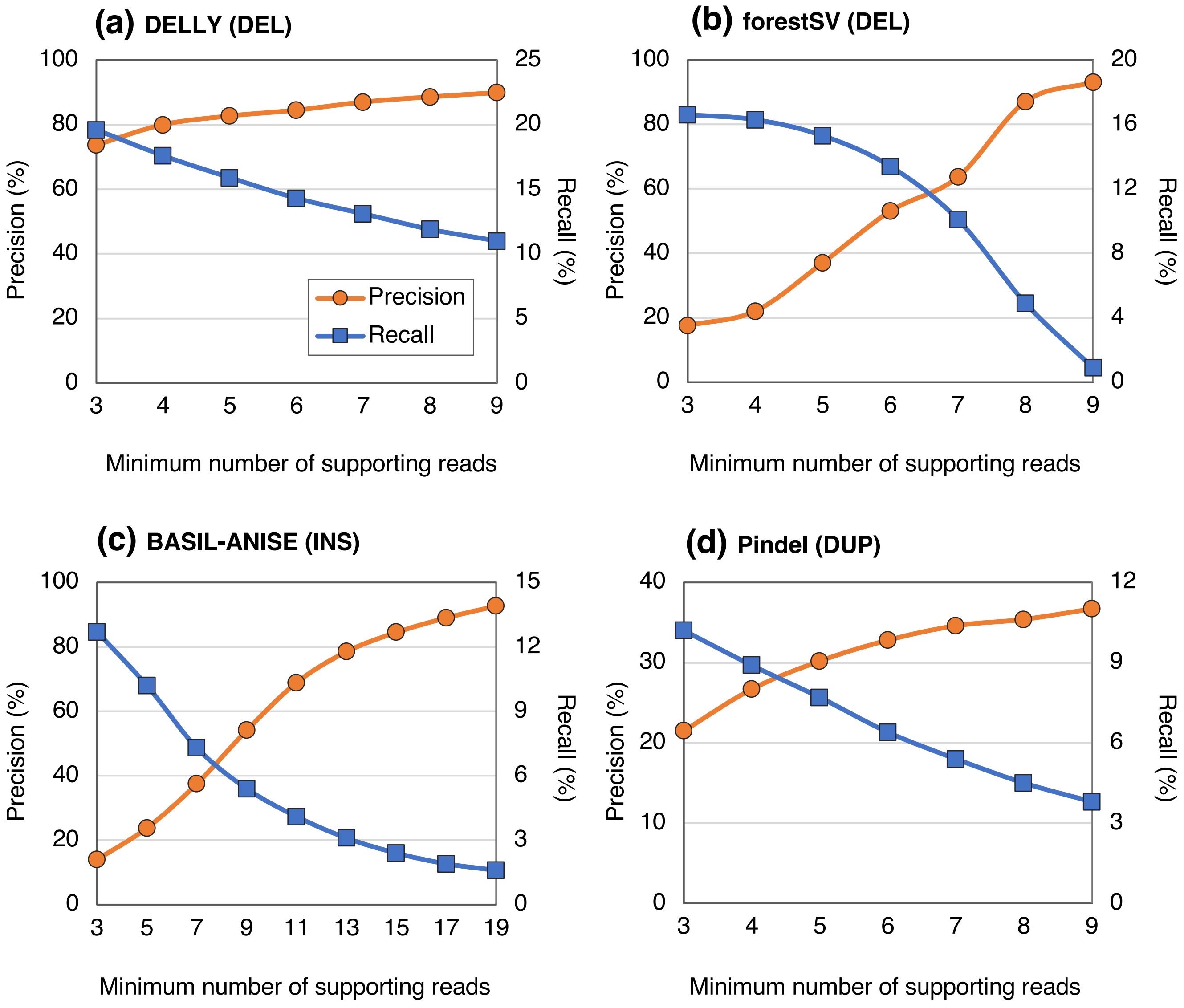

Figure S2. Changes in precision and recall depending on the threshold number of reads supporting the called SVs for filtering. (**a**) DELs called using DELLY with the NA12878 read data. (**b**) DELs called using forestSV with the NA12878 read data. (**c**) INSs called using BASIL-ANISE with the NA12878 read data. (**d**) DUPs called using Pindel with the NA12878 read data. Precision and recall percentages of the called SVs are indicated with the scales at the left and right sides on the y-axis, respectively, which were plotted against the minimum number of reads supporting the called SVs, indicated on the x-axis. As SVs with a lower number of supporting reads than the minimum number of supporting reads are removed, the minimum number of supporting reads functions as a filtering classifier to increase precision, despite decreasing recall.

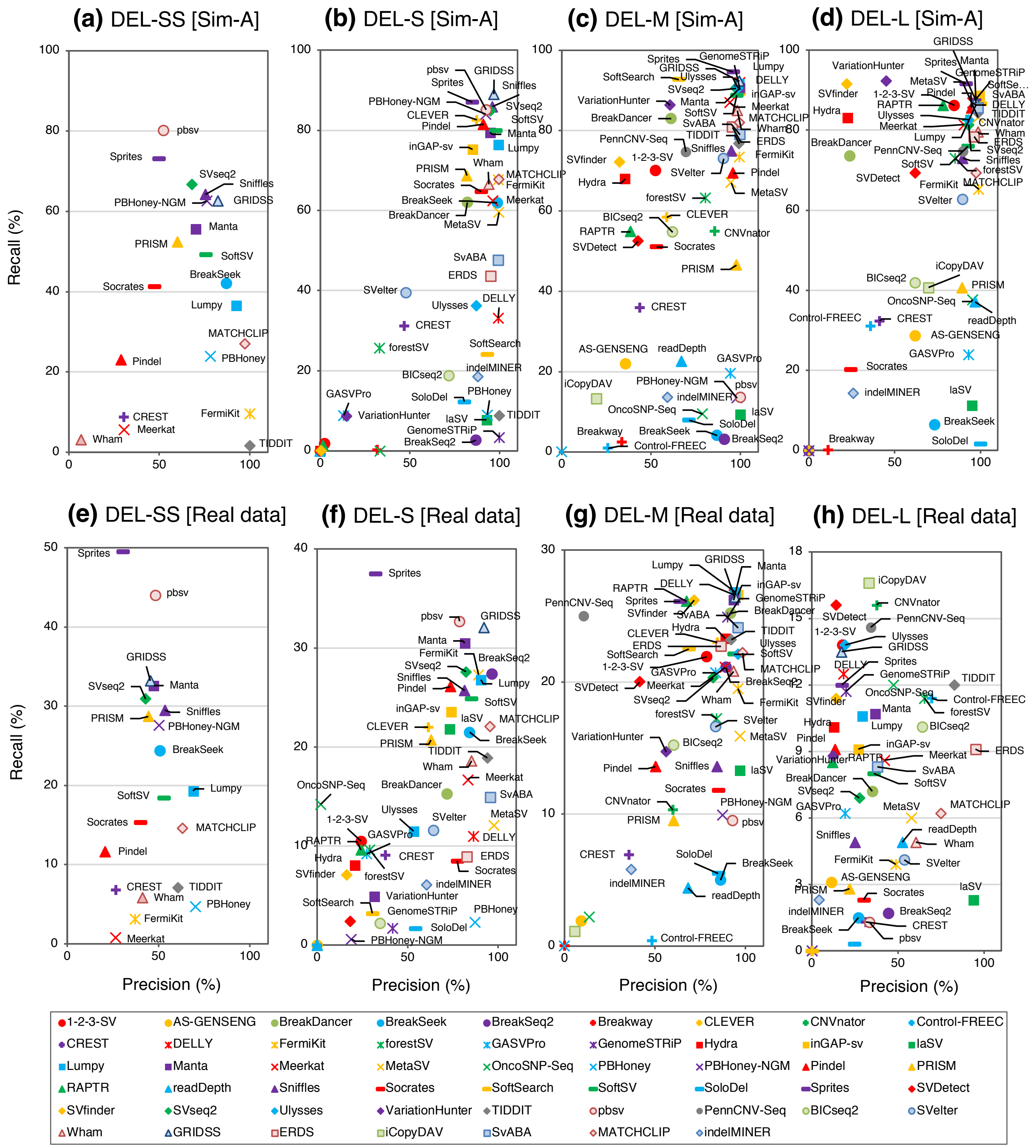

Figure S3. Precision and recall of DELs called using existing algorithms with the simulated and the NA12878 real data. DELs were called with the indicated algorithms and the simulated data (**a–d**) and the real data (**e–h**). DELs were categorized into four sets depending on the size-ranges; SS: 50–100 bp (**a,e**), S: 100 bp–1 Kb (**b,f**), M: 1 Kb–100 Kb (**c,g**), L: 100 Kb–1 Mb (**d,h**). The precision and recall percentages determined for the respective DEL call sets are indicated with the scales on the x-axis and y-axis, respectively. For the real data the mean values of the results obtained with the four NA12878 real datasets (data1 to data4) are indicated. Tools exhibiting a higher precision and a higher recall are placed toward the upper right corner.

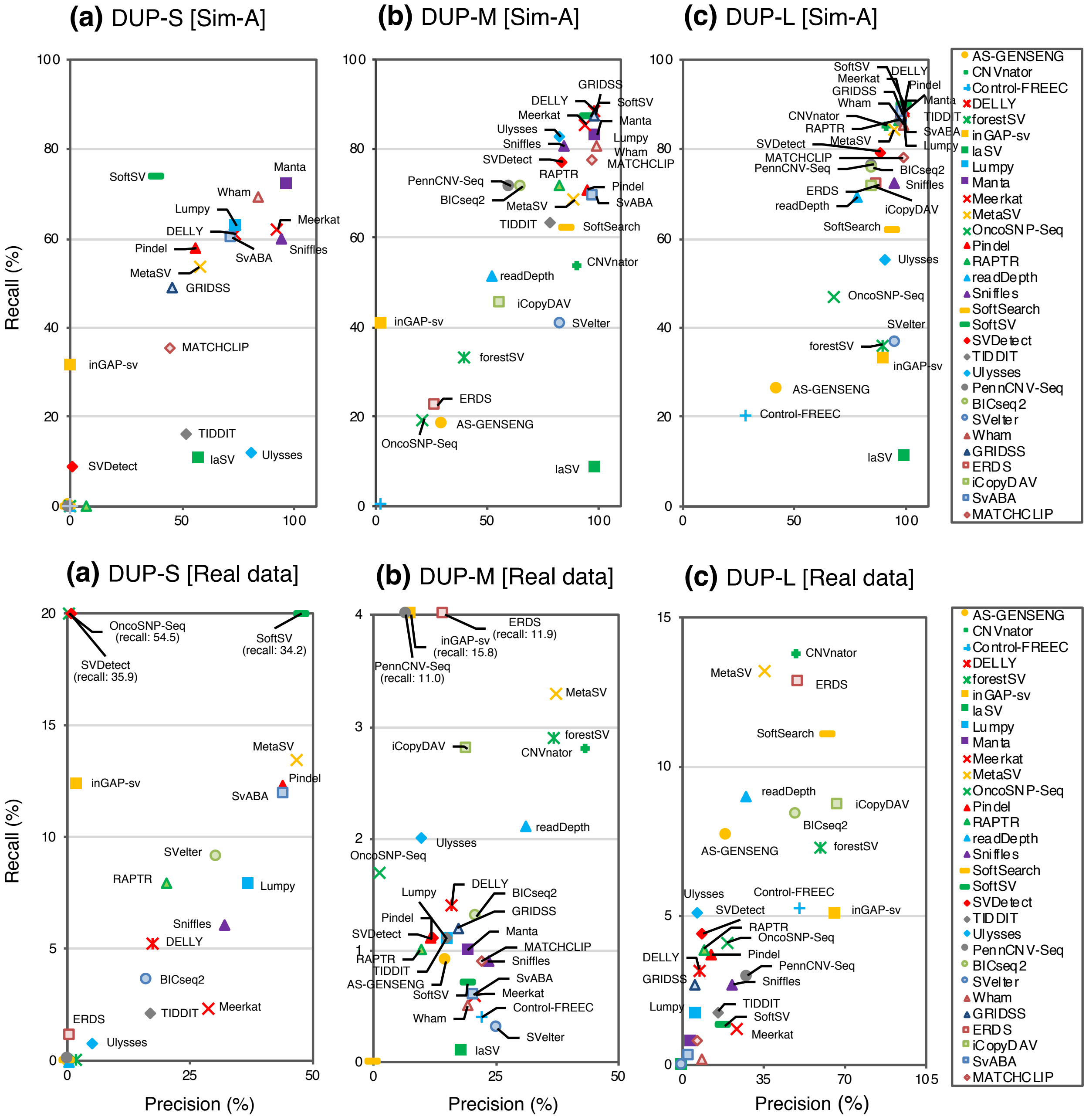

Figure S4. Precision and recall of DUPs called using existing algorithms with the simulated and the NA12878 real data. DUPs were called using the indicated algorithms and the simulated data (**a–c**) and the four NA12878 real datasets (**d–f**). DUPs were categorized into three sets depending on the size-ranges; S: 50 bp–1 Kb (**a,d**), M: 1 Kb–100 Kb (**b,e**), L: 100 Kb–1 Mb (**c,f**). The precision and recall percentages are indicated as in Figure S3.

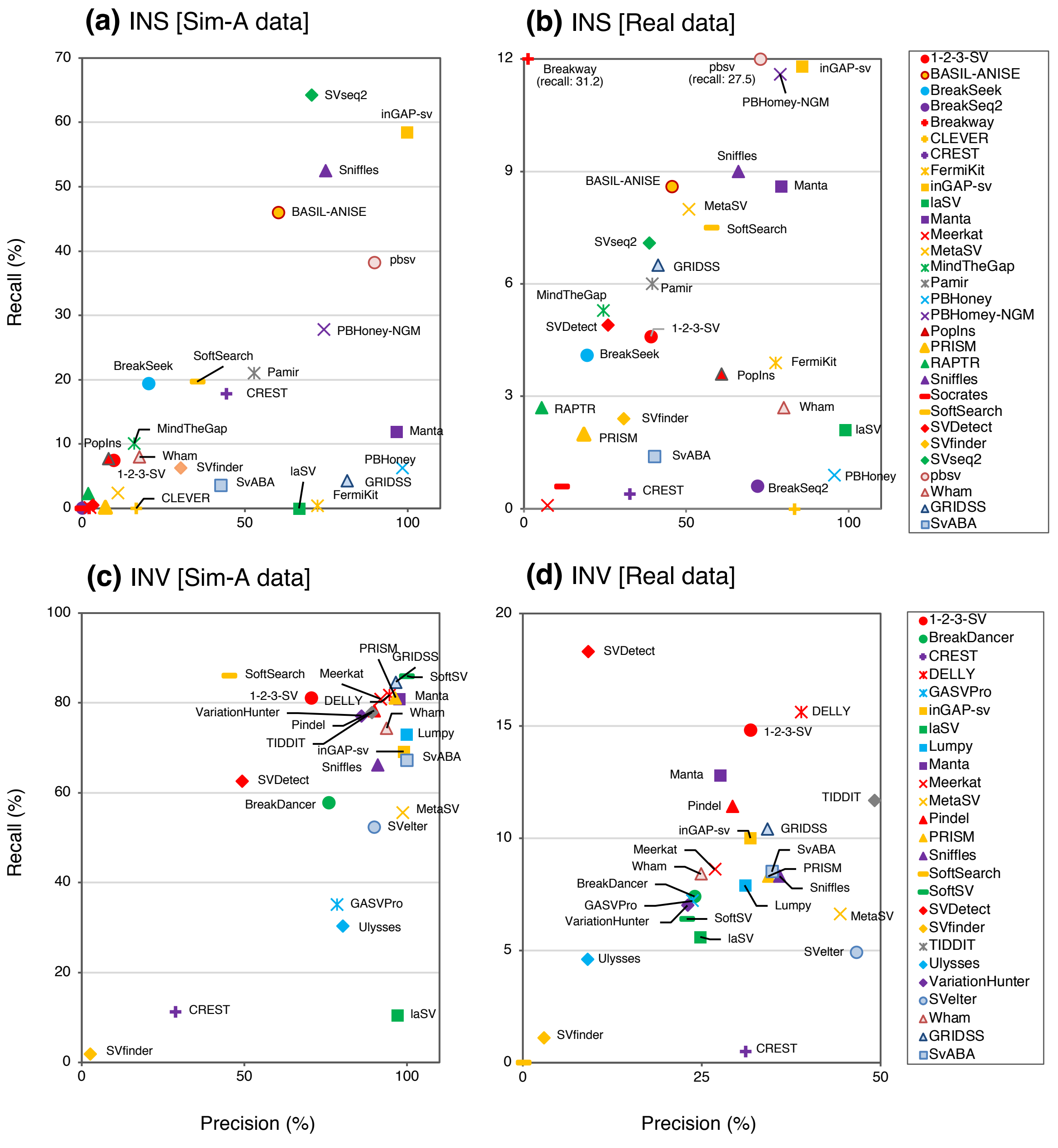

Figure S5. Precision and recall of INSs and INVs called using existing algorithms with the simulated and the NA12878 real data. INSs (**a**,**b**) and INVs (**c**,**d**) were called using the indicated algorithms and with the simulated (**a**,**c**) or the four NA12878 real datasets (three datasets for long reads) (**b**,**d**). The precision and recall percentages are indicated as in Figure S3. The data for real data represent the mean values of the results obtained with the four NA12878 real datasets.

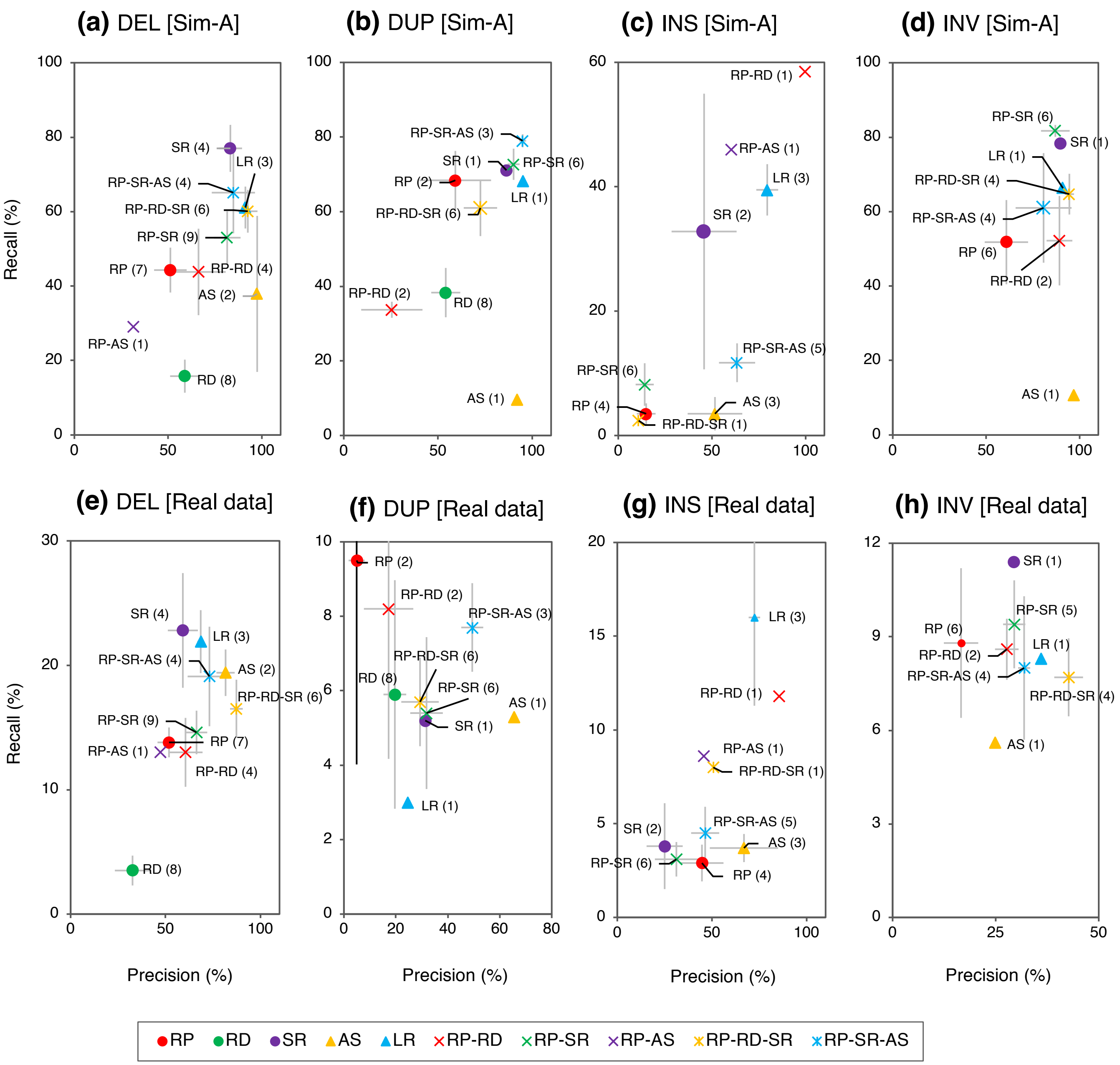

Figure S6. Precision and recall of called SVs based on SV calling methods. Precision and recall of DELs (**a,e**), DUPs (**b,f**), INSs (**c,g**), and INVs (**d,h**) were determined with the simulated data (**a–d**) and real data (**e–h**) using the algorithms indicated in Table S1 in Additional file 1. For the real data the mean values of the results obtained with the four (or three) NA12878 real datasets (data1 to data4) are indicated. The algorithms were categorized by the 10 methods: RP: read pair, RD: read pair, SR: split read, AS: assembly, LR: long read, RP-RD, RP-SR, RP-AS, RP-RD-SR, and RP-SR-AS. The hyphenated tools are combinations of RD, RD, SR, and AS. MetaSV was categorized into the RP-RD-SR method, and the data for BreakSeq2, which did not meet the criteria for these methods, was not used. The means of the precision and recall percentages of the algorithms categorized for each method are indicated with the scales on the x-axis and y-axis, respectively. The number of tools used for taking the means is indicated in parentheses for each symbol. The standard errors for precision and recall for each method are indicated with bars in light gray. Methods exhibiting a higher precision and a higher recall are placed toward the upper right corner.

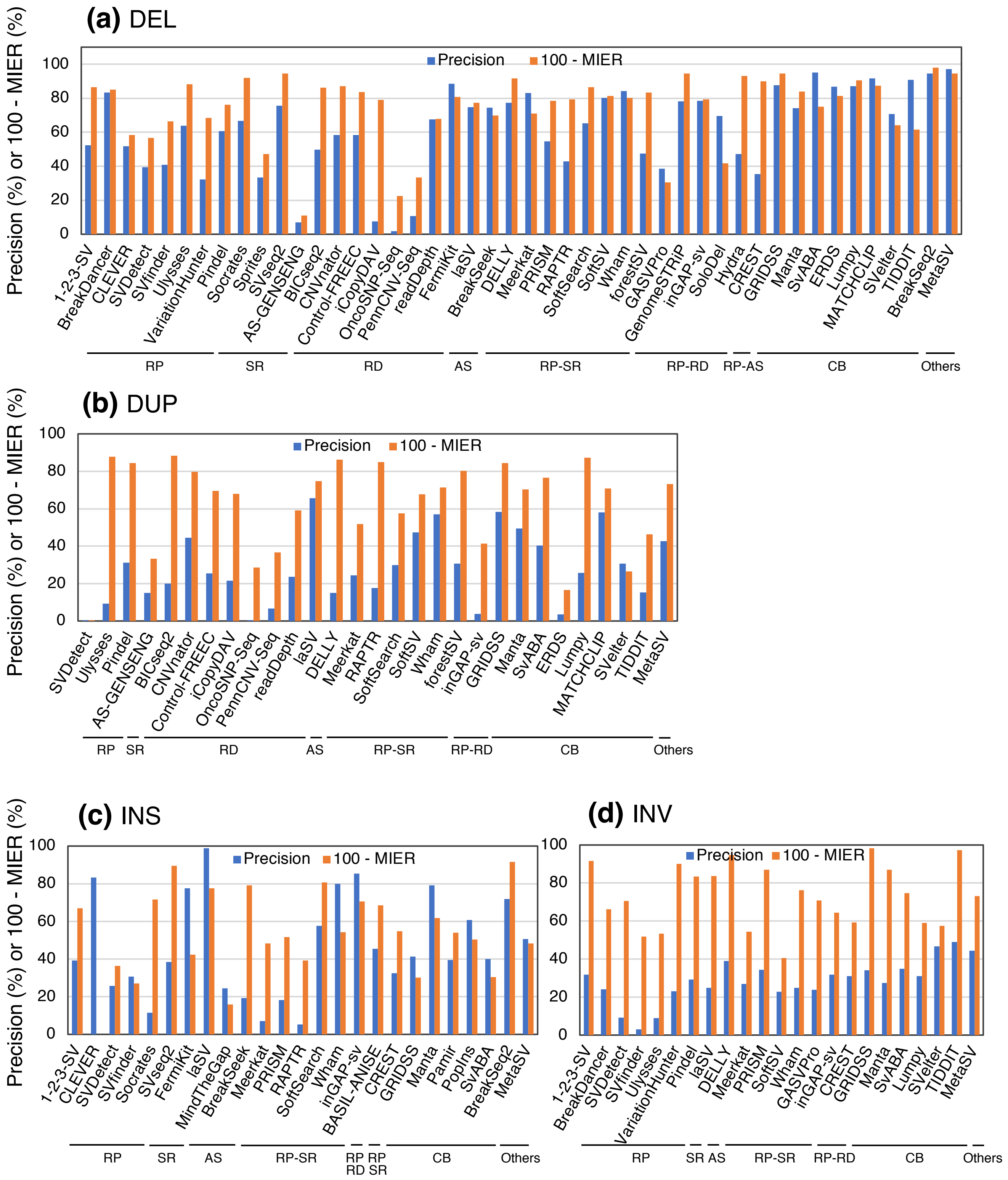

Figure S7. Mean precision and Mendelian inheritance error rate for SV detection algorithms. Mean precision and Mendelian inheritance error rates (MIER) of SV detection algorithms were determined for the different SV types, DEL (**a**), DUP (**b**), INS (**c**), and INV (**d**), using four sets of NA12878 real data. MIER is the percentage of Mendelian inheritance errors, which are the number of sites called only for NA12878 but not for the parents NA12891 and NA12892, in the number of the calls for the respective SV type. The mean precision and 100 – MIER are indicated with blue and orange bars, respectively. The algorithms were categorized according to the methods used to detect SV signals (RP: read pairs, SR: split reads, RD: read depth, AS: assembly, LR: long reads, and CB: their combined methods). The numerical data, including those for the other SV categories, are presented in Tables S6–S10 in Additional file 3.

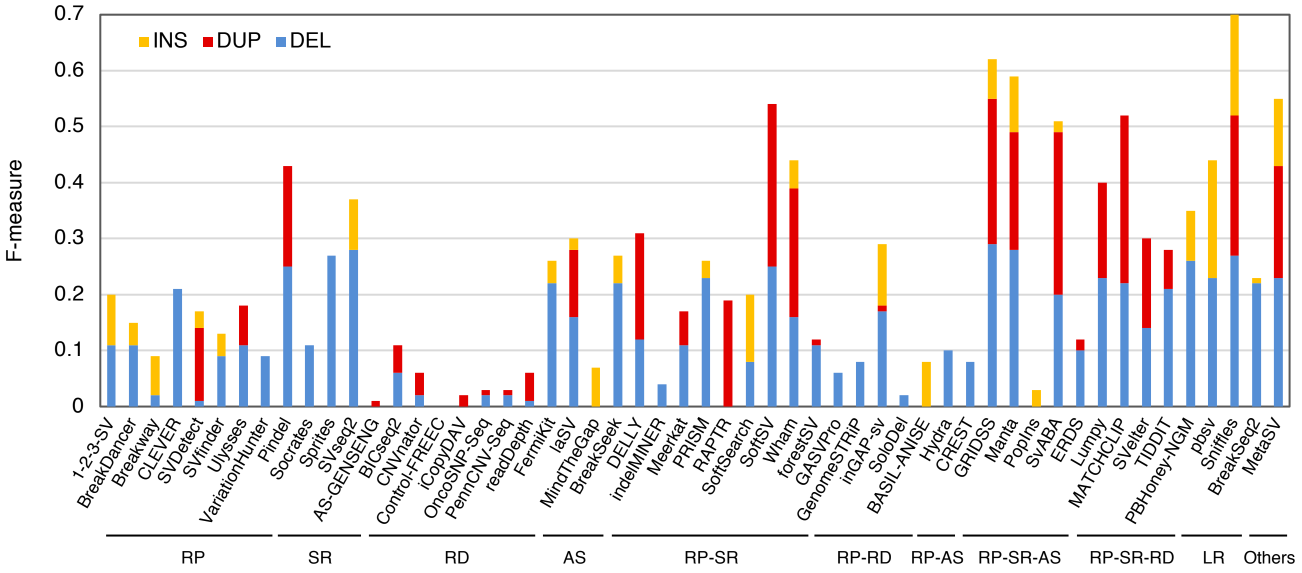

Figure S8. SV type-specificity of SV detection algorithms, determined with the HG00514 real data. Precision and recall of DELs, DUPs, and INSs were determined with the HG00514 real data. Modified F-measures (the combined statistics for precision and recall (see Methods for details)), are shown for the algorithms indicated with blue (for DEL), red (for DUP), and orange (for INS) bars. The algorithms were categorized according to the methods used to detect SV signals (RP: read pairs, SR: split reads, RD: read depth, AS: assembly, LR: long reads) and their combined methods (RP-SR, RP-RD, RP-AS, RP-SR-AS, and RP-SR-RD).

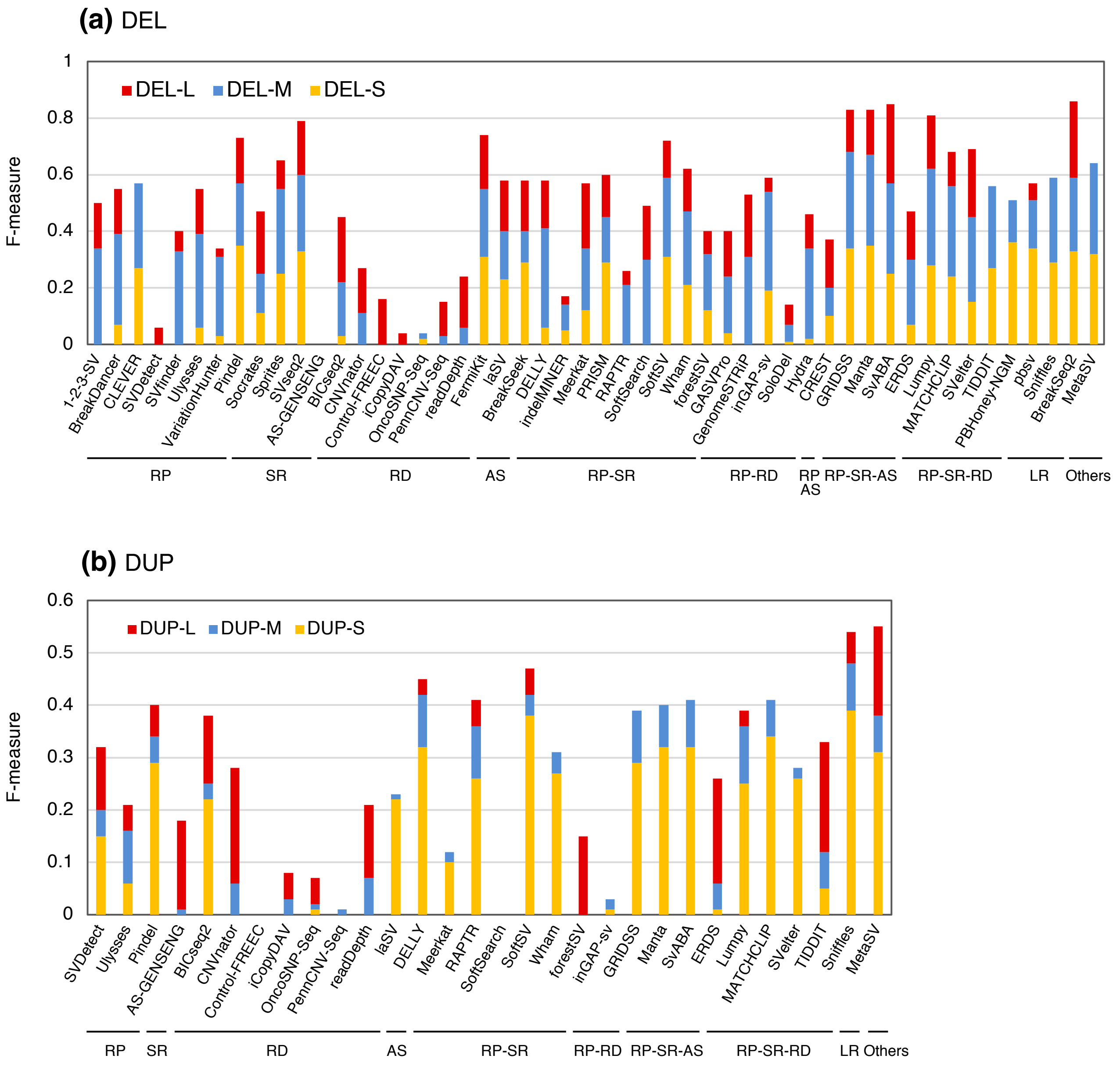

Figure S9. Size range-specificity of SV detection algorithms for DELs and DUPs, determined with the HG00514 real data. Precision and recall of each size range of DELs (a,b) and DUPs (c,d) were determined with the HG00514 real data. Modified F-measures (the combined statistics for precision and recall) are shown for the algorithms indicated with orange (for S: 100 bp–1 Kb). blue (for M: 1 Kb–100 Kb), and red (for L: 100 Kb–1 Mb) bars. The algorithms were categorized according to the methods used to detect SV signals, as in Figure S8.

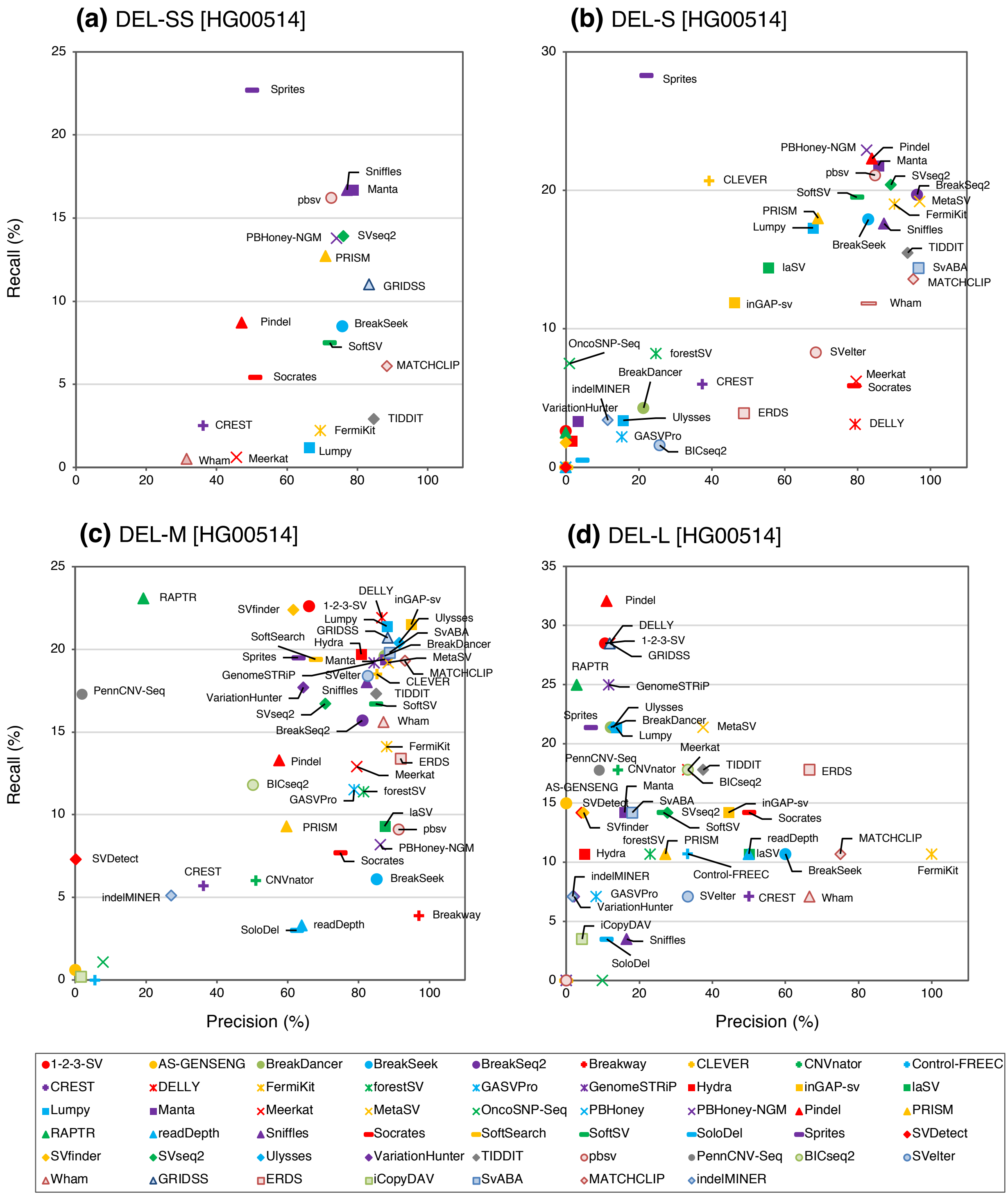

Figure S10. Precision and recall of DELs called using existing algorithms with the HG00514 real data. DELs were called with the indicated algorithms and the HG00514 real data. DELs were categorized into four sets depending on the size-ranges; SS: 50–100 bp (**a**), S: 100 bp–1 Kb (**b**), M: 1 Kb–100 Kb (**c**), L: 100 Kb–1 Mb (**d**). The precision and recall percentages are indicated as in Figure S3.

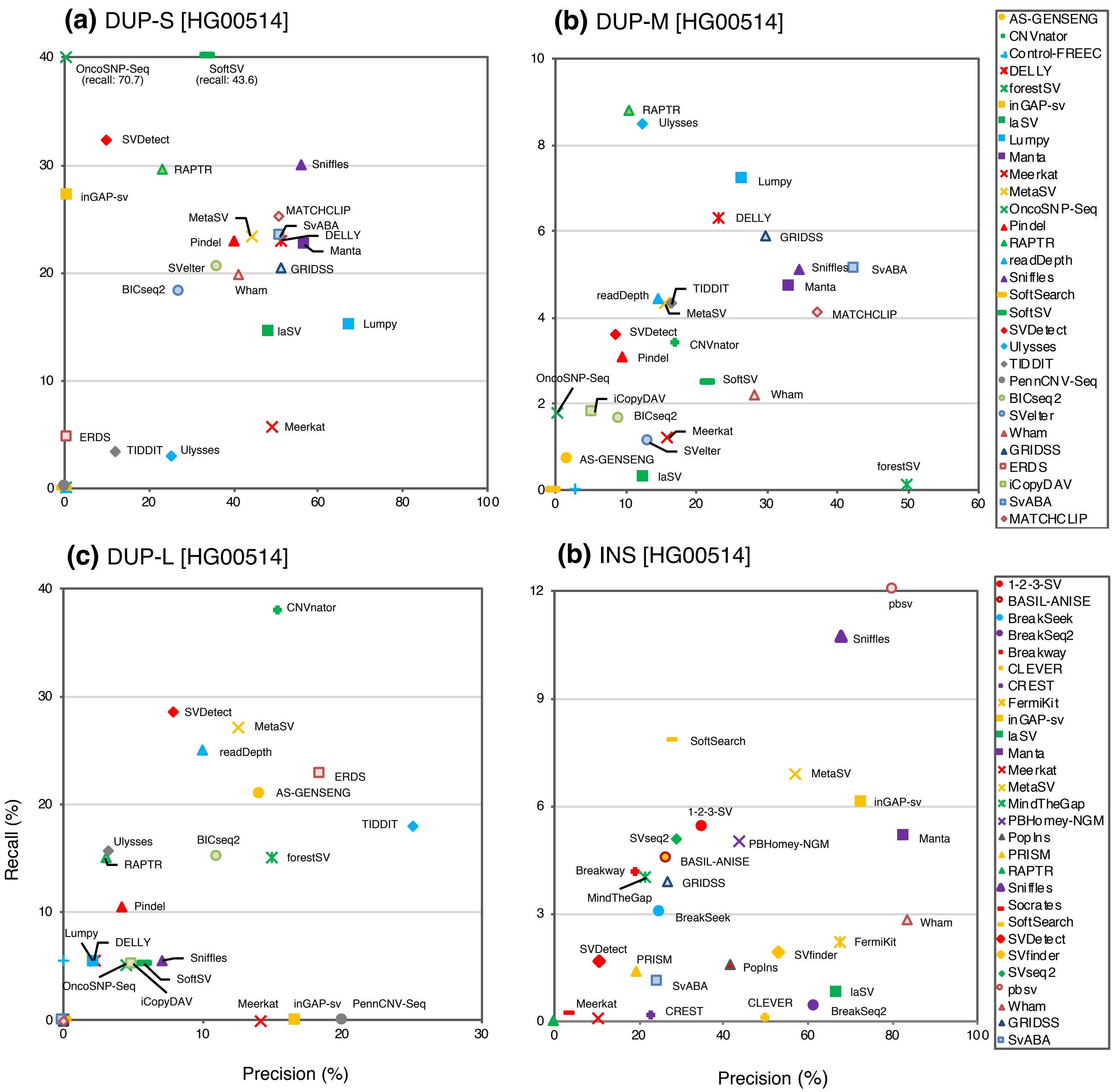

Figure S11. Precision and recall of DUPs and INSs called using existing algorithms with the HG00514 real data. DUPs (**a–c**) and INSs (**d**) were called using the indicated algorithms and the HG00514 real data. DUPs were categorized into three sets depending on the size-ranges; S: 50 bp–1 Kb (**a**), M: 1 Kb–100 Kb (**b**), L: 100 Kb–1 Mb (**c**). The precision and recall percentages are indicated as in Figure S3.

(a) DEL

	Sim-A	NA128 (data-1)	NA128 (data-2)	NA128 (data-3)	NA128 (data-4)
NA128 (data-1)	0.749				
NA128 (data-2)	0.773	0.971			
NA128 (data-3)	0.722	0.897	0.869		
NA128 (data-4)	0.733	0.837	0.832	0.928	
HG00514	0.724	0.813	0.819	0.904	0.758

(b) DUP

	Sim-A	NA128 (data-1)	NA128 (data-2)	NA128 (data-3)	NA128 (data-4)
NA128 (data-1)	0.494				
NA128 (data-2)	0.510	0.986			
NA128 (data-3)	0.482	0.891	0.888		
NA128 (data-4)	0.481	0.809	0.802	0.947	
HG00514	0.717	0.771	0.755	0.810	0.760

Color scale
1.0
0.9
0.8
0.7
0.6
0.5
0.4
0.3

(c) INS

	Sim-A	NA128 (data-1)	NA128 (data-2)	NA128 (data-3)	NA128 (data-4)
NA128 (data-1)	0.667				
NA128 (data-2)	0.679	0.920			
NA128 (data-3)	0.516	0.934	0.939		
NA128 (data-4)	0.260	0.730	0.770	0.882	
HG00514	0.567	0.849	0.883	0.889	0.722

(d) INV

	Sim-A	NA128 (data-1)	NA128 (data-2)	NA128 (data-3)
NA128 (data-1)	0.543			
NA128 (data-2)	0.535	0.932		
NA128 (data-3)	0.686	0.814	0.850	
NA128 (data-4)	0.764	0.711	0.675	0.855

Figure S12. Spearman's rank correlation of SV calling accuracy between the datasets. Spearman's rank correlation coefficients were determined with the F-measures calculated with precision and recall values from pairs of datasets, which included the evaluation results for 23~45 algorithms for each SV type. The Spearman's rank correlation coefficients for DEL (**a**), DUP (**b**), INS (**c**), and INV (**d**) were determined with the pairs of the Sim-A data, NA12878 real data-1, NA12878 real data-2, NA12878 real data-3, NA12878 real data-4, and HG00514 real datasets, which are indicated at the left side and the upper panel. The levels of the Spearman's rank correlation coefficients are shown as a heatmap.

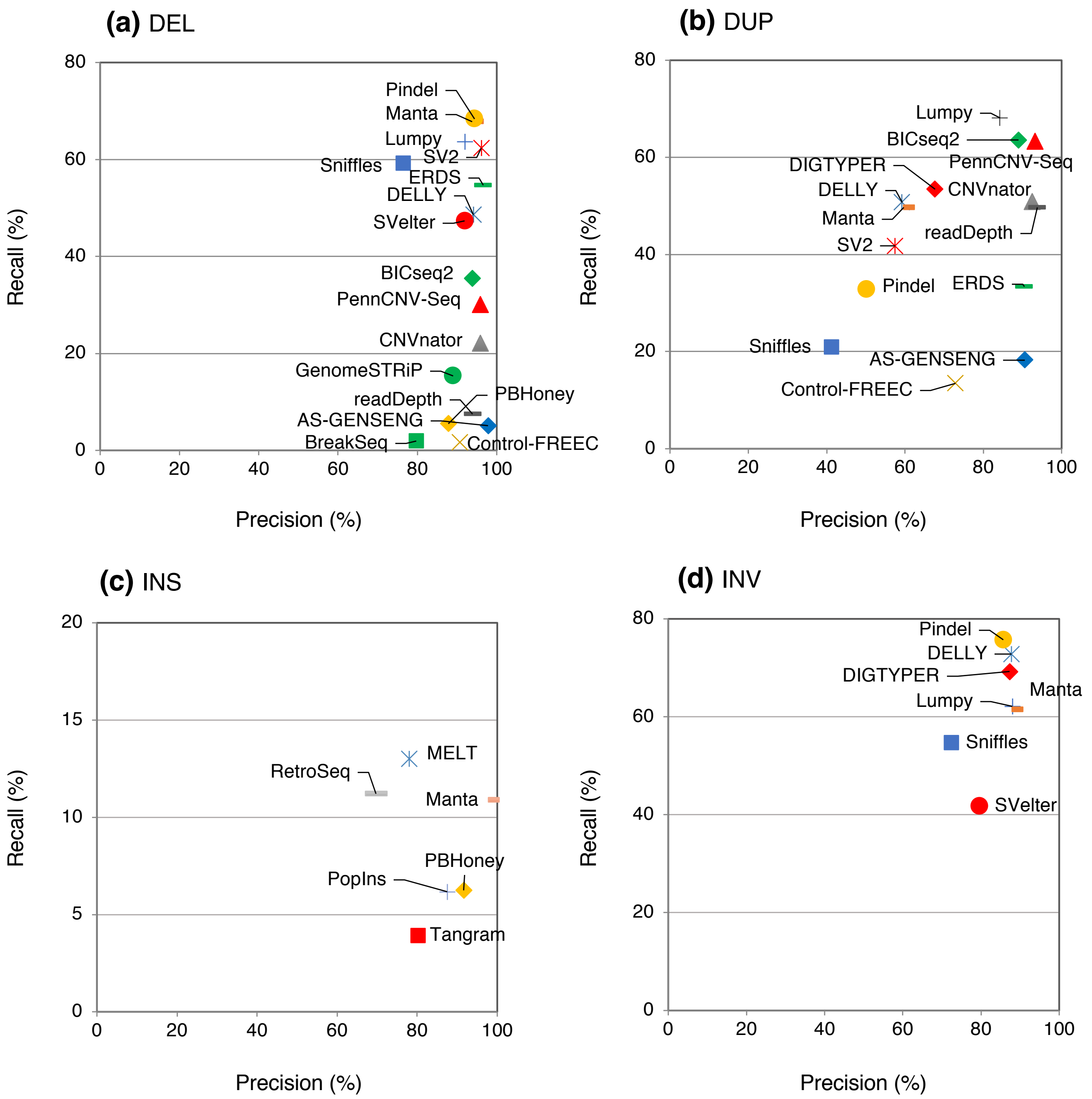

Figure S14. Precision and recall of genotypes called with the simulated data. The genotypes of DEL (**a**), DUP (**b**), INS (**c**), and INV (**d**) were called using the indicated algorithms with the simulated data. Precision was calculated by dividing the number of correctly called genotypes with the number of true positive calls with genotyped information and recall was calculated by dividing the number of correctly called genotypes with the total number of the corresponding reference SVs. The precision and recall determined for each respective SV type are indicated with the scales on the x-axis and y-axis, respectively.

Algorithm	BreakSeek:3	CREST:3	GRIDSS:6	Lumpy:7	Manta:3	PBHoney-NGM:3	pbsv:3	Pindel:9	PRISM:5	Sniffles:2	Socrates:4	SoftSV:5	Sprites:3	SVseq2:5
BreakSeek:3	41.2/81.8<br>25.9/53.5	7.1/100<br>4.2/73.6	34.1/100<br>18.3/70.5	23.0/100<br>9.6/81.1	35.7/88.4<br>23.3/67.5	31.7/93.1<br>10.8/71.4	33.3/91.6<br>11.3/53.1	11.1/93.3<br>4.9/77.6	35.7/97.8<br>17.2/67.0	30.9/93.0<br>14.1/71.8	23.8/100<br>8.7/82.8	33.3/91.4<br>11.6/76.2	36.5/97.8<br>23.2/68.8	34.1/97.7<br>15.4/69.8
CREST:3	7.1/100<br>4.2/73.6	7.9/30.3<br>6.7/26.7	7.9/100<br>5.8/73.9	5.5/100<br>3.8/79.6	7.1/100<br>6.4/72.8	7.1/100<br>3.1/81.1	7.9/100<br>3.1/58.3	3.1/100<br>1.8/73.1	6.3/100<br>4.1/72.3	6.3/100<br>3.8/81.0	5.5/100<br>3.3/84.2	7.9/100<br>4.0/78.6	7.9/100<br>6.4/73.4	7.1/100<br>5.1/75.6
GRIDSS:6	34.1/100<br>18.3/70.5	7.9/100<br>5.8/73.9	54.7/92.2<br>31.5/55.0	30.1/100<br>12.8/80.1	44.4/100<br>29.6/64.5	44.4/100<br>11.4/70.8	45.2/100<br>12.2/52.9	19.8/100<br>6.1/75.5	48.4/100<br>18.0/63.7	42.0/100<br>15.2/70.5	30.9/100<br>11.1/84.6	38.8/100<br>13.6/78.2	49.2/100<br>29.4/63.5	46.0/100<br>20.1/66.7
Lumpy:7	23.0/100<br>9.6/81.1	5.5/100<br>3.8/79.6	30.1/100<br>12.8/80.1	30.1/100<br>13.1/76.9	28.5/100<br>12.6/81.2	26.9/100<br>6.1/85.9	28.5/100<br>6.3/63.8	15.0/100<br>3.9/82.6	26.9/100<br>7.6/83.5	26.1/100<br>8.0/85.5	25.3/100<br>8.6/86.7	26.9/100<br>9.5/82.7	27.7/100<br>12.5/82.6	27.7/100<br>10.6/82.5
Manta:3	35.7/88.4<br>23.3/67.5	7.1/100<br>6.4/72.8	44.4/100<br>29.6/64.5	28.5/100<br>12.6/81.2	55.5/69.4<br>46.0/42.5	46.8/91.0<br>18.5/64.9	48.4/90.1<br>19.1/48.4	18.2/92.0<br>7.7/76.6	46.8/92.3<br>28.7/58.1	45.2/90.7<br>23.8/65.4	28.5/97.2<br>10.9/84.1	37.3/94.1<br>14.5/76.0	46.8/93.8<br>40.1/61.1	41.2/98.1<br>23.3/66.3
PBHoney-NGM:3	31.7/93.1<br>10.8/71.4	7.1/100<br>3.1/81.1	44.4/100<br>11.4/70.8	26.9/100<br>6.1/85.9	46.8/91.0<br>18.5/64.9	72.2/65.4<br>29.7/47.4	70.6/69.3<br>28.8/53.6	20.6/100<br>3.8/70.3	53.9/100<br>15.5/64.4	63.4/71.5<br>26.2/55.1	27.7/100<br>5.6/88.8	37.3/96.1<br>6.7/78.2	55.5/95.9<br>18.9/65.5	38.8/100<br>9.3/72.4
pbsv:3	33.3/91.6<br>11.3/53.1	7.9/100<br>3.1/58.3	45.2/100<br>12.2/52.9	28.5/100<br>6.3/63.8	48.4/90.1<br>19.1/48.4	70.6/69.3<br>28.8/53.6	80.9/53.4<br>43.6/48.4	21.4/93.1<br>3.6/56.7	57.1/100<br>16.4/48.1	67.4/64.8<br>36.1/52.3	30.1/100<br>5.9/65.0	39.6/91.3<br>7.1/59.0	56.3/88.0<br>19.9/48.7	42.8/93.3<br>13.6/71.2
Pindel:9	11.1/93.3<br>4.9/77.6	3.1/100<br>1.8/73.1	19.8/100<br>6.1/75.5	15.0/100<br>3.9/82.6	18.2/92.0<br>7.7/76.6	20.6/100<br>3.8/70.3	21.4/93.1<br>3.6/56.7	21.4/58.6<br>7.9/34.7	19.0/88.8<br>4.8/68.6	19.0/100<br>4.7/71.9	12.6/100<br>2.9/96.6	15.8/86.9<br>4.0/83.8	16.6/100<br>7.1/72.6	15.8/100<br>4.7/76.5
PRISM:5	35.7/97.8<br>17.2/67.0	6.3/100<br>4.1/72.3	48.4/100<br>18.0/63.7	26.9/100<br>7.6/83.5	46.8/92.3<br>28.7/58.1	53.9/100<br>15.5/64.4	57.1/100<br>16.4/48.1	19.0/88.8<br>4.8/68.6	69.0/47.5<br>35.0/39.1	50.0/100<br>19.8/61.9	27.7/100<br>7.3/83.8	38.0/100<br>10.1/76.6	57.9/94.8<br>29.7/58.7	46.0/98.3<br>20.2/60.3
Sniffles:2	30.9/93.0<br>14.1/71.8	6.3/100<br>3.8/81.0	42.0/100<br>15.2/70.5	26.1/100<br>8.0/85.5	45.2/90.7<br>23.8/65.4	63.4/71.5<br>26.2/55.1	67.4/64.8<br>36.1/52.3	19.0/100<br>4.7/71.9	50.0/100<br>19.8/61.9	69.8/63.5<br>37.9/48.5	26.9/100<br>7.3/87.0	34.1/93.8<br>9.0/78.4	50.7/91.7<br>24.7/66.0	37.3/94.1<br>12.5/70.6
Socrates:4	23.8/100<br>8.7/82.8	5.5/100<br>3.3/84.2	30.9/100<br>11.1/84.6	25.3/100<br>8.6/86.7	28.5/97.2<br>10.9/84.1	27.7/100<br>5.6/88.8	30.1/100<br>5.9/65.0	12.6/100<br>2.9/96.6	27.7/100<br>7.3/83.8	26.9/100<br>7.3/87.0	32.5/48.2<br>11.6/39.5	27.7/100<br>8.0/84.7	30.9/97.5<br>11.2/85.4	30.9/100<br>10.0/82.1
SoftSV:5	33.3/91.4<br>11.6/76.2	7.9/100<br>4.0/78.6	38.8/100<br>13.6/78.2	26.9/100<br>9.5/82.7	37.3/94.1<br>14.5/76.0	37.3/96.1<br>6.7/78.2	39.6/91.3<br>7.1/59.0	15.8/86.9<br>4.0/83.8	38.0/100<br>10.1/76.6	34.1/93.8<br>9.0/78.4	27.7/100<br>8.0/84.7	45.2/83.3<br>15.3/66.1	40.4/100<br>14.5/77.0	36.5/97.9<br>13.2/76.8
Sprites:3	36.5/97.8<br>23.2/68.8	7.9/100<br>6.4/73.4	49.2/100<br>29.4/63.5	27.7/100<br>12.5/82.6	46.8/93.8<br>40.1/61.1	55.5/95.9<br>18.9/65.5	56.3/88.0<br>19.9/48.7	16.6/100<br>7.1/72.6	57.9/94.8<br>29.7/58.7	50.7/91.7<br>24.7/66.0	30.9/97.5<br>11.2/85.4	40.4/100<br>14.5/77.0	67.4/53.3<br>48.4/30.2	47.6/92.4<br>24.2/62.8
SVseq2:5	34.1/97.7<br>15.4/69.8	7.1/100<br>5.1/75.6	46.0/100<br>20.1/66.7	27.7/100<br>10.6/82.5	41.2/98.1<br>23.3/66.3	38.8/100<br>9.3/72.4	42.8/93.3<br>13.6/71.2	15.8/100<br>4.7/76.5	46.0/98.3<br>20.2/60.3	37.3/94.1<br>12.5/70.6	30.9/100<br>10.0/82.1	36.5/97.9<br>13.2/76.8	47.6/92.4<br>24.2/62.8	52.3/86.0<br>26.0/51.7

Figure S15. Recall and precision of SVs commonly called between a pair of SV detection algorithms for the DEL-SS category. DELs in the small size-range (50 bp–100 bp), called using the indicated algorithms, were filtered with the minimum number of reads supporting the called SVs, indicated with the suffix number of the algorithm name. The DELs overlapping between the filtered SV sets from a pair of the indicated algorithms were selected, and the recall and precision of the selected DELs were determined. Recall (%) and precision (%) are presented with an intervening slash, and the recall/precision values for the simulated and real data are indicated in the upper and lower lines of each cell, respectively. The values for the real data are the mean of the values determined with four different NA12878 datasets. The recall/precision values for each individual algorithm are indicated with blue letters and white background. The data contained in the top 20th percentile of the combined precision scores for the simulated and real data are highlighted with a red background, and the next data contained in the top 21st–50th percentile of the combined precision scores are shown with a pale red background.

Algorithm	BreakDancer:4	BreakSeek:3	CLEVER:8	CREST:4	DELLY:4	ERDS:3	FermiKit:5	forestSV:5	GRIDSS:6	inGAP-sv:7	Lumpy:4	Manta:3	MATCHCLIP:4	Meerkat:7	MetaSV:3	PBHoney-NGM:2	pbsv:3	Pindel:4	PRISM:4	Sniffles:2	Socrates:3	SoftSV:4	Sprites:3	SvABA:3	SVelter:3	SVseq2:6	Ulysses:4	Wham:5
BreakDancer:4	60.3/84.2<br>13.8/80.2	49.7/99.7<br>9.2/97.8	59.8/99.7<br>10.7/92.4	21.8/100<br>3.5/99.1	28.0/100<br>7.8/87.7	41.9/99.3<br>7.2/97.6	49.5/99.5<br>9.7/98.0	26.2/91.6<br>4.9/92.1	62.3/99.7<br>13.3/95.0	60.8/99.1<br>13.3/93.3	58.6/99.7<br>10.7/95.4	57.3/99.8<br>11.5/96.7	54.6/99.8<br>11.0/96.1	47.9/99.7<br>9.0/99.0	54.7/99.7<br>10.9/97.8	56.1/99.5<br>8.2/95.4	56.5/99.7<br>6.1/72.1	58.2/99.8<br>11.3/94.7	43.8/99.7<br>9.2/86.3	58.3/99.7<br>7.9/96.2	48.3/99.8<br>2.8/99.0	55.7/99.8<br>10.4/98.9	60.9/99.7<br>12.1/95.8	37.7/99.8<br>7.2/97.0	37.4/99.5<br>8.8/97.7	57.1/99.7<br>12.8/98.7	34.2/99.1<br>10.3/70.6	53.8/99.7<br>10.7/98.6
BreakSeek:3	49.7/99.7<br>9.2/97.8	69.1/98.5<br>23.1/85.8	60.7/99.4<br>15.7/97.0	23.1/100<br>5.6/98.4	24.7/100<br>6.1/96.4	37.1/99.8<br>5.6/98.9	55.7/99.9<br>17.8/97.8	17.6/99.7<br>2.5/98.2	67.6/99.9<br>21.6/97.2	55.3/100<br>12.6/97.2	61.4/99.9<br>18.6/98.0	62.5/99.4<br>20.7/97.0	56.2/99.9<br>16.1/98.7	47.5/99.5<br>9.2/99.2	47.8/99.8<br>9.1/99.4	61.7/99.5<br>15.0/98.1	62.1/99.5<br>11.3/73.6	64.4/99.7<br>20.8/97.5	51.4/99.7<br>13.2/95.2	63.5/99.6<br>14.5/98.2	54.7/100<br>6.4/98.6	64.0/99.6<br>16.9/97.6	67.9/99.4<br>21.7/95.5	37.7/100<br>10.7/98.1	34.0/100<br>9.2/99.1	65.4/99.5<br>19.1/98.1	29.9/99.8<br>8.1/72.2	55.4/99.7<br>14.0/99.3
CLEVER:8	59.8/99.7<br>10.7/92.4	60.7/99.4<br>15.7/97.0	81.4/87.3<br>27.9/40.3	26.3/100<br>5.2/98.8	28.2/99.8<br>6.7/94.0	41.1/99.8<br>6.5/98.2	60.0/99.7<br>16.8/98.0	21.9/99.7<br>3.2/93.2	77.4/99.8<br>22.0/96.4	67.6/99.8<br>16.1/93.3	68.0/99.8<br>16.8/98.8	69.4/99.7<br>19.3/96.7	63.0/99.8<br>15.8/98.7	54.2/99.4<br>9.7/99.1	56.3/99.9<br>9.8/98.0	72.1/99.7<br>17.8/93.0	73.1/99.6<br>13.6/70.0	70.9/99.6<br>19.4/96.1	56.7/99.3<br>13.8/91.0	74.4/99.7<br>17.2/93.6	57.7/100<br>4.7/99.1	67.3/99.6<br>15.6/98.5	75.8/99.6<br>20.3/96.0	43.6/99.8<br>10.5/97.8	37.6/99.8<br>11.0/97.2	69.4/99.7<br>18.1/98.3	33.9/99.0<br>9.1/71.6	61.1/99.9<br>13.7/99.5
CREST:4	21.8/100<br>3.5/99.1	23.1/100<br>5.6/98.4	26.3/100<br>5.2/98.8	29.8/46.3<br>8.2/38.1	11.0/99.5<br>2.2/93.2	14.9/100<br>1.9/99.1	25.1/100<br>6.7/98.6	7.8/100<br>0.9/99.6	29.5/100<br>8.0/97.9	24.2/100<br>4.5/99.1	27.8/100<br>7.4/98.3	27.6/100<br>7.7/97.9	23.3/100<br>5.1/99.3	21.2/100<br>3.5/98.9	20.6/100<br>3.3/99.8	26.4/99.8<br>4.9/99.1	26.6/100<br>3.7/74.3	28.5/100<br>7.8/97.9	21.9/100<br>4.3/96.2	26.8/100<br>4.7/99.0	25.6/100<br>2.8/97.6	27.7/99.6<br>6.4/98.4	29.5/100<br>8.1/97.5	17.3/100<br>3.9/97.6	14.9/100<br>2.4/99.1	28.9/100<br>6.6/98.4	13.5/100<br>3.0/74.3	25.1/99.7<br>5.4/99.5
DELLY:4	28.0/100<br>7.8/87.7	24.7/100<br>6.1/96.4	28.2/99.8<br>6.7/94.0	11.0/99.5<br>2.2/93.2	30.4/99.4<br>8.9/88.8	23.1/100<br>5.2/96.7	23.0/99.7<br>6.0/97.3	9.3/100<br>2.4/66.9	29.6/99.8<br>8.4/95.1	28.4/100<br>8.2/95.4	29.7/99.8<br>6.7/95.9	27.3/100<br>7.2/96.5	26.7/99.8<br>7.2/97.6	24.0/99.7<br>5.6/96.8	25.6/100<br>6.5/98.5	26.3/99.8<br>5.2/95.4	26.4/99.8<br>3.9/72.4	27.2/99.8<br>7.0/95.9	18.6/99.7<br>5.8/97.0	27.9/100<br>5.3/96.4	22.9/100<br>1.9/97.8	26.8/99.6<br>6.5/97.4	29.1/99.8<br>7.6/94.9	18.8/99.7<br>4.6/95.0	21.3/100<br>5.8/94.6	26.4/99.8<br>7.8/98.6	28.9/99.6<br>8.3/69.2	25.8/100<br>6.5/97.7
ERDS:3	41.9/99.3<br>7.2/97.6	37.1/99.8<br>5.6/98.9	41.1/99.8<br>6.5/98.2	14.9/100<br>1.9/99.1	23.1/100<br>5.2/96.7	43.6/95.1<br>8.8/82.8	34.1/100<br>5.6/99.4	16.7/99.0<br>2.2/94.0	44.5/99.8<br>8.1/97.8	43.0/98.9<br>8.1/96.1	42.3/99.8<br>6.2/98.7	43.1/100<br>6.9/99.2	39.4/100<br>6.4/99.3	35.7/99.8<br>5.1/98.7	39.6/99.8<br>6.0/99.9	40.4/99.7<br>5.4/96.0	40.6/99.8<br>4.0/72.4	42.0/100<br>6.6/99.2	31.3/100<br>5.4/99.0	41.7/99.8<br>5.3/96.8	34.1/100<br>2.0/99.7	40.0/100<br>6.1/99.1	43.0/99.7<br>7.2/98.7	26.1/100<br>4.5/99.4	30.9/98.6<br>5.9/98.6	40.0/100<br>7.4/99.0	27.8/99.8<br>6.6/70.2	37.3/100<br>6.0/99.6
FermiKit:5	49.5/99.5<br>9.7/98.0	55.7/99.9<br>17.8/97.8	60.0/99.7<br>16.8/98.0	25.1/100<br>6.7/98.6	23.0/99.7<br>6.0/97.3	34.1/100<br>5.6/99.4	67.8/99.2<br>27.2/89.6	17.6/100<br>3.0/98.9	66.5/99.8<br>24.4/96.4	55.2/99.9<br>13.4/98.6	61.1/99.8<br>21.1/97.4	61.1/99.9<br>23.7/95.7	54.5/99.8<br>17.2/98.8	46.2/99.7<br>9.5/99.6	48.4/99.8<br>9.5/98.7	59.5/99.9<br>15.5/97.7	59.6/100<br>11.8/73.3	64.4/99.8<br>23.6/95.7	51.3/99.7<br>13.5/94.5	61.4/100<br>15.1/97.9	55.6/100<br>6.7/97.1	61.1/99.8<br>17.5/98.5	66.1/99.8<br>24.4/95.4	38.1/99.8<br>10.9/97.5	32.8/99.8<br>9.3/99.1	64.1/99.8<br>19.1/97.7	28.2/99.8<br>8.2/74.4	55.5/99.9<br>14.8/99.4
forestSV:5	26.2/91.6<br>4.9/92.1	17.6/99.7<br>2.5/98.2	21.9/99.7<br>3.2/93.2	7.8/100<br>0.9/99.6	9.3/100<br>2.4/66.9	16.7/99.0<br>2.2/94.0	17.6/100<br>3.0/98.9	21.8/54.0<br>8.0/28.0	20.4/99.7<br>4.1/95.8	20.3/99.4<br>3.8/93.2	18.3/98.8<br>2.6/98.5	19.6/100<br>3.5/97.1	18.4/100<br>2.8/98.9	15.5/100<br>2.2/98.4	18.2/99.1<br>2.3/99.4	19.6/99.4<br>2.7/93.9	18.9/99.7<br>2.0/71.9	19.2/100<br>3.2/99.1	14.8/100<br>2.5/98.9	19.5/99.7<br>2.7/95.4	15.6/100<br>1.1/98.7	18.3/100<br>2.6/99.1	20.2/99.7<br>3.5/96.4	11.3/100<br>2.4/98.6	13.4/100<br>2.6/96.6	19.1/99.7<br>3.4/99.4	14.6/82.5<br>7.9/68.9	17.3/99.1<br>2.5/98.1
GRIDSS:6	62.3/99.7<br>13.3/95.0	67.6/99.9<br>21.6/97.2	77.4/99.8<br>22.0/96.4	29.5/100<br>8.0/97.9	29.6/99.8<br>8.4/95.1	44.5/99.8<br>8.1/97.8	66.5/99.8<br>24.4/96.4	20.4/99.7<br>4.1/95.8	88.0/97.9<br>34.7/87.9	70.2/99.6<br>19.3/95.5	75.8/99.5<br>25.7/94.2	77.4/99.8<br>28.9/95.5	67.4/99.8<br>21.2/98.0	57.8/99.7<br>12.0/98.9	59.2/99.9<br>12.0/98.7	76.9/99.6<br>19.4/97.3	77.5/99.7<br>14.7/73.0	79.5/99.8<br>28.6/95.4	61.6/99.7<br>16.9/95.0	79.2/99.7<br>18.9/97.1	65.1/100<br>8.3/95.2	74.7/99.7<br>21.4/97.8	83.8/99.6<br>30.5/93.3	47.8/99.8<br>14.4/97.1	39.4/99.7<br>12.9/98.4	78.6/99.8<br>23.4/96.6	35.9/99.5<br>11.5/71.2	66.1/100<br>17.8/98.9
inGAP-sv:7	60.8/99.1<br>13.3/93.3	55.3/100<br>12.6/97.2	67.6/99.8<br>16.1/93.3	24.2/100<br>4.5/99.1	28.4/100<br>8.2/95.4	43.0/98.9<br>8.1/96.1	55.2/99.9<br>13.4/98.6	20.3/99.4<br>3.8/93.2	70.2/99.6<br>19.3/95.5	71.9/95.8<br>21.1/86.1	64.6/99.9<br>14.1/97.4	64.4/99.9<br>15.9/97.7	60.2/100<br>14.0/98.1	53.8/99.8<br>10.9/98.9	57.4/99.9<br>11.7/99.0	63.8/99.6<br>13.0/96.0	64.5/99.7<br>9.7/72.4	65.9/100<br>15.7/98.2	50.6/99.8<br>12.5/96.7	66.4/99.9<br>12.5/96.6	53.8/100<br>4.1/99.4	62.3/100<br>13.8/99.0	68.5/99.8<br>16.7/97.4	43.1/100<br>10.4/98.0	38.9/99.2<br>12.0/97.5	63.8/100<br>16.7/99.3	34.5/99.7<br>11.2/70.2	60.4/99.4<br>13.5/99.0
Lumpy:4	58.6/99.7<br>10.7/95.4	61.4/99.9<br>18.6/98.0	68.0/99.8<br>16.8/98.8	27.8/100<br>7.4/98.3	29.7/99.8<br>6.7/95.9	42.3/99.8<br>6.2/98.7	61.1/99.8<br>21.1/97.4	18.3/98.8<br>2.6/98.5	75.8/99.5<br>25.7/94.2	64.6/99.9<br>14.1/97.4	76.5/99.2<br>26.2/90.3	69.7/99.8<br>24.2/96.9	62.1/99.8<br>18.4/98.8	53.7/99.8<br>10.4/99.4	55.6/99.9<br>10.4/99.2	67.3/99.8<br>15.2/98.8	67.6/99.8<br>11.4/74.2	72.3/99.8<br>24.0/96.5	54.0/99.7<br>12.5/98.2	69.9/99.9<br>14.6/98.9	62.4/100<br>7.7/94.6	68.2/99.8<br>18.6/98.2	74.4/99.6<br>25.3/92.5	44.3/99.8<br>11.9/97.9	38.3/100<br>10.1/99.1	70.9/99.8<br>20.5/97.7	35.7/99.4<br>8.9/71.0	63.5/99.9<br>16.5/99.3
Manta:3	57.3/99.8<br>11.5/96.7	62.5/99.4<br>20.7/97.0	69.4/99.7<br>19.3/96.7	27.6/100<br>7.7/97.9	27.3/100<br>7.2/96.5	43.1/100<br>6.9/99.2	61.1/99.9<br>23.7/95.7	19.6/100<br>3.5/97.1	77.4/99.8<br>28.9/95.5	64.4/99.9<br>15.9/97.7	69.7/99.8<br>24.2/96.9	79.6/99.6<br>32.5/78.9	62.5/99.7<br>19.9/98.3	53.6/99.7<br>11.1/99.1	55.0/99.9<br>11.1/98.7	70.5/99.0<br>17.8/96.0	70.7/98.9<br>13.6/72.5	73.4/99.4<br>27.7/94.7	56.8/98.8<br>16.6/88.8	72.5/99.2<br>17.3/96.6	60.6/100<br>8.0/97.6	69.1/99.2<br>20.6/97.5	76.5/98.6<br>29.5/92.6	43.3/100<br>13.3/97.1	38.5/99.8<br>11.2/98.3	72.1/99.5<br>22.5/97.0	33.1/100<br>10.0/72.9	61.6/99.9<br>16.8/99.2
MATCHCLIP:4	54.6/99.8<br>11.0/96.1	56.2/99.9<br>16.1/98.7	63.0/99.8<br>15.8/98.7	23.3/100<br>5.1/99.3	26.7/99.8<br>7.2/97.6	39.4/100<br>6.4/99.3	54.5/99.8<br>17.2/98.8	18.4/100<br>2.8/98.9	67.4/99.8<br>21.2/98.0	60.2/100<br>14.0/98.1	62.1/99.8<br>18.4/98.8	62.5/99.7<br>19.9/98.3	67.8/99.6<br>21.4/96.5	49.0/99.7<br>10.1/99.4	51.7/99.9<br>10.6/99.3	60.5/100<br>13.3/99.2	60.9/100<br>10.0/74.4	64.1/99.8<br>19.8/98.2	50.6/99.6<br>12.7/94.9	62.4/100<br>12.9/99.2	54.9/100<br>6.0/99.2	62.1/99.7<br>16.7/98.9	66.2/99.7<br>20.5/98.0	38.4/99.8<br>10.4/98.2	36.6/100<br>10.1/98.8	63.7/99.8<br>18.5/98.5	32.2/99.6<br>9.6/74.4	57.2/100<br>14.9/99.2
Meerkat:7	47.9/99.7<br>9.0/99.0	47.5/99.5<br>9.2/99.2	54.2/99.4<br>9.7/99.1	21.2/100<br>3.5/98.9	24.0/99.7<br>5.6/96.8	35.7/99.8<br>5.1/98.7	46.2/99.7<br>9.5/99.6	15.5/100<br>2.2/98.4	57.8/99.7<br>12.0/98.9	53.8/99.8<br>10.9/98.9	53.7/99.8<br>10.4/99.4	53.6/99.7<br>11.1/99.1	49.0/99.7<br>10.1/99.4	58.2/97.3<br>12.3/91.9	46.4/99.8<br>8.6/99.9	52.1/99.8<br>7.7/99.4	52.5/99.7<br>5.7/74.5	55.4/99.8<br>11.1/99.3	42.0/99.7<br>8.3/99.1	53.9/99.9<br>7.3/99.0	47.4/100<br>3.0/98.6	53.0/99.5<br>10.4/98.9	57.0/99.4<br>11.8/98.8	35.5/99.8<br>7.0/98.4	31.5/100<br>7.3/98.8	54.7/99.5<br>12.3/99.3	28.7/99.4<br>7.8/74.1	51.5/100<br>10.3/99.4
MetaSV:3	54.7/99.7<br>10.9/97.8	47.8/99.8<br>9.1/99.4	56.3/99.9<br>9.8/98.0	20.6/100<br>3.3/99.8	25.6/100<br>6.5/98.5	39.6/99.8<br>6.0/99.9	48.4/99.8<br>9.5/98.7	18.2/99.1<br>2.3/99.4	59.2/99.9<br>12.0/98.7	57.4/99.9<br>11.7/99.0	55.6/99.9<br>10.4/99.2	55.0/99.9<br>11.1/98.7	51.7/99.9<br>10.6/99.3	46.4/99.8<br>8.6/99.9	59.4/99.7<br>12.1/97.7	53.1/99.9<br>7.6/99.5	53.3/99.8<br>5.7/74.4	58.8/99.9<br>11.9/98.6	43.6/99.8<br>9.0/95.9	55.2/99.9<br>7.3/99.4	47.7/100<br>2.9/99.3	53.7/99.9<br>10.5/99.8	58.2/99.9<br>11.6/99.3	37.3/100<br>7.0/98.3	34.6/100<br>8.0/99.7	55.4/99.9<br>12.7/99.7	31.2/99.8<br>9.0/74.6	52.6/99.9<br>10.5/99.8
PBHoney-NGM:2	56.1/99.5<br>8.2/95.4	61.7/99.5<br>15.0/98.1	72.1/99.7<br>17.8/93.0	26.4/99.8<br>4.9/99.1	26.3/99.8<br>5.2/95.4	40.4/99.7<br>5.4/96.0	59.5/99.9<br>15.5/97.7	19.6/99.4<br>2.7/93.9	76.9/99.6<br>19.4/97.3	63.8/99.6<br>13.0/96.0	67.3/99.8<br>15.2/98.8	70.5/99.0<br>17.8/96.0	60.5/100<br>13.3/99.2	52.1/99.8<br>7.7/99.4	53.1/99.9<br>7.6/99.5	83.3/92.3<br>28.8/72.3	82.8/94.2<br>27.3/81.0	71.3/99.7<br>18.0/97.0	57.5/99.8<br>13.7/95.2	81.1/96.3<br>25.6/81.2	58.6/100<br>4.8/99.2	67.8/99.6<br>14.0/98.5	76.6/99.5<br>19.1/95.8	41.4/100<br>10.1/97.8	35.9/99.8<br>9.9/97.0	70.2/99.8<br>14.0/98.8	31.4/98.2<br>6.9/94.2	58.8/100<br>11.6/99.6
pbsv:3	56.5/99.7<br>6.1/72.1	62.1/99.5<br>11.3/73.6	73.1/99.6<br>13.6/70.0	26.6/100<br>3.7/74.3	26.4/99.8<br>3.9/72.4	40.6/99.8<br>4.0/72.4	59.6/100<br>11.8/73.3	18.9/99.7<br>2.0/71.9	77.5/99.7<br>14.7/73.0	64.5/99.7<br>9.7/72.4	67.6/99.8<br>11.4/74.2	70.7/98.9<br>13.6/72.5	60.9/100<br>10.0/74.4	52.5/99.7<br>5.7/74.5	53.3/99.8<br>5.7/74.4	82.8/94.2<br>27.3/81.0	84.4/92.5<br>29.4/78.4	71.7/99.5<br>13.6/72.7	58.1/99.2<br>10.5/71.3	81.9/96.1<br>26.3/81.9	59.1/99.9<br>3.7/74.4	67.9/99.2<br>10.6/73.8	76.8/98.9<br>14.5/72.5	41.8/99.6<br>7.6/73.3	36.2/99.8<br>7.5/73.2	70.7/99.2<br>14.1/98.8	31.9/99.1<br>5.2/70.7	59.4/99.7<br>8.7/74.6
Pindel:4	58.2/99.8<br>11.3/94.7	64.4/99.7<br>20.8/97.5	70.9/99.6<br>19.4/96.1	28.5/100<br>7.8/97.9	27.2/99.8<br>7.0/95.9	42.0/100<br>6.6/99.2	64.4/99.8<br>23.6/95.7	19.2/100<br>3.2/99.1	79.5/99.8<br>28.6/95.4	65.9/100<br>15.7/98.2	72.3/99.8<br>24.0/96.5	73.4/99.4<br>27.7/94.7	64.1/99.8<br>19.8/98.2	55.4/99.8<br>11.1/99.3	58.8/99.9<br>11.9/98.6	71.3/99.7<br>18.0/97.0	71.7/99.5<br>13.6/72.7	80.6/93.3<br>31.9/59.3	59.8/98.0<br>17.0/78.9	73.7/99.7<br>17.4/96.0	63.7/100<br>8.0/95.7	71.5/99.5<br>20.8/97.4	78.7/99.4<br>29.1/89.0	45.4/99.8<br>13.2/97.2	37.4/100<br>10.7/98.9	75.2/99.5<br>22.8/94.5	33.0/99.6<br>9.8/74.1	64.4/99.7<br>17.0/97.5
PRISM:4	43.8/99.7<br>9.2/86.3	51.4/99.7<br>13.2/95.2	56.7/99.3<br>13.8/91.0	21.9/100<br>4.3/96.2	18.6/99.7<br>5.8/97.0	31.3/100<br>5.4/99.0	51.3/99.7<br>13.5/94.5	14.8/100<br>2.5/98.9	61.6/99.7<br>16.9/95.0	50.6/99.8<br>12.5/96.7	54.0/99.7<br>12.5/98.2	56.8/98.8<br>16.6/88.8	50.6/99.6<br>12.7/94.9	42.0/99.7<br>8.3/99.1	43.6/99.8<br>9.0/95.9	57.5/99.8<br>13.7/95.2	58.1/99.2<br>10.5/71.3	59.8/98.0<br>17.0/78.9	64.7/84.6<br>20.5/60.4	59.1/99.7<br>13.3/94.6	48.3/100<br>4.1/97.3	55.5/99.8<br>13.7/96.7	61.0/98.8<br>17.4/84.9	33.2/99.8<br>8.9/95.4	29.7/99.8<br>8.8/98.9	58.1/99.3<br>18.0/93.6	23.5/99.7<br>8.3/71.7	48.3/99.6<br>11.4/94.6
Sniffles:2	58.3/99.7<br>7.9/96.2	63.5/99.6<br>14.5/98.2	74.4/99.7<br>17.2/93.6	26.8/100<br>4.7/99.0	27.9/100<br>5.3/96.4	41.7/99.8<br>5.3/96.8	61.4/100<br>15.1/97.9	19.5/99.7<br>2.7/95.4	79.2/99.7<br>18.9/97.1	66.4/99.9<br>12.5/96.6	69.9/99.9<br>14.6/98.9	72.5/99.2<br>17.3/96.6	62.4/100<br>12.9/99.2	53.9/99.9<br>7.3/99.0	55.2/99.9<br>7.3/99.4	81.1/96.3<br>25.6/81.2	81.9/96.1<br>26.3/81.9	73.7/99.7<br>17.4/96.0	59.1/99.7<br>13.3/94.6	85.5/95.5<br>27.9/77.8	60.4/100<br>4.8/99.1	69.5/99.7<br>13.6/98.5	78.4/99.7<br>18.5/96.2	43.2/100<br>9.7/97.8	37.5/100<br>9.6/97.4	72.4/99.7<br>13.5/98.4	33.6/99.5<br>6.8/94.5	61.0/100<br>11.0/99.4
Socrates:3	48.3/99.8<br>2.8/99.0	54.7/100<br>6.4/98.6	57.7/100<br>4.7/99.1	25.6/100<br>2.8/97.6	22.9/100<br>1.9/97.8	34.1/100<br>2.0/99.7	55.6/100<br>6.7/97.1	15.6/100<br>1.1/98.7	65.1/100<br>8.3/95.2	53.8/100<br>4.1/99.4	62.4/100<br>7.7/94.6	60.6/100<br>8.0/97.6	54.9/100<br>6.0/99.2	47.4/100<br>3.0/98.6	47.7/100<br>2.9/99.3	58.6/100<br>4.8/99.2	59.1/99.9<br>3.7/74.4	63.7/100<br>8.0/95.7	48.3/100<br>4.1/97.3	60.4/100<br>4.8/99.1	65.3/89.3<br>8.5/77.2	60.1/100<br>6.4/98.6	64.8/99.9<br>8.3/94.0	37.9/99.4<br>3.8/96.9	33.3/99.5<br>3.0/97.2	64.3/100<br>6.7/97.2	28.1/100<br>2.2/74.7	57.0/99.9<br>5.1/98.8
SoftSV:4	55.7/99.8<br>10.4/98.9	64.0/99.6<br>16.9/97.6	67.3/99.6<br>15.6/98.5	27.7/99.6<br>6.4/98.4	26.8/99.6<br>6.5/97.4	40.0/100<br>6.1/99.1	61.1/99.8<br>17.5/98.5	18.3/100<br>2.6/99.1	74.7/99.7<br>21.4/97.8	62.3/100<br>13.8/99.0	68.2/99.8<br>18.6/98.2	69.1/99.2<br>20.6/97.5	62.1/99.7<br>16.7/98.9	53.0/99.5<br>10.4/98.9	53.7/99.9<br>10.5/99.8	67.8/99.6<br>14.0/98.5	67.9/99.2<br>10.6/73.8	71.5/99.5<br>20.8/97.4	55.5/99.8<br>13.7/96.7	69.5/99.7<br>13.6/98.5	60.1/100<br>6.4/98.6	76.4/98.3<br>22.3/90.8	75.0/99.3<br>21.5/96.4	42.5/99.8<br>10.8/98.7	36.8/100<br>9.9/99.5	71.5/99.5<br>19.7/97.9	32.6/99.8<br>9.0/73.1	61.2/100<br>14.8/99.3
Sprites:3	60.9/99.7<br>12.1/95.8	67.9/99.4<br>21.7/95.5	75.8/99.6<br>20.3/96.0	29.5/100<br>8.1/97.5	29.1/99.8<br>7.6/94.9	43.0/99.7<br>7.2/98.7	66.1/99.8<br>24.4/95.4	20.2/99.7<br>3.5/96.4	83.8/99.6<br>30.5/93.3	68.5/99.8<br>16.7/97.4	74.4/99.6<br>25.3/92.5	76.5/98.6<br>29.5/92.6	66.2/99.7<br>20.5/98.0	57.0/99.4<br>11.8/98.8	58.2/99.9<br>11.6/99.3	76.6/99.5<br>19.1/95.8	76.8/98.9<br>14.5/72.5	78.7/99.4<br>29.1/89.0	61.0/98.8<br>17.4/84.9	78.4/99.7<br>18.5/96.2	64.8/99.9<br>8.3/94.0	75.0/99.3<br>21.5/96.4	87.2/84.8<br>35.9/31.7	46.7/98.6<br>13.7/97.6	38.6/99.2<br>11.6/97.7	78.6/98.3<br>23.4/94.1	35.4/98.7<br>10.6/70.9	65.9/98.6<br>17.7/97.9
SvABA:3	37.7/99.8<br>7.2/97.0	37.7/100<br>10.7/98.1	43.6/99.8<br>10.5/97.8	17.3/100<br>3.9/97.6	18.8/99.7<br>4.6/95.0	26.1/100<br>4.5/99.4	38.1/99.8<br>10.9/97.5	11.3/100<br>2.4/98.6	47.8/99.8<br>14.4/97.1	43.1/100<br>10.4/98.0	44.3/99.8<br>11.9/97.9	43.3/100<br>13.3/97.1	38.4/99.8<br>10.4/98.2	35.5/99.8<br>7.0/98.4	37.3/100<br>7.0/98.3	41.4/100<br>10.1/97.8	41.8/99.6<br>7.6/73.3	45.4/99.8<br>13.2/97.2	33.2/99.8<br>8.9/95.4	43.2/100<br>9.7/97.8	37.9/99.4<br>3.8/96.9	42.5/99.8<br>10.8/98.7	46.7/98.6<br>13.7/97.6	47.9/99.3<br>14.9/95.8	23.4/100<br>7.4/98.6	44.6/99.8<br>13.7/98.4	22.4/99.7<br>6.4/73.7	41.5/100<br>10.1/99.3
SVelter:3	37.4/99.5<br>8.8/97.7	34.0/100<br>9.2/99.1	37.6/99.8<br>11.0/97.2	14.9/100<br>2.4/99.1	21.3/100<br>5.8/94.6	30.9/98.6<br>5.9/98.6	32.8/99.8<br>9.3/99.1	13.4/100<br>2.6/96.6	39.4/99.7<br>12.9/98.4	38.9/99.2<br>12.0/97.5	38.3/100<br>10.1/99.1	38.5/99.8<br>11.2/98.3	36.6/100<br>10.1/98.8	31.5/100<br>7.3/98.8	34.6/100<br>8.0/99.7	35.9/99.8<br>9.9/97.0	36.2/99.8<br>7.5/73.2	37.4/100<br>10.7/98.9	29.7/99.8<br>8.8/98.9	37.5/100<br>9.6/97.4	33.3/99.5<br>3.0/97.2	36.8/100<br>9.9/99.5	38.6/99.2<br>11.6/97.7	23.4/100<br>7.4/98.6	39.5/47.7<br>14.6/64.3	36.4/100<br>12.0/99.2	24.5/99.5<br>7.7/72.5	35.8/99.4<br>9.6/99.3
SVseq2:6	57.1/99.7<br>12.8/98.7	65.4/99.5<br>19.1/98.1	69.4/99.7<br>18.1/98.3	28.9/100<br>6.6/98.4	26.4/99.8<br>7.8/98.6	40.0/100<br>7.4/99.0	64.1/99.8<br>19.1/97.7	19.1/99.7<br>3.4/99.4	78.6/99.8<br>23.4/96.6	63.8/100<br>16.7/99.3	70.9/99.8<br>20.5/97.7	72.1/99.5<br>22.5/97.0	63.7/99.8<br>18.5/98.5	54.7/99.5<br>12.3/99.3	55.4/99.9<br>12.7/99.7	70.2/99.8<br>14.0/98.8	70.7/99.2<br>14.1/98.8	75.2/99.5<br>22.8/94.5	58.1/99.3<br>18.0/93.6	72.4/99.7<br>13.5/98.4	64.3/100<br>6.7/97.2	71.5/99.5<br>19.7/97.9	78.6/98.3<br>23.4/94.1	44.6/99.8<br>13.7/98.4	36.4/100<br>12.0/99.2	79.7/98.4<br>24.1/91.7	32.5/99.5<br>11.3/99.4	64.1/99.5<br>17.6/99.0
Ulysses:4	34.2/99.1<br>10.3/70.6	29.9/99.8<br>8.1/72.2	33.9/99.0<br>9.1/71.6	13.5/100<br>3.0/74.3	28.9/99.6<br>8.3/69.2	27.8/99.8<br>6.6/70.2	28.2/99.8<br>8.2/74.4	14.6/82.5<br>7.9/68.9	35.9/99.5<br>11.5/71.2	34.5/99.7<br>11.2/70.2	35.7/99.4<br>8.9/71.0	33.1/100<br>10.0/72.9	32.2/99.6<br>9.6/74.4	28.7/99.4<br>7.8/74.1	31.2/99.8<br>9.0/74.6	31.4/98.2<br>6.9/94.2	31.9/99.1<br>5.2/70.7	33.0/99.6<br>9.8/74.1	23.5/99.7<br>8.3/71.7	33.6/99.5<br>6.8/94.5	28.1/100<br>2.2/74.7	32.6/99.8<br>9.0/73.1	35.4/98.7<br>10.6/70.9	22.4/99.7<br>6.4/73.7	24.5/99.5<br>7.7/72.5	32.5/99.5<br>11.3/99.4	34.6/88.4<br>16.7/51.8	27.9/99.8<br>8.6/74.5
Wham:5	53.8/99.7<br>10.7/98.6	55.4/99.7<br>14.0/99.3	61.1/99.9<br>13.7/99.5	25.1/99.7<br>5.4/99.5	25.8/100<br>6.5/97.7	37.3/100<br>6.0/99.6	55.5/99.9<br>14.8/99.4	17.3/99.1<br>2.5/98.1	66.1/100<br>17.8/98.9	60.4/99.4<br>13.5/99.0	63.5/99.9<br>16.5/99.3	61.6/99.9<br>16.8/99.2	57.2/100<br>14.9/99.2	51.5/100<br>10.3/99.4	52.6/99.9<br>10.5/99.8	58.8/100<br>11.6/99.6	59.4/99.7<br>8.7/74.6	64.4/99.7<br>17.0/97.5	48.3/99.6<br>11.4/94.6	61.0/100<br>11.0/99.4	57.0/99.9<br>5.1/98.8	61.2/100<br>14.8/99.3	65.9/98.6<br>17.7/97.9	41.5/100<br>10.1/99.3	35.8/99.4<br>9.6/99.3	64.1/99.5<br>17.6/99.0	27.9/99.8<br>8.6/74.5	66.3/97.2<br>18.0/93.8

Figure S16. Recall and precision of SVs commonly called between a pair of SV detection algorithms for the DEL-S category. DELs in the short size-range (100 bp–1 Kb), called using the indicated algorithms, were filtered with the minimum number of reads supporting the called SVs, indicated with the suffix number of the algorithm name. The DELs overlapping between the filtered SV sets from a pair of the indicated algorithms were selected, and the recall and precision of the selected DELs were determined. Recall and precision percentages are presented as in Figure S15. The data contained in the top 20th percentile of the combined precision scores for the simulated and real data are highlighted with a red background, and the next data contained in the top 21st–50th percentile of the combined precision scores are shown with a pale red background.

Algorithm	1-2-3-SV:8	BICseq2:8	BreakDancer:4	CLEVER:4	CNVnator:4	CREST:3	DELLY:5	ERDS:3	FermiKit:5	forestSV:6	GASVPro:7	GenomeSTRiP:7	GRIDSS:6	Hydra-sv:7	inGAP-sv:7	Lumpy:6	Manta:3	MATCHCLIP:4	Meerkat:7
1-2-3-SV:8	72.9/51.6<br>28.8/72.0	25.8/98.5<br>7.9/98.9	4.9/86.8<br>8.3/81.9	11.5/77.7<br>13.0/75.4	25.7/88.8<br>3.5/97.9	2.3/100<br>2.8/74.4	5.9/92.8<br>13.0/66.4	4.9/85.5<br>9.4/86.6	3.6/94.1<br>7.5/90.5	57.8/64.2<br>15.7/94.0	17.6/97.8<br>14.2/93.6	13.3/86.7<br>13.2/79.9	6.5/94.4<br>12.3/86.2	72.2/53.8<br>25.8/89.7	5.6/97.3<br>11.5/90.1	6.2/93.1<br>11.7/76.3	5.4/98.6<br>11.8/86.9	4.2/93.2<br>9.2/84.8	5.2/86.0<br>9.1/86.7
BICseq2:8	25.8/98.5<br>7.9/98.9	69.6/64.7<br>17.6/58.1	66.7/94.8<br>11.4/100	61.9/97.9<br>13.1/99.6	37.5/91.4<br>6.0/86.1	26.6/94.5<br>3.2/100	69.8/96.0<br>13.9/99.6	65.4/94.1<br>13.5/97.8	56.2/96.1<br>9.2/100	66.7/93.3<br>14.4/93.2	13.6/95.6<br>9.0/99.9	70.0/96.4<br>12.8/100	70.0/96.2<br>13.4/99.9	70.3/99.0<br>13.0/99.8	68.7/96.6<br>12.9/100	68.7/95.9<br>13.1/99.8	69.4/96.0<br>13.3/99.9	64.2/96.0<br>10.8/100	64.8/94.8<br>9.5/99.7
BreakDancer:4	4.9/86.8<br>8.3/81.9	66.7/94.8<br>11.4/100	88.8/61.2<br>26.7/90.3	78.8/99.4<br>23.5/96.5	40.8/97.0<br>5.2/99.5	34.0/100<br>6.3/97.3	88.9/99.8<br>25.7/94.3	77.0/99.0<br>17.8/99.4	71.9/99.8<br>18.0/97.9	75.6/95.9<br>18.5/99.4	17.2/100<br>16.1/96.7	89.3/99.7<br>22.5/95.6	89.0/99.7<br>25.0/95.2	87.2/91.9<br>23.9/95.3	85.8/100<br>24.1/97.8	89.2/99.7<br>25.6/94.8	87.3/99.8<br>24.4/96.0	79.7/99.8<br>20.1/99.2	81.0/99.8<br>18.2/98.4
CLEVER:4	11.5/77.7<br>13.0/75.4	61.9/97.9<br>13.1/99.6	78.8/99.4<br>23.5/96.5	84.0/59.7<br>28.2/78.7	35.2/97.2<br>5.2/98.5	31.1/100<br>6.1/98.4	83.0/99.8<br>26.7/90.9	71.0/99.2<br>18.5/98.3	64.9/100<br>17.1/98.1	70.4/96.4<br>19.0/98.9	16.2/100<br>16.5/95.8	82.0/99.9<br>22.7/94.6	82.5/100<br>25.2/95.4	81.4/61.5<br>24.9/88.7	78.1/100<br>23.9/97.1	80.5/100<br>24.6/94.9	79.5/100<br>23.9/96.2	73.0/99.5<br>20.1/99.3	75.7/97.8<br>18.0/96.5
CNVnator:4	25.7/88.8<br>3.5/97.9	37.5/91.4<br>6.0/86.1	40.8/97.0<br>5.2/99.5	35.2/97.2<br>5.2/98.5	54.6/86.0<br>10.1/62.9	17.1/99.5<br>1.2/100	40.5/99.6<br>5.7/99.4	38.8/96.3<br>5.7/98.4	32.5/99.7<br>3.5/100	47.2/92.7<br>7.3/81.2	9.0/100<br>3.8/100	40.8/99.6<br>5.4/99.5	40.4/99.6<br>5.2/100	51.2/95.5<br>6.2/99.4	39.3/99.6<br>5.0/99.7	39.1/99.6<br>5.2/99.5	39.7/99.6<br>5.2/99.5	35.3/99.5<br>4.3/99.4	38.3/97.6<br>4.0/99.8
CREST:3	2.3/100<br>2.8/74.4	26.6/94.5<br>3.2/100	34.0/100<br>6.3/97.3	31.1/100<br>6.1/98.4	17.1/99.5<br>1.2/100	36.0/43.5<br>6.8/35.5	35.7/100<br>6.8/97.5	31.0/99.5<br>4.2/99.7	29.7/100<br>5.2/95.3	30.9/97.1<br>4.5/99.8	14.0/100<br>3.9/98.5	35.4/100<br>5.9/98.3	35.6/100<br>6.7/95.3	35.1/98.9<br>6.0/99.1	34.2/100<br>6.2/99.0	34.8/100<br>6.7/95.7	34.8/100<br>6.6/96.8	31.0/100<br>4.9/99.8	33.5/99.7<br>5.8/98.6
DELLY:5	5.9/92.8<br>13.0/66.4	69.8/96.0<br>13.9/99.6	88.9/99.8<br>25.7/94.3	83.0/99.8<br>26.7/90.9	40.5/99.6<br>5.7/99.4	35.7/100<br>6.8/97.5	94.7/99.5<br>30.6/83.3	80.0/99.1<br>20.0/98.4	73.1/99.8<br>18.7/97.3	78.9/96.7<br>20.5/98.9	18.7/99.1<br>17.6/93.7	92.8/99.5<br>24.7/91.6	93.5/99.7<br>27.8/93.1	92.5/98.7<br>26.2/92.3	88.4/99.9<br>25.8/96.1	91.3/99.6<br>27.3/92.2	90.3/99.9<br>26.4/94.1	81.4/99.8<br>21.3/98.5	85.0/99.5<br>19.4/97.1
ERDS:3	4.9/85.5<br>9.4/86.6	65.4/94.1<br>13.5/97.8	77.0/99.0<br>17.8/99.4	71.0/99.2<br>18.5/98.3	38.8/96.3<br>5.7/98.4	31.0/99.5<br>4.2/99.7	80.0/99.1<br>20.0/98.4	80.7/95.6<br>22.3/86.6	63.1/99.8<br>12.3/99.8	75.7/94.8<br>18.4/97.5	16.8/99.5<br>12.2/99.7	79.0/99.7<br>16.3/99.7	79.2/99.3<br>18.6/99.6	78.8/98.3<br>17.8/98.8	77.7/99.8<br>17.8/99.3	77.7/99.3<br>18.1/99.3	78.1/99.3<br>17.9/99.9	71.6/99.6<br>14.2/99.9	73.0/98.5<br>13.1/99.8
FermiKit:5	3.6/94.1<br>7.5/90.5	56.2/96.1<br>9.2/100	71.9/99.8<br>18.0/97.9	64.9/100<br>17.1/98.1	32.5/99.7<br>3.5/100	29.7/100<br>5.2/95.3	73.1/99.8<br>18.7/97.3	63.1/99.8<br>12.3/99.8	73.7/99.6<br>19.1/95.9	62.6/97.2<br>12.9/99.9	14.3/99.4<br>11.7/98.5	73.4/99.7<br>16.3/97.8	73.4/99.7<br>18.6/96.8	72.2/98.6<br>17.5/98.7	70.2/99.8<br>17.5/99.0	73.2/99.6<br>18.5/97.0	71.5/99.8<br>18.0/97.6	66.8/99.7<br>15.9/99.8	68.4/99.6<br>14.8/98.7
forestSV:6	57.8/64.2<br>15.7/94.0	66.7/93.3<br>14.4/93.2	75.6/95.9<br>18.5/99.4	70.4/96.4<br>19.0/98.9	47.2/92.7<br>7.3/81.2	30.9/97.1<br>4.5/99.8	78.9/96.7<br>20.5/98.9	75.7/94.8<br>18.4/97.5	62.6/97.2<br>12.9/99.9	68.5/58.1<br>24.5/52.4	15.1/95.1<br>12.0/99.8	67.5/94.0<br>15.5/99.1	64.7/95.6<br>15.9/99.1	65.2/63.8<br>18.7/97.7	63.4/96.2<br>15.1/98.6	63.2/95.6<br>15.4/98.7	63.8/95.7<br>15.8/99.3	58.3/95.9<br>12.3/99.1	59.8/95.2<br>11.4/99.3
GASVPro:7	17.6/97.8<br>14.2/93.6	13.6/95.6<br>9.0/99.9	17.2/100<br>16.1/96.7	16.2/100<br>16.5/95.8	9.0/100<br>3.8/100	14.0/100<br>3.9/98.5	18.7/99.1<br>17.6/93.7	16.8/99.5<br>12.2/99.7	14.3/99.4<br>11.7/98.5	15.1/95.1<br>12.0/99.8	18.8/95.0<br>17.7/90.1	18.6/99.1<br>15.4/93.3	18.2/100<br>14.7/95.8	18.6/97.5<br>16.2/95.9	17.6/99.5<br>14.2/97.5	17.8/99.1<br>14.6/94.0	17.7/100<br>14.3/96.0	15.8/99.5<br>11.7/98.8	16.6/99.5<br>10.6/97.5
GenomeSTRiP:7	13.3/86.7<br>13.2/79.9	70.0/96.4<br>12.8/100	89.3/99.7<br>22.5/95.6	82.0/99.9<br>22.7/94.6	40.8/99.6<br>5.4/99.5	35.4/100<br>5.9/98.3	92.8/99.5<br>24.7/91.6	79.0/99.7<br>16.3/99.7	73.4/99.7<br>16.3/97.8	67.5/94.0<br>15.5/99.1	18.6/99.1<br>15.4/93.3	93.4/99.4<br>24.9/91.0	93.0/99.5<br>23.8/94.3	90.3/94.3<br>22.9/94.6	88.7/99.9<br>22.8/96.7	91.6/99.5<br>23.9/92.9	90.2/99.8<br>23.2/95.1	81.7/99.7<br>18.8/98.6	84.0/99.8<br>17.2/97.3
GRIDSS:6	6.5/94.4<br>12.3/86.2	70.0/96.2<br>13.4/99.9	89.0/99.7<br>25.0/95.2	82.5/100<br>25.2/95.4	40.4/99.6<br>5.2/100	35.6/100<br>6.7/95.3	93.5/99.7<br>27.8/93.1	79.2/99.3<br>18.6/99.6	73.4/99.7<br>18.6/96.8	64.7/95.6<br>15.9/99.1	18.2/100<br>14.7/95.8	93.0/99.5<br>23.8/94.3	94.4/99.2<br>28.6/90.4	92.3/98.6<br>25.3/95.6	88.5/99.9<br>25.4/97.4	91.3/99.4<br>26.4/92.9	90.5/99.7<br>25.8/94.9	81.6/99.7<br>20.8/98.9	85.1/99.6<br>18.9/97.7
Hydra-sv:7	72.2/53.8<br>25.8/89.7	70.3/99.0<br>13.0/99.8	87.2/91.9<br>23.9/95.3	81.4/61.5<br>24.9/88.7	51.2/95.5<br>6.2/99.4	35.1/98.9<br>6.0/99.1	92.5/98.7<br>26.2/92.3	78.8/98.3<br>17.8/98.8	72.2/98.6<br>17.5/98.7	65.2/63.8<br>18.7/97.7	18.6/97.5<br>16.2/95.9	90.3/94.3<br>22.9/94.6	92.3/98.6<br>25.3/95.6	72.8/31.9<br>28.0/77.9	10.3/97.8<br>11.3/93.1	11.0/96.0<br>11.8/79.2	9.8/97.7<br>12.0/86.4	7.0/93.8<br>9.3/88.7	8.8/83.3<br>9.0/87.7
inGAP-sv:7	5.6/97.3<br>11.5/90.1	68.7/96.6<br>12.9/100	85.8/100<br>24.1/97.8	78.1/100<br>23.9/97.1	39.3/99.6<br>5.0/99.7	34.2/100<br>6.2/99.0	88.4/99.9<br>25.8/96.1	77.7/99.8<br>17.8/99.3	70.2/99.8<br>17.5/99.0	63.4/96.2<br>15.1/98.6	17.6/99.5<br>14.2/97.5	88.7/99.9<br>22.8/96.7	88.5/99.9<br>25.4/97.4	10.3/97.8<br>11.3/93.1	88.7/99.9<br>26.2/95.9	87.7/99.9<br>25.0/96.9	87.0/100<br>24.3/97.6	79.0/99.9<br>20.5/99.3	80.8/99.8<br>18.0/98.8
Lumpy:6	6.2/93.1<br>11.7/76.3	68.7/95.9<br>13.1/99.8	89.2/99.7<br>25.6/94.8	80.5/100<br>24.6/94.9	39.1/99.6<br>5.2/99.5	34.8/100<br>6.7/95.7	91.3/99.6<br>27.3/92.2	77.7/99.3<br>18.1/99.3	73.2/99.6<br>18.5/97.0	63.2/95.6<br>15.4/98.7	17.8/99.1<br>14.6/94.0	91.6/99.5<br>23.9/92.9	91.3/99.4<br>26.4/92.9	11.0/96.0<br>11.8/79.2	87.7/99.9<br>25.0/96.9	91.7/99.5<br>27.7/90.6	89.3/99.8<br>25.9/95.0	80.9/99.7<br>20.8/98.9	82.8/99.6<br>18.9/98.0
Manta:3	5.4/98.6<br>11.8/86.9	69.4/96.0<br>13.3/99.9	87.3/99.8<br>24.4/96.0	79.5/100<br>23.9/96.2	39.7/99.6<br>5.2/99.5	34.8/100<br>6.6/96.8	90.3/99.9<br>26.4/94.1	78.1/99.3<br>17.9/99.9	71.5/99.8<br>18.0/97.6	63.8/95.7<br>15.8/99.3	17.7/100<br>14.3/96.0	90.2/99.8<br>23.2/95.1	90.5/99.7<br>25.8/94.9	9.8/97.7<br>12.0/86.4	87.0/100<br>24.3/97.6	89.3/99.8<br>25.9/95.0	91.0/99.6<br>27.0/93.3	80.3/99.8<br>20.3/98.8	82.3/99.7<br>18.3/98.0
MATCHCLIP:4	4.2/93.2<br>9.2/84.8	64.2/96.0<br>10.8/100	79.7/99.8<br>20.1/99.2	73.0/99.5<br>20.1/99.3	35.3/99.5<br>4.3/99.4	31.0/100<br>4.9/99.8	81.4/99.8<br>21.3/98.5	71.6/99.6<br>14.2/99.9	66.8/99.7<br>15.9/99.8	58.3/95.9<br>12.3/99.1	15.8/99.5<br>11.7/98.8	81.7/99.7<br>18.8/98.6	81.6/99.7<br>20.8/98.9	7.0/93.8<br>9.3/88.7	79.0/99.9<br>20.5/99.3	80.9/99.7<br>20.8/98.9	80.3/99.8<br>20.3/98.8	82.2/99.3<br>21.5/98.5	75.1/99.2<br>15.6/99.6
Meerkat:7	5.2/86.0<br>9.1/86.7	64.8/94.8<br>9.5/99.7	81.0/99.8<br>18.2/98.4	75.7/97.8<br>18.0/96.5	38.3/97.6<br>4.0/99.8	33.5/99.7<br>5.8/98.6	85.0/99.5<br>19.4/97.1	73.0/98.5<br>13.1/99.8	68.4/99.6<br>14.8/98.7	59.8/95.2<br>11.4/99.3	16.6/99.5<br>10.6/97.5	84.0/99.8<br>17.2/97.3	85.1/99.6<br>18.9/97.7	8.8/83.3<br>9.0/87.7	80.8/99.8<br>18.0/98.8	82.8/99.6<br>18.9/98.0	82.3/99.7<br>18.3/98.0	75.1/99.2<br>15.6/99.6	87.0/94.0<br>20.1/94.3
MetaSV:3	7.6/68.2<br>9.1/95.1	57.7/94.7<br>9.4/99.6	65.7/98.0<br>14.4/98.1	58.5/98.7<br>14.3/98.9	40.1/94.7<br>6.0/98.9	25.2/98.7<br>3.7/99.7	68.1/98.8<br>15.1/97.5	59.6/97.8<br>11.0/99.7	53.5/98.4<br>10.5/99.4	48.2/90.4<br>10.2/99.3	11.6/96.8<br>9.4/98.2	67.4/98.1<br>14.7/97.9	68.0/98.5<br>14.7/98.1	10.6/74.1<br>9.0/94.8	65.4/98.6<br>14.1/99.2	67.4/98.6<br>14.7/97.8	66.4/98.6<br>14.5/98.1	60.5/98.6<br>12.1/99.6	63.2/98.7<br>11.2/99.6
pbsv:3	1.1/100<br>0.9/70.6	8.2/100<br>3.1/73.6	13.1/100<br>5.8/74.7	13.3/100<br>6.0/73.7	1.1/100<br>1.1/66.4	4.4/100<br>1.4/75.0	13.2/100<br>6.2/74.0	11.9/100<br>4.3/73.7	11.7/100<br>4.3/74.8	9.3/97.5<br>3.4/74.7	2.6/100<br>3.3/74.9	13.3/100<br>5.1/74.8	13.0/100<br>6.0/74.6	2.0/100<br>0.9/70.4	12.6/100<br>5.7/74.8	13.2/100<br>5.9/74.7	12.6/100<br>5.7/74.7	12.6/100<br>5.0/74.8	11.7/100<br>3.8/74.9
PennCNV-Seq:3	29.5/72.5<br>9.0/84.4	57.4/90.1<br>11.7/74.5	69.3/98.2<br>14.7/98.8	62.1/98.0<br>14.4/96.5	38.8/95.2<br>5.9/79.7	26.4/99.1<br>3.2/99.7	71.3/98.6<br>16.0/97.5	66.6/96.7<br>14.6/92.3	55.6/99.0<br>9.5/99.5	50.9/81.0<br>13.1/73.3	12.6/100<br>8.1/99.4	69.8/98.0<br>13.5/99.0	71.2/98.8<br>15.4/99.1	30.9/71.4<br>8.8/90.9	68.4/98.7<br>14.5/99.0	69.1/98.7<br>14.9/99.2	69.3/98.9<br>14.5/99.3	64.4/98.5<br>11.5/99.4	65.7/97.8<br>10.2/99.3
Pindel:4	5.1/89.3<br>8.1/52.1	57.0/95.3<br>7.1/74.5	64.9/99.8<br>11.4/90.6	58.0/97.7<br>12.4/69.3	38.6/99.4<br>3.9/98.9	26.7/100<br>3.4/98.2	68.3/100<br>12.8/70.9	57.7/99.0<br>8.5/74.4	52.9/99.8<br>8.9/83.9	47.4/94.9<br>7.7/86.4	12.5/99.3<br>8.3/84.8	67.4/99.7<br>12.3/77.0	68.3/99.8<br>12.5/90.2	8.1/91.3<br>7.9/59.0	64.4/100<br>11.7/73.4	66.6/99.7<br>12.0/82.3	66.3/100<br>12.0/83.9	59.3/99.3<br>10.2/84.6	63.3/98.0<br>9.9/73.1
PRISM:3	1.6/75.8<br>5.6/51.1	35.1/95.8<br>5.1/100	43.1/99.6<br>7.8/97.1	40.9/98.3<br>8.6/91.5	19.8/99.2<br>2.0/100	17.2/100<br>1.8/99.1	45.6/99.8<br>8.8/87.1	39.4/99.4<br>6.5/98.1	35.3/99.7<br>5.7/96.8	31.8/94.3<br>6.1/99.8	8.6/100<br>5.8/96.1	45.4/99.6<br>7.9/91.0	45.9/99.6<br>8.6/95.0	4.1/88.5<br>5.5/67.6	43.2/100<br>7.9/97.1	44.3/99.6<br>8.0/94.4	44.2/99.8<br>8.2/95.6	40.8/99.0<br>6.9/97.4	41.2/98.1<br>5.4/96.7
RAPTR:10	55.0/50.1<br>17.7/80.4	55.9/96.6<br>9.1/98.4	69.4/93.9<br>17.9/96.1	63.1/92.6<br>17.9/93.6	38.6/91.4<br>4.1/99.0	26.1/97.1<br>3.7/99.2	73.0/96.6<br>19.0/91.2	65.4/96.1<br>13.7/96.6	57.5/96.5<br>13.1/98.6	52.1/61.5<br>13.7/94.9	13.4/95.6<br>11.6/95.4	71.2/91.9<br>15.9/94.3	73.1/96.6<br>18.2/95.2	55.4/46.3<br>17.3/88.8	70.1/97.3<br>17.7/96.8	71.4/96.5<br>17.7/94.8	70.7/96.9<br>17.4/96.2	65.7/96.7<br>15.3/98.5	67.4/95.3<br>12.9/97.5
Sniffles:2	7.7/87.0<br>5.7/86.2	64.4/96.5<br>7.5/99.3	80.7/99.4<br>12.5/99.3	74.2/98.0<br>13.3/97.5	37.3/99.3<br>3.3/98.1	32.8/100<br>3.0/99.2	83.8/99.6<br>13.6/95.8	72.4/99.5<br>9.5/98.8	66.9/99.8<br>9.2/99.6	58.8/95.3<br>8.0/98.2	16.4/99.5<br>7.8/99.5	83.3/99.7<br>11.9/98.2	83.8/99.7<br>13.3/98.6	11.2/85.4<br>5.6/94.3	79.4/99.8<br>12.3/99.2	82.3/99.7<br>12.9/98.4	81.3/99.7<br>12.3/99.2	74.6/99.7<br>11.0/99.7	77.4/99.1<br>9.0/99.5
Socrates:3	4.3/91.8<br>4.4/94.5	57.1/96.6<br>6.8/98.9	72.4/61.4<br>10.6/96.1	64.4/65.6<br>10.0/97.0	33.2/99.5<br>2.9/100	29.2/100<br>3.9/94.4	73.7/99.7<br>11.2/96.4	64.4/99.7<br>8.9/100	63.8/99.8<br>9.3/96.7	52.9/96.9<br>7.4/99.1	14.5/99.4<br>5.6/98.3	73.7/99.7<br>9.7/97.4	73.9/99.6<br>11.0/95.5	6.3/9.0<br>4.8/70.7	70.8/100<br>10.1/99.1	73.4/99.6<br>11.2/95.5	72.4/99.7<br>10.9/96.7	67.1/99.8<br>9.0/99.9	70.6/98.8<br>9.8/98.8
SoftSV:4	4.5/92.1<br>8.5/70.6	66.2/96.2<br>9.3/99.9	83.4/99.9<br>18.0/98.0	77.6/98.7<br>18.0/97.4	38.5/99.6<br>3.9/99.8	34.0/99.7<br>5.9/97.3	87.3/99.9<br>19.6/96.5	75.0/99.6<br>13.5/99.9	71.4/100<br>15.6/97.9	60.7/95.9<br>11.3/99.4	16.9/100<br>10.4/98.5	86.3/99.9<br>16.9/97.5	87.3/99.9<br>19.5/96.8	8.5/90.9<br>8.3/90.4	82.5/100<br>18.1/98.8	85.3/99.9<br>18.9/97.5	84.7/99.9<br>18.7/97.1	77.5/99.5<br>15.7/99.5	82.7/98.2<br>16.1/97.9
Sprites:3	6.8/84.7<br>10.8/61.8	69.9/96.1<br>11.0/99.6	88.1/99.7<br>21.7/96.7	82.7/98.2<br>22.1/93.5	40.8/99.2<br>4.5/99.4	35.8/99.7<br>6.7/97.1	93.3/99.7<br>24.1/91.6	79.1/99.6<br>16.0/99.1	73.1/99.8<br>17.4/97.8	64.9/95.3<br>13.7/99.1	18.0/99.5<br>12.4/97.4	91.9/99.6<br>20.4/95.4	93.1/99.7<br>23.8/95.2	11.3/89.6<br>10.5/75.0	87.3/100<br>21.7/98.3	90.4/99.7<br>22.9/95.4	89.7/99.9<br>22.4/96.5	81.2/99.4<br>18.4/99.2	86.3/98.0<br>18.7/97.6
SvABA:3	4.4/98.3<br>10.8/90.2	56.4/95.9<br>11.3/100	74.1/99.7<br>21.4/97.2	66.9/100<br>21.6/97.7	31.3/99.7<br>4.2/99.5	30.0/100<br>5.9/97.7	77.4/99.9<br>23.4/96.1	66.0/99.6<br>15.9/99.9	61.0/99.8<br>16.2/98.2	51.2/95.5<br>13.3/99.4	14.7/100<br>12.7/96.9	77.0/99.8<br>20.6/96.3	77.1/99.8<br>22.9/96.5	8.3/99.0<br>10.8/84.1	73.4/100<br>21.8/98.3	76.0/99.7<br>22.6/96.7	75.0/100<br>22.3/96.8	67.9/99.8<br>18.5/98.9	71.4/99.8<br>16.6/98.5
SVDetect:6	70.2/53.6<br>18.3/69.1	69.1/98.5<br>10.5/95.1	84.6/95.3<br>16.6/94.2	79.3/89.1<br>18.2/83.6	50.3/95.6<br>4.9/96.4	34.4/98.6<br>4.4/96.4	90.6/98.4<br>19.2/86.1	77.5/98.0<br>14.1/93.0	70.7/98.6<br>11.3/96.9	63.4/63.5<br>14.5/91.2	17.9/97.0<br>11.9/94.8	87.0/93.2<br>16.8/92.7	90.6/98.8<br>18.2/93.7	70.2/53.1<br>17.3/88.9	85.3/98.8<br>16.7/96.1	88.0/98.6<br>17.3/93.1	87.2/98.8<br>16.9/94.7	79.0/98.4<br>13.3/98.3	83.4/96.3<br>13.0/95.8
SVelter:3	4.6/95.2<br>8.2/93.6	59.6/96.2<br>8.9/99.0	72.0/99.7<br>15.9/99.1	64.8/99.4<br>15.6/98.9	34.1/99.7<br>3.4/99.7	29.7/100<br>3.6/100	72.9/99.6<br>16.9/98.8	68.3/99.5<br>12.6/98.5	59.9/99.8<br>11.3/100	55.6/96.6<br>10.8/98.6	13.4/99.4<br>9.8/99.5	73.0/99.8<br>15.0/99.4	72.8/100<br>16.7/99.5	7.6/80.4<br>8.0/91.3	72.1/99.7<br>16.2/99.3	72.8/99.7<br>16.6/98.9	72.4/100<br>16.0/99.5	67.2/99.8<br>13.6/99.8	67.2/100<br>12.4/99.8
SVfinder:10	72.0/53.0<br>26.1/84.2	70.1/98.7<br>12.8/99.1	86.3/93.6<br>23.8/94.9	80.4/59.8<br>24.8/86.9	50.7/95.1<br>6.5/99.3	34.9/98.9<br>5.9/98.7	92.0/98.3<br>26.2/90.4	78.4/97.7<br>17.6/98.5	71.4/98.2<br>17.4/97.9	65.3/64.0<br>18.6/96.1	18.2/97.1<br>16.1/95.8	89.3/93.6<br>22.7/93.8	91.6/98.2<br>25.0/94.6	72.2/32.7<br>26.0/82.0	86.8/98.7<br>23.8/97.1	89.2/98.1<br>24.3/94.9	88.6/98.6<br>24.0/95.6	80.3/98.0<br>20.2/98.4	84.6/96.4<br>17.8/96.9
SVseq2:5	5.9/92.8<br>11.0/73.6	67.5/96.0<br>8.7/100	86.1/99.9<br>16.3/98.9	79.3/98.3<br>16.9/96.8	39.3/99.6<br>3.9/100	35.4/100<br>5.0/98.3	89.8/99.9<br>17.6/95.2	76.4/99.5<br>12.1/100	72.2/100<br>13.1/98.5	62.9/95.5<br>10.9/99.5	17.7/100<br>11.5/98.9	89.2/99.9<br>16.3/97.6	90.1/99.8<br>17.5/97.9	10.6/92.6<br>10.9/95.9	84.9/100<br>16.4/98.9	88.0/99.8<br>17.0/98.1	87.0/99.9<br>16.9/98.3	79.4/99.5<br>14.3/99.6	84.3/98.0<br>14.4/97.8
TIDDIT:3	55.8/98.7<br>14.9/93.3	68.4/99.2<br>14.1/98.6	72.7/100<br>18.9/98.3	66.6/99.6<br>19.6/97.4	44.5/99.4<br>6.4/98.8	29.7/100<br>4.7/99.5	76.6/100<br>21.6/97.1	71.1/99.7<br>18.0/97.0	59.7/100<br>13.0/99.7	67.6/96.7<br>18.4/97.3	14.7/99.4<br>12.8/99.4	75.7/100<br>18.3/98.8	76.4/100<br>20.3/99.1	54.2/98.6<br>14.0/96.3	74.6/100<br>19.1/98.4	74.5/100<br>19.8/98.4	75.4/100<br>19.7/98.8	68.8/100<br>15.4/99.4	71.5/100<br>13.8/99.8
Ulysses:4	50.9/94.5<br>18.3/78.8	71.3/96.1<br>14.9/98.3	89.0/99.6<br>26.1/93.6	83.5/97.1<br>27.4/87.7	44.2/99.3<br>6.4/98.0	36.0/99.7<br>6.8/95.9	93.8/99.5<br>30.0/86.2	80.7/99.1<br>20.7/96.9	73.2/99.7<br>18.8/96.6	74.0/94.6<br>20.2/97.5	18.8/99.1<br>17.1/93.8	92.9/99.5<br>24.6/92.2	93.9/99.5<br>28.3/91.6	51.2/94.6<br>17.3/85.6	88.3/99.9<br>26.0/96.2	91.1/99.5<br>27.4/91.4	90.5/99.8<br>26.7/93.4	81.8/99.3<br>21.3/98.6	86.6/97.2<br>19.5/96.4
VariationHunter:6	30.2/66.1<br>19.6/84.0	27.6/99.4<br>11.4/98.9	35.0/99.1<br>19.5/96.8	32.4/97.2<br>21.1/91.4	19.4/97.3<br>5.1/97.4	10.6/100<br>5.2/99.4	35.8/99.7<br>21.2/91.0	29.9/99.4<br>15.0/97.8	28.5/99.7<br>14.0/98.5	28.2/75.5<br>15.2/96.9	4.2/100<br>13.9/97.1	35.8/97.5<br>18.9/94.8	36.0/99.5<br>20.6/95.8	30.1/65.5<br>18.6/90.3	34.2/100<br>19.7/97.6	35.5/99.5<br>20.1/96.0	35.2/100<br>19.6/97.0	31.9/99.2<br>16.9/99.1	33.8/98.2<br>14.7/98.2
Wham:4	5.5/90.0<br>8.8/88.4	65.0/96.2<br>9.2/99.8	83.2/100<br>18.9/98.4	75.0/100<br>18.1/98.6	37.8/99.5<br>3.7/100	33.7/100<br>6.1/98.2	85.0/100<br>19.8/98.0	72.7/99.7<br>13.3/99.9	70.7/100<br>15.6/98.8	59.2/95.3<br>11.3/99.5	16.1/100<br>10.4/98.9	84.9/99.9<br>17.5/98.3	85.0/100<br>19.7/98.0	8.8/92.7<br>8.6/94.9	81.6/100<br>18.9/99.1	84.5/100<br>19.7/97.9	83.3/100<br>19.1/98.5	76.3/100<br>16.4/99.5	80.6/99.8<br>16.6/99.3

Algorithm	MetaSV:3	pbsv:3	PennCNV-Seq:3	Pindel:4	PRISM:3	RAPTR:10	Sniffles:2	Socrates:3	SoftSV:4	Sprites:3	SvABA:3	SVDetect:6	SVelter:3	SVfinder:10	SVseq2:5	TIDDIT:3	Ulysses:4	VariationHunter:6	Wham:4
1-2-3-SV:8	7.6/68.2<br>9.1/95.1	1.1/100<br>0.9/70.6	29.5/72.5<br>9.0/84.4	5.1/89.3<br>8.1/52.1	1.6/75.8<br>5.6/51.1	55.0/50.1<br>17.7/80.4	7.7/87.0<br>5.7/86.2	4.3/91.8<br>4.4/94.5	4.5/92.1<br>8.5/70.6	6.8/84.7<br>10.8/61.8	4.4/98.3<br>10.8/90.2	70.2/53.6<br>18.3/69.1	4.6/95.2<br>8.2/93.6	72.0/53.0<br>26.1/84.2	5.9/92.8<br>11.0/73.6	55.8/98.7<br>14.9/93.3	50.9/94.5<br>18.3/78.8	30.2/66.1<br>19.6/84.0	5.5/90.0<br>8.8/88.4
BICseq2:8	57.7/94.7<br>9.4/99.6	8.2/100<br>3.1/73.6	57.4/90.1<br>11.7/74.5	57.0/95.3<br>7.1/74.5	35.1/95.8<br>5.1/100	55.9/96.6<br>9.1/98.4	64.4/96.5<br>7.5/99.3	57.1/96.6<br>6.8/98.9	66.2/96.2<br>9.3/99.9	69.9/96.1<br>11.0/99.6	56.4/95.9<br>11.3/100	69.1/98.5<br>10.5/95.1	59.6/96.2<br>8.9/99.0	70.1/98.7<br>12.8/99.1	67.5/96.0<br>8.7/100	68.4/99.2<br>14.1/98.6	71.3/96.1<br>14.9/98.3	27.6/99.4<br>11.4/98.9	65.0/96.2<br>9.2/99.8
BreakDancer:4	65.7/98.0<br>14.4/98.1	13.1/100<br>5.8/74.7	69.3/98.2<br>14.7/98.8	64.9/99.8<br>11.4/90.6	43.1/99.6<br>7.8/97.1	69.4/93.9<br>17.9/96.1	80.7/99.4<br>12.5/99.3	72.4/61.4<br>10.6/96.1	83.4/99.9<br>18.0/98.0	88.1/99.7<br>21.7/96.7	74.1/99.7<br>21.4/97.2	84.6/95.3<br>16.6/94.2	72.0/99.7<br>15.9/99.1	86.3/93.6<br>23.8/94.9	86.1/99.9<br>16.3/98.9	72.7/100<br>18.9/98.3	89.0/99.6<br>26.1/93.6	35.0/99.1<br>19.5/96.8	83.2/100<br>18.9/98.4
CLEVER:4	58.5/98.7<br>14.3/98.9	13.3/100<br>6.0/73.7	62.1/98.0<br>14.4/96.5	58.0/97.7<br>12.4/69.3	40.9/98.3<br>8.6/91.5	63.1/92.6<br>17.9/93.6	74.2/98.0<br>13.3/97.5	64.4/65.6<br>10.0/97.0	77.6/98.7<br>18.0/97.4	82.7/98.2<br>22.1/93.5	66.9/100<br>21.6/97.7	79.3/89.1<br>18.2/83.6	64.8/99.4<br>15.6/98.9	80.4/59.8<br>24.8/86.9	79.3/98.3<br>16.9/96.8	66.6/99.6<br>19.6/97.4	83.5/97.1<br>27.4/87.7	32.4/97.2<br>21.1/91.4	75.0/100<br>18.1/98.6
CNVnator:4	40.1/94.7<br>6.0/98.9	1.1/100<br>1.1/66.4	38.8/95.2<br>5.9/79.7	38.6/99.4<br>3.9/98.9	19.8/99.2<br>2.0/100	38.6/91.4<br>4.1/99.0	37.3/99.3<br>3.3/98.1	33.2/99.5<br>2.9/100	38.5/99.6<br>3.9/99.8	40.8/99.2<br>4.5/99.4	31.3/99.7<br>4.2/99.5	50.3/95.6<br>4.9/96.4	34.1/99.7<br>3.4/99.7	50.7/95.1<br>6.5/99.3	39.3/99.6<br>3.9/100	44.5/99.4<br>6.4/98.8	44.2/99.3<br>6.4/98.0	19.4/97.3<br>5.1/97.4	37.8/99.5<br>3.7/100
CREST:3	25.2/98.7<br>3.7/99.7	4.4/100<br>1.4/75.0	26.4/99.1<br>3.2/99.7	26.7/100<br>3.4/98.2	17.2/100<br>1.8/99.1	26.1/97.1<br>3.7/99.2	32.8/100<br>3.0/99.2	29.2/100<br>3.9/94.4	34.0/99.7<br>5.9/97.3	35.8/99.7<br>6.7/97.1	30.0/100<br>5.9/97.7	34.4/98.6<br>4.4/96.4	29.7/100<br>3.6/100	34.9/98.9<br>5.9/98.7	35.4/100<br>5.0/98.3	29.7/100<br>4.7/99.5	36.0/99.7<br>6.8/95.9	10.6/100<br>5.2/99.4	33.7/100<br>6.1/98.2
DELLY:5	68.1/98.8<br>15.1/97.5	13.2/100<br>6.2/74.0	71.3/98.6<br>16.0/97.5	68.3/100<br>12.8/70.9	45.6/99.8<br>8.8/87.1	73.0/96.6<br>19.0/91.2	83.8/99.6<br>13.6/95.8	73.7/99.7<br>11.2/96.4	87.3/99.9<br>19.6/96.5	93.3/99.7<br>24.1/91.6	77.4/99.9<br>23.4/96.1	90.6/98.4<br>19.2/86.1	72.9/99.6<br>16.9/98.8	92.0/98.3<br>26.2/90.4	89.8/99.9<br>17.6/95.2	76.6/100<br>21.6/97.1	93.8/99.5<br>30.0/86.2	35.8/99.7<br>21.2/91.0	85.0/100<br>19.8/98.0
ERDS:3	59.6/97.8<br>11.0/99.7	11.9/100<br>4.3/73.7	66.6/96.7<br>14.6/92.3	57.7/99.0<br>8.5/74.4	39.4/99.4<br>6.5/98.1	65.4/96.1<br>13.7/96.6	72.4/99.5<br>9.5/98.8	64.4/99.7<br>8.9/100	75.0/99.6<br>13.5/99.9	79.1/99.6<br>16.0/99.1	66.0/99.6<br>15.9/99.9	77.5/98.0<br>14.1/93.0	68.3/99.5<br>12.6/98.5	78.4/97.7<br>17.6/98.5	76.4/99.5<br>12.1/100	71.1/99.7<br>18.0/97.0	80.7/99.1<br>20.7/96.9	29.9/99.4<br>15.0/97.8	72.7/99.7<br>13.3/99.9
FermiKit:5	53.5/98.4<br>10.5/99.4	11.7/100<br>4.3/74.8	55.6/99.0<br>9.5/99.5	52.9/99.8<br>8.9/83.9	35.3/99.7<br>5.7/96.8	57.5/96.5<br>13.1/98.6	66.9/99.8<br>9.2/99.6	63.8/99.8<br>9.3/96.7	71.4/100<br>15.6/97.9	73.1/99.8<br>17.4/97.8	61.0/99.8<br>16.2/98.2	70.7/98.6<br>11.3/96.9	59.9/99.8<br>11.3/100	71.4/98.2<br>17.4/97.9	72.2/100<br>13.1/98.5	59.7/100<br>13.0/99.7	73.2/99.7<br>18.8/96.6	28.5/99.7<br>14.0/98.5	70.7/100<br>15.6/98.8
forestSV:6	48.2/90.4<br>10.2/99.3	9.3/97.5<br>3.4/74.7	50.9/81.0<br>13.1/73.3	47.4/94.9<br>7.7/86.4	31.8/94.3<br>6.1/99.8	52.1/61.5<br>13.7/94.9	58.8/95.3<br>8.0/98.2	52.9/96.9<br>7.4/99.1	60.7/95.9<br>11.3/99.4	64.9/95.3<br>13.7/99.1	51.2/95.5<br>13.3/99.4	63.4/63.5<br>14.5/91.2	55.6/96.6<br>10.8/98.6	65.3/64.0<br>18.6/96.1	62.9/95.5<br>10.9/99.5	67.6/96.7<br>18.4/97.3	74.0/94.6<br>20.2/97.5	28.2/75.5<br>15.2/96.9	59.2/95.3<br>11.3/99.5
GASVPro:7	11.6/96.8<br>9.4/98.2	2.6/100<br>3.3/74.9	12.6/100<br>8.1/99.4	12.5/99.3<br>8.3/84.8	8.6/100<br>5.8/96.1	13.4/95.6<br>11.6/95.4	16.4/99.5<br>7.8/99.5	14.5/99.4<br>5.6/98.3	16.9/100<br>10.4/98.5	18.0/99.5<br>12.4/97.4	14.7/100<br>12.7/96.9	17.9/97.0<br>11.9/94.8	13.4/99.4<br>9.8/99.5	18.2/97.1<br>16.1/95.8	17.7/100<br>11.5/98.9	14.7/99.4<br>12.8/99.4	18.8/99.1<br>17.1/93.8	4.2/100<br>13.9/97.1	16.1/100<br>10.4/98.9
GenomeSTRiP:7	67.4/98.1<br>14.7/97.9	13.3/100<br>5.1/74.8	69.8/98.0<br>13.5/99.0	67.4/99.7<br>12.3/77.0	45.4/99.6<br>7.9/91.0	71.2/91.9<br>15.9/94.3	83.3/99.7<br>11.9/98.2	73.7/99.7<br>9.7/97.4	86.3/99.9<br>16.9/97.5	91.9/99.6<br>20.4/95.4	77.0/99.8<br>20.6/96.3	87.0/93.2<br>16.8/92.7	73.0/99.8<br>15.0/99.4	89.3/93.6<br>22.7/93.8	89.2/99.9<br>16.3/97.6	75.7/100<br>18.3/98.8	92.9/99.5<br>24.6/92.2	35.8/97.5<br>18.9/94.8	84.9/99.9<br>17.5/98.3
GRIDSS:6	68.0/98.5<br>14.7/98.1	13.0/100<br>6.0/74.6	71.2/98.8<br>15.4/99.1	68.3/99.8<br>12.5/90.2	45.9/99.6<br>8.6/95.0	73.1/96.6<br>18.2/95.2	83.8/99.7<br>13.3/98.6	73.9/99.6<br>11.0/95.5	87.3/99.9<br>19.5/96.8	93.1/99.7<br>23.8/95.2	77.1/99.8<br>22.9/96.5	90.6/98.8<br>18.2/93.7	72.8/100<br>16.7/99.5	91.6/98.2<br>25.0/94.6	90.1/99.8<br>17.5/97.9	76.4/100<br>20.3/99.1	93.9/99.5<br>28.3/91.6	36.0/99.5<br>20.6/95.8	85.0/100<br>19.7/98.0
Hydra-sv:7	10.6/74.1<br>9.0/94.8	2.0/100<br>0.9/70.4	30.9/71.4<br>8.8/90.9	8.1/91.3<br>7.9/59.0	4.1/88.5<br>5.5/67.6	55.4/46.3<br>17.3/88.8	11.2/85.4<br>5.6/94.3	6.3/9.0<br>4.8/70.7	8.5/90.9<br>8.3/90.4	11.3/89.6<br>10.5/75.0	8.3/99.0<br>10.8/84.1	70.2/53.1<br>17.3/88.9	7.6/80.4<br>8.0/91.3	72.2/32.7<br>26.0/82.0	10.6/92.6<br>10.9/95.9	54.2/98.6<br>14.0/96.3	51.2/94.6<br>17.3/85.6	30.1/65.5<br>18.6/90.3	8.8/92.7<br>8.6/94.9
inGAP-sv:7	65.4/98.6<br>14.1/99.2	12.6/100<br>5.7/74.8	68.4/98.7<br>14.5/99.0	64.4/100<br>11.7/73.4	43.2/100<br>7.9/97.1	70.1/97.3<br>17.7/96.8	79.4/99.8<br>12.3/99.2	70.8/100<br>10.1/99.1	82.5/100<br>18.1/98.8	87.3/100<br>21.7/98.3	73.4/100<br>21.8/98.3	85.3/98.8<br>16.7/96.1	72.1/99.7<br>16.2/99.3	86.8/98.7<br>23.8/97.1	84.9/100<br>16.4/98.9	74.6/100<br>19.1/98.4	88.3/99.9<br>26.0/96.2	34.2/100<br>19.7/97.6	81.6/100<br>18.9/99.1
Lumpy:6	67.4/98.6<br>14.7/97.8	13.2/100<br>5.9/74.7	69.1/98.7<br>14.9/99.2	66.6/99.7<br>12.0/82.3	44.3/99.6<br>8.0/94.4	71.4/96.5<br>17.7/94.8	82.3/99.7<br>12.9/98.4	73.4/99.6<br>11.2/95.5	85.3/99.9<br>18.9/97.5	90.4/99.7<br>22.9/95.4	76.0/99.7<br>22.6/96.7	88.0/98.6<br>17.3/93.1	72.8/99.7<br>16.6/98.9	89.2/98.1<br>24.3/94.9	88.0/99.8<br>17.0/98.1	74.5/100<br>19.8/98.4	91.1/99.5<br>27.4/91.4	35.5/99.5<br>20.1/96.0	84.5/100<br>19.7/97.9
Manta:3	66.4/98.6<br>14.5/98.1	12.6/100<br>5.7/74.7	69.3/98.9<br>14.5/99.3	66.3/100<br>12.0/83.9	44.2/99.8<br>8.2/95.6	70.7/96.9<br>17.4/96.2	81.3/99.7<br>12.3/99.2	72.4/99.7<br>10.9/96.7	84.7/99.9<br>18.7/97.1	89.7/99.9<br>22.4/96.5	75.0/100<br>22.3/96.8	87.2/98.8<br>16.9/94.7	72.4/100<br>16.0/99.5	88.6/98.6<br>24.0/95.6	87.0/99.9<br>16.9/98.3	75.4/100<br>19.7/98.8	90.5/99.8<br>26.7/93.4	35.2/100<br>19.6/97.0	83.3/100<br>19.1/98.5
MATCHCLIP:4	60.5/98.6<br>12.1/99.6	12.6/100<br>5.0/74.8	64.4/98.5<br>11.5/99.4	59.3/99.3<br>10.2/84.6	40.8/99.0<br>6.9/97.4	65.7/96.7<br>15.3/98.5	74.6/99.7<br>11.0/99.7	67.1/99.8<br>9.0/99.9	77.5/99.5<br>15.7/99.5	81.2/99.4<br>18.4/99.2	67.9/99.8<br>18.5/98.9	79.0/98.4<br>13.3/98.3	67.2/99.8<br>13.6/99.8	80.3/98.0<br>20.2/98.4	79.4/99.5<br>14.3/99.6	68.8/100<br>15.4/99.4	81.8/99.3<br>21.3/98.6	31.9/99.2<br>16.9/99.1	76.3/100<br>16.4/99.5
Meerkat:7	63.2/98.7<br>11.2/99.6	11.7/100<br>3.8/74.9	65.7/97.8<br>10.2/99.3	63.3/98.0<br>9.9/73.1	41.2/98.1<br>5.4/96.7	67.4/95.3<br>12.9/97.5	77.4/99.1<br>9.0/99.5	70.6/98.8<br>9.8/98.8	82.7/98.2<br>16.1/97.9	86.3/98.0<br>18.7/97.6	71.4/99.8<br>16.6/98.5	83.4/96.3<br>13.0/95.8	67.2/100<br>12.4/99.8	84.6/96.4<br>17.8/96.9	84.3/98.0<br>14.4/97.8	71.5/100<br>13.8/99.8	86.6/97.2<br>19.5/96.4	33.8/98.2<br>14.7/98.2	80.6/99.8<br>16.6/99.3
MetaSV:3	67.0/94.9<br>15.6/97.1	1.9/100<br>3.3/74.8	51.5/98.6<br>8.5/99.4	63.4/99.6<br>11.6/89.0	31.9/99.7<br>5.1/97.4	51.8/93.0<br>9.2/99.1	60.0/99.4<br>8.3/99.8	53.4/100<br>6.2/99.2	62.3/99.8<br>10.5/99.5	65.7/99.6<br>12.4/98.8	51.8/99.7<br>11.9/98.9	65.0/96.6<br>10.7/98.3	53.2/100<br>9.0/99.9	65.4/96.1<br>14.0/98.8	64.2/99.8<br>11.8/99.6	60.0/99.7<br>11.6/99.5	66.4/99.6<br>14.6/97.6	26.5/97.1<br>12.1/98.3	61.3/99.8<br>10.6/99.6
pbsv:3	1.9/100<br>3.3/74.8	13.5/100<br>9.0/92.9	9.5/99.2<br>3.6/73.0	0.9/100<br>3.3/74.8	6.6/100<br>2.0/74.5	12.2/95.7<br>4.5/74.5	13.4/100<br>8.8/94.5	10.8/100<br>2.5/75.0	12.6/100<br>4.2/74.8	13.2/100<br>5.1/74.7	12.6/100<br>5.2/74.7	12.7/95.4<br>2.9/73.9	11.1/99.3<br>4.4/74.9	12.7/94.8<br>5.9/74.6	12.7/100<br>4.7/99.8	7.9/100<br>4.6/74.5	13.5/100<br>6.4/73.5	4.9/100<br>5.7/73.7	11.9/100<br>4.5/74.9
PennCNV-Seq:3	51.5/98.6<br>8.5/99.4	9.5/99.2<br>3.6/73.0	74.7/69.5<br>25.0/10.5	49.2/98.1<br>7.2/70.2	33.0/98.8<br>5.3/92.6	58.1/80.7<br>11.6/90.6	57.5/98.4<br>7.3/95.9	50.8/98.3<br>6.9/98.1	59.5/98.6<br>10.0/98.8	63.5/98.2<br>12.2/94.9	50.6/98.2<br>12.3/99.4	70.3/83.1<br>12.7/77.0	51.9/98.5<br>9.6/96.5	71.0/82.7<br>15.4/91.6	61.0/98.3<br>8.9/98.0	64.1/98.8<br>15.7/95.9	70.2/98.6<br>17.5/91.4	27.9/90.0<br>12.7/87.8	57.8/97.9<br>9.8/99.2
Pindel:4	63.4/99.6<br>11.6/89.0	0.9/100<br>3.3/74.8	49.2/98.1<br>7.2/70.2	69.1/96.2<br>13.5/53.7	32.7/98.6<br>5.3/59.2	51.3/95.8<br>7.5/68.6	61.8/98.5<br>8.1/90.9	54.8/99.7<br>5.3/77.4	64.5/98.1<br>10.2/75.4	68.7/98.0<br>11.9/65.7	53.3/99.8<br>11.1/87.6	67.3/97.0<br>10.3/55.4	52.9/100<br>8.2/74.8	67.8/98.2<br>12.4/63.6	66.7/98.0<br>12.5/91.8	60.9/100<br>9.3/81.7	68.4/97.5<br>13.2/68.3	26.7/98.0<br>11.3/59.8	63.1/99.8<br>9.7/79.8
PRISM:3	31.9/99.7<br>5.1/97.4	6.6/100<br>2.0/74.5	33.0/98.8<br>5.3/92.6	32.7/98.6<br>5.3/59.2	46.6/97.7<br>9.2/65.4	36.8/95.8<br>5.9/86.4	40.8/99.2<br>4.6/72.9	34.6/99.7<br>2.6/82.8	43.5/98.4<br>6.2/93.1	46.1/98.1<br>7.1/66.9	37.4/99.7<br>7.6/95.4	44.4/96.8<br>6.9/66.4	35.3/99.7<br>5.1/99.4	45.1/96.7<br>8.2/73.3	43.8/98.7<br>7.8/77.1	37.7/100<br>6.8/99.6	46.2/98.2<br>9.2/90.2	17.2/98.2<br>7.6/70.7	41.4/100<br>5.1/85.5
RAPTR:10	51.8/93.0<br>9.2/99.1	12.2/95.7<br>4.5/74.5	58.1/80.7<br>11.6/90.6	51.3/95.8<br>7.5/68.6	36.8/95.8<br>5.9/86.4	55.4/38.2<br>28.2/63.3	16.2/91.0<br>6.6/95.4	11.5/54.7<br>5.3/86.5	13.2/91.5<br>9.3/94.8	16.2/88.2<br>11.9/84.0	12.3/89.9<br>11.6/95.4	58.4/52.0<br>13.7/67.4	11.2/92.4<br>8.8/95.4	59.7/46.3<br>22.2/82.3	15.5/90.5<br>11.0/95.5	51.8/96.8<br>13.3/93.6	52.4/92.9<br>17.8/82.4	23.7/64.3<br>16.0/87.4	13.9/89.1<br>9.9/95.2
Sniffles:2	60.0/99.4<br>8.3/99.8	13.4/100<br>8.8/94.5	57.5/98.4<br>7.3/95.9	61.8/98.5<br>8.1/90.9	40.8/99.2<br>4.6/72.9	16.2/91.0<br>6.6/95.4	85.4/85.2<br>15.6/82.2	67.3/98.7<br>5.3/98.9	78.7/99.4<br>9.2/98.7	84.0/99.0<br>11.1/88.9	68.6/99.8<br>11.3/99.1	82.3/95.9<br>9.1/90.4	66.7/99.6<br>9.6/99.6	82.8/96.4<br>12.9/91.3	81.2/99.1<br>8.2/88.2	69.1/99.7<br>9.9/98.6	84.6/98.6<br>14.1/96.4	32.2/96.9<br>12.4/87.5	77.4/99.8<br>9.8/98.5
Socrates:3	53.4/100<br>6.2/99.2	10.8/100<br>2.5/75.0	50.8/98.3<br>6.9/98.1	54.8/99.7<br>5.3/77.4	34.6/99.7<br>2.6/82.8	11.5/54.7<br>5.3/86.5	67.3/98.7<br>5.3/98.9	74.2/52.5<br>11.8/84.6	71.5/99.7<br>9.8/97.8	74.0/99.7<br>11.1/97.0	61.7/99.8<br>9.9/98.0	71.7/98.7<br>6.9/97.2	60.9/97.7<br>7.2/99.3	71.9/82.4<br>10.3/90.3	73.8/99.7<br>8.4/98.3	61.3/99.8<br>8.8/99.9	73.8/99.5<br>11.4/94.9	28.9/99.7<br>8.1/98.5	72.3/99.7<br>10.2/99.1
SoftSV:4	62.3/99.8<br>10.5/99.5	12.6/100<br>4.2/74.8	59.5/98.6<br>10.0/98.8	64.5/98.1<br>10.2/75.4	43.5/98.4<br>6.2/93.1	13.2/91.5<br>9.3/94.8	78.7/99.4<br>9.2/98.7	71.5/99.7<br>9.8/97.8	89.1/97.8<br>20.1/94.8	88.4/98.3<br>19.1/96.9	72.2/100<br>17.0/98.3	85.8/97.0<br>12.9/95.6	69.1/99.7<br>12.2/100	86.3/97.6<br>17.8/97.5	86.3/98.5<br>15.8/97.9	72.0/100<br>13.8/99.8	88.5/98.3<br>19.8/96.1	34.3/98.6<br>14.8/98.1	81.4/100<br>16.4/98.5
Sprites:3	65.7/99.6<br>12.4/98.8	13.2/100<br>5.1/74.7	63.5/98.2<br>12.2/94.9	68.7/98.0<br>11.9/65.7	46.1/98.1<br>7.1/66.9	16.2/88.2<br>11.9/84.0	84.0/99.0<br>11.1/88.9	74.0/99.7<br>11.1/97.0	88.4/98.3<br>19.1/96.9	95.0/96.0<br>25.7/66.4	76.8/99.8<br>19.9/97.8	91.0/95.4<br>16.3/78.2	72.4/100<br>14.7/99.7	91.4/96.3<br>21.9/80.1	90.7/97.7<br>17.1/79.0	76.1/99.7<br>17.0/98.3	94.3/97.1<br>24.8/88.3	36.3/97.7<br>18.0/75.0	84.9/99.9<br>19.1/96.2
SvABA:3	51.8/99.7<br>11.9/98.9	12.6/100<br>5.2/74.7	50.6/98.2<br>12.3/99.4	53.3/99.8<br>11.1/87.6	37.4/99.7<br>7.6/95.4	12.3/89.9<br>11.6/95.4	68.6/99.8<br>11.3/99.1	61.7/99.8<br>9.9/98.0	72.2/100<br>17.0/98.3	76.8/99.8<br>19.9/97.8	77.8/99.8<br>23.7/95.9	74.5/98.8<br>15.6/96.7	61.0/99.8<br>14.5/99.6	75.8/98.5<br>21.5/97.1	74.5/100<br>15.8/98.6	61.9/100<br>17.0/99.3	77.3/99.8<br>23.5/96.2	29.7/99.4<br>18.1/97.8	70.7/100<br>17.2/98.7
SVDetect:6	65.0/96.6<br>10.7/98.3	12.7/95.4<br>2.9/73.9	70.3/83.1<br>12.7/77.0	67.3/97.0<br>10.3/55.4	44.4/96.8<br>6.9/66.4	58.4/52.0<br>13.7/67.4	82.3/95.9<br>9.1/90.4	71.7/98.7<br>6.9/97.2	85.8/97.0<br>12.9/95.6	91.0/95.4<br>16.3/78.2	74.5/98.8<br>15.6/96.7	71.4/44.1<br>23.1/42.2	8.6/96.5<br>8.7/94.5	70.0/51.7<br>17.5/74.4	11.1/87.3<br>11.8/81.9	60.2/98.2<br>14.4/90.0	58.9/88.9<br>18.7/70.5	28.5/64.1<br>15.0/68.3	9.4/91.7<br>9.0/86.3
SVelter:3	53.2/100<br>9.0/99.9	11.1/99.3<br>4.4/74.9	51.9/98.5<br>9.6/96.5	52.9/100<br>8.2/74.8	35.3/99.7<br>5.1/99.4	11.2/92.4<br>8.8/95.4	66.7/99.6<br>9.6/99.6	60.9/97.7<br>7.2/99.3	69.1/99.7<br>12.2/100	72.4/100<br>14.7/99.7	61.0/99.8<br>14.5/99.6	8.6/96.5<br>8.7/94.5	73.1/90.8<br>17.3/82.9	72.0/96.6<br>15.1/98.7	71.0/100<br>12.1/100	64.5/100<br>13.0/98.1	73.4/99.6<br>17.0/97.9	28.5/100<br>13.2/99.0	68.6/99.5<br>12.9/99.7
SVfinder:10	65.4/96.1<br>14.0/98.8	12.7/94.8<br>5.9/74.6	71.0/82.7<br>15.4/91.6	67.8/98.2<br>12.4/63.6	45.1/96.7<br>8.2/73.3	59.7/46.3<br>22.2/82.3	82.8/96.4<br>12.9/91.3	71.9/82.4<br>10.3/90.3	86.3/97.6<br>17.8/97.5	91.4/96.3<br>21.9/80.1	75.8/98.5<br>21.5/97.1	70.0/51.7<br>17.5/74.4	72.0/96.6<br>15.1/98.7	73.1/31.2<br>29.4/64.5	11.0/91.1<br>11.2/76.3	61.5/98.5<br>13.3/94.3	59.6/94.7<br>16.8/77.5	29.9/65.8<br>18.0/79.0	9.8/92.0<br>9.0/92.4
SVseq2:5	64.2/99.8<br>11.8/99.6	12.7/100<br>4.7/99.8	61.0/98.3<br>8.9/98.0	66.7/98.0<br>12.5/91.8	43.8/98.7<br>7.8/77.1	15.5/90.5<br>11.0/95.5	81.2/99.1<br>8.2/88.2	73.8/99.7<br>8.4/98.3	86.3/98.5<br>15.8/97.9	90.7/97.7<br>17.1/79.0	74.5/100<br>15.8/98.6	11.1/87.3<br>11.8/81.9	71.0/100<br>12.1/100	11.0/91.1<br>11.2/76.3	91.0/97.4<br>18.0/89.7	73.2/100<br>11.8/100	90.5/97.8<br>17.8/96.7	35.3/98.2<br>15.3/92.7	83.9/100<br>14.8/98.8
TIDDIT:3	60.0/99.7<br>11.6/99.5	7.9/100<br>4.6/74.5	64.1/98.8<br>15.7/95.9	60.9/100<br>9.3/81.7	37.7/100<br>6.8/99.6	51.8/96.8<br>13.3/93.6	69.1/99.7<br>9.9/98.6	61.3/99.8<br>8.8/99.9	72.0/100<br>13.8/99.8	76.1/99.7<br>17.0/98.3	61.9/100<br>17.0/99.3	60.2/98.2<br>14.4/90.0	64.5/100<br>13.0/98.1	61.5/98.5<br>13.3/94.3	73.2/100<br>11.8/100	77.0/99.4<br>23.2/91.2	76.8/99.9<br>22.6/94.1	29.0/99.7<br>15.6/97.4	69.5/100<br>13.3/99.5
Ulysses:4	66.4/99.6<br>14.6/97.6	13.5/100<br>6.4/73.5	70.2/98.6<br>17.5/91.4	68.4/97.5<br>13.2/68.3	46.2/98.2<br>9.2/90.2	52.4/92.9<br>17.8/82.4	84.6/98.6<br>14.1/96.4	73.8/99.5<br>11.4/94.9	88.5/98.3<br>19.8/96.1	94.3/97.1<br>24.8/88.3	77.3/99.8<br>23.5/96.2	58.9/88.9<br>18.7/70.5	73.4/99.6<br>17.0/97.9	59.6/94.7<br>16.8/77.5	90.5/97.8<br>17.8/96.7	76.8/99.9<br>22.6/94.1	96.2/94.4<br>33.4/69.2	36.4/97.3<br>22.1/87.8	84.7/99.8<br>19.7/97.6
VariationHunter:6	26.5/97.1<br>12.1/98.3	4.9/100<br>5.7/73.7	27.9/90.0<br>12.7/87.8	26.7/98.0<br>11.3/59.8	17.2/98.2<br>7.6/70.7	23.7/64.3<br>16.0/87.4	32.2/96.9<br>12.4/87.5	28.9/99.7<br>8.1/98.5	34.3/98.6<br>14.8/98.1	36.3/97.7<br>18.0/75.0	29.7/99.4<br>18.1/97.8	28.5/64.1<br>15.0/68.3	28.5/100<br>13.2/99.0	29.9/65.8<br>18.0/79.0	35.3/98.2<br>15.3/92.7	29.0/99.7<br>15.6/97.4	36.4/97.3<br>22.1/87.8	33.1/65.5<br>24.0/44.2	26.2/99.1<br>11.6/97.8
Wham:4	61.3/99.8<br>10.6/99.6	11.9/100<br>4.5/74.9	57.8/97.9<br>9.8/99.2	63.1/99.8<br>9.7/79.8	41.4/100<br>5.1/85.5	13.9/89.1<br>9.9/95.2	77.4/99.8<br>9.8/98.5	72.3/99.7<br>10.2/99.1	81.4/100<br>16.4/98.5	84.9/99.9<br>19.1/96.2	70.7/100<br>17.2/98.7	9.4/91.7<br>9.0/86.3	68.6/99.5<br>12.9/99.7	9.8/92.0<br>9.0/92.4	83.9/100<br>14.8/98.8	69.5/100<br>13.3/99.5	84.7/99.8<br>19.7/97.6	26.2/99.1<br>11.6/97.8	85.0/98.8<br>20.0/95.3

Figure S17. Recall and precision of SVs commonly called between a pair of SV detection algorithms for the DEL-M category. DELs in the middle size-range (1 Kb–100 Kb), called using the indicated algorithms, were filtered with the minimum number of reads supporting the called SVs, indicated with the suffix number of the algorithm name. The DELs overlapping between the filtered SV sets from a pair of the indicated algorithms were selected, and the recall and precision of the selected DELs were determined. Recall and precision percentages are presented as in Figure S15. The data contained in the top 20th percentile of the combined precision scores for the simulated and real data are highlighted with a red background, and the next data contained in the top 21st–50th percentile of the combined precision scores are shown with a pale red background.

Algorithm	1-2-3-SV:7	BICseq2:8	BreakDancer:3	CNVnator:3	Control-FREEC:3	CREST:5	DELLY:8	ERDS:3	FermiKit:5	forestSV:6	GASVPro:5	GenomeSTRiP:4	GRIDSS:6	Hydra-sv:8	iCopyDAV:3	Lumpy:4	Manta:3	Meerkat:6
1-2-3-SV:7	91.5/79.7<br>11.4/20.8	16.2/96.4<br>1.0/100	4.8/100<br>1.8/23.9	35.5/95.1<br>2.6/67.9	45.7/86.5<br>4.1/100	1.8/75.0<br>0.5/50.0	6.0/71.4<br>3.1/19.4	4.2/100<br>3.1/70.8	3.0/83.3<br>1.3/35.4	59.0/98.0<br>4.4/100	20.4/91.8<br>3.9/22.4	9.0/75.0<br>4.4/20.0	5.4/90.0<br>4.1/27.5	89.1/85.7<br>6.2/29.3	2.4/66.6<br>3.1/100	5.4/81.8<br>3.3/22.1	4.2/87.5<br>3.3/28.1	4.8/57.1<br>2.3/21.0
BICseq2:8	16.2/96.4<br>1.0/100	40.3/61.4<br>5.7/62.8	43.3/92.3<br>1.5/100	31.9/86.8<br>2.8/72.9	19.8/76.7<br>3.1/74.2	14.4/96.0<br>0.5/50.0	43.9/92.4<br>1.5/100	43.9/90.1<br>2.3/100	30.1/89.2<br>0.3/25.0	40.3/83.7<br>3.6/100	12.0/90.9<br>1.5/100	44.5/92.5<br>3.1/100	44.5/92.5<br>2.8/100	44.5/98.6<br>1.5/75.0	7.2/75.0<br>2.6/86.7	43.3/92.3<br>1.8/100	43.3/92.3<br>2.0/100	41.5/94.5<br>2.0/100
BreakDancer:3	4.8/100<br>1.8/23.9	43.3/92.3<br>1.5/100	84.3/24.8<br>5.7/35.1	56.0/96.8<br>2.8/100	45.7/86.3<br>3.1/100	28.3/97.9<br>0.3/25.0	82.5/97.1<br>5.2/42.4	72.2/98.3<br>4.1/100	63.2/98.1<br>2.8/62.9	69.2/95.8<br>3.3/100	21.6/100<br>3.4/34.9	83.1/97.1<br>5.7/38.9	82.5/97.1<br>5.4/45.8	82.5/80.1<br>3.9/48.2	9.0/100<br>2.3/100	83.1/97.1<br>5.7/40.8	82.5/97.1<br>4.9/45.3	75.3/99.2<br>2.8/62.5
CNVnator:3	35.5/95.1<br>2.6/67.9	31.9/86.8<br>2.8/72.9	56.0/96.8<br>2.8/100	84.3/90.9<br>11.4/37.8	46.3/82.7<br>3.9/64.9	18.6/96.8<br>0/0	56.0/96.8<br>2.8/93.8	53.0/96.7<br>4.4/86.6	43.9/96.0<br>2.0/100	60.2/90.9<br>4.1/83.8	16.8/100<br>2.0/79.2	58.4/96.0<br>3.4/82.5	56.0/96.8<br>3.6/72.5	76.5/96.2<br>2.8/66.3	16.2/93.1<br>4.7/50.9	55.4/96.8<br>3.1/93.8	54.8/95.7<br>3.1/100	55.4/95.8<br>2.3/76.7
Control-FREEC:3	45.7/86.5<br>4.1/100	19.8/76.7<br>3.1/74.2	45.7/86.3<br>3.1/100	46.3/82.7<br>3.9/64.9	31.3/35.2<br>10.1/61.6	1.2/100<br>0/0	6.0/100<br>1.3/75.0	5.4/90.0<br>2.0/100	3.6/100<br>0.3/25.0	4.8/88.8<br>1.8/100	2.4/100<br>1.0/100	6.0/100<br>1.8/100	6.0/100<br>2.6/100	6.6/91.6<br>1.8/81.3	3.0/83.3<br>2.3/100	6.0/100<br>1.3/75.0	5.4/100<br>1.3/100	5.4/100<br>2.3/100
CREST:5	1.8/75.0<br>0.5/50.0	14.4/96.0<br>0.5/50.0	28.3/97.9<br>0.3/25.0	18.6/96.8<br>0/0	1.2/100<br>0/0	30.7/42.8<br>1.0/29.2	29.5/100<br>0.3/25.0	24.6/100<br>0.3/25.0	24.6/100<br>0.3/25.0	22.2/94.8<br>0.5/50.0	14.4/100<br>0.3/25.0	30.1/100<br>0.3/25.0	29.5/100<br>0.5/50.0	29.5/100<br>0/0	3.0/83.3<br>0.8/75.0	28.3/100<br>0.5/50.0	29.5/100<br>0.5/50.0	28.9/100<br>0.8/75.0
DELLY:8	6.0/71.4<br>3.1/19.4	43.9/92.4<br>1.5/100	82.5/97.1<br>5.2/42.4	56.0/96.8<br>2.8/93.8	6.0/100<br>1.3/75.0	29.5/100<br>0.3/25.0	89.1/93.6<br>7.0/32.9	76.5/99.2<br>5.4/100	65.0/98.1<br>2.8/66.7	71.6/95.2<br>3.9/100	22.8/92.6<br>3.6/28.0	87.9/96.0<br>7.0/33.3	87.3/97.9<br>6.7/44.2	86.7/94.1<br>5.2/38.8	7.8/92.8<br>2.3/100	85.5/96.5<br>6.7/37.3	85.5/97.9<br>6.0/41.4	78.9/99.2<br>2.8/54.3
ERDS:3	4.2/100<br>3.1/70.8	43.9/90.1<br>2.3/100	72.2/98.3<br>4.1/100	53.0/96.7<br>4.4/86.6	5.4/90.0<br>2.0/100	24.6/100<br>0.3/25.0	76.5/99.2<br>5.4/100	78.9/96.3<br>8.0/95.0	57.2/98.9<br>3.1/100	69.8/94.3<br>4.4/100	19.8/100<br>4.1/100	75.9/99.2<br>6.2/100	75.9/99.2<br>6.2/100	75.3/99.2<br>4.4/100	7.2/92.3<br>2.3/100	74.6/99.2<br>5.2/100	74.0/99.1<br>5.2/100	71.0/99.1<br>3.9/100
FermiKit:5	3.0/83.3<br>1.3/35.4	30.1/89.2<br>0.3/25.0	63.2/98.1<br>2.8/62.9	43.9/96.0<br>2.0/100	3.6/100<br>0.3/25.0	24.6/100<br>0.3/25.0	65.0/98.1<br>2.8/66.7	57.2/98.9<br>3.1/100	65.6/99.0<br>3.1/50.0	53.6/95.6<br>2.1/75.0	15.0/100<br>2.0/52.5	65.6/99.0<br>2.8/53.2	65.0/99.0<br>2.8/56.7	64.4/99.0<br>1.8/79.2	4.2/100<br>1.5/100	64.4/99.0<br>2.8/58.8	65.0/99.0<br>2.8/58.8	59.6/99.0<br>2.0/83.3
forestSV:6	59.0/98.0<br>4.4/100	40.3/83.7<br>3.6/100	69.2/95.8<br>3.3/100	60.2/90.9<br>4.1/83.8	4.8/88.8<br>1.8/100	22.2/94.8<br>0.5/50.0	71.6/95.2<br>3.9/100	69.8/94.3<br>4.4/100	53.6/95.6<br>2.1/75.0	72.8/84.7<br>8.6/63.1	16.8/96.5<br>2.3/100	61.4/93.5<br>4.4/100	58.4/93.2<br>3.6/100	72.2/97.5<br>3.1/100	6.6/78.5<br>3.4/100	58.4/93.2<br>3.4/100	59.0/93.3<br>3.4/100	55.4/92.0<br>1.8/75.0
GASVPro:5	20.4/91.8<br>3.9/22.4	12.0/90.9<br>1.5/100	21.6/100<br>3.4/34.9	16.8/100<br>2.0/79.2	2.4/100<br>1.0/100	14.4/100<br>0.3/25.0	22.8/92.6<br>3.6/28.0	19.8/100<br>4.1/100	15.0/100<br>2.0/52.5	16.8/96.5<br>2.3/100	22.8/92.6<br>4.7/20.6	22.8/92.6<br>4.1/21.6	22.8/97.4<br>3.9/45.6	22.2/92.5<br>2.6/32.1	1.2/100<br>1.5/100	21.6/94.7<br>3.3/25.8	22.2/97.3<br>3.4/35.1	19.8/100<br>2.3/56.2
GenomeSTRiP:4	9.0/75.0<br>4.4/20.0	44.5/92.5<br>3.1/100	83.1/97.1<br>5.7/38.9	58.4/96.0<br>3.4/82.5	6.0/100<br>1.8/100	30.1/100<br>0.3/25.0	87.9/96.0<br>7.0/33.3	75.9/99.2<br>6.2/100	65.6/99.0<br>2.8/53.2	61.4/93.5<br>4.4/100	22.8/92.6<br>4.1/21.6	89.7/96.1<br>9.6/23.8	88.5/97.3<br>8.0/39.8	86.7/96.0<br>4.9/32.2	7.2/100<br>3.1/100	86.7/96.6<br>7.8/28.2	87.3/97.3<br>7.5/37.5	78.9/99.2<br>3.6/47.5
GRIDSS:6	5.4/90.0<br>4.1/27.5	44.5/92.5<br>2.8/100	82.5/97.1<br>5.4/45.8	56.0/96.8<br>3.6/72.5	6.0/100<br>2.6/100	29.5/100<br>0.5/50.0	87.3/97.9<br>6.7/44.2	75.9/99.2<br>6.2/100	65.0/99.0<br>2.8/56.7	58.4/93.2<br>3.6/100	22.8/97.4<br>3.9/45.6	88.5/97.3<br>8.0/39.8	89.1/96.7<br>9.6/38.6	86.1/96.6<br>5.4/45.7	7.8/100<br>3.1/100	86.1/97.2<br>7.0/42.1	86.1/97.9<br>8.0/51.4	78.9/99.2<br>4.7/60.8
Hydra-sv:8	89.1/85.7<br>6.2/29.3	44.5/98.6<br>1.5/75.0	82.5/80.1<br>3.9/48.2	76.5/96.2<br>2.8/66.3	6.6/91.6<br>1.8/81.3	29.5/100<br>0/0	86.7/94.1<br>5.2/38.8	75.3/99.2<br>4.4/100	64.4/99.0<br>1.8/79.2	72.2/97.5<br>3.1/100	22.2/92.5<br>2.6/32.1	86.7/96.0<br>4.9/32.2	86.1/96.6<br>5.4/45.7	90.3/18.6<br>8.8/10.5	2.4/66.6<br>1.8/58.3	9.6/84.2<br>2.8/29.6	8.4/87.5<br>2.6/30.3	7.2/57.1<br>1.8/20.2
iCopyDAV:3	2.4/66.6<br>3.1/100	7.2/75.0<br>2.6/86.7	9.0/100<br>2.3/100	16.2/93.1<br>4.7/50.9	3.0/83.3<br>2.3/100	3.0/83.3<br>0.8/75.0	7.8/92.8<br>2.3/100	7.2/92.3<br>2.3/100	4.2/100<br>1.5/100	6.6/78.5<br>3.4/100	1.2/100<br>1.5/100	7.2/100<br>3.1/100	7.8/100<br>3.1/100	2.4/66.6<br>1.8/58.3	39.7/69.7<br>12.7/33.2	36.7/98.3<br>3.1/100	36.7/98.3<br>3.3/100	36.7/96.8<br>3.1/95.0
Lumpy:4	5.4/81.8<br>3.3/22.1	43.3/92.3<br>1.8/100	83.1/97.1<br>5.7/40.8	55.4/96.8<br>3.1/93.8	6.0/100<br>1.3/75.0	28.3/100<br>0.5/50.0	85.5/96.5<br>6.7/37.3	74.6/99.2<br>5.2/100	64.4/99.0<br>2.8/58.8	58.4/93.2<br>3.4/100	21.6/94.7<br>3.3/25.8	86.7/96.6<br>7.8/28.2	86.1/97.2<br>7.0/42.1	9.6/84.2<br>2.8/29.6	36.7/98.3<br>3.1/100	86.7/96.6<br>8.0/29.1	86.1/97.9<br>6.5/40.2	77.1/99.2<br>3.1/54.2
Manta:3	4.2/87.5<br>3.3/28.1	43.3/92.3<br>2.0/100	82.5/97.1<br>4.9/45.3	54.8/95.7<br>3.1/100	5.4/100<br>1.3/100	29.5/100<br>0.5/50.0	85.5/97.9<br>6.0/41.4	74.0/99.1<br>5.2/100	65.0/99.0<br>2.8/58.8	59.0/93.3<br>3.4/100	22.2/97.3<br>3.4/35.1	87.3/97.3<br>7.5/37.5	86.1/97.9<br>8.0/51.4	8.4/87.5<br>2.6/30.3	36.7/98.3<br>3.3/100	86.1/97.9<br>6.5/40.2	87.3/96.6<br>8.3/36.9	77.7/99.2<br>3.9/54.9
Meerkat:6	4.8/57.1<br>2.3/21.0	41.5/94.5<br>2.0/100	75.3/99.2<br>2.8/62.5	55.4/95.8<br>2.3/76.7	5.4/100<br>2.3/100	28.9/100<br>0.8/75.0	78.9/99.2<br>2.8/54.3	71.0/99.1<br>3.9/100	59.6/99.0<br>2.0/83.3	55.4/92.0<br>1.8/75.0	19.8/100<br>2.3/56.2	78.9/99.2<br>3.6/47.5	78.9/99.2<br>4.7/60.8	7.2/57.1<br>1.8/20.2	36.7/96.8<br>3.1/95.0	77.1/99.2<br>3.1/54.2	77.7/99.2<br>3.9/54.9	82.5/87.8<br>5.7/43.7
MetaSV:3	11.4/100<br>1.8/36.9	43.9/92.4<br>1.8/100	80.7/97.8<br>2.8/59.2	57.8/96.9<br>3.6/100	5.4/100<br>0.5/50.0	25.9/97.7<br>0/0	81.9/99.2<br>2.6/49.3	74.0/99.1<br>2.6/100	62.6/98.1<br>1.8/83.3	57.8/93.2<br>2.3/100	21.0/100<br>1.8/47.5	82.5/98.5<br>3.6/55.3	82.5/99.2<br>3.1/53.6	16.2/96.4<br>1.3/36.8	36.7/98.3<br>2.6/93.8	82.5/99.2<br>2.8/51.8	81.9/98.5<br>3.1/54.3	75.9/98.4<br>1.8/83.3
OncoSNP-SEQ:3	5.4/100<br>4.4/85.8	9.6/80.0<br>4.7/97.2	10.2/100<br>3.4/100	9.6/88.8<br>5.4/70.0	2.4/100<br>2.0/100	3.0/83.3<br>0.5/50.0	10.8/100<br>3.6/100	10.2/94.4<br>4.9/100	5.4/90.0<br>1.8/75.0	7.2/85.7<br>5.7/96.4	2.4/100<br>3.1/100	10.8/94.7<br>5.4/100	10.8/100<br>4.6/100	4.8/100<br>2.5/86.7	12.0/95.2<br>4.9/68.7	10.8/100<br>4.4/100	10.8/94.7<br>4.4/100	9.6/88.8<br>2.8/100
PennCNV-Seq:3	31.9/100<br>1.8/91.7	34.3/82.6<br>1.8/57.5	59.6/98.0<br>1.0/50.0	49.3/93.1<br>3.1/61.3	6.0/90.9<br>0.5/50.0	19.8/100<br>0.8/75.0	62.0/98.0<br>1.8/75.0	60.8/96.1<br>2.1/87.5	48.7/98.7<br>1.0/50.0	51.2/90.4<br>2.8/91.7	15.0/100<br>1.3/66.7	62.0/98.0<br>2.1/93.8	62.6/98.1<br>2.3/100	32.5/98.1<br>1.0/66.7	30.1/89.2<br>1.3/47.5	60.8/98.0<br>2.1/93.8	60.8/98.0<br>1.8/75.0	57.2/98.9<br>1.8/100
Pindel:4	3.6/50.0<br>2.6/16.1	42.7/94.6<br>1.0/75.0	78.3/97.0<br>2.8/31.6	53.0/95.6<br>2.8/87.5	5.4/100<br>0.5/25.0	29.5/100<br>0.3/25.0	81.9/97.8<br>3.4/27.0	71.6/99.1<br>2.6/75.0	61.4/99.0<br>1.5/44.2	57.2/92.2<br>1.8/75.0	21.6/97.2<br>1.8/20.4	83.1/97.8<br>4.2/20.9	82.5/97.8<br>4.4/31.0	7.8/59.0<br>2.3/16.2	34.9/96.6<br>2.3/63.8	80.7/97.8<br>3.6/26.1	81.3/97.8<br>4.4/30.4	78.3/96.2<br>2.0/35.7
PRISM:3	1.8/50.0<br>0.8/4.8	19.2/96.9<br>0.5/50.0	37.3/96.8<br>1.8/51.7	24.6/97.6<br>1.3/87.5	4.2/100<br>0.3/25.0	12.0/100<br>0/0	40.3/98.5<br>2.0/46.3	34.3/100<br>1.8/100	27.7/100<br>1.0/70.8	27.7/88.4<br>1.5/100	9.6/100<br>0.5/10.4	39.7/98.5<br>2.0/41.9	39.7/98.5<br>2.3/44.0	4.2/58.3<br>1.3/24.2	15.0/100<br>1.3/66.7	38.5/98.4<br>2.0/46.0	38.5/98.4<br>2.3/45.5	35.5/89.3<br>0.8/33.3
RAPTR:4	86.1/91.1<br>5.2/18.3	43.9/98.6<br>2.6/100	79.5/97.0<br>3.9/38.9	72.8/97.6<br>5.5/83.9	6.0/90.9<br>1.8/100	27.1/100<br>0/0	84.9/97.9<br>4.7/33.6	74.0/99.1<br>5.2/100	63.2/99.0<br>1.8/45.8	69.8/98.3<br>4.9/100	21.6/100<br>3.1/24.8	85.5/99.3<br>5.7/24.2	84.3/99.2<br>5.2/34.4	84.9/78.5<br>5.7/19.0	37.3/93.9<br>4.9/79.3	83.1/99.2<br>5.1/32.8	83.7/99.2<br>4.4/31.7	77.7/94.8<br>2.8/40.3
readDepth:5	0.6/100<br>0.3/25.0	4.8/53.3<br>0.3/25.0	5.4/90.0<br>0/0	4.2/87.5<br>0.3/25.0	2.4/100<br>0/0	1.2/100<br>0.3/25.0	4.2/87.5<br>0/0	3.6/100<br>0/0	2.4/100<br>0/0	3.0/100<br>0/0	0/0<br>0/0	3.6/85.7<br>0/0	4.2/87.5<br>0/0	0.6/100<br>0/0	0/0<br>0/0	4.2/87.5<br>0/0	4.2/87.5<br>0/0	3.6/100<br>0.3/25.0
Sniffles:2	9.0/50.0<br>1.7/49.9	37.3/91.1<br>1.0/66.7	74.0/96.8<br>1.7/100	51.2/94.4<br>1.3/100	6.6/100<br>0.3/33.3	27.1/100<br>0.7/66.7	77.7/97.7<br>2.0/86.7	68.0/99.1<br>1.0/66.7	61.4/99.0<br>0.3/33.3	53.0/89.7<br>1.4/66.7	18.6/96.8<br>1.0/55.5	77.7/97.7<br>1.7/50.0	77.1/98.4<br>1.7/53.3	12.6/48.8<br>1.3/31.7	31.9/92.9<br>1.0/55.5	75.9/97.6<br>1.7/83.3	75.9/97.6<br>1.7/70.0	72.2/89.5<br>1.0/83.3
SoftSV:3	4.8/61.5<br>2.1/15.5	42.7/93.4<br>2.1/75.0	78.3/97.7<br>2.8/47.2	55.4/95.8<br>3.9/91.7	6.0/100<br>1.0/100	30.1/100<br>0.5/50.0	84.3/97.9<br>2.8/42.1	74.6/100<br>3.6/100	63.8/100<br>2.0/66.3	57.8/93.2<br>2.8/100	21.0/94.5<br>1.0/19.8	84.9/97.9<br>3.6/38.6	84.3/98.5<br>4.9/54.0	8.4/53.8<br>2.3/29.7	35.5/98.3<br>3.1/90.8	81.9/97.8<br>3.1/38.5	82.5/97.8<br>4.1/47.3	80.1/93.0<br>3.3/53.8
Sprites:3	5.4/64.2<br>3.6/20.3	43.9/92.4<br>2.8/100	81.3/97.1<br>4.6/53.9	57.2/95.9<br>3.3/80.4	5.4/100<br>1.8/100	30.7/100<br>0.5/50.0	87.3/97.3<br>4.9/48.1	75.9/99.2<br>4.9/100	65.0/99.0<br>2.3/66.7	59.6/91.6<br>3.1/93.8	22.2/94.8<br>2.6/35.4	87.9/97.3<br>6.5/39.4	87.3/97.9<br>6.7/52.3	9.6/69.5<br>3.3/32.2	36.1/96.7<br>4.2/100	84.9/97.2<br>5.2/44.4	85.5/97.2<br>6.2/53.0	82.5/93.8<br>3.9/49.6
SvABA:3	4.2/77.7<br>2.8/33.0	40.9/91.8<br>1.3/100	79.5/97.0<br>5.2/48.5	52.4/95.6<br>2.6/83.3	5.4/100<br>1.0/75.0	28.9/100<br>0.3/25.0	85.5/97.9<br>6.2/41.7	72.2/99.1<br>4.9/100	63.8/99.0<br>2.8/62.9	57.2/93.1<br>2.3/100	22.2/97.3<br>2.9/30.1	85.5/97.9<br>6.2/40.0	84.3/97.9<br>5.7/46.4	8.4/82.3<br>2.6/30.6	34.9/98.3<br>2.3/100	83.1/97.8<br>6.2/42.4	84.3/97.9<br>5.7/42.4	77.1/99.2<br>2.8/55.6
SVDetect:4	91.5/80.6<br>10.6/26.5	46.9/97.5<br>4.2/100	83.7/96.5<br>4.9/43.9	79.5/96.3<br>6.2/88.1	7.2/92.3<br>2.6/100	30.7/100<br>1.0/100	89.1/94.8<br>6.0/38.6	78.9/98.4<br>6.5/100	65.6/99.0<br>2.8/50.7	74.6/96.8<br>4.9/91.4	22.8/95.0<br>4.4/28.0	89.7/96.7<br>8.6/32.8	89.1/97.3<br>8.6/46.2	89.7/82.9<br>5.9/26.1	40.3/97.1<br>4.7/87.3	86.7/97.2<br>7.0/38.4	87.3/97.3<br>7.5/40.9	83.7/88.5<br>5.2/51.6
SVelter:3	3.6/85.7<br>1.8/61.7	33.7/91.8<br>0.5/25.0	62.0/96.2<br>2.3/100	40.9/95.7<br>2.3/93.8	1.2/100<br>1.3/100	21.6/100<br>0/0	62.6/97.1<br>2.3/100	59.0/98.9<br>3.1/100	50.0/98.8<br>2.3/100	45.7/93.8<br>1.8/100	13.2/95.6<br>1.8/50.0	62.6/97.1<br>2.6/100	62.6/97.1<br>3.1/100	5.4/69.2<br>1.0/41.7	27.7/97.8<br>2.0/100	62.0/97.1<br>2.3/100	62.0/97.1<br>2.6/100	59.6/99.0<br>2.8/86.7
SVfinder:8	91.5/80.2<br>10.7/22.9	45.7/98.7<br>2.6/100	83.1/84.6<br>5.2/38.9	77.7/96.2<br>4.9/70.8	6.6/84.6<br>2.6/82.5	30.1/100<br>0.8/75.0	89.1/93.6<br>7.0/33.7	77.7/99.2<br>6.2/100	65.0/99.0<br>3.1/64.2	73.4/97.6<br>3.9/100	22.8/92.6<br>3.9/25.9	88.5/96.0<br>7.8/28.1	87.9/97.3<br>7.8/42.3	90.3/21.8<br>8.8/15.5	38.5/91.4<br>3.6/65.3	86.1/96.6<br>7.0/33.8	86.1/96.6<br>6.5/37.1	83.1/89.6<br>4.9/51.7
SVseq2:8	3.0/55.5<br>1.0/27.8	32.5/93.1<br>0.3/33.3	67.4/99.1<br>1.7/72.2	48.7/97.5<br>1.7/100	3.0/100<br>0/0	25.9/100<br>0.3/33.3	69.2/98.2<br>1.7/72.2	62.6/100<br>1.7/100	59.6/100<br>1.7/100	51.2/93.4<br>1.3/100	15.6/96.2<br>1.0/50.0	69.2/98.2<br>2.0/72.2	69.2/99.1<br>2.4/72.2	7.8/72.2<br>0.7/22.2	25.3/97.6<br>1.3/100	68.6/98.2<br>2.4/77.7	68.0/98.2<br>2.4/77.7	65.0/95.5<br>1.3/61.1
TIDDIT:3	59.6/99.0<br>5.4/82.7	45.1/97.4<br>3.9/100	80.1/97.7<br>4.6/100	62.0/96.2<br>4.4/95.0	6.6/100<br>2.6/100	27.7/100<br>0.8/75.0	83.7/98.5<br>5.4/88.7	75.9/99.2<br>6.2/100	63.2/99.0<br>3.1/100	68.6/94.2<br>4.7/100	21.6/100<br>3.6/79.2	84.9/98.6<br>7.0/84.9	83.7/98.5<br>7.2/90.2	61.4/99.0<br>3.1/79.1	38.5/98.4<br>3.9/100	83.1/98.5<br>6.0/90.6	83.1/98.5<br>6.2/85.2	78.3/99.2<br>4.7/100
Ulysses:5	42.7/83.5<br>5.7/41.3	44.5/96.1<br>3.1/100	83.1/97.1<br>4.7/60.6	62.0/96.2<br>3.6/83.3	6.0/90.9<br>2.8/100	30.7/100<br>0.8/75.0	86.7/94.1<br>6.0/59.9	75.9/99.2<br>6.0/100	65.0/99.0<br>2.8/75.0	65.6/92.3<br>3.6/100	22.8/92.6<br>3.9/59.9	87.3/96.0<br>7.0/56.8	86.1/97.2<br>7.8/64.8	47.5/89.7<br>3.9/41.1	37.9/96.9<br>3.1/95.8	84.9/96.5<br>6.0/57.7	85.5/96.5<br>6.2/63.3	80.7/96.4<br>5.2/80.1
VariationHunter:8	33.7/94.9<br>5.9/34.4	21.0/97.2<br>0.8/50.0	31.9/98.1<br>3.1/32.7	29.5/98.0<br>3.6/68.4	2.4/100<br>0.3/25.0	6.6/100<br>0/0	34.3/98.2<br>3.4/28.2	29.5/98.0<br>3.4/75.0	26.5/97.7<br>1.8/48.3	27.7/97.8<br>2.3/52.5	3.6/100<br>2.6/25.3	33.7/98.2<br>3.6/24.9	33.1/98.2<br>3.6/31.1	32.5/93.1<br>3.6/24.9	16.2/96.4<br>3.6/100	32.5/98.1<br>3.4/26.7	32.5/98.1<br>3.6/30.3	31.3/96.2<br>1.8/37.6
Wham:3	4.8/100<br>1.8/39.3	40.9/91.8<br>0.5/50.0	77.7/97.7<br>3.1/66.8	54.2/96.7<br>1.5/87.5	4.8/100<br>0.8/50.0	28.9/100<br>0.3/25.0	78.9/97.7<br>3.4/64.1	70.4/99.1<br>3.4/100	63.8/99.0<br>2.6/87.5	56.0/93.0<br>1.5/100	21.0/97.2<br>2.3/68.8	80.1/97.7<br>3.9/62.2	80.1/98.5<br>3.9/72.5	8.4/100<br>1.8/59.5	33.1/98.2<br>2.1/100	79.5/97.7<br>3.4/64.4	79.5/97.7<br>3.9/69.2	74.0/99.1<br>3.1/64.6

Algorithm	MetaSV:3	OncoSNP-SEQ:3	PennCNV-Seq:3	Pindel:4	PRISM:3	RAPTR:4	readDepth:5	Sniffles:2	SoftSV:3	Sprites:3	SvABA:3	SVDetect:4	SVelter:3	SVfinder:8	SVseq2:8	TIDDIT:3	Ulysses:5	VariationHunter:8	Wham:3
1-2-3-SV:7	11.4/100<br>1.8/36.9	5.4/100<br>4.4/85.8	31.9/100<br>1.8/91.7	3.6/50.0<br>2.6/16.1	1.8/50.0<br>0.8/4.8	86.1/91.1<br>5.2/18.3	0.6/100<br>0.3/25.0	9.0/50.0<br>1.7/49.9	4.8/61.5<br>2.1/15.5	5.4/64.2<br>3.6/20.3	4.2/77.7<br>2.8/33.0	91.5/80.6<br>10.6/26.5	3.6/85.7<br>1.8/61.7	91.5/80.2<br>10.7/22.9	3.0/55.5<br>1.0/27.8	59.6/99.0<br>5.4/82.7	42.7/83.5<br>5.7/41.3	33.7/94.9<br>5.9/34.4	4.8/100<br>1.8/39.3
BICseq2:8	43.9/92.4<br>1.8/100	9.6/80.0<br>4.7/97.2	34.3/82.6<br>1.8/57.5	42.7/94.6<br>1.0/75.0	19.2/96.9<br>0.5/50.0	43.9/98.6<br>2.6/100	4.8/53.3<br>0.3/25.0	37.3/91.1<br>1.0/66.7	42.7/93.4<br>2.1/75.0	43.9/92.4<br>2.8/100	40.9/91.8<br>1.3/100	46.9/97.5<br>4.2/100	33.7/91.8<br>0.5/25.0	45.7/98.7<br>2.6/100	32.5/93.1<br>0.3/33.3	45.1/97.4<br>3.9/100	44.5/96.1<br>3.1/100	21.0/97.2<br>0.8/50.0	40.9/91.8<br>0.5/50.0
BreakDancer:3	80.7/97.8<br>2.8/59.2	10.2/100<br>3.4/100	59.6/98.0<br>1.0/50.0	78.3/97.0<br>2.8/31.6	37.3/96.8<br>1.8/51.7	79.5/97.0<br>3.9/38.9	5.4/90.0<br>0/0	74.0/96.8<br>1.7/100	78.3/97.7<br>2.8/47.2	81.3/97.1<br>4.6/53.9	79.5/97.0<br>5.2/48.5	83.7/96.5<br>4.9/43.9	62.0/96.2<br>2.3/100	83.1/84.6<br>5.2/38.9	67.4/99.1<br>1.7/72.2	80.1/97.7<br>4.6/100	83.1/97.1<br>4.7/60.6	31.9/98.1<br>3.1/32.7	77.7/97.7<br>3.1/66.8
CNVnator:3	57.8/96.9<br>3.6/100	9.6/88.8<br>5.4/70.0	49.3/93.1<br>3.1/61.3	53.0/95.6<br>2.8/87.5	24.6/97.6<br>1.3/87.5	72.8/97.6<br>5.5/83.9	4.2/87.5<br>0.3/25.0	51.2/94.4<br>1.3/100	55.4/95.8<br>3.9/91.7	57.2/95.9<br>3.3/80.4	52.4/95.6<br>2.6/83.3	79.5/96.3<br>6.2/88.1	40.9/95.7<br>2.3/93.8	77.7/96.2<br>4.9/70.8	48.7/97.5<br>1.7/100	62.0/96.2<br>4.4/95.0	62.0/96.2<br>3.6/83.3	29.5/98.0<br>3.6/68.4	54.2/96.7<br>1.5/87.5
Control-FREEC:3	5.4/100<br>0.5/50.0	2.4/100<br>2.0/100	6.0/90.9<br>0.5/50.0	5.4/100<br>0.5/25.0	4.2/100<br>0.3/25.0	6.0/90.9<br>1.8/100	2.4/100<br>0/0	6.6/100<br>0.3/33.3	6.0/100<br>1.0/100	5.4/100<br>1.8/100	5.4/100<br>1.0/75.0	7.2/92.3<br>2.6/100	1.2/100<br>1.3/100	6.6/84.6<br>2.6/82.5	3.0/100<br>0/0	6.6/100<br>2.6/100	6.0/90.9<br>2.8/100	2.4/100<br>0.3/25.0	4.8/100<br>0.8/50.0
CREST:5	25.9/97.7<br>0/0	3.0/83.3<br>0.5/50.0	19.8/100<br>0.8/75.0	29.5/100<br>0.3/25.0	12.0/100<br>0/0	27.1/100<br>0/0	1.2/100<br>0.3/25.0	27.1/100<br>0.7/66.7	30.1/100<br>0.5/50.0	30.7/100<br>0.5/50.0	28.9/100<br>0.3/25.0	30.7/100<br>1.0/100	21.6/100<br>0/0	30.1/100<br>0.8/75.0	25.9/100<br>0.3/33.3	27.7/100<br>0.8/75.0	30.7/100<br>0.8/75.0	6.6/100<br>0/0	28.9/100<br>0.3/25.0
DELLY:8	81.9/99.2<br>2.6/49.3	10.8/100<br>3.6/100	62.0/98.0<br>1.8/75.0	81.9/97.8<br>3.4/27.0	40.3/98.5<br>2.0/46.3	84.9/97.9<br>4.7/33.6	4.2/87.5<br>0/0	77.7/97.7<br>2.0/86.7	84.3/97.9<br>2.8/42.1	87.3/97.3<br>4.9/48.1	85.5/97.9<br>6.2/41.7	89.1/94.8<br>6.0/38.6	62.6/97.1<br>2.3/100	89.1/93.6<br>7.0/33.7	69.2/98.2<br>1.7/72.2	83.7/98.5<br>5.4/88.7	86.7/94.1<br>6.0/59.9	34.3/98.2<br>3.4/28.2	78.9/97.7<br>3.4/64.1
ERDS:3	74.0/99.1<br>2.6/100	10.2/94.4<br>4.9/100	60.8/96.1<br>2.1/87.5	71.6/99.1<br>2.6/75.0	34.3/100<br>1.8/100	74.0/99.1<br>5.2/100	3.6/100<br>0/0	68.0/99.1<br>1.0/66.7	74.6/100<br>3.6/100	75.9/99.2<br>4.9/100	72.2/99.1<br>4.9/100	78.9/98.4<br>6.5/100	59.0/98.9<br>3.1/100	77.7/99.2<br>6.2/100	62.6/100<br>1.7/100	75.9/99.2<br>6.2/100	75.9/99.2<br>6.0/100	29.5/98.0<br>3.4/75.0	70.4/99.1<br>3.4/100
FermiKit:5	62.6/98.1<br>1.8/83.3	5.4/90.0<br>1.8/75.0	48.7/98.7<br>1.0/50.0	61.4/99.0<br>1.5/44.2	27.7/100<br>1.0/70.8	63.2/99.0<br>1.8/45.8	2.4/100<br>0/0	61.4/99.0<br>0.3/33.3	63.8/100<br>2.0/66.3	65.0/99.0<br>2.3/66.7	63.8/99.0<br>2.8/62.9	65.6/99.0<br>2.8/50.7	50.0/98.8<br>2.3/100	65.0/99.0<br>3.1/64.2	59.6/100<br>1.7/100	63.2/99.0<br>3.1/100	65.0/99.0<br>2.8/75.0	26.5/97.7<br>1.8/48.3	63.8/99.0<br>2.6/87.5
forestSV:6	57.8/93.2<br>2.3/100	7.2/85.7<br>5.7/96.4	51.2/90.4<br>2.8/91.7	57.2/92.2<br>1.8/75.0	27.7/88.4<br>1.5/100	69.8/98.3<br>4.9/100	3.0/100<br>0/0	53.0/89.7<br>1.4/66.7	57.8/93.2<br>2.8/100	59.6/91.6<br>3.1/93.8	57.2/93.1<br>2.3/100	74.6/96.8<br>4.9/91.4	45.7/93.8<br>1.8/100	73.4/97.6<br>3.9/100	51.2/93.4<br>1.3/100	68.6/94.2<br>4.7/100	65.6/92.3<br>3.6/100	27.7/97.8<br>2.3/52.5	56.0/93.0<br>1.5/100
GASVPro:5	21.0/100<br>1.8/47.5	2.4/100<br>3.1/100	15.0/100<br>1.3/66.7	21.6/97.2<br>1.8/20.4	9.6/100<br>0.5/10.4	21.6/100<br>3.1/24.8	0/0<br>0/0	18.6/96.8<br>1.0/55.5	21.0/94.5<br>1.0/19.8	22.2/94.8<br>2.6/35.4	22.2/97.3<br>2.9/30.1	22.8/95.0<br>4.4/28.0	13.2/95.6<br>1.8/50.0	22.8/92.6<br>3.9/25.9	15.6/96.2<br>1.0/50.0	21.6/100<br>3.6/79.2	22.8/92.6<br>3.9/59.9	3.6/100<br>2.6/25.3	21.0/97.2<br>2.3/68.8
GenomeSTRiP:4	82.5/98.5<br>3.6/55.3	10.8/94.7<br>5.4/100	62.0/98.0<br>2.1/93.8	83.1/97.8<br>4.2/20.9	39.7/98.5<br>2.0/41.9	85.5/99.3<br>5.7/24.2	3.6/85.7<br>0/0	77.7/97.7<br>1.7/50.0	84.9/97.9<br>3.6/38.6	87.9/97.3<br>6.5/39.4	85.5/97.9<br>6.2/40.0	89.7/96.7<br>8.6/32.8	62.6/97.1<br>2.6/100	88.5/96.0<br>7.8/28.1	69.2/98.2<br>2.0/72.2	84.9/98.6<br>7.0/84.9	87.3/96.0<br>7.0/56.8	33.7/98.2<br>3.6/24.9	80.1/97.7<br>3.9/62.2
GRIDSS:6	82.5/99.2<br>3.1/53.6	10.8/100<br>4.6/100	62.6/98.1<br>2.3/100	82.5/97.8<br>4.4/31.0	39.7/98.5<br>2.3/44.0	84.3/99.2<br>5.2/34.4	4.2/87.5<br>0/0	77.1/98.4<br>1.7/53.3	84.3/98.5<br>4.9/54.0	87.3/97.9<br>6.7/52.3	84.3/97.9<br>5.7/46.4	89.1/97.3<br>8.6/46.2	62.6/97.1<br>3.1/100	87.9/97.3<br>7.8/42.3	69.2/99.1<br>2.4/72.2	83.7/98.5<br>7.2/90.2	86.1/97.2<br>7.8/64.8	33.1/98.2<br>3.6/31.1	80.1/98.5<br>3.9/72.5
Hydra-sv:8	16.2/96.4<br>1.3/36.8	4.8/100<br>2.5/86.7	32.5/98.1<br>1.0/66.7	7.8/59.0<br>2.3/16.2	4.2/58.3<br>1.3/24.2	84.9/78.5<br>5.7/19.0	0.6/100<br>0/0	12.6/48.8<br>1.3/31.7	8.4/53.8<br>2.3/29.7	9.6/69.5<br>3.3/32.2	8.4/82.3<br>2.6/30.6	89.7/82.9<br>5.9/26.1	5.4/69.2<br>1.0/41.7	90.3/21.8<br>8.8/15.5	7.8/72.2<br>0.7/22.2	61.4/99.0<br>3.1/79.1	47.5/89.7<br>3.9/41.1	32.5/93.1<br>3.6/24.9	8.4/100<br>1.8/59.5
iCopyDAV:3	36.7/98.3<br>2.6/93.8	12.0/95.2<br>4.9/68.7	30.1/89.2<br>1.3/47.5	34.9/96.6<br>2.3/63.8	15.0/100<br>1.3/66.7	37.3/93.9<br>4.9/79.3	0/0<br>0/0	31.9/92.9<br>1.0/55.5	35.5/98.3<br>3.1/90.8	36.1/96.7<br>4.2/100	34.9/98.3<br>2.3/100	40.3/97.1<br>4.7/87.3	27.7/97.8<br>2.0/100	38.5/91.4<br>3.6/65.3	25.3/97.6<br>1.3/100	38.5/98.4<br>3.9/100	37.9/96.9<br>3.1/95.8	16.2/96.4<br>3.6/100	33.1/98.2<br>2.1/100
Lumpy:4	82.5/99.2<br>2.8/51.8	10.8/100<br>4.4/100	60.8/98.0<br>2.1/93.8	80.7/97.8<br>3.6/26.1	38.5/98.4<br>2.0/46.0	83.1/99.2<br>5.1/32.8	4.2/87.5<br>0/0	75.9/97.6<br>1.7/83.3	81.9/97.8<br>3.1/38.5	84.9/97.2<br>5.2/44.4	83.1/97.8<br>6.2/42.4	86.7/97.2<br>7.0/38.4	62.0/97.1<br>2.3/100	86.1/96.6<br>7.0/33.8	68.6/98.2<br>2.4/77.7	83.1/98.5<br>6.0/90.6	84.9/96.5<br>6.0/57.7	32.5/98.1<br>3.4/26.7	79.5/97.7<br>3.4/64.4
Manta:3	81.9/98.5<br>3.1/54.3	10.8/94.7<br>4.4/100	60.8/98.0<br>1.8/75.0	81.3/97.8<br>4.4/30.4	38.5/98.4<br>2.3/45.5	83.7/99.2<br>4.4/31.7	4.2/87.5<br>0/0	75.9/97.6<br>1.7/70.0	82.5/97.8<br>4.1/47.3	85.5/97.2<br>6.2/53.0	84.3/97.9<br>5.7/42.4	87.3/97.3<br>7.5/40.9	62.0/97.1<br>2.6/100	86.1/96.6<br>6.5/37.1	68.0/98.2<br>2.4/77.7	83.1/98.5<br>6.2/85.2	85.5/96.5<br>6.2/63.3	32.5/98.1<br>3.6/30.3	79.5/97.7<br>3.9/69.2
Meerkat:6	75.9/98.4<br>1.8/83.3	9.6/88.8<br>2.8/100	57.2/98.9<br>1.8/100	78.3/96.2<br>2.0/35.7	35.5/89.3<br>0.8/33.3	77.7/94.8<br>2.8/40.3	3.6/100<br>0.3/25.0	72.2/89.5<br>1.0/83.3	80.1/93.0<br>3.3/53.8	82.5/93.8<br>3.9/49.6	77.1/99.2<br>2.8/55.6	83.7/88.5<br>5.2/51.6	59.6/99.0<br>2.8/86.7	83.1/89.6<br>4.9/51.7	65.0/95.5<br>1.3/61.1	78.3/99.2<br>4.7/100	80.7/96.4<br>5.2/80.1	31.3/96.2<br>1.8/37.6	74.0/99.1<br>3.1/64.6
MetaSV:3	83.7/98.5<br>4.4/57.7	10.2/94.4<br>2.8/95.0	58.4/98.9<br>0.8/75.0	77.7/98.4<br>3.1/51.6	35.5/100<br>1.5/77.1	80.7/98.5<br>3.1/47.4	3.0/100<br>0/0	70.4/98.3<br>1.3/100	78.3/99.2<br>2.5/74.2	78.9/98.4<br>3.1/70.0	77.1/98.4<br>2.3/52.5	83.1/98.5<br>3.9/65.0	59.0/98.9<br>1.5/100	81.9/99.2<br>3.1/51.8	65.0/100<br>1.7/83.3	77.7/98.4<br>2.8/91.7	79.5/98.5<br>2.3/81.7	31.9/98.1<br>2.3/72.9	74.6/99.2<br>1.8/93.8
OncoSNP-SEQ:3	10.2/94.4<br>2.8/95.0	37.3/95.3<br>9.3/40.9	25.3/91.3<br>1.8/68.8	31.3/96.2<br>1.3/66.7	12.6/100<br>0.8/75.0	33.1/96.4<br>3.3/100	0/0<br>0.3/25.0	28.9/96.0<br>1.0/66.7	30.7/98.0<br>2.3/100	31.9/96.3<br>2.3/75.0	31.3/96.2<br>2.0/100	34.3/96.6<br>4.4/86.7	22.8/97.4<br>1.8/100	33.1/98.2<br>4.1/95.0	22.2/100<br>1.0/66.7	33.1/96.4<br>3.9/100	33.7/96.5<br>3.6/95.8	13.8/95.8<br>2.6/64.2	30.7/98.0<br>1.3/100
PennCNV-Seq:3	58.4/98.9<br>0.8/75.0	25.3/91.3<br>1.8/68.8	74.6/89.2<br>7.8/31.6	58.4/97.0<br>1.0/75.0	28.9/96.0<br>1.0/75.0	69.2/98.2<br>3.6/95.0	4.2/87.5<br>0.5/50.0	56.0/96.8<br>2.0/100	60.8/98.0<br>3.1/100	61.4/96.2<br>2.3/87.5	57.2/96.9<br>1.8/100	74.0/96.8<br>4.6/78.7	48.1/97.5<br>1.5/100	73.4/97.6<br>3.6/95.0	50.0/97.6<br>0.7/66.7	63.8/96.3<br>4.4/100	64.4/96.3<br>3.3/95.0	26.5/95.6<br>1.3/33.3	56.6/96.9<br>0.3/25.0
Pindel:4	77.7/98.4<br>3.1/51.6	31.3/96.2<br>1.3/66.7	58.4/97.0<br>1.0/75.0	86.7/92.9<br>6.7/14.4	38.5/96.9<br>1.8/15.6	81.9/98.5<br>3.6/16.2	3.6/85.7<br>0/0	73.4/93.8<br>2.0/51.2	82.5/95.1<br>2.8/27.5	84.9/95.9<br>5.2/28.8	80.1/97.7<br>3.1/30.8	86.7/93.5<br>5.9/19.7	59.0/97.0<br>1.5/75.0	85.5/94.0<br>4.7/16.3	66.2/94.8<br>2.4/77.7	79.5/98.5<br>2.8/58.8	83.1/96.5<br>3.3/39.9	31.9/94.6<br>4.9/48.2	75.9/98.4<br>2.3/55.4
PRISM:3	35.5/100<br>1.5/77.1	12.6/100<br>0.8/75.0	28.9/96.0<br>1.0/75.0	38.5/96.9<br>1.8/15.6	40.9/88.3<br>2.8/22.4	39.7/95.6<br>2.0/26.2	1.8/75.0<br>0/0	34.3/90.4<br>1.0/38.9	38.5/88.8<br>1.8/34.0	39.7/92.9<br>2.3/32.7	39.7/98.5<br>2.0/48.9	40.9/88.3<br>2.0/20.0	27.1/97.8<br>0.8/62.5	40.9/88.3<br>2.3/25.4	31.9/92.9<br>1.0/55.5	37.3/98.4<br>1.8/85.4	39.1/95.5<br>1.8/48.2	15.0/96.1<br>2.1/21.8	33.1/98.2<br>1.3/58.3
RAPTR:4	80.7/98.5<br>3.1/47.4	33.1/96.4<br>3.3/100	69.2/98.2<br>3.6/95.0	81.9/98.5<br>3.6/16.2	39.7/95.6<br>2.0/26.2	88.5/68.6<br>15.3/10.3	1.2/100<br>0/0	10.8/72.0<br>0.7/18.5	6.6/73.3<br>1.3/16.0	6.6/68.7<br>3.9/19.2	4.8/88.8<br>1.8/22.5	88.5/87.6<br>9.6/20.5	5.4/75.0<br>1.3/37.5	87.3/74.2<br>9.6/12.3	6.0/83.3<br>1.0/57.8	72.8/98.3<br>4.4/75.3	52.4/94.5<br>4.1/45.8	33.1/96.4<br>5.7/26.4	6.0/100<br>1.3/27.5
readDepth:5	3.0/100<br>0/0	0/0<br>0.3/25.0	4.2/87.5<br>0.5/50.0	3.6/85.7<br>0/0	1.8/75.0<br>0/0	1.2/100<br>0/0	36.7/95.3<br>2.3/93.8	34.3/98.2<br>0.3/33.3	34.3/98.2<br>0.5/50.0	35.5/98.3<br>0.5/25.0	34.9/98.3<br>0.8/50.0	35.5/98.3<br>1.5/100	28.9/97.9<br>0/0	35.5/98.3<br>1.5/100	33.7/98.2<br>0/0	34.9/98.3<br>1.8/100	34.9/98.3<br>1.8/100	13.2/100<br>0/0	33.7/98.2<br>0/0
Sniffles:2	70.4/98.3<br>1.3/100	28.9/96.0<br>1.0/66.7	56.0/96.8<br>2.0/100	73.4/93.8<br>2.0/51.2	34.3/90.4<br>1.0/38.9	10.8/72.0<br>0.7/18.5	34.3/98.2<br>0.3/33.3	84.3/53.4<br>4.5/26.1	75.3/86.2<br>2.4/53.7	78.9/89.7<br>2.4/45.4	76.5/98.4<br>1.7/88.9	83.1/75.8<br>3.4/37.6	57.8/97.9<br>1.0/66.7	82.5/74.4<br>3.1/27.4	63.2/91.3<br>0.3/33.3	76.5/97.6<br>2.4/91.7	78.3/94.8<br>2.4/80.5	31.3/96.2<br>3.4/52.6	70.4/96.6<br>0.7/66.7
SoftSV:3	78.3/99.2<br>2.5/74.2	30.7/98.0<br>2.3/100	60.8/98.0<br>3.1/100	82.5/95.1<br>2.8/27.5	38.5/88.8<br>1.8/34.0	6.6/73.3<br>1.3/16.0	34.3/98.2<br>0.5/50.0	75.3/86.2<br>2.4/53.7	89.1/90.7<br>6.7/36.7	87.3/94.7<br>5.4/46.5	81.3/99.2<br>2.8/44.4	89.1/91.9<br>5.9/40.3	61.4/99.0<br>2.3/100	87.3/91.7<br>5.2/38.1	69.8/92.8<br>2.0/66.7	80.1/99.2<br>4.7/100	84.9/95.9<br>5.2/71.4	33.7/96.5<br>3.1/43.4	78.3/98.4<br>2.0/58.3
Sprites:3	78.9/98.4<br>3.1/70.0	31.9/96.3<br>2.3/75.0	61.4/96.2<br>2.3/87.5	84.9/95.9<br>5.2/28.8	39.7/92.9<br>2.3/32.7	6.6/68.7<br>3.9/19.2	35.5/98.3<br>0.5/25.0	78.9/89.7<br>2.4/45.4	87.3/94.7<br>5.4/46.5	92.1/90.5<br>10.6/18.8	84.3/97.9<br>4.4/47.1	92.1/91.0<br>9.1/31.0	61.4/97.1<br>2.1/100	90.9/90.9<br>7.2/31.3	71.0/94.4<br>2.0/66.7	83.1/98.5<br>5.4/85.6	87.9/94.1<br>6.8/59.3	33.7/96.5<br>5.2/30.3	80.1/97.7<br>3.4/64.1
SvABA:3	77.1/98.4<br>2.3/52.5	31.3/96.2<br>2.0/100	57.2/96.9<br>1.8/100	80.1/97.7<br>3.1/30.8	39.7/98.5<br>2.0/48.9	4.8/88.8<br>1.8/22.5	34.9/98.3<br>0.8/50.0	76.5/98.4<br>1.7/88.9	81.3/99.2<br>2.8/44.4	84.3/97.9<br>4.4/47.1	86.1/97.9<br>6.2/39.0	86.1/97.9<br>5.2/39.7	60.8/97.1<br>2.3/100	85.5/97.9<br>6.2/40.6	68.6/99.1<br>1.7/72.2	81.3/98.5<br>5.1/92.9	84.3/97.9<br>5.2/63.2	32.5/98.1<br>3.4/35.5	76.5/98.4<br>3.1/61.3
SVDetect:4	83.1/98.5<br>3.9/65.0	34.3/96.6<br>4.4/86.7	74.0/96.8<br>4.6/78.7	86.7/93.5<br>5.9/19.7	40.9/88.3<br>2.0/20.0	88.5/87.6<br>9.6/20.5	35.5/98.3<br>1.5/100	83.1/75.8<br>3.4/37.6	89.1/91.9<br>5.9/40.3	92.1/91.0<br>9.1/31.0	86.1/97.9<br>5.2/39.7	96.9/65.4<br>18.0/16.6	10.2/94.4<br>2.3/87.5	93.3/76.2<br>12.5/22.9	10.8/75.0<br>1.7/66.7	71.6/97.5<br>7.0/79.5	58.4/85.8<br>7.8/53.5	34.3/93.4<br>7.8/29.1	11.4/100<br>2.6/64.6
SVelter:3	59.0/98.9<br>1.5/100	22.8/97.4<br>1.8/100	48.1/97.5<br>1.5/100	59.0/97.0<br>1.5/75.0	27.1/97.8<br>0.8/62.5	5.4/75.0<br>1.3/37.5	28.9/97.9<br>0/0	57.8/97.9<br>1.0/66.7	61.4/99.0<br>2.3/100	61.4/97.1<br>2.1/100	60.8/97.1<br>2.3/100	10.2/94.4<br>2.3/87.5	63.2/88.9<br>3.3/53.7	62.6/94.5<br>3.1/91.7	54.8/98.9<br>1.7/100	62.0/98.0<br>3.1/100	61.4/97.1<br>3.1/100	24.6/97.6<br>1.5/75.0	59.6/98.0<br>2.1/87.5
SVfinder:8	81.9/99.2<br>3.1/51.8	33.1/98.2<br>4.1/95.0	73.4/97.6<br>3.6/95.0	85.5/94.0<br>4.7/16.3	40.9/88.3<br>2.3/25.4	87.3/74.2<br>9.6/12.3	35.5/98.3<br>1.5/100	82.5/74.4<br>3.1/27.4	87.3/91.7<br>5.2/38.1	90.9/90.9<br>7.2/31.3	85.5/97.9<br>6.2/40.6	93.3/76.2<br>12.5/22.9	62.6/94.5<br>3.1/91.7	92.7/20.3<br>17.4/11.0	6.6/68.7<br>1.3/27.8	74.0/97.6<br>5.7/78.4	54.2/81.0<br>7.5/50.2	34.3/93.4<br>7.0/26.3	6.0/90.9<br>2.1/41.7
SVseq2:8	65.0/100<br>1.7/83.3	22.2/100<br>1.0/66.7	50.0/97.6<br>0.7/66.7	66.2/94.8<br>2.4/77.7	31.9/92.9<br>1.0/55.5	6.0/83.3<br>1.0/57.8	33.7/98.2<br>0/0	63.2/91.3<br>0.3/33.3	69.8/92.8<br>2.0/66.7	71.0/94.4<br>2.0/66.7	68.6/99.1<br>1.7/72.2	10.8/75.0<br>1.7/66.7	54.8/98.9<br>1.7/100	6.6/68.7<br>1.3/27.8	71.0/92.9<br>2.4/66.7	66.8/99.1<br>1.7/100	71.0/95.9<br>1.7/66.7	27.7/95.8<br>1.3/61.1	68.0/98.2<br>1.7/72.2
TIDDIT:3	77.7/98.4<br>2.8/91.7	33.1/96.4<br>3.9/100	63.8/96.3<br>4.4/100	79.5/98.5<br>2.8/58.8	37.3/98.4<br>1.8/85.4	72.8/98.3<br>4.4/75.3	34.9/98.3<br>1.8/100	76.5/97.6<br>2.4/91.7	80.1/99.2<br>4.7/100	83.1/98.5<br>5.4/85.6	81.3/98.5<br>5.1/92.9	71.6/97.5<br>7.0/79.5	62.0/98.0<br>3.1/100	74.0/97.6<br>5.7/78.4	66.8/99.1<br>1.7/100	84.9/97.9<br>8.8/82.7	83.1/98.5<br>7.3/94.4	32.5/98.1<br>3.1/61.7	77.7/98.4<br>3.4/100
Ulysses:5	79.5/98.5<br>2.3/81.7	33.7/96.5<br>3.6/95.8	64.4/96.3<br>3.3/95.0	83.1/96.5<br>3.3/39.9	39.1/95.5<br>1.8/48.2	52.4/94.5<br>4.1/45.8	34.9/98.3<br>1.8/100	78.3/94.8<br>2.4/80.5	84.9/95.9<br>5.2/71.4	87.9/94.1<br>6.8/59.3	84.3/97.9<br>5.2/63.2	58.4/85.8<br>7.8/53.5	61.4/97.1<br>3.1/100	54.2/81.0<br>7.5/50.2	71.0/95.9<br>1.7/66.7	83.1/98.5<br>7.3/94.4	89.7/87.6<br>9.6/51.7	33.1/98.2<br>3.9/42.1	80.1/97.7<br>3.9/76.3
VariationHunter:8	31.9/98.1<br>2.3/72.9	13.8/95.8<br>2.6/64.2	26.5/95.6<br>1.3/33.3	31.9/94.6<br>4.9/48.2	15.0/96.1<br>2.1/21.8	33.1/96.4<br>5.7/26.4	13.2/100<br>0/0	31.3/96.2<br>3.4/52.6	33.7/96.5<br>3.1/43.4	33.7/96.5<br>5.2/30.3	32.5/98.1<br>3.4/35.5	34.3/93.4<br>7.8/29.1	24.6/97.6<br>1.5/75.0	34.3/93.4<br>7.0/26.3	27.7/95.8<br>1.3/61.1	32.5/98.1<br>3.1/61.7	33.1/98.2<br>3.9/42.1	34.3/78.0<br>13.8/14.1	24.0/95.2<br>1.5/44.2
Wham:3	74.6/99.2<br>1.8/93.8	30.7/98.0<br>1.3/100	56.6/96.9<br>0.3/25.0	75.9/98.4<br>2.3/55.4	33.1/98.2<br>1.3/58.3	6.0/100<br>1.3/27.5	33.7/98.2<br>0/0	70.4/96.6<br>0.7/66.7	78.3/98.4<br>2.0/58.3	80.1/97.7<br>3.4/64.1	76.5/98.4<br>3.1/61.3	11.4/100<br>2.6/64.6	59.6/98.0<br>2.1/87.5	6.0/90.9<br>2.1/41.7	68.0/98.2<br>1.7/72.2	77.7/98.4<br>3.4/100	80.1/97.7<br>3.9/76.3	24.0/95.2<br>1.5/44.2	80.1/97.7<br>3.9/60.4

Figure S18. Recall and precision of SVs commonly called between a pair of SV detection algorithms for the DEL-L category. DELs in the large size-range (100 Kb–1 Mb), called using the indicated algorithms, were filtered with the minimum number of reads supporting the called SVs, indicated with the suffix number of the algorithm name. The DELs overlapping between the filtered SV sets from a pair of the indicated algorithms were selected, and the recall and precision of the selected DELs were determined. Recall and precision percentages are presented as in Figure S15. The data contained in the top 20th percentile of the combined precision scores for the simulated and real data are highlighted with a red background, and the next data contained in the top 21st–50th percentile of the combined precision scores are shown with a pale red background.

Algorithm	DELLY:3	GRIDSS:7	Lumpy:4	Manta:4	MATCHCLIP:7	Meerkat:8	MetaSV:6	Pindel:3	Sniffles:3	SoftSV:3	SvABA:3	Wham:6
DELLY:3	28.9/69.4<br>5.9/15.3	27.9/83.7<br>2.0/24.6	27.9/83.0<br>2.1/18.1	26.9/99.0<br>1.4/33.9	21.4/80.6<br>1.7/31.5	25.5/96.9<br>1.1/52.3	25.0/92.9<br>0.7/25.1	27.9/87.2<br>1.5/22.0	22.2/100<br>0.4/40.0	28.4/84.6<br>2.6/24.1	27.6/84.2<br>3.5/19.8	26.0/96.9<br>1.2/40.0
GRIDSS:7	27.9/83.7<br>2.0/24.6	31.3/51.3<br>15.5/51.6	29.5/75.1<br>6.6/47.1	29.6/99.0<br>6.3/63.9	22.8/73.6<br>7.8/70.9	25.2/96.8<br>0.9/58.0	27.4/84.8<br>5.9/65.7	30.6/81.8<br>7.6/65.2	22.2/100<br>0.3/51.5	30.8/80.2<br>8.9/63.8	29.8/83.3<br>8.6/52.2	29.0/95.5<br>8.5/74.7
Lumpy:4	27.9/83.0<br>2.1/18.1	29.5/75.1<br>6.6/47.1	29.8/70.9<br>7.7/36.5	28.2/99.0<br>4.6/46.9	22.8/79.2<br>5.2/61.6	25.0/97.8<br>1.0/57.1	26.3/88.9<br>4.6/58.5	29.2/88.5<br>5.6/55.6	22.5/100<br>0.2/30.9	29.5/90.0<br>5.0/46.0	28.4/87.5<br>5.7/37.2	27.7/96.2<br>5.4/65.1
Manta:4	26.9/99.0<br>1.4/33.9	29.6/99.0<br>6.3/63.9	28.2/99.0<br>4.6/46.9	30.1/98.2<br>7.4/54.9	22.0/100<br>4.5/73.7	24.7/98.9<br>0.9/63.1	26.6/98.9<br>4.1/58.7	29.3/99.0<br>5.2/61.0	22.0/100<br>0.2/44.0	29.8/99.0<br>5.4/65.4	28.8/99.0<br>4.8/50.9	27.9/99.0<br>5.9/77.6
MATCHCLIP:7	21.4/80.6<br>1.7/31.5	22.8/73.6<br>7.8/70.9	22.8/79.2<br>5.2/61.6	22.0/100<br>4.5/73.7	23.9/54.6<br>13.1/55.8	19.8/98.6<br>0.5/37.7	22.0/83.5<br>5.3/69.0	23.6/77.6<br>6.6/60.3	17.6/100<br>0.3/57.1	23.9/68.2<br>8.1/65.8	23.6/85.2<br>7.9/63.9	23.0/96.5<br>6.0/77.0
Meerkat:8	25.5/96.9<br>1.1/52.3	25.2/96.8<br>0.9/58.0	25.0/97.8<br>1.0/57.1	24.7/98.9<br>0.9/63.1	19.8/98.6<br>0.5/37.7	25.5/96.9<br>1.2/30.6	22.2/97.6<br>0.4/65.7	24.7/97.8<br>0.7/67.0	20.6/100<br>0.2/42.2	25.2/96.8<br>0.9/29.5	24.7/98.9<br>1.0/29.8	23.3/100<br>0.7/71.5
MetaSV:6	25.0/92.9<br>0.7/25.1	27.4/84.8<br>5.9/65.7	26.3/88.9<br>4.6/58.5	26.6/98.9<br>4.1/58.7	22.0/83.5<br>5.3/69.0	22.2/97.6<br>0.4/65.7	29.3/54.8<br>9.1/45.0	29.3/55.1<br>9.1/45.1	19.8/100<br>0.1/66.7	28.8/89.8<br>6.1/65.6	27.4/93.5<br>5.6/54.8	26.9/99.0<br>5.4/76.3
Pindel:3	27.9/87.2<br>1.5/22.0	30.6/81.8<br>7.6/65.2	29.2/88.5<br>5.6/55.6	29.3/99.0<br>5.2/61.0	23.6/77.6<br>6.6/60.3	24.7/97.8<br>0.7/67.0	29.3/55.1<br>9.1/45.1	32.3/33.0<br>15.1/29.4	22.0/100<br>0.3/66.7	31.9/84.2<br>8.1/51.6	30.3/88.8<br>7.4/51.0	29.3/98.1<br>6.5/78.1
Sniffles:3	22.2/100<br>0.4/40.0	22.2/100<br>0.3/51.5	22.5/100<br>0.2/30.9	22.0/100<br>0.2/44.0	17.6/100<br>0.3/57.1	20.6/100<br>0.2/42.2	19.8/100<br>0.1/66.7	22.0/100<br>0.3/66.7	24.4/96.8<br>2.1/41.7	23.3/100<br>0.4/70.8	22.8/100<br>0.8/62.4	21.4/100<br>0.2/66.7
SoftSV:3	28.4/84.6<br>2.6/24.1	30.8/80.2<br>8.9/63.8	29.5/90.0<br>5.0/46.0	29.8/99.0<br>5.4/65.4	23.9/68.2<br>8.1/65.8	25.2/96.8<br>0.9/29.5	28.8/89.8<br>6.1/65.6	31.9/84.2<br>8.1/51.6	23.3/100<br>0.4/70.8	33.3/33.4<br>39.3/25.8	31.1/88.4<br>8.8/49.1	29.8/95.6<br>7.6/72.6
SvABA:3	27.6/84.2<br>3.5/19.8	29.8/83.3<br>8.6/52.2	28.4/87.5<br>5.7/37.2	28.8/99.0<br>4.8/50.9	23.6/85.2<br>7.9/63.9	24.7/98.9<br>1.0/29.8	27.4/93.5<br>5.6/54.8	30.3/88.8<br>7.4/51.0	22.8/100<br>0.8/62.4	31.1/88.4<br>8.8/49.1	31.7/71.3<br>18.6/36.4	28.5/95.4<br>5.6/73.6
Wham:6	26.0/96.9<br>1.2/40.0	29.0/95.5<br>8.5/74.7	27.7/96.2<br>5.4/65.1	27.9/99.0<br>5.9/77.6	23.0/96.5<br>6.0/77.0	23.3/100<br>0.7/71.5	26.9/99.0<br>5.4/76.3	29.3/98.1<br>6.5/78.1	21.4/100<br>0.2/66.7	29.8/95.6<br>7.6/72.6	28.5/95.4<br>5.6/73.6	30.1/86.7<br>11.7/65.4

Figure S19. Recall and precision of SVs commonly called between a pair of SV detection algorithms for the DUP-S category. DUPs in the short size-range (100 bp–1 Kb), called using the indicated algorithms, were filtered with the minimum number of reads supporting the called SVs, indicated with the suffix number of the algorithm name. The DUPs overlapping between the filtered SV sets from a pair of the indicated algorithms were selected, and the recall and precision of the selected DUPs were determined. Recall and precision percentages are presented as in Figure S15. The data contained in the top 20th percentile of the combined precision scores for the simulated and real data are highlighted with a red background, and the next data contained in the top 21st–50th percentile of the combined precision scores are shown with a pale red background.

Algorithm	BICseq2:8	CNVnator:4	DELLY:3	ERDS:3	forestSV:4	GRIDSS:6	iCopyDAV:3	Lumpy:7	Manta:4	Meerkat:8	MetaSV:6	PennCNV-Seq:3	Pindel:5	RAPTR:5	readDepth:6	Sniffles:3	SoftSV:3	SVDetect:6	TIDDIT:3	Ulysses:3	Wham:6
BICseq2:8	32.3/65.7<br>2.9/22.3	16.1/94.4<br>0.6/67.7	30.4/97.1<br>0.6/40.8	6.5/79.5<br>1.4/38.0	13.5/69.4<br>1.1/29.9	30.2/97.3<br>0.5/57.2	12.5/79.0<br>0.5/30.2	29.5/97.1<br>0.3/54.4	29.5/97.6<br>0.3/69.4	29.3/98.0<br>0.2/78.3	24.6/96.1<br>0.6/63.6	10.0/80.7<br>0.6/25.4	26.2/97.6<br>0.3/50.3	2.1/72.3<br>0/4.2	17.2/81.0<br>1.0/43.2	24.4/96.9<br>0.1/13.9	30.4/97.8<br>0.2/49.7	2.2/71.0<br>0.2/31.9	1.7/61.9<br>0.1/41.7	30.0/94.9<br>0.6/31.9	28.6/97.6<br>0.2/62.0
CNVnator:4	16.1/94.4<br>0.6/67.7	24.2/91.3<br>2.4/44.1	14.0/99.0<br>0.1/100	8.5/93.1<br>1.4/49.3	6.7/89.7<br>1.0/38.0	13.8/99.0<br>0/50.0	14.9/94.5<br>0.5/41.4	13.0/99.3<br>0/50.0	13.4/99.0<br>0/50.0	13.6/99.6<br>0/25.0	14.4/89.8<br>1.6/42.6	6.6/89.6<br>0.3/39.7	13.5/99.0<br>0/25.0	7.2/98.1<br>0/0	14.5/91.7<br>0.7/67.0	10.6/97.9<br>0/33.3	14.1/98.7<br>0/25.0	8.0/98.3<br>0.1/75.0	4.8/98.1<br>0.1/75.0	14.2/98.1<br>0.1/87.5	12.5/99.2<br>0/25.0
DELLY:3	30.4/97.1<br>0.6/40.8	14.0/99.0<br>0.1/100	40.8/98.2<br>1.8/12.7	7.5/95.4<br>0.5/37.9	15.6/95.5<br>0.3/39.9	39.4/98.6<br>1.0/18.8	13.9/97.1<br>0/5.3	37.1/98.7<br>0.9/18.0	37.7/99.2<br>0.9/19.1	37.7/99.1<br>0.4/37.3	28.5/99.3<br>0.4/22.8	13.3/97.6<br>0.1/22.2	30.9/99.2<br>0.6/14.8	1.3/80.5<br>0.2/7.8	18.3/95.7<br>0.2/39.3	30.4/99.2<br>0.1/7.4	40.0/98.9<br>0.5/22.6	0.9/68.9<br>0.5/12.9	1.6/82.2<br>0.2/15.6	38.9/98.3<br>1.7/12.9	35.7/99.4<br>0.3/24.8
ERDS:3	6.5/79.5<br>1.4/38.0	8.5/93.1<br>1.4/49.3	7.5/95.4<br>0.5/37.9	10.0/19.5<br>10.3/3.3	3.7/38.0<br>2.8/20.6	5.4/99.1<br>0.3/59.0	6.7/84.7<br>0.6/38.0	5.0/99.1<br>0.2/53.4	5.1/99.1<br>0.2/49.2	5.3/98.3<br>0.1/52.5	5.5/87.7<br>1.4/47.3	3.0/73.9<br>1.4/16.4	5.3/98.3<br>0.3/45.2	3.0/89.4<br>0.1/25.6	6.1/85.5<br>1.4/59.1	4.8/96.4<br>0.1/66.7	5.5/98.4<br>0.1/46.1	3.3/91.4<br>0.3/40.1	2.3/78.4<br>0.3/48.3	5.8/94.8<br>0.5/42.4	4.9/99.0<br>0.1/54.1
forestSV:4	13.5/69.4<br>1.1/29.9	6.7/89.7<br>1.0/38.0	15.6/95.5<br>0.3/39.9	3.7/38.0<br>2.8/20.6	20.2/5.3<br>15.1/9.8	20.4/94.7<br>0.3/60.2	10.1/67.7<br>0.7/30.5	19.3/94.9<br>0.2/51.8	19.5/95.3<br>0.2/51.8	19.8/95.6<br>0.1/62.5	15.7/90.6<br>1.4/39.8	10.1/59.9<br>2.4/12.0	16.4/95.5<br>0.3/37.5	7.2/66.3<br>0.1/22.8	10.7/72.4<br>1.0/42.2	17.1/94.5<br>0.1/69.0	21.0/95.4<br>0.1/51.3	8.2/69.5<br>0.3/42.9	5.7/57.2<br>0.4/48.4	19.2/81.5<br>0.6/39.1	18.7/95.8<br>0.1/52.5
GRIDSS:6	30.2/97.3<br>0.5/57.2	13.8/99.0<br>0/50.0	39.4/98.6<br>1.0/18.8	5.4/99.1<br>0.3/59.0	20.4/94.7<br>0.3/60.2	39.4/98.7<br>1.1/17.0	13.7/97.4<br>0.1/45.8	37.1/98.7<br>0.9/20.8	37.6/99.4<br>0.8/21.6	36.9/99.1<br>0.3/41.8	28.1/99.6<br>0.3/32.7	12.8/98.9<br>0.1/70.8	30.6/99.5<br>0.4/27.9	1.0/82.1<br>0.1/6.0	18.0/96.1<br>0.2/66.4	30.0/99.4<br>0.1/47.6	38.9/98.9<br>0.5/26.5	0.7/84.2<br>0.3/13.2	1.4/86.8<br>0.1/10.4	37.4/99.0<br>0.9/17.6	35.7/99.4<br>0.3/27.4
iCopyDAV:3	12.5/79.0<br>0.5/30.2	14.9/94.5<br>0.5/41.4	13.9/97.1<br>0/5.3	6.7/84.7<br>0.6/38.0	10.1/67.7<br>0.7/30.5	13.7/97.4<br>0.1/45.8	20.4/56.8<br>2.5/19.2	14.3/98.4<br>0/37.5	14.4/99.0<br>0/50.0	14.4/98.1<br>0/0	14.0/94.2<br>0.6/49.5	8.4/86.5<br>0.7/29.2	14.3/98.1<br>0.1/5.3	9.2/91.8<br>0/0	13.3/83.0<br>0.6/39.5	11.6/98.4<br>0/0	14.8/97.0<br>0/0	10.0/93.2<br>0.1/15.0	7.4/92.6<br>0.1/23.3	15.0/94.5<br>0.1/11.0	13.7/99.6<br>0/0
Lumpy:7	29.5/97.1<br>0.3/54.4	13.0/99.3<br>0/50.0	37.1/98.7<br>0.9/18.0	5.0/99.1<br>0.2/53.4	19.3/94.9<br>0.2/51.8	37.1/98.7<br>0.9/20.8	14.3/98.4<br>0/37.5	37.1/98.9<br>0.9/17.4	36.7/99.3<br>0.8/22.1	35.1/99.2<br>0.3/41.1	26.8/99.6<br>0.3/33.8	12.0/98.8<br>0.1/66.7	29.1/99.5<br>0.4/27.4	0.9/86.9<br>0.1/9.3	17.3/96.0<br>0.1/80.4	29.2/99.3<br>0.1/44.4	36.8/99.0<br>0.5/29.2	0.4/76.9<br>0.2/13.7	0.9/81.4<br>0.1/14.9	35.4/98.9<br>0.8/16.9	35.3/99.7<br>0.3/26.1
Manta:4	29.5/97.6<br>0.3/69.4	13.4/99.0<br>0/50.0	37.7/99.2<br>0.9/19.1	5.1/99.1<br>0.2/49.2	19.5/95.3<br>0.2/51.8	37.6/99.4<br>0.8/21.6	14.4/99.0<br>0/50.0	36.7/99.3<br>0.8/22.1	37.8/99.4<br>0.9/19.0	35.6/99.4<br>0.3/41.7	27.2/99.8<br>0.3/33.1	12.1/98.8<br>0.1/43.3	29.6/99.6<br>0.4/29.9	0.7/94.4<br>0.1/7.4	17.6/96.7<br>0.1/67.9	29.4/99.5<br>0.1/50.0	37.5/99.5<br>0.5/29.5	0.3/77.7<br>0.2/12.1	1.2/93.1<br>0.1/20.6	35.9/99.4<br>0.8/18.0	35.3/99.8<br>0.3/31.1
Meerkat:8	29.3/98.0<br>0.2/78.3	13.6/99.6<br>0/25.0	37.7/99.1<br>0.4/37.3	5.3/98.3<br>0.1/52.5	19.8/95.6<br>0.1/62.5	36.9/99.1<br>0.3/41.8	14.4/98.1<br>0/0	35.1/99.2<br>0.3/41.1	35.6/99.4<br>0.3/41.7	38.3/96.4<br>0.4/37.2	27.4/98.5<br>0.2/67.9	12.6/98.5<br>0.1/43.8	29.5/98.0<br>0.2/59.7	1.1/76.4<br>0/19.6	17.6/96.2<br>0.1/45.8	28.5/99.0<br>0.1/55.5	37.8/97.3<br>0.3/47.5	0.8/69.2<br>0.1/33.2	1.6/87.8<br>0/62.5	36.4/96.4<br>0.3/35.9	34.1/99.6<br>0.3/55.9
MetaSV:6	24.6/96.1<br>0.6/63.6	14.4/89.8<br>1.6/42.6	28.5/99.3<br>0.4/22.8	5.5/87.7<br>1.4/47.3	15.7/90.6<br>1.4/39.8	28.1/99.6<br>0.3/32.7	14.0/94.2<br>0.6/49.5	26.8/99.6<br>0.3/33.8	27.2/99.8<br>0.3/33.1	27.4/98.5<br>0.2/67.9	30.0/89.9<br>3.3/33.8	9.3/95.4<br>0.4/39.6	28.0/96.8<br>0.7/25.4	0.9/86.9<br>0.1/15.8	16.4/94.0<br>0.6/59.4	21.4/99.1<br>0.1/33.3	28.2/98.1<br>0.2/46.2	0.4/84.6<br>0.2/29.3	0.3/88.8<br>0.1/38.3	28.0/97.7<br>0.4/25.5	25.6/99.8<br>0.2/47.6
PennCNV-Seq:3	10.0/80.7<br>0.6/25.4	6.6/89.6<br>0.3/39.7	13.3/97.6<br>0.1/22.2	3.0/73.9<br>1.4/16.4	10.1/59.9<br>2.4/12.0	12.8/98.9<br>0.1/70.8	8.4/86.5<br>0.7/29.2	12.0/98.8<br>0.1/66.7	12.1/98.8<br>0.1/43.3	12.6/98.5<br>0.1/43.8	9.3/95.4<br>0.4/39.6	32.4/60.7<br>9.8/6.6	26.9/98.3<br>0.2/37.8	24.1/88.4<br>0.2/30.4	21.6/84.7<br>1.0/35.7	25.7/98.9<br>0/16.7	32.9/98.6<br>0.1/56.3	24.7/88.6<br>0.4/48.3	17.6/82.7<br>0.6/50.8	30.7/89.3<br>0.7/39.4	29.1/99.0<br>0.1/72.9
Pindel:5	26.2/97.6<br>0.3/50.3	13.5/99.0<br>0/25.0	30.9/99.2<br>0.6/14.8	5.3/98.3<br>0.3/45.2	16.4/95.5<br>0.3/37.5	30.6/99.5<br>0.4/27.9	14.3/98.1<br>0.1/5.3	29.1/99.5<br>0.4/27.4	29.6/99.6<br>0.4/29.9	29.5/98.0<br>0.2/59.7	28.0/96.8<br>0.7/25.4	26.9/98.3<br>0.2/37.8	31.3/95.8<br>1.2/13.6	0.8/86.3<br>0.1/5.9	17.7/97.2<br>0.1/33.3	23.5/99.2<br>0.1/5.1	31.1/97.8<br>0.3/38.1	0.4/76.9<br>0.3/11.3	0.5/80.0<br>0.1/22.8	30.8/97.8<br>0.6/11.3	28.3/99.8<br>0.2/41.3
RAPTR:5	2.1/72.3<br>0/4.2	7.2/98.1<br>0/0	1.3/80.5<br>0.2/7.8	3.0/89.4<br>0.1/25.6	7.2/66.3<br>0.1/22.8	1.0/82.1<br>0.1/6.0	9.2/91.8<br>0/0	0.9/86.9<br>0.1/9.3	0.7/94.4<br>0.1/7.4	1.1/76.4<br>0/19.6	0.9/86.9<br>0.1/15.8	24.1/88.4<br>0.2/30.4	0.8/86.3<br>0.1/5.9	31.8/84.0<br>1.1/8.9	20.5/98.0<br>0.1/49.0	26.8/98.9<br>0/16.6	34.6/97.5<br>0.1/15.8	30.8/86.7<br>0.2/9.9	26.7/86.4<br>0.3/17.0	31.7/84.8<br>0.6/9.2	30.7/99.7<br>0.1/10.2
readDepth:6	17.2/81.0<br>1.0/43.2	14.5/91.7<br>0.7/67.0	18.3/95.7<br>0.2/39.3	6.1/85.5<br>1.4/59.1	10.7/72.4<br>1.0/42.2	18.0/96.1<br>0.2/66.4	13.3/83.0<br>0.6/39.5	17.3/96.0<br>0.1/80.4	17.6/96.7<br>0.1/67.9	17.6/96.2<br>0.1/45.8	16.4/94.0<br>0.6/59.4	21.6/84.7<br>1.0/35.7	17.7/97.2<br>0.1/33.3	20.5/98.0<br>0.1/49.0	23.2/52.1<br>2.9/28.0	9.9/96.0<br>0/22.2	12.3/95.7<br>0.1/93.8	7.4/91.6<br>0.1/41.7	5.7/84.1<br>0.2/62.2	12.3/93.4<br>0.2/41.4	11.3/96.8<br>0.1/75.0
Sniffles:3	24.4/96.9<br>0.1/13.9	10.6/97.9<br>0/33.3	30.4/99.2<br>0.1/7.4	4.8/96.4<br>0.1/66.7	17.1/94.5<br>0.1/69.0	30.0/99.4<br>0.1/47.6	11.6/98.4<br>0/0	29.2/99.3<br>0.1/44.4	29.4/99.5<br>0.1/50.0	28.5/99.0<br>0.1/55.5	21.4/99.1<br>0.1/33.3	25.7/98.9<br>0/16.7	23.5/99.2<br>0.1/5.1	26.8/98.9<br>0/16.6	9.9/96.0<br>0/22.2	31.2/93.3<br>0.3/18.3	30.4/99.2<br>0.1/24.4	0.9/76.9<br>0/6.7	1.1/86.2<br>0/0	29.4/98.9<br>0.1/6.0	28.2/99.6<br>0/8.3
SoftSV:3	30.4/97.8<br>0.2/49.7	14.1/98.7<br>0/25.0	40.0/98.9<br>0.5/22.6	5.5/98.4<br>0.1/46.1	21.0/95.4<br>0.1/51.3	38.9/98.9<br>0.5/26.5	14.8/97.0<br>0/0	36.8/99.0<br>0.5/29.2	37.5/99.5<br>0.5/29.5	37.8/97.3<br>0.3/47.5	28.2/98.1<br>0.2/46.2	32.9/98.6<br>0.1/56.3	31.1/97.8<br>0.3/38.1	34.6/97.5<br>0.1/15.8	12.3/95.7<br>0.1/93.8	30.4/99.2<br>0.1/24.4	40.9/96.1<br>0.6/18.9	0.9/70.9<br>0.2/13.1	1.7/82.6<br>0/13.5	38.7/96.5<br>0.5/21.0	35.4/99.4<br>0.3/35.6
SVDetect:6	2.2/71.0<br>0.2/31.9	8.0/98.3<br>0.1/75.0	0.9/68.9<br>0.5/12.9	3.3/91.4<br>0.3/40.1	8.2/69.5<br>0.3/42.9	0.7/84.2<br>0.3/13.2	10.0/93.2<br>0.1/15.0	0.4/76.9<br>0.2/13.7	0.3/77.7<br>0.2/12.1	0.8/69.2<br>0.1/33.2	0.4/84.6<br>0.2/29.3	24.7/88.6<br>0.4/48.3	0.4/76.9<br>0.3/11.3	30.8/86.7<br>0.2/9.9	7.4/91.6<br>0.1/41.7	0.9/76.9<br>0/6.7	0.9/70.9<br>0.2/13.1	34.5/83.4<br>1.0/12.6	27.8/87.4<br>0.5/32.9	34.4/85.3<br>0.9/14.2	32.5/99.4<br>0.1/21.1
TIDDIT:3	1.7/61.9<br>0.1/41.7	4.8/98.1<br>0.1/75.0	1.6/82.2<br>0.2/15.6	2.3/78.4<br>0.3/48.3	5.7/57.2<br>0.4/48.4	1.4/86.8<br>0.1/10.4	7.4/92.6<br>0.1/23.3	0.9/81.4<br>0.1/14.9	1.2/93.1<br>0.1/20.6	1.6/87.8<br>0/62.5	0.3/88.8<br>0.1/38.3	17.6/82.7<br>0.6/50.8	0.5/80.0<br>0.1/22.8	26.7/86.4<br>0.3/17.0	5.7/84.1<br>0.2/62.2	1.1/86.2<br>0/0	1.7/82.6<br>0/13.5	27.8/87.4<br>0.5/32.9	28.8/78.4<br>1.0/14.6	29.9/87.0<br>0.8/26.9	30.2/99.8<br>0.2/39.9
Ulysses:3	30.0/94.9<br>0.6/31.9	14.2/98.1<br>0.1/87.5	38.9/98.3<br>1.7/12.9	5.8/94.8<br>0.5/42.4	19.2/81.5<br>0.6/39.1	37.4/99.0<br>0.9/17.6	15.0/94.5<br>0.1/11.0	35.4/98.9<br>0.8/16.9	35.9/99.4<br>0.8/18.0	36.4/96.4<br>0.3/35.9	28.0/97.7<br>0.4/25.5	30.7/89.3<br>0.7/39.4	30.8/97.8<br>0.6/11.3	31.7/84.8<br>0.6/9.2	12.3/93.4<br>0.2/41.4	29.4/98.9<br>0.1/6.0	38.7/96.5<br>0.5/21.0	34.4/85.3<br>0.9/14.2	29.9/87.0<br>0.8/26.9	35.7/82.9<br>2.3/8.9	33.9/99.6<br>0.2/21.4
Wham:6	28.6/97.6<br>0.2/62.0	12.5/99.2<br>0/25.0	35.7/99.4<br>0.3/24.8	4.9/99.0<br>0.1/54.1	18.7/95.8<br>0.1/52.5	35.7/99.4<br>0.3/27.4	13.7/99.6<br>0/0	35.3/99.7<br>0.3/26.1	35.3/99.8<br>0.3/31.1	34.1/99.6<br>0.3/55.9	25.6/99.8<br>0.2/47.6	29.1/99.0<br>0.1/72.9	28.3/99.8<br>0.2/41.3	30.7/99.7<br>0.1/10.2	11.3/96.8<br>0.1/75.0	28.2/99.6<br>0/8.3	35.4/99.4<br>0.3/35.6	32.5/99.4<br>0.1/21.1	30.2/99.8<br>0.2/39.9	33.9/99.6<br>0.2/21.4	35.7/99.4<br>0.3/22.9

Figure S20. Recall and precision of SVs commonly called between a pair of SV detection algorithms for the DUP-M category. DUPs in the middle size-range (1 Kb–100 Kb), called using the indicated algorithms, were filtered with the minimum number of reads supporting the called SVs, indicated with the suffix number of the algorithm name. The DUPs overlapping between the filtered SV sets from a pair of the indicated algorithms were selected, and the recall and precision of the selected DUPs were determined. Recall and precision percentages are presented as in Figure S15. The data contained in the top 20th percentile of the combined precision scores for the simulated and real data are highlighted with a red background, and the next data contained in the top 21st–50th percentile of the combined precision scores are shown with a pale red background.

Algorithm	AS-GENSENG:3	BICseq2:8	CNVnator:4	DELLY:3	ERDS:3	forestSV:6	GRIDSS:6	iCopyDAV:3	inGAP-sv:3	Lumpy:4	Manta:4	Meerkat:8	MetaSV:7	OncoSNP-SEQ:4	PennCNV-Seq:3	Pindel:4	RAPTR:3	readDepth:6	Sniffles:2	SoftSV:6	SVDetect:3	TIDDIT:3	Ulysses:3
AS-GENSENG:3	13.1/43.2<br>5.1/19.1	11.2/88.9<br>1.1/75.0	10.6/88.4<br>1.4/58.5	12.0/89.6<br>0.1/12.5	8.5/86.8<br>1.0/74.4	5.3/93.1<br>0.4/37.5	12.0/89.6<br>0/0	6.0/83.5<br>0.8/60.4	5.5/94.9<br>0.1/25.0	11.8/90.1<br>0/0	11.8/90.1<br>0/0	11.5/89.9<br>0.3/50.0	10.5/88.3<br>1.8/63.1	1.8/65.5<br>0.1/12.5	5.2/89.8<br>0/0	11.4/89.8<br>0.8/75.0	6.3/87.6<br>0.6/64.6	8.5/82.6<br>0.9/55.5	11.4/82.1<br>0.5/55.5	11.9/89.5<br>0.3/37.5	6.6/87.0<br>0.6/51.7	4.1/87.5<br>0.5/56.3	12.1/89.0<br>0.8/53.5
BICseq2:8	11.2/88.9<br>1.1/75.0	36.8/84.9<br>4.4/48.7	25.1/92.7<br>2.7/73.0	35.9/97.0<br>0.1/25.0	23.1/94.3<br>1.9/68.3	13.1/96.3<br>1.4/65.8	35.7/97.0<br>0/0	13.7/86.8<br>1.5/76.3	14.1/95.3<br>0.5/62.5	34.9/97.5<br>0/0	35.1/97.5<br>0/0	34.5/96.9<br>0.4/75.0	30.2/95.9<br>2.3/100	5.0/80.9<br>0/0	11.1/87.5<br>0.3/25.0	33.9/96.8<br>0.6/100	4.7/77.4<br>0.1/25.0	22.9/88.5<br>0.9/39.6	32.6/97.0<br>0.5/100	35.6/97.0<br>0.5/100	5.2/79.1<br>0.3/25.0	3.3/69.3<br>0.1/25.0	36.0/95.0<br>0.8/91.7
CNVnator:4	10.6/88.4<br>1.4/58.5	25.1/92.7<br>2.7/73.0	41.7/91.1<br>8.2/49.3	26.5/98.5<br>0.1/8.3	31.5/94.6<br>5.0/59.5	9.6/97.0<br>1.8/75.0	26.0/98.5<br>0/0	20.9/93.7<br>1.6/70.8	10.4/98.1<br>0.1/25.0	25.5/98.8<br>0/0	25.3/98.8<br>0/0	25.7/98.8<br>0/0	25.2/94.4<br>6.1/59.2	5.3/90.0<br>0/0	12.5/95.4<br>0.1/25.0	24.8/98.4<br>0.1/25.0	14.8/96.1<br>0.1/12.5	25.6/94.1<br>1.3/49.6	24.7/98.0<br>0/33.3	26.7/98.5<br>0/0	16.4/96.5<br>0.1/12.5	10.4/93.7<br>0.1/8.3	27.2/97.5<br>0.1/8.3
DELLY:3	12.0/89.6<br>0.1/12.5	35.9/97.0<br>0.1/25.0	26.5/98.5<br>0.1/8.3	44.9/98.0<br>2.4/7.0	26.3/96.0<br>0.6/100	14.2/97.9<br>0.6/100	43.8/98.6<br>0.8/7.0	15.5/90.2<br>0.6/91.7	15.4/97.5<br>0.1/25.0	42.2/99.0<br>1.0/6.1	42.3/99.0<br>0.4/3.9	42.2/99.7<br>0/0	36.5/99.4<br>0.9/23.4	5.5/82.3<br>0/0	13.2/97.8<br>0/25.0	41.8/99.5<br>1.3/10.9	1.2/65.0<br>1.1/6.8	25.4/96.2<br>0/0	39.7/99.5<br>0/0	44.5/99.5<br>0.1/12.5	1.5/66.6<br>1.3/8.4	0.9/58.8<br>0.6/12.1	44.8/98.0<br>2.4/7.1
ERDS:3	8.5/86.8<br>1.0/74.4	23.1/94.3<br>1.9/68.3	31.5/94.6<br>5.0/59.5	26.3/96.0<br>0.6/100	35.1/87.9<br>7.8/50.8	9.2/97.8<br>1.6/67.9	21.8/97.7<br>0/0	18.3/93.4<br>1.8/68.7	9.2/98.9<br>0.1/25.0	21.2/97.7<br>0/0	21.1/97.7<br>0/0	21.6/97.7<br>0/0	21.0/93.8<br>4.2/79.1	5.6/86.5<br>0/0	10.7/94.7<br>0/0	21.5/97.7<br>0.5/87.5	13.1/97.0<br>0.6/87.5	22.5/94.5<br>0.8/42.5	20.3/96.2<br>0/33.3	22.4/97.8<br>0/0	13.9/97.2<br>0.5/62.5	9.4/95.0<br>0.5/75.0	23.0/97.8<br>0.6/75.0
forestSV:6	5.3/93.1<br>0.4/37.5	13.1/96.3<br>1.4/65.8	9.6/97.0<br>1.8/75.0	14.2/97.9<br>0.6/100	9.2/97.8<br>1.6/67.9	17.4/92.1<br>3.8/62.4	16.1/98.1<br>0/0	12.0/95.2<br>0.4/29.2	14.8/96.1<br>0.4/75.0	15.8/98.1<br>0/0	16.0/98.1<br>0/0	15.5/98.1<br>0/0	14.5/98.0<br>1.6/76.2	0.8/100<br>0/0	7.2/97.3<br>0.3/50.0	15.9/98.1<br>0/0	4.2/93.4<br>0.1/25.0	10.5/94.6<br>0.8/66.7	15.7/96.9<br>0/33.3	16.5/98.2<br>0/0	5.2/94.6<br>0/0	3.0/88.5<br>0/0	16.6/98.2<br>0.1/25.0
GRIDSS:6	12.0/89.6<br>0/0	35.7/97.0<br>0/0	26.0/98.5<br>0/0	43.8/98.6<br>0.8/7.0	21.8/97.7<br>0/0	16.1/98.1<br>0/0	43.9/98.2<br>0.8/6.8	15.5/90.2<br>0/0	15.4/97.5<br>0/0	42.3/99.0<br>0.6/7.8	42.4/99.0<br>0.4/7.0	41.3/99.7<br>0/0	35.7/99.4<br>0.3/11.3	5.4/82.0<br>0/0	12.9/97.7<br>0/0	41.0/99.5<br>0.5/9.3	1.0/73.3<br>0/0	25.1/96.2<br>0/0	39.6/99.7<br>0/0	43.6/99.5<br>0/0	1.4/78.9<br>0/0	0.9/66.6<br>0/0	43.8/98.8<br>0.6/6.2
iCopyDAV:3	6.0/83.5<br>0.8/60.4	13.7/86.8<br>1.5/76.3	20.9/93.7<br>1.6/70.8	15.5/90.2<br>0.6/91.7	18.3/93.4<br>1.8/68.7	12.0/95.2<br>0.4/29.2	15.5/90.2<br>0/0	34.6/85.9<br>3.8/67.1	11.1/95.7<br>0.3/25.0	30.3/97.4<br>0/0	30.1/97.4<br>0/0	29.7/96.7<br>0/0	28.0/96.2<br>2.4/87.3	10.2/92.7<br>0/0	19.9/89.7<br>0.3/25.0	29.0/96.6<br>0/0	23.2/96.6<br>0.1/25.0	24.9/92.2<br>0.5/45.8	28.3/96.6<br>0/0	31.0/97.2<br>0/0	25.0/96.5<br>0/0	19.9/95.7<br>0/0	31.3/96.9<br>0.1/25.0
inGAP-sv:3	5.5/94.9<br>0.1/25.0	14.1/95.3<br>0.5/62.5	10.4/98.1<br>0.1/25.0	15.4/97.5<br>0.1/25.0	9.2/98.9<br>0.1/25.0	14.8/96.1<br>0.4/75.0	15.4/97.5<br>0/0	11.1/95.7<br>0.3/25.0	16.3/90.7<br>1.9/66.4	15.5/96.9<br>0/0	15.6/96.9<br>0/0	15.3/96.8<br>0/0	14.3/97.3<br>0.5/66.7	0.2/100<br>0/0	5.7/92.0<br>0.5/50.0	15.5/96.9<br>0.1/25.0	4.0/89.1<br>0/0	9.6/92.3<br>0.1/12.5	15.4/96.2<br>0/0	16.2/97.0<br>0.1/25.0	4.5/90.1<br>0/0	1.2/72.2<br>0/0	16.1/97.0<br>0.1/12.5
Lumpy:4	11.8/90.1<br>0/0	34.9/97.5<br>0/0	25.5/98.8<br>0/0	42.2/99.0<br>1.0/6.1	21.2/97.7<br>0/0	15.8/98.1<br>0/0	42.3/99.0<br>0.6/7.8	30.3/97.4<br>0/0	15.5/96.9<br>0/0	42.4/98.8<br>1.0/6.1	42.0/98.8<br>0.4/4.5	40.1/99.7<br>0/0	34.5/99.4<br>0.6/23.8	5.1/81.2<br>0/0	12.5/98.4<br>0/0	39.6/99.5<br>0.8/11.9	1.4/83.3<br>0.4/4.5	24.3/96.4<br>0/0	38.7/99.7<br>0/0	42.1/99.5<br>0/0	1.4/78.9<br>0.9/12.5	0.8/60.0<br>0.1/3.1	42.4/98.8<br>1.0/6.1
Manta:4	11.8/90.1<br>0/0	35.1/97.5<br>0/0	25.3/98.8<br>0/0	42.3/99.0<br>0.4/3.9	21.1/97.7<br>0/0	16.0/98.1<br>0/0	42.4/99.0<br>0.4/7.0	30.1/97.4<br>0/0	15.6/96.9<br>0/0	42.0/98.8<br>0.4/4.5	42.4/98.8<br>0.5/5.3	40.2/99.7<br>0/0	34.8/99.4<br>0.3/14.6	5.1/81.2<br>0/0	12.5/98.4<br>0/0	39.8/99.5<br>0.3/6.8	0.8/75.0<br>0/0	23.9/96.4<br>0/0	38.5/99.7<br>0/0	42.2/99.5<br>0/0	1.1/75.0<br>0.3/6.9	0.6/58.3<br>0/0	42.3/99.0<br>0.4/4.0
Meerkat:8	11.5/89.9<br>0.3/50.0	34.5/96.9<br>0.4/75.0	25.7/98.8<br>0/0	42.2/99.7<br>0/0	21.6/97.7<br>0/0	15.5/98.1<br>0/0	41.3/99.7<br>0/0	29.7/96.7<br>0/0	15.3/96.8<br>0/0	40.1/99.7<br>0/0	40.2/99.7<br>0/0	43.3/98.8<br>0.5/41.7	35.2/98.6<br>0.4/62.5	5.2/81.5<br>0/0	12.5/97.6<br>0/0	40.0/98.7<br>0.4/58.3	1.2/72.2<br>0.1/12.5	24.4/96.0<br>0/0	38.2/98.7<br>0.5/66.7	42.8/99.0<br>0.4/62.5	1.3/70.0<br>0.1/8.3	0.9/71.4<br>0/0	43.1/99.0<br>0.4/31.3
MetaSV:7	10.5/88.3<br>1.8/63.1	30.2/95.9<br>2.3/100	25.2/94.4<br>6.1/59.2	36.5/99.4<br>0.9/23.4	21.0/93.8<br>4.2/79.1	14.5/98.0<br>1.6/76.2	35.7/99.4<br>0.3/11.3	28.0/96.2<br>2.4/87.3	14.3/97.3<br>0.5/66.7	34.5/99.4<br>0.6/23.8	34.8/99.4<br>0.3/14.6	35.2/98.6<br>0.4/62.5	38.9/94.2<br>7.2/39.5	5.3/91.5<br>0/0	11.2/99.1<br>0.1/25.0	34.9/98.5<br>1.0/18.2	2.7/87.5<br>0.5/16.1	21.5/94.3<br>1.1/58.3	33.4/97.9<br>0.2/33.3	37.0/99.2<br>0.3/33.3	3.0/88.5<br>0.5/19.6	1.6/80.9<br>0/0	37.3/98.6<br>0.9/20.6
OncoSNP-SEQ:4	1.8/65.5<br>0.1/12.5	5.0/80.9<br>0/0	5.3/90.0<br>0/0	5.5/82.3<br>0/0	5.6/86.5<br>0/0	0.8/100<br>0/0	5.4/82.0<br>0/0	10.2/92.7<br>0/0	0.2/100<br>0/0	5.1/81.2<br>0/0	5.1/81.2<br>0/0	5.2/81.5<br>0/0	5.3/91.5<br>0/0	22.8/67.2<br>2.5/19.2	17.4/92.6<br>0/0	20.6/98.1<br>0/0	20.9/97.6<br>0/0	18.7/94.5<br>0/0	19.7/97.0<br>0/0	21.9/98.6<br>0/0	21.7/97.7<br>0/0	20.8/97.6<br>0/0	22.4/98.2<br>0/0
PennCNV-Seq:3	5.2/89.8<br>0/0	11.1/87.5<br>0.3/25.0	12.5/95.4<br>0.1/25.0	13.2/97.8<br>0/25.0	10.7/94.7<br>0/0	7.2/97.3<br>0.3/50.0	12.9/97.7<br>0/0	19.9/89.7<br>0.3/25.0	5.7/92.0<br>0.5/50.0	12.5/98.4<br>0/0	12.5/98.4<br>0/0	12.5/97.6<br>0/0	11.2/99.1<br>0.1/25.0	17.4/92.6<br>0/0	37.3/85.4<br>2.0/58.4	36.0/98.1<br>0/0	32.0/95.2<br>0/0	29.2/88.5<br>0.3/25.0	34.7/98.3<br>0/0	37.7/98.4<br>0/0	33.2/95.4<br>0/0	25.2/95.5<br>0/0	38.0/96.9<br>0/0
Pindel:4	11.4/89.8<br>0.8/75.0	33.9/96.8<br>0.6/100	24.8/98.4<br>0.1/25.0	41.8/99.5<br>1.3/10.9	21.5/97.7<br>0.5/87.5	15.9/98.1<br>0/0	41.0/99.5<br>0.5/9.3	29.0/96.6<br>0/0	15.5/96.9<br>0.1/25.0	39.6/99.5<br>0.8/11.9	39.8/99.5<br>0.3/6.8	40.0/98.7<br>0.4/58.3	34.9/98.5<br>1.0/18.2	20.6/98.1<br>0/0	36.0/98.1<br>0/0	42.8/98.4<br>3.4/15.3	1.1/70.5<br>1.1/19.7	23.6/95.9<br>0.1/12.5	38.1/98.4<br>1.2/68.2	42.5/99.0<br>0.5/45.2	1.6/77.2<br>1.6/23.4	1.0/73.3<br>1.0/30.8	42.7/98.8<br>3.2/21.0
RAPTR:3	6.3/87.6<br>0.6/64.6	4.7/77.4<br>0.1/25.0	14.8/96.1<br>0.1/12.5	1.2/65.0<br>1.1/6.8	13.1/97.0<br>0.6/87.5	4.2/93.4<br>0.1/25.0	1.0/73.3<br>0/0	23.2/96.6<br>0.1/25.0	4.0/89.1<br>0/0	1.4/83.3<br>0.4/4.5	0.8/75.0<br>0/0	1.2/72.2<br>0.1/12.5	2.7/87.5<br>0.5/16.1	20.9/97.6<br>0/0	32.0/95.2<br>0/0	1.1/70.5<br>1.1/19.7	42.9/95.1<br>3.2/9.1	28.5/95.6<br>0.1/8.3	37.0/97.6<br>0.8/38.3	41.8/99.0<br>0.5/28.8	42.9/95.1<br>2.6/16.0	39.4/98.5<br>1.1/24.0	42.7/95.5<br>3.0/10.0
readDepth:6	8.5/82.6<br>0.9/55.5	22.9/88.5<br>0.9/39.6	25.6/94.1<br>1.3/49.6	25.4/96.2<br>0/0	22.5/94.5<br>0.8/42.5	10.5/94.6<br>0.8/66.7	25.1/96.2<br>0/0	24.9/92.2<br>0.5/45.8	9.6/92.3<br>0.1/12.5	24.3/96.4<br>0/0	23.9/96.4<br>0/0	24.4/96.0<br>0/0	21.5/94.3<br>1.1/58.3	18.7/94.5<br>0/0	29.2/88.5<br>0.3/25.0	23.6/95.9<br>0.1/12.5	28.5/95.6<br>0.1/8.3	33.9/77.4<br>4.5/29.3	19.1/92.7<br>0/0	21.4/94.3<br>0/0	16.2/91.6<br>0.3/25.0	12.6/85.9<br>0.3/37.5	21.6/91.9<br>0.4/33.3
Sniffles:2	11.4/82.1<br>0.5/55.5	32.6/97.0<br>0.5/100	24.7/98.0<br>0/33.3	39.7/99.5<br>0/0	20.3/96.2<br>0/33.3	15.7/96.9<br>0/33.3	39.6/99.7<br>0/0	28.3/96.6<br>0/0	15.4/96.2<br>0/0	38.7/99.7<br>0/0	38.5/99.7<br>0/0	38.2/98.7<br>0.5/66.7	33.4/97.9<br>0.2/33.3	19.7/97.0<br>0/0	34.7/98.3<br>0/0	38.1/98.4<br>1.2/68.2	37.0/97.6<br>0.8/38.3	19.1/92.7<br>0/0	41.5/80.7<br>2.0/20.9	40.5/98.5<br>0.8/100	4.0/78.8<br>0.8/57.8	2.1/73.3<br>0.2/11.1	40.9/97.8<br>1.5/42.9
SoftSV:6	11.9/89.5<br>0.3/37.5	35.6/97.0<br>0.5/100	26.7/98.5<br>0/0	44.5/99.5<br>0.1/12.5	22.4/97.8<br>0/0	16.5/98.2<br>0/0	43.6/99.5<br>0/0	31.0/97.2<br>0/0	16.2/97.0<br>0.1/25.0	42.1/99.5<br>0/0	42.2/99.5<br>0/0	42.8/99.0<br>0.4/62.5	37.0/99.2<br>0.3/33.3	21.9/98.6<br>0/0	37.7/98.4<br>0/0	42.5/99.0<br>0.5/45.2	41.8/99.0<br>0.5/28.8	21.4/94.3<br>0/0	40.5/98.5<br>0.8/100	46.0/98.7<br>0.5/20.6	1.6/73.9<br>0.1/6.3	0.9/71.4<br>0/0	45.7/98.9<br>0.5/21.9
SVDetect:3	6.6/87.0<br>0.6/51.7	5.2/79.1<br>0.3/25.0	16.4/96.5<br>0.1/12.5	1.5/66.6<br>1.3/8.4	13.9/97.2<br>0.5/62.5	5.2/94.6<br>0/0	1.4/78.9<br>0/0	25.0/96.5<br>0/0	4.5/90.1<br>0/0	1.4/78.9<br>0.9/12.5	1.1/75.0<br>0.3/6.9	1.3/70.0<br>0.1/8.3	3.0/88.5<br>0.5/19.6	21.7/97.7<br>0/0	33.2/95.4<br>0/0	1.6/77.2<br>1.6/23.4	42.9/95.1<br>2.6/16.0	16.2/91.6<br>0.3/25.0	4.0/78.8<br>0.8/57.8	1.6/73.9<br>0.1/6.3	47.1/89.2<br>3.7/10.5	41.7/97.4<br>1.3/27.5	46.9/92.3<br>3.1/11.1
TIDDIT:3	4.1/87.5<br>0.5/56.3	3.3/69.3<br>0.1/25.0	10.4/93.7<br>0.1/8.3	0.9/58.8<br>0.6/12.1	9.4/95.0<br>0.5/75.0	3.0/88.5<br>0/0	0.9/66.6<br>0/0	19.9/95.7<br>0/0	1.2/72.2<br>0/0	0.8/60.0<br>0.1/3.1	0.6/58.3<br>0/0	0.9/71.4<br>0/0	1.6/80.9<br>0/0	20.8/97.6<br>0/0	25.2/95.5<br>0/0	1.0/73.3<br>1.0/30.8	39.4/98.5<br>1.1/24.0	12.6/85.9<br>0.3/37.5	2.1/73.3<br>0.2/11.1	0.9/71.4<br>0/0	41.7/97.4<br>1.3/27.5	43.4/95.4<br>1.5/17.0	43.5/97.5<br>1.5/21.1
Ulysses:3	12.1/89.0<br>0.8/53.5	36.0/95.0<br>0.8/91.7	27.2/97.5<br>0.1/8.3	44.8/98.0<br>2.4/7.1	23.0/97.8<br>0.6/75.0	16.6/98.2<br>0.1/25.0	43.8/98.8<br>0.6/6.2	31.3/96.9<br>0.1/25.0	16.1/97.0<br>0.1/12.5	42.4/98.8<br>1.0/6.1	42.3/99.0<br>0.4/4.0	43.1/99.0<br>0.4/31.3	37.3/98.6<br>0.9/20.6	22.4/98.2<br>0/0	38.0/96.9<br>0/0	42.7/98.8<br>3.2/21.0	42.7/95.5<br>3.0/10.0	21.6/91.9<br>0.4/33.3	40.9/97.8<br>1.5/42.9	45.7/98.9<br>0.5/21.9	46.9/92.3<br>3.1/11.1	43.5/97.5<br>1.5/21.1	46.9/91.6<br>4.6/7.6

Figure S21. Recall and precision of SVs commonly called between a pair of SV detection algorithms for the DUP-L category. DUPs in the large size-range (100 Kb–2 Mb), called using the indicated algorithms, were filtered with the minimum number of reads supporting the called SVs, indicated with the suffix number of the algorithm name. The DUPs overlapping between the filtered SV sets from a pair of the indicated algorithms were selected, and the recall and precision of the selected DUPs were determined. Recall and precision percentages are presented as in Figure S15. The data contained in the top 20th percentile of the combined precision scores for the simulated and real data are highlighted with a red background, and the next data contained in the top 21st–50th percentile of the combined precision scores are shown with a pale red background.

Algorithm	1-2-3-SV:12	BreakDancer:5	DELLY:8	GASVPro:8	GRIDSS:7	inGAP-sv:8	Lumpy:9	Manta:3	Meerkat:7	MetaSV:3	Pindel:9	PRISM:3	Sniffles:3	SoftSV:7	SvABA:3	SVelter:3	TIDDIT:8	Ulysses:3	VariationHunter:4	Wham:5
1-2-3-SV:12	6.4/70.2<br>1.3/33.7	0.7/73.6<br>0.2/38.7	5.4/94.6<br>0.8/41.6	2.3/85.8<br>0.4/48.2	5.4/94.6<br>0.7/44.6	4.8/95.5<br>0.5/54.5	4.9/97.7<br>0.3/75.3	5.3/96.0<br>0.7/44.1	5.1/95.8<br>0.2/61.3	4.3/99.3<br>0.3/61.6	5.2/97.4<br>0.5/46.8	5.5/97.0<br>0.5/46.6	5.1/94.8<br>0.2/75.0	5.3/96.9<br>0.2/75.9	4.8/96.1<br>0.4/41.9	3.6/96.9<br>0.1/76.9	5.5/92.5<br>0.9/44.3	1.6/82.1<br>0.1/24.0	2.1/93.9<br>0.3/44.4	4.8/96.0<br>0.5/51.5
BreakDancer:5	0.7/73.6<br>0.2/38.7	4.4/72.8<br>0.8/19.2	5.0/97.8<br>0.8/38.7	1.8/90.2<br>0.4/40.9	5.0/98.3<br>0.7/36.9	4.5/98.7<br>0.4/47.1	4.8/98.2<br>0.2/57.0	4.9/97.8<br>0.7/37.4	4.6/98.8<br>0.2/48.8	4.2/98.0<br>0.4/44.2	4.8/98.8<br>0.4/40.3	5.0/98.9<br>0.4/56.8	4.7/98.8<br>0.2/84.3	4.8/98.8<br>0.2/50.0	4.6/98.2<br>0.4/49.2	3.5/98.4<br>0.1/56.7	4.7/94.9<br>0.8/41.8	1.9/85.3<br>0.1/19.7	1.8/88.3<br>0.2/55.8	4.6/97.6<br>0.5/38.5
DELLY:8	5.4/94.6<br>0.8/41.6	5.0/97.8<br>0.8/38.7	6.8/95.7<br>1.7/30.9	2.7/93.3<br>0.7/34.8	6.7/97.5<br>1.5/36.5	5.5/98.5<br>0.9/43.5	5.9/99.0<br>0.7/52.7	6.5/97.5<br>1.5/34.9	6.3/97.8<br>0.5/45.9	4.7/98.8<br>0.5/47.2	6.3/99.1<br>1.0/43.6	6.3/99.5<br>0.7/47.0	5.8/99.0<br>0.4/87.7	6.5/100<br>0.5/56.8	5.6/99.0<br>0.6/36.7	4.4/98.7<br>0.4/62.1	5.9/98.6<br>1.1/34.9	0.1/66.6<br>0.1/15.0	2.6/100<br>0.6/55.5	5.8/97.2<br>1.0/38.5
GASVPro:8	2.3/85.8<br>0.4/48.2	1.8/90.2<br>0.4/40.9	2.7/93.3<br>0.7/34.8	2.6/80.6<br>0.7/19.3	2.5/97.8<br>0.5/43.0	2.0/98.6<br>0.3/49.6	2.2/98.7<br>0.2/48.9	2.6/100<br>0.6/36.9	2.4/100<br>0.2/50.3	1.8/95.5<br>0.2/51.8	2.5/100<br>0.4/52.6	2.4/100<br>0.3/54.0	2.2/97.5<br>0.1/100	2.5/100<br>0.2/73.1	2.0/100<br>0.2/54.9	1.4/98.1<br>0.2/72.1	2.2/98.7<br>0.4/37.4	0.02/100<br>0/4.2	0.8/100<br>0.1/28.8	2.2/98.7<br>0.4/49.9
GRIDSS:7	5.4/94.6<br>0.7/44.6	5.0/98.3<br>0.7/36.9	6.7/97.5<br>1.5/36.5	2.5/97.8<br>0.5/43.0	6.8/96.8<br>1.7/26.5	5.5/99.0<br>0.8/49.2	6.0/99.5<br>0.6/46.9	6.6/98.3<br>1.5/32.0	6.3/98.2<br>0.5/48.8	4.7/98.8<br>0.5/43.8	6.3/99.5<br>1.0/39.3	6.2/99.5<br>0.7/51.0	5.8/99.5<br>0.4/87.9	6.5/100<br>0.5/45.9	5.6/99.5<br>0.6/37.9	4.4/99.3<br>0.4/66.8	5.9/99.0<br>1.1/33.2	0.1/100<br>0.1/14.9	2.5/100<br>0.4/67.9	5.8/98.5<br>1.1/33.9
inGAP-sv:8	4.8/95.5<br>0.5/54.5	4.5/98.7<br>0.4/47.1	5.5/98.5<br>0.9/43.5	2.0/98.6<br>0.3/49.6	5.5/99.0<br>0.8/49.2	5.5/99.0<br>0.8/36.8	5.1/99.4<br>0.4/61.8	5.4/99.4<br>0.8/42.8	5.2/98.9<br>0.3/60.5	4.2/98.7<br>0.3/55.9	5.3/99.4<br>0.4/51.7	5.2/99.4<br>0.4/59.9	5.0/99.4<br>0.2/100	5.4/100<br>0.3/73.1	4.8/99.4<br>0.4/45.2	4.1/99.3<br>0.2/80.8	5.0/99.4<br>0.7/48.8	0/0<br>0/11.5	2.1/100<br>0.3/70.1	5.0/99.4<br>0.5/50.2
Lumpy:9	4.9/97.7<br>0.3/75.3	4.8/98.2<br>0.2/57.0	5.9/99.0<br>0.7/52.7	2.2/98.7<br>0.2/48.9	6.0/99.5<br>0.6/46.9	5.1/99.4<br>0.4/61.8	6.0/100<br>0.7/40.2	6.0/100<br>0.6/50.3	5.6/99.0<br>0.2/68.5	4.5/98.7<br>0.2/92.9	5.7/100<br>0.4/79.1	5.4/100<br>0.3/90.8	5.2/100<br>0.2/100	5.9/100<br>0.4/50.4	5.3/100<br>0.3/71.2	4.3/100<br>0.3/64.8	5.5/99.4<br>0.5/69.4	0.02/50.0<br>0/15.2	2.3/100<br>0.4/76.2	5.6/100<br>0.4/47.1
Manta:3	5.3/96.0<br>0.7/44.1	4.9/97.8<br>0.7/37.4	6.5/97.5<br>1.5/34.9	2.6/100<br>0.6/36.9	6.6/98.3<br>1.5/32.0	5.4/99.4<br>0.8/42.8	6.0/100<br>0.6/50.3	6.7/98.3<br>1.6/26.1	6.2/98.6<br>0.5/45.2	4.7/98.2<br>0.5/44.0	6.3/99.5<br>0.9/38.1	6.1/100<br>0.6/45.1	5.7/99.5<br>0.3/90.4	6.4/100<br>0.4/44.9	5.6/100<br>0.6/35.7	4.4/99.3<br>0.4/57.6	5.8/99.0<br>1.1/34.1	0.1/75.0<br>0.1/15.4	2.5/100<br>0.4/56.6	5.9/99.5<br>1.0/33.5
Meerkat:7	5.1/95.8<br>0.2/61.3	4.6/98.8<br>0.2/48.8	6.3/97.8<br>0.5/45.9	2.4/100<br>0.2/50.3	6.3/98.2<br>0.5/48.8	5.2/98.9<br>0.3/60.5	5.6/99.0<br>0.2/68.5	6.2/98.6<br>0.5/45.2	6.9/92.9<br>0.6/33.7	4.4/96.9<br>0.2/66.6	6.2/96.9<br>0.4/56.7	6.1/95.6<br>0.2/67.0	5.8/98.5<br>0.1/50.0	6.5/98.7<br>0.2/76.3	5.4/98.9<br>0.2/53.1	4.2/98.0<br>0.2/71.7	5.8/97.6<br>0.4/54.0	0.1/75.0<br>0/29.2	2.6/97.9<br>0.2/61.0	5.5/98.5<br>0.4/54.2
MetaSV:3	4.3/99.3<br>0.3/61.6	4.2/98.0<br>0.4/44.2	4.7/98.8<br>0.5/47.2	1.8/95.5<br>0.2/51.8	4.7/98.8<br>0.5/43.8	4.2/98.7<br>0.3/55.9	4.5/98.7<br>0.2/92.9	4.7/98.2<br>0.5/44.0	4.4/96.9<br>0.2/66.6	4.6/98.8<br>0.5/44.2	4.6/98.8<br>0.4/49.2	4.6/98.8<br>0.3/61.4	4.3/99.3<br>0.1/66.7	4.5/100<br>0.1/83.9	4.2/99.3<br>0.2/48.4	3.4/99.1<br>0.1/56.7	4.4/100<br>0.4/50.1	0.02/100<br>0.1/30.9	1.8/100<br>0.1/83.3	4.3/99.3<br>0.3/41.6
Pindel:9	5.2/97.4<br>0.5/46.8	4.8/98.8<br>0.4/40.3	6.3/99.1<br>1.0/43.6	2.5/100<br>0.4/52.6	6.3/99.5<br>1.0/39.3	5.3/99.4<br>0.4/51.7	5.7/100<br>0.4/79.1	6.3/99.5<br>0.9/38.1	6.2/96.9<br>0.4/56.7	4.6/98.8<br>0.4/49.2	6.8/88.4<br>1.1/30.4	6.1/98.6<br>0.5/53.2	5.6/98.5<br>0.2/78.4	6.3/100<br>0.3/80.1	5.4/100<br>0.5/49.3	4.2/99.3<br>0.3/80.5	5.6/98.5<br>0.7/41.3	0.1/62.5<br>0.1/14.8	2.5/100<br>0.4/60.1	5.6/99.5<br>0.7/40.5
PRISM:3	5.5/97.0<br>0.5/46.6	5.0/98.9<br>0.4/56.8	6.3/99.5<br>0.7/47.0	2.4/100<br>0.3/54.0	6.2/99.5<br>0.7/51.0	5.2/99.4<br>0.4/59.9	5.4/100<br>0.3/90.8	6.1/100<br>0.6/45.1	6.1/95.6<br>0.2/67.0	4.6/98.8<br>0.3/61.4	6.1/98.6<br>0.5/53.2	6.8/95.7<br>0.8/36.7	6.0/99.0<br>0.2/81.4	6.2/99.5<br>0.2/86.9	5.3/99.4<br>0.4/53.5	3.9/99.2<br>0.1/83.0	5.6/98.5<br>0.5/44.6	0.1/87.5<br>0/2.3	2.7/100<br>0.3/56.9	5.3/100<br>0.5/58.6
Sniffles:3	5.1/94.8<br>0.2/75.0	4.7/98.8<br>0.2/84.3	5.8/99.0<br>0.4/87.7	2.2/97.5<br>0.1/100	5.8/99.5<br>0.4/87.9	5.0/99.4<br>0.2/100	5.2/100<br>0.2/100	5.7/99.5<br>0.3/90.4	5.8/98.5<br>0.1/50.0	4.3/99.3<br>0.1/66.7	5.6/98.5<br>0.2/78.4	6.0/99.0<br>0.2/81.4	6.2/70.3<br>0.6/44.1	5.8/94.5<br>0.2/100	5.0/95.7<br>0.2/80.8	3.8/95.1<br>0.1/91.7	5.3/93.6<br>0.2/81.4	0.1/50.0<br>0/33.3	2.3/93.3<br>0.3/62.7	5.0/96.2<br>0.3/96.8
SoftSV:7	5.3/96.9<br>0.2/75.9	4.8/98.8<br>0.2/50.0	6.5/100<br>0.5/56.8	2.5/100<br>0.2/73.1	6.5/100<br>0.5/45.9	5.4/100<br>0.3/73.1	5.9/100<br>0.4/50.4	6.4/100<br>0.4/44.9	6.5/98.7<br>0.2/76.3	4.5/100<br>0.1/83.9	6.3/100<br>0.3/80.1	6.2/99.5<br>0.2/86.9	5.8/94.5<br>0.2/100	7.0/100<br>0.5/35.1	5.5/100<br>0.3/74.7	4.3/99.3<br>0.3/66.0	5.9/99.0<br>0.3/67.2	0.1/75.0<br>0/16.5	2.5/100<br>0.3/82.1	5.7/100<br>0.4/50.2
SvABA:3	4.8/96.1<br>0.4/41.9	4.6/98.2<br>0.4/49.2	5.6/99.0<br>0.6/36.7	2.0/100<br>0.2/54.9	5.6/99.5<br>0.6/37.9	4.8/99.4<br>0.4/45.2	5.3/100<br>0.3/71.2	5.6/100<br>0.6/35.7	5.4/98.9<br>0.2/53.1	4.2/99.3<br>0.2/48.4	5.4/100<br>0.5/49.3	5.3/99.4<br>0.4/53.5	5.0/95.7<br>0.2/80.8	5.5/100<br>0.3/74.7	5.6/99.5<br>0.7/33.8	3.9/99.3<br>0.2/63.0	5.1/98.9<br>0.4/37.5	0.1/80.0<br>0.1/20.0	2.1/100<br>0.3/71.5	5.1/99.4<br>0.5/39.3
SVelter:3	3.6/96.9<br>0.1/76.9	3.5/98.4<br>0.1/56.7	4.4/98.7<br>0.4/62.1	1.4/98.1<br>0.2/72.1	4.4/99.3<br>0.4/66.8	4.1/99.3<br>0.2/80.8	4.3/100<br>0.3/64.8	4.4/99.3<br>0.4/57.6	4.2/98.0<br>0.2/71.7	3.4/99.1<br>0.1/56.7	4.2/99.3<br>0.3/80.5	3.9/99.2<br>0.1/83.0	3.8/95.1<br>0.1/91.7	4.3/99.3<br>0.3/66.0	3.9/99.3<br>0.2/63.0	4.4/89.8<br>0.4/47.1	4.1/100<br>0.3/81.5	0.02/100<br>0/28.1	1.5/100<br>0.2/84.7	4.2/100<br>0.3/60.8
TIDDIT:8	5.5/92.5<br>0.9/44.3	4.7/94.9<br>0.8/41.8	5.9/98.6<br>1.1/34.9	2.2/98.7<br>0.4/37.4	5.9/99.0<br>1.1/33.2	5.0/99.4<br>0.7/48.8	5.5/99.4<br>0.5/69.4	5.8/99.0<br>1.1/34.1	5.8/97.6<br>0.4/54.0	4.4/100<br>0.4/50.1	5.6/98.5<br>0.7/41.3	5.6/98.5<br>0.5/44.6	5.3/93.6<br>0.2/81.4	5.9/99.0<br>0.3/67.2	5.1/98.9<br>0.4/37.5	4.1/100<br>0.3/81.5	6.5/89.9<br>1.5/27.0	2.2/98.7<br>0.2/18.6	2.4/98.8<br>0.4/56.0	5.4/98.9<br>0.9/42.5
Ulysses:3	1.6/82.1<br>0.1/24.0	1.9/85.3<br>0.1/19.7	0.1/66.6<br>0.1/15.0	0.02/100<br>0/4.2	0.1/100<br>0.1/14.9	0/0<br>0/11.5	0.02/50.0<br>0/15.2	0.1/75.0<br>0.1/15.4	0.1/75.0<br>0/29.2	0.02/100<br>0.1/30.9	0.1/62.5<br>0.1/14.8	0.1/87.5<br>0/2.3	0.1/50.0<br>0/33.3	0.1/75.0<br>0/16.5	0.1/80.0<br>0.1/20.0	0.02/100<br>0/28.1	2.2/98.7<br>0.2/18.6	2.4/78.3<br>0.4/8.0	1.1/95.3<br>0.2/35.8	2.3/97.6<br>0.1/20.6
VariationHunter:4	2.1/93.9<br>0.3/44.4	1.8/88.3<br>0.2/55.8	2.6/100<br>0.6/55.5	0.8/100<br>0.1/28.8	2.5/100<br>0.4/67.9	2.1/100<br>0.3/70.1	2.3/100<br>0.4/76.2	2.5/100<br>0.4/56.6	2.6/97.9<br>0.2/61.0	1.8/100<br>0.1/83.3	2.5/100<br>0.4/60.1	2.7/100<br>0.3/56.9	2.3/93.3<br>0.3/62.7	2.5/100<br>0.3/82.1	2.1/100<br>0.3/71.5	1.5/100<br>0.2/84.7	2.4/98.8<br>0.4/56.0	1.1/95.3<br>0.2/35.8	2.8/90.9<br>1.6/19.0	2.2/100<br>0.3/85.8
Wham:5	4.8/96.0<br>0.5/51.5	4.6/97.6<br>0.5/38.5	5.8/97.2<br>1.0/38.5	2.2/98.7<br>0.4/49.9	5.8/98.5<br>1.1/33.9	5.0/99.4<br>0.5/50.2	5.6/100<br>0.4/47.1	5.9/99.5<br>1.0/33.5	5.5/98.5<br>0.4/54.2	4.3/99.3<br>0.3/41.6	5.6/99.5<br>0.7/40.5	5.3/100<br>0.5/58.6	5.0/96.2<br>0.3/96.8	5.7/100<br>0.4/50.2	5.1/99.4<br>0.5/39.3	4.2/100<br>0.3/60.8	5.4/98.9<br>0.9/42.5	2.3/97.6<br>0.1/20.6	2.2/100<br>0.3/85.8	5.9/95.1<br>1.1/23.9

Figure S22. Recall and precision of SVs commonly called between a pair of SV detection algorithms **for the INV category.** INVs, called using the indicated algorithms, were filtered with the minimum number of reads supporting the called SVs, indicated with the suffix number of the algorithm name. The INVs overlapping between the filtered SV sets from a pair of the indicated algorithms were selected, and the recall and precision of the selected INVs were determined. Recall and precision percentages are presented as in Figure S15. The data contained in the top 20th percentile of the combined precision scores for the simulated and real data are highlighted with a red background, and the next data contained in the top 21st–50th percentile of the combined precision scores are shown with a pale red background.

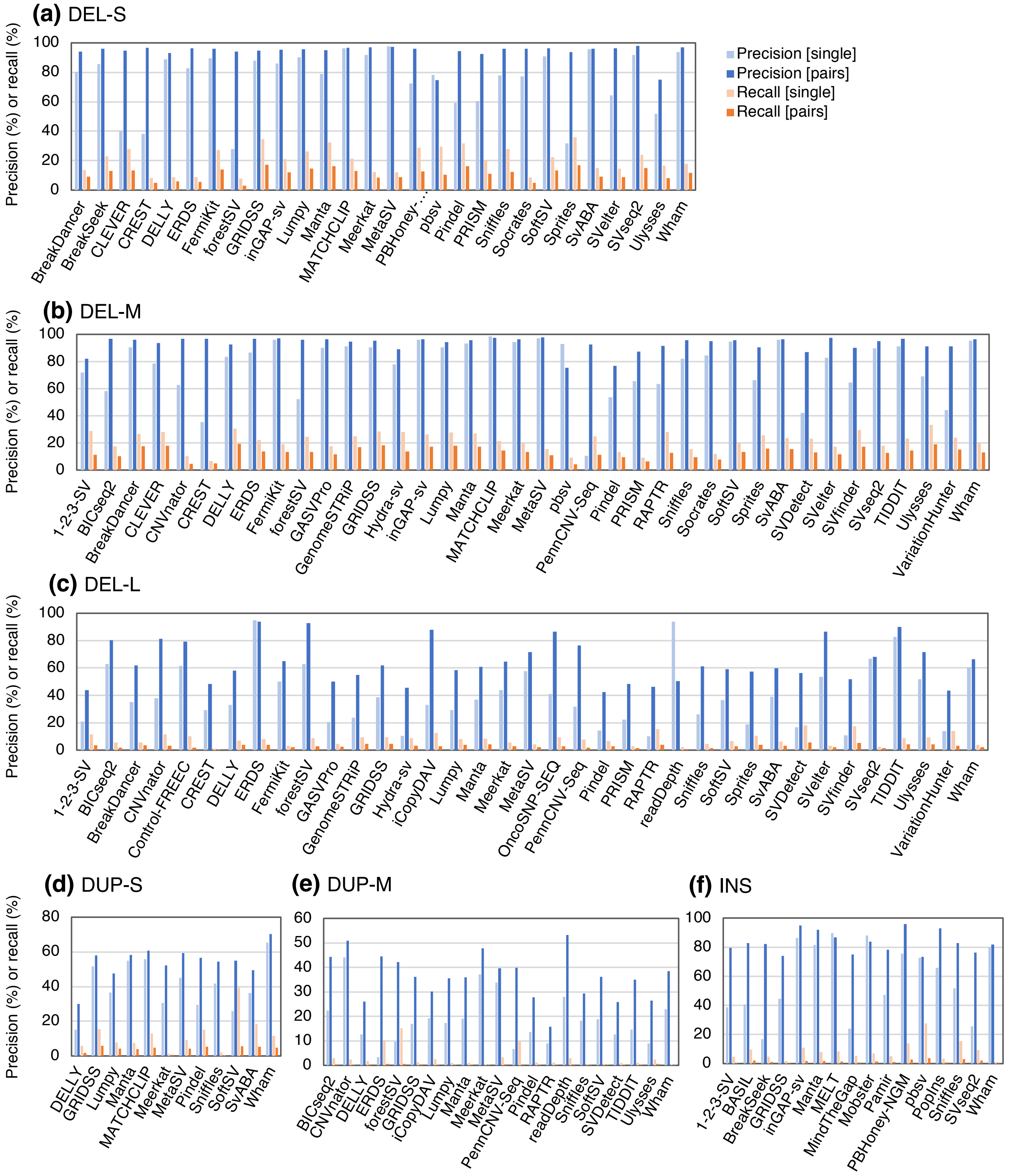

Figure S23. Mean precision and recall of overlapped calls between SV detection algorithms. Mean precision and recall values of overlapped calls between pairs of algorithms were determined for the different SV categories, DEL-S (**a**), DEL-M (**b**), DUP-L (**c**), DUP-S (**d**), DUP-M (**e**), and INS (**f**), using four (or three) sets of NA12878 real data. The mean precision and the mean recall are indicated with blue and orange bars, respectively. The precision and recall obtained with the indicated single tool are shown as light blue and light orange bars, respectively. The numerical data, including those for the other SV categories, are presented in Table S17 in Additional file 3.

Table S1. SV detection algorithms used in this study

Algorithms	SV types *1	Detection methods *2	Input data	Version (released year)	Reference
1-2-3-SV	DEL, INS, INV, TRA	RP	bam	1 (2012)	url *8
AS-GENSENG	DEL, DUP	RD	bam	1.0.2 (2015)	[1]
BASIL-ANISE	INS	RP, AS	bam	0.3.0 (2015)	[2]
BatVI	VEI	RP, SR	fastq	1.02 (2017)	[3]
BICseq2	DEL, DUP	RD	bam	0.2.4/0.7.2 (2016)	[4]
BreakDancer	DEL, INS, INV, TRA	RP	bam	1.3.6 (2015)	[5]
BreakSeek	DEL, INS	RP, SR	bam	1.2 (2015)	[6]
BreakSeq2	DEL, INS	SV-library	bam	2.2 (2015)	[7]
Breakway	DEL, INS	RP	bam	0.7.1 (2011)	[8]
CLEVER	DEL, INS	RP	bam	2 (2015)	[9]
CNVnator	DEL, DUP	RD	bam	0.3.2 (2015)	[10]
Control-FREEC	DEL, DUP	RD	bam	8.7 (2016)	[11]
CREST	DEL, INS, INV, TRA	RP, SR, AS	bam	1.0 (2011)	[12]
DELLY	DEL, DUP, INV, TRA	RP, SR	bam	0.7.2 (2016)	[13]
DIGTYPER	DUP, INV (genotyping)	RP, SR	bam	unknown (2017)	[14]
DINUMT	NUMT	RP	bam	0.0.23 (2014)	[15]
ERDS	DEL, DUP	RP, SR, RD	bam	1.1 (2013)	[16]
FermiKit	DEL, INS	AS	fastq	0.13 (2015)	[17]
forestSV	DEL, DUP	RP, RD, RF	bam	0.3.3 (2013)	[18]
GASVpro	DEL, INV	RP, RD	bam	1.2.1 (2013)	[19]
GenomeSTRiP	DEL [MS *4]	RP, RD	bam	2.00 (2016)	[20]
GRIDSS	DEL, DUP, INS, INV, TRA	RP, SR, AS	bam	1.5.0 (2017)	[21]
HGT-ID	VEI	RP, SR	bam	1.0 (2018)	[22]
Hydra-sv	DEL	RP, AS	bam	0.5.3 (2014)	[23]
iCopyDAV	DEL, DUP	RD	bam	1.0 (2018)	[24]
indelMINER	DEL	RP, SR	bam	0.10 (2015)	[25]
inGAP-sv	DEL, DUP, INS, INV	RP, RD	sam	3.1.1 (2014)	[26]
ITIS	MEI	RP, SR	bam	1.0 (2015)	[27]

laSV	DEL, DUP, INV, TRA	AS	fastq	1.0.2 (2015)	[28]
Lumpy	DEL, DUP, INV, TRA	RP, SR, RD	bam	0.2.9 (2015)	[29]
Manta	DEL, DUP, INS, INV, TRA	RP, SR, AS	bam	0.29.5 (2016)	[30]
MATCHCLIP	DEL, DUP	RP, SR, RD	bam	2.0 (2013)	[31]
Meerkat	DEL, DUP, INS, INV, TRA	RP, SR	bam	0.185 (2015)	[32]
MELT	MEI, (NUMT)	RP, (SR)	bam	2.0.1 (2016)	[33]
MetaSV	DEL, DUP, INS, INV	Meta (RP, SR, RD)	bam, etc *6	0.5.2 (2016)	[34]
MindTheGap	INS	k-mer, AS	fastq	0.6447 (2014)	[35]
Mobster	MEI, (NUMT, VEI)	RP, SR	bam	0.2.4.1 (2018)	[36]
OncoSNP-SEQ	DEL, DUP	RD	bam	2.0 (2015)	[37]
Pamir	INS	RP, SR, AS	fastq, bam	unknown (2017)	[38]
PBHoney *3	DEL, INS	LR (SR, SP)	fastq	15.8.24 (2015)	[39]
pbsv	DEL, INS	LR	fastq, bam	In SMRT Link v5.0.1	url *9
PennCNV-Seq	DEL, DUP	RD	bam	Unknown (2017)	[40]
Pindel	DEL, DUP, INS, INV, TRA	SR	bam	0.2.5 (2015)	[41]
PopIns	INS	RP, SR, AS	bam	unknown (2015)	[42]
PRISM	DEL, INS, INV	RP, SR	sam	1.1.6 (2012)	[43]
RAPTR-SV	DEL, DUP, INS	RP, SR	bam	0.0.15 (2015)	[44]
readDepth	DEL, DUP	RD	bam	0.9.8.4 (2015)	[45]
RetroSeq	MEI	RP, SR	bam	1.41 (2014)	[46]
Sniffles	DEL, DUP, INS, INV, TRA	LR (SR)	bam	1.0.6 (2017)	[47]
Socrates	DEL, INS	SR	bam	1.1 (2015)	[48]
SoftSearch	DEL, DUP, INS, INV, TRA	RP, SR	bam	2.4 (2014)	[49]
SoftSV	DEL, DUP, INV, TRA	RP, SR	bam	1.4.2 (2015)	[50]
SoloDel	DEL	RP, RD	bam	1.0.0 (2015)	[51]
Sprites	DEL	SR	bam	0.3 (2016)	[52]
SV^2	DEL, DUP (genotyping)	RP, SR, RD	bam	1.4.0 (2017)	[53]
SvABA	DEL, DUP, INS, INV	RP, SR, AS	bam	0.2.1 (2017)	[54]
SVDetect	DEL, DUP, INS, INV, TRA	RP	bam	0.8b (2013)	[55]
SVelter	DEL, DUP, INV	RP, SR, RD	bam	1.1 (2016)	[56]
SVfinder	DEL, INS, INV, TRA	RP	bam	unknown (2014)	[57]
SVSeq2	DEL, INS	SR	bam	2.2 (2015)	[58]

Tangram	MEI	RP, SR	bam	0.3.1 (2015)	[59]
Tea	MEI	RP, SR	bam	0.6.2 (2015)	[60]
TEMP	MEI	RP, SR	bam	1.0.5 (2017)	[61]
TIDDIT	DEL, DUP, INV, TRA	RP, SR, RD	bam	1.0.2 (2017)	[62]
Ulysses	DEL, DUP, INV [MP-lib *5]	RP	bam	1.0 (2015)	[63]
VariationHunter	DEL, INS, INV	RP	divet *7	0.04 (2012)	[64]
VirusFinder2	VEI	Others	bam	2.0 (2015)	[65]
VirusSeq	VEI	RP	fastq	Unknown (2012)	[66]
Wham	DEL, DUP, INS, INV	RP, SR	bam	1.8 (2016)	[67]

*1 MEI: mobile element insertion, NUMT: nuclear mitochondrial genome insertion, VEI: virus element insertion. For MELT and Mobster, NUMT and/or VEI can be detected by a modification of the pipelines.

*2 RP: read pairs-based signal, RD: read depth-based signal, SR: split (soft-clipped) reads-based signal, AS: assembly-based approach, LR: method using long reads, SP: signals within the span of reads, SV library: short read mapping to the break point regions from the public SV libraries, RF: Random Forest classifiers, Meta: integrating outputs from multiple algorithms, k-mer: k-mer-based detection of insertion sites, Others: using CREST and SVDetect to detect virus integration sites.

*3 PBHoney uses PacBio reads as input. PBHoney-NGM used in this study is a modification of PBHoney, that uses the NGM-LR long read aligner (v0.2.6b) and altered options instead of using the blasr aligner and the default options.

*4 MS: optimized for SV calling with multiple samples.

*5 MP-lib: optimized for data with mate-pair libraries.

*6 Output files from BreakDancer, Pindel, CNVnator, and BreakSeq2.

*7 DIVET alignment file with mrFAST.

*8 https://github.com/Vityay/1-2-3-SV.

*9 https://github.com/PacificBiosciences/pbsv.

Table S2. List of algorithms that did not work in our computational environment

Algorithms	SV type	Version (release year)	Reference
Alu-detect	MEI (ALU)	1.3 (2015)	[68]
Gustaf	DEL, DUP, INV, TRA	1.0.9 (2017)	[69]
ngs_te_mapper	MEI	Unknown (2015)	[70]
PEMer	DEL, INS, INV	Unknown (2009)	[71]
PeSV-Fisher	DEL, DUP, INV, TRA	0.93 (2012)	[72]
RelocaTE	MEI	1.0.5 (2014)	[73]
RDXplorer	DEL, DUP	3.2 (2012)	[74]
Spanner	DEL, DUP, INV, TRA, MEI	7.0 (2011)	*1
TE-locate	MEI	1.0 (2015)	[75]
TE-Tracker	MEI	Unknown (2016)	[76]

*1 https://github.com/chipstewart/Spanner.

Table S3. Whole genome sequencing reads used in this study

Human WGS data	Read length (bp)	Insert size (bp)	Genome coverage	Platform	Accession number of NCBI SRA or DDBJ DRA
NA12878 (data1) [*1]	100, 102	320	31×	HiSeq 2000	ERP001775 (ERR174336–ERR174340)
NA12878 (data2) [*2]	101	318	30×	HiSeq 2000	ERP001229 (ERR091571–ERR091573)
NA12878 (data3) [*3]	148	546	31×	HiSeq 2500	SRP047086 (SRR2052337–SRR2052352)
NA12878 (data4) [*4]	251	681	30×	HiSeq 2500	SRP056043 (SRR1910373)
NA12891 [*5]	101	296	52×	HiSeq 2000	ERR194160
NA12892 [*5]	101	303	56×	HiSeq 2000	ERR194161
NA12878 (PacBio-data1) [*6]	7,424	–	10×	PacBio RS II	SRP043609 (SRR1947646–SRR1950271)
NA12878 (PacBio-data2) [*7]	19,647	–	10×	PacBio RS II	SRP076397 (SRR3658380–SRR3658389)
NA12878 (PacBio-data3) [*8]	7,716	–	10.5×	Sequel	Obtained at the PacBio website [*13]
HG00514 [*9]	125	572	30×	HiSeq 2500	ERR894729, ERR903030
HG00514 (PacBio-HG00514) [*10]	15,994	–	10×	PacBio RS II	ERX1366175, ERR125124, ERR125128, ERR125130, ERR125!32
HG002 (PacBio-HG002) [*11]	7,278	–	10×	PacBio RS II	SRX1033794
Simulated data (Sim-A, Sim-MEI, Sim-VEI, Sim-NUMT)	125	500	30×	–	Data generated with the VarSim and ART simulators
Simulated data (Sim-A-PacBio) [*12]	7,500	–	10×	–	Data generated with the PBSIM simulator

[*1] NA12878 (data1): Deep whole genome sequence data from the CEPH 1463 family (Illumina Platinum Genomes).

[*2] NA12878 (data2): Whole human genome sequencing of an CEPH/UTAH female individual (HapMap: NA12878) using the Illumina HiSeq 2000 and paired 100 base reads.

[*3] NA12878 (data3): A public–private–academic consortium hosted by NIST to develop reference materials and standards for clinical sequencing.

[*4] NA12878 (data4): Homo sapiens PCR-free whole genome shotgun sequencing of NA12878.

[*5] NA12891, NA12892: parents of NA12978, deep whole genome sequence data from the CEPH 1463 family (Illumina Platinum Genomes).

[*6] NA12878 (PacBio-data1): Mean read size: 7,424 bp, N50 of reads: 15,401 bp, sequenced with PacBio RS II and P5-C3 chemistry.

[*7] NA12878 (PacBio-data2): Mean read size: 19,647 bp, N50 of reads: 29,247 bp.

[*8] NA12878 (PacBio-data3): Mean read size: 7,716 bp, N50 of reads: 11,823 bp, sequenced with the PacBio Sequel system.

[*9] HG00514: Illumina PCR-free sequencing of 1000 Genomes Han Chinese trios for SV discovery.

[*10] HG00514 PacBio data from the Human Genome Structural Variation Consortium.

[*11] HG002 PacBio data from the Genome in a Bottle (GIAB) Consortium.

[*12] Sim-A-PacBio: Mean read size: 7,500 bp, N50 of reads: 7,846 bp, generated with the PBSIM simulator.

[*13]

http://www.pacb.com/blog/identifying-structural-variants-na12878-low-fold-coverage-sequencing-pacbio-sequel-system/.

Table S4. Components of reference SVs for real and simulated datasets

(A) Sim-A and Real data

Data set	Number of reference SVs										
	DEL					DUP				INS	INV
	SS [*1] [Het]	S [*2] [Het]	M [*3] [Het]	L [*4] [Het]	Total [Het]	S [Het]	M [Het]	L [Het]	Total [Het]	[Het]	[Het]
Sim-A	122 [71]	1,937 [1,133]	1,301 [760]	166 [100]	3,526 [2,064]	166 [83]	998 [553]	492 [295]	1,656 [931]	2,819 [1,667]	309 [190]
Real (NA12878)	1,192	5,558	2,332	102	9,184	877	1,585	146	2,608	13,669	275
Real-GT (NA12878)	0	36 [22]	283 [196]	16 [6]	335 [224]	17 [11]	88 [48]	15 [8]	120 [67]	0	0
Real (HG00514) [*5]	4,782	7,604	2,838	28	15,249	472	537	18	1,027	16,543	0
Real (HG002) [*5]	13,869	7,487	1,572	39	22,967	0	0	0	0	22,864	0

[*1] Super small size: 50–100 bp.

[*2] Small size: 101–1,000 bp (50–1,000 bp for DUP).

[*3] Middle size: 1,001–100,000 bp.

[*4] Large size: 100,001–2,000,000 bp.

[*5] A minimal 30 bp of variants were extracted.

The number of heterozygous alleles is indicated within brackets.

(B) Sim-MEI, Sim-NUMT, and Sim-VEI data

Data set	Number of reference SVs		
	MEI [Het]	NUMT [Het]	VEI [Het]
Sim-MEI	651 (ALU: 480, L1: 94, SVA: 37, HERVK: 40) [429]	0	0
Sim-NUMT	0	200 [132]	0
Sim-VEI	0	0	100 [64]

These simulation data were generated only for chromosome 17.

The number of heterozygous alleles is indicated within brackets.

(C) Component of SVs identified in the 1000 Genomes project [77]

SV type	All Types	DEL	DUP	INV	MEI	NUMT
Total SV number	68,818	42,279	8,954	786	16,631	168
Number per sample	4,405	2,788	357	37	1,218	5

Table S12. Effect of the length, insert size, and coverage of reads on precision and recall of SV detection algorithms (summarized data from Figure S13 in Additional file 2)

SV type	Algorithms	Read length		Insert size		Read coverage	
		Precision	Recall	Precision	Recall	Precision	Recall
DEL	1-2-3-SV	I	C	D	D	D	I
	BreakDancer	C	C	D	D	I	I
	CLEVER	R-125	D	I-500	I	C	I
	CNVnator	C	C	C	C	D	I
	DELLY	C	C	C	D	D	I
	forestSV	C	C	C	C	I	I
	inGAP-sv	C	C	C	D	D	I
	Lumpy	C	I	C	I	D	I
	Manta	C	U	C	C	D	I
	Meerkat	C	C	C	D	C	I
	MetaSV	C	R-125	C	D	C	I
	Pindel	D	R-125	C	C	D	I
	PRISM	C	D	C	C	D	I
	RAPTR	I	C	D	D	D	I
	Socrates	C	R-125	C	C	C	I
	SoftSV	C	C	C	C	D	I
	SVseq2	C	R-125	C	C	D	I
	Ulysses	C	D	C	D	C	I
DUP	CNVnator	C	C	C	C	D	C
	DELLY	C	C	C	C	D	I
	forestSV	C	C	C	C	C	C
	inGAP-sv	C	C	C	C	I	I
	Lumpy	C	C	C	C	D	I
	Manta	C	C	C	C	D	I
	Meerkat	C	C	C	C	D	I
	MetaSV	I	R-125	C	C	D	I

	Pindel	I	R-125	C	C	I	I
	RAPTR	C	C	C	C	D	I
	SoftSV	I	C	C	C	D	I
	Ulysses	C	I	C	D	D	U
INS	BASIL-ANISE	I	D	C	I	D	I
	BreakDancer	C	C	I	C	D	I
	inGAP-sv	D	I	I	D	I	I
	Manta	C	C	C	C	D	I
	PopIns	C	D	C	I	U	I
	SVseq2	I	R-125	I	I	D	I
INV	1-2-3-SV	I	C	D	D	D	I
	BreakDancer	C	C	C	I	D	I
	DELLY	C	C	C	D	D	I
	inGAP-sv	C	I	C	D	U	I
	Lumpy	C	C	C	C	D	I
	Manta	C	I	C	C	D	U
	Meerkat	C	C	C	C	C	I
	MetaSV	C	R-125	C	C	C	I
	Pindel	R-125	R-125	D	C	D	I
	PRISM	C	D	C	C	D	I
	SoftSV	C	C	C	I	D	I
	Ulysses	D	I	I-500	C	D	D

I: Increased precision or recall with increased read length, insert size or coverage.

D: Decreased precision or recall with increased read length, insert size or coverage.

C: Constant precision or recall with increased read length, insert size or coverage.

U: Unclear tendency.

Others: the highest precision or recall at the category of the indicated values.

The classification of the precision (recall) tendency into I, D, and C is based on > 5-pont differences between the categories (e.g., R-100, R-125, and R-150) at multiple points of the minimum number of supporting reads.

Table S13. Mean coefficient of variation of SV detection accuracy for each category of SV type and read property

SV type	Read library	Precision CV	Recall CV
DEL	RL	0.01	0.07
	IS	0.01	0.03
	RC (Sim-A)	0.03	0.1
	RC (Real data1)	0.09	0.21
DUP	RL	0.04	0.07
	IS	0.03	0.04
	RC (Sim-A)	0.07	0.07
	RC (Real data1)	0.2	0.19
INS	RL	0.07	0.2
	IS	0.07	0.09
	RC (Sim-A)	0.24	0.47
	RC (Real data1)	0.16	0.45
INV	RL	0.02	0.09
	IS	0.02	0.03
	RC (Sim-A)	0.02	0.04
	RC (Real data1)	0.27	0.22

Mean values of coefficient of variation (CV) for each category are indicated. CVs were calculated by dividing the standard deviation of recall or precision percentage with the corresponding mean value.

RL: Read length (100 bp, 125 bp, 150 bp).

IS: Insert size (400 bp, 500 bp, 600 bp).

RC: Read coverage (10×, 20×, 30×, 60×).

Table S15. Genotyping precision of SV detection algorithms

Algorithms	Type	TP *1	FP *2	UnGT *3	Precision1 *4	Precision2 *5
AS-GENSENG	DEL	180 (13)	4 (0)	0 (0)	97.8 (100)	97.8 (100)
	DUP	302	31	0	90.6	90.6
BICseq2	DEL	1251 (142)	82 (4)	0 (0)	93.8 (97.3)	93.8 (97.3)
	DUP	1054	129	0	89.1	89.1
BreakSeq2	DEL	68 (94)	17 (4)	0 (0)	79.7 (94.8)	79.7 (94.8)
CNVnator	DEL	779 (115)	34 (4)	0 (0)	95.8 (97.8)	95.8 (97.8)
	DUP	842	68	46	88.1	92.5
Control-FREEC	DEL	59 (7)	6 (1)	0 (0)	90.7 (87.5)	90.7 (87.5)
	DUP	223	83	8	71.1	72.9
DELLY	DEL	1715 (138)	108 (7)	3 (3)	93.9 (93.2)	94.1 (95.2)
	DUP	840	562	14	59.3	59.9
	INV	225	32	1	87.2	87.8
DIGTYPER	DUP	885	424	159	60.3	67.6
	INV	214	31	28	78.4	87.3
ERDS	DEL	1931 (121)	67 (2)	15 (1)	95.9 (97.6)	96.6 (98.4)
	DUP	611	58	0	90.5	90.5
GenomeSTRiP	DEL	542 (43)	67 (2)	764 (98)	39.5 (30.1)	89.0 (95.5)
Lumpy *6	DEL	2246	200	1	91.8	91.9
	DUP	1128	210	0	84.3	84.3
	INV	192	26	3	86.8	88.0
Manta	DEL	2393 (133)	158 (7)	0 (0)	93.8 (95.0)	93.8 (95.0)
	DUP	823	555	0	59.7	59.7
	INS	307	7	0	97.7	97.7
	INV	190	26	0	87.9	87.9
MELT	INS (MEI)	367	103	0	78.0	78.0
Pindel	DEL	2413 (2)	141 (0)	199 (112)	87.6 (1.7)	94.4 (100)
	DUP	543	534	215	42.0	50.4
	INV	225	36	0	86.2	86.2
PopIns	INS	174	25	118	55.0	87.5

PBHoney	DEL	194 (0)	27 (0)	1 (0)	87.4 (0)	87.8 (0)
	INS	175	16	0	91.6	91.6
PennCNV-Seq	DEL	1059 (155)	46 (3)	0 (0)	95.8 (98.1)	95.8 (98.1)
	DUP	1048	75	0	93.3	93.3
readDepth	DEL	264 (48)	17 (2)	0 (0)	93.9 (96.0)	93.9 (96.0)
	DUP	823	54	0	93.8	93.8
RetroSeq	INS (MEI)	316	137	0	69.7	69.7
Sniffles	DEL	2087 (47)	644 (22)	242 (0)	70.2 (68.1)	76.4 (68.1)
	DUP	346	489	479	26.3	41.4
	INS	649	256	50	68.0	71.7
	INV	169	64	6	70.7	72.5
SV^2	DEL	2202 (133)	89 (3)	641 (4)	75.1 (95.0)	96.1 (97.8)
	DUP	692	511	180	50.0	57.5
SVelter	DEL	1671 (98)	147 (4)	0 (0)	91.9 (96.1)	91.9 (96.1)
	INV	129	33	0	79.6	79.6
Tangram	INS (MEI)	110	27	8	75.8	80.2

[*1] TP: Number of correctly called genotypes.

[*2] FP: Number of wrongly called genotypes.

[*3] UnGT: Number of true positive calls with undefined genotype.

[*4] Precision1: Percentage of correctly called genotypes in true positive calls (i.e., TP/(TP + FP + UnGT) × 100.

[*5] Precision2: Percentage of correctly called genotypes in true positive calls with genotyped information (i.e., TP/(TP + FP) × 100.

[*6] Genotyping of Lumpy calls was conducted using SVTyper.

MELT, RetroSeq, and Tangram were run with the Sim-MEI data, and the other algorithms indicated were run with the Sim-A and real (NA12878) datasets. We selected an RSS filtering threshold at which the sum of the prec1 and prec2 values gave the highest value for each category.

Results for the real data were indicated within parentheses.

Table S19. Extended list of algorithms providing good SV calling results for both the simulated and the NA12878 real datasets

SV type	Size	Algorithms	Simulated data		Real data		RMSE of BP*1	Run time*2	nF *3
			Precision	Recall	Precision	Recall			
DEL	All	GRIDSS	98.9	86.6	87.6	28.9	16	17	3.56
		Lumpy	99.1	81.4	87.1	26.1	77	4	3.41
		SVseq2	96.2	86.1	75.7	24.9	13	34	3.28
		SoftSV	96.8	83.6	80.2	23.2	169	44	3.25
		Manta	95.9	83.1	74.2	24.3	77	63	3.22
		MATCHCLIP	99.4	71.7	91.6	20.9	83	6.5	3.12
		inGAP-sv	91.1	78.6	78.3	22.5	19	8	3.11
		FermiKit	99.4	67.6	88.4	22.1	87	218	3.10
		Sniffles	95.6	74.9	72.9	20.6	644	1.5	2.98
		Wham	94.1	71.7	84.2	17.3	38	4	2.87
		pbsv	88.8	54.6	67.2	27.9	25	9.5	2.84
		Pindel	86.7	75.3	60.6	20.5	181	44	2.79
		Meerkat	93.9	70.6	82.9	15.7	714	11	2.77
		Sprites	87.3	89.5	33.3	35.9	167	16	2.73
		SvABA	99.5	58.7	95.2	15.3	18	23	2.68
		DELLY	98.8	57.7	77.2	15.1	23	34	2.56
	SS	pbsv	52.3	80.1	48.2	44.0	N.D.	9.5	8.15
		GRIDSS	82.4	62.6	45.5	33.2	N.D.	17	7.90
		Sniffles	75.4	64.2	53.4	29.5	N.D.	1.5	7.78
		PBHoney-NGM	76.2	62.6	50.2	27.6	N.D.	8.5	7.51
		SVseq2	68.1	66.6	42.9	30.9	N.D.	34	7.43
		Manta	70.0	55.5	47.3	32.6	N.D.	63	7.38
		Sprites	49.7	73.0	30.3	49.5	N.D.	16	7.05
		PRISM	60.0	52.3	44.4	28.7	N.D.	53	6.67
	S	GRIDSS	96.5	89.0	92.1	32.1	18	17	4.17
		pbsv	92.5	85.1	78.7	32.7	16	9.5	3.96
		Manta	94.6	79.5	81.6	30.5	7	63	3.87
		SVseq2	94.8	84.8	82.3	27.6	9	34	3.84
		Lumpy	99.3	76.4	90.5	26.8	18	4	3.81
		Sniffles	96.0	85.9	81.5	25.7	26	1.5	3.78

		SoftSV	97.8	79.9	81.5	24.9	8	44	3.72
		FermiKit	99.2	67.7	89.2	27.3	10	218	3.69
		PBHoney-NGM	92.3	84	75.2	24.7	33	8.5	3.62
		Pindel	91.1	81.5	73.7	26.1	5	196	3.62
		MATCHCLIP	99.6	67.8	95.5	22.1	5	6.5	3.50
		inGAP-sv	84.9	75.2	74.1	23.6	21	8	3.39
		CLEVER	87.3	82.5	61.3	22.0	31	47	3.32
	M	Lumpy	99.5	91.6	94.5	26.8	117	4	3.03
		DELLY	99.9	91.9	93.8	26.6	26	34	3.03
		inGAP-sv	99.9	90.0	95.9	26.6	17	8	3.03
		GRIDSS	99.5	92.3	94.2	26.4	13	18	3.03
		Manta	99.7	90.6	93.5	26.2	121	63	3.00
		GenomeSTRiP	99.8	90.2	90.2	24.9	28	25	2.94
		Ulysses	98.8	90.6	96.0	22.1	273	11	2.89
		SoftSV	97.9	88.8	93.6	22.1	268	44	2.85
		SvABA	99.8	77.6	96.0	24.1	22	23	2.84
		MATCHCLIP	99.4	82.0	98.5	22.2	127	6.5	2.84
		Wham	98.3	85.0	93.5	20.8	52	4	2.78
		TIDDIT	99.4	76.7	92.2	23.2	88	3.5	2.77
		Meerkat	94.1	86.9	88.8	21.1	122	11	2.74
		SVseq2	97.6	90.5	82.2	20.3	18	34	2.73
		ERDS	95.6	80.8	86.5	22.7	128	25	2.73
	L	TIDDIT	98.6	84.4	82.8	12.0	81	3.5	3.75
		ERDS	96.3	78.4	95.0	9.1	38	25	3.37
		forestSV	85.3	73.0	65.4	11.4	3883	33	3.29
		CNVnator	92.0	82.6	37.7	15.6	1500	1	3.19
		Manta	97.3	86.8	32.6	9.1	5	63	3.14
		PennCNV-Seq	89.8	74.8	25.0	10.7	10756	72	2.84
		SvABA	98.6	85.6	35.6	7.6	18	23	3.06
		Meerkat	90.6	81.4	31.8	7.1	2991	11	2.83
		Lumpy	97.2	86.2	27.2	9.5	19	4	3.00
		MetaSV	98.5	83.2	57.7	6.0	69	1[*5]	2.74
		MATCHCLIP	97.4	69.4	74.9	6.2	5	6.5	2.71
		inGAP-sv	99.3	88.6	27.0	9.1	17	8	2.69
		BICseq2	62.0	41.9	64.2	10.1	14893	7.7	2.67

		OncoSNP-Seq	95.3	37.7	47.4	12.0	3830	63	2.67
		SoftSV	92.7	76.0	35.9	8.0	2	44	2.63
DUP	All	Wham	96.9	81.7	57.1	10.2	20	4	3.92
		SoftSV	84.2	67.8	47.3	14.3	181	44	3.91
		MATCHCLIP	87.6	77.5	58.0	9.9	427	6.5	3.79
		GRIDSS	91.1	77.9	58.4	9.6	15	17	3.78
		Manta	99.0	83.2	49.4	7.1	309	63	3.35
		SvABA	94.8	76.0	40.4	6.5	40	23	3.02
		MetaSV	82.6	69.6	42.7	7.2	2531	1*5	3.02
		Pindel	86.3	71.1	31.3	5.2	25	196	2.57
		Lumpy	96.1	81.1	25.6	3.5	35	4	2.38
		DELLY	97.4	82.7	15.0	2.9	22	6	2.14
	S	SvABA	73.3	59.2	42.1	9.1	1	23	4.54
		Manta	98.2	66.2	27.2	7.7	5	63	4.44
		Lumpy	76.0	62.8	41.2	7.9	2	4	4.43
		Pindel	67.6	55.4	38.7	9.5	1	196	4.37
		Sniffles	94.4	61.4	38.7	9.5	9	1.5	4.21
		SoftSV	47.5	59.2	26.0	15.8	1	44	4.21
		Wham	81.0	66.8	26.0	7.4	1	4	4.17
	M	MetaSV	89.5	68.6	37.5	3.3	338	1*5	3.68
		CNVnator	91.2	54.0	43.7	2.8	732	1	3.38
		forestSV	39.9	33.3	37.0	2.9	995	33	2.82
		ERDS	27.6	22.2	14.7	11.9	2216	25	2.67
		readDepth	52.2	51.3	31.9	2.1	702	3.5	2.53
		DELLY	98.9	88.7	16.3	1.4	11	34	2.52
		iCopyDAV	56.9	45.6	19.3	2.8	1357	8.2	2.50
		GRIDSS	98.8	87.5	17.3	1.2	15	17	2.43
		Manta	99.4	83.1	19.5	1.0	6	63	2.30
		Lumpy	99.0	83.1	15.1	1.1	21	4	2.29
		Ulysses	83.1	78.4	9.9	2.0	37	11	2.29
	L	CNVnator	91.5	85.8	48.6	13.8	3812	1	3.79
		ERDS	88.1	72.4	50.8	12.8	8482	25	3.63
		iCopyDAV	85.9	70.7	67.1	8.7	4701	8.2	3.35
		MetaSV	94.5	84.3	35.6	13.2	4289	1*5	3.33
		BICseq2	85.3	75.6	49.5	8.4	8541	7.7	3.09

		forestSV	89.5	36.5	59.7	7.3	3492	33	2.67
		readDepth	78.3	69.7	27.6	9.0	8051	3.5	2.56
		inGAP-sv	90.7	33.6	66.4	5.1	1550	8	2.23
		Pindel	98.6	88.0	12.6	3.7	1	196	1.92
		PennCNV-Seq	85.6	76.2	28.0	3.0	8408	72	1.92
INS	Unspecified INS	pbsv	89.7	38.2	72.7	27.5	6	9.5	6.68
		inGAP-sv	99.7	58.5	85.5	11.8	22	8	6.27
		Sniffles	74.8	52.5	65.9	9.0	11	1.5	5.08
		SVseq2	70.4	64.2	38.5	7.1	4	34	4.84
		PBHoney-NGM	74.2	27.8	78.8	11.5	141	8.5	4.43
		BASIL-ANISE	60.3	46.0	45.5	8.6	13	4	4.34
		Manta	96.5	11.9	79.1	8.6	1	63	2.94
	MEI	MELT	99.7	68.9	88.9	(85.6)[*4]	6	8	3.21
		Mobster	100	67.1	88.3	(71.9)[*4]	9	4.5	3.04
	NUMT	MELT-numt	100	20.0	92.3	(33) [*4]	10	8	2.57
	VEI	Mobster-vei	98.0	100	25.2	(60) [*4]	1	4.5	1.96
INV	ALL	DELLY	94.7	81.8	38.9	15.6	15	34	3.07
		TIDDIT	89.2	77.9	49.1	11.7	145	3.5	2.89
		1-2-3-SV	70.7	81.2	31.8	14.8	254	32	2.67
		GRIDSS	96.6	84.7	34.2	10.4	30	17	2.67
		Manta	97.6	80.9	27.5	12.8	6	63	2.65
		Pindel	89.9	78.3	29.3	11.4	2256	196	2.55
		inGAP-sv	99.0	69.2	31.8	10.0	23	8	2.47
		PRISM	96.5	81.2	34.3	8.3	1937	53	2.47

[*1] Root mean square error of breakpoints determined by the algorithms for the Sim-A data.

[*2] Time (minute) spent on a run with 30× read data (10× for long reads) for chromosome 8 using a single CPU.

[*3] Sum of normalized F-measures of the simulated and the real data. Normalized F-measure = F-mesure/the mean F-measure for the corresponding category.

[*4] Provisional recall value: the number of true positives was calculated by dividing by the provisional number of reference MEIs, NUMTs, and VEIs (1,350, 15, and 10, respectively) which were estimated with the data from the 1000 Genome project.

[*5] Run time without the time spent on Pindel, BreakDancer, CNVnator, and BreakSeq2.

Table S20. List of poor performing algorithms that cannot be recommended for SV detection with human WGS data

Algorithms	Comments
Breakway	Run time is extremely long.
indelMINER	Precision and recall are low. This algorithm can detect only DELs.
MindTheGap	Precision and recall are low despite of long runtime and high memory consumption.
VirusSeq	Precision and recall are low despite of long runtime and high memory consumption.
AS-GENSENG, OncoSNP-Seq	Their precision and recall are low. However, these algorithms may more effectively and accurately detect somatic CNVs or germline CNVs from whole exome sequencing data. The use of CNVnator, PennCNV-Seq, or readDepth is recommended if read depth-based algorithms are selected.

References

1. Wang W, Wang W, Sun W, Crowley JJ, Szatkiewicz JP. Allele-specific copy-number discovery from whole-genome and whole-exome sequencing. Nucleic Acids Res. 2015;43:e90.
2. Holtgrewe M, Kuchenbecker L, Reinert K. Methods for the detection and assembly of novel sequence in high-throughput sequencing data. Bioinformatics. 2015;31:1904-12.
3. Tennakoon C, Sung WK. BATVI: Fast, sensitive and accurate detection of virus integrations. BMC Bioinformatics. 2017;18:71.
4. Xi R, Lee S, Xia Y, Kim TM, Park PJ. Copy number analysis of whole-genome data using BIC-seq2 and its application to detection of cancer susceptibility variants. Nucleic Acids Res. 2016;44:6274-86.
5. Chen K, Wallis JW, McLellan MD, Larson DE, Kalicki JM, Pohl CS, et al. BreakDancer: an algorithm for high-resolution mapping of genomic structural variation. Nat Methods. 2009;6:677-81.
6. Zhao H, Zhao F. BreakSeek: a breakpoint-based algorithm for full spectral range INDEL detection. Nucleic Acids Res. 2015;43:6701-13.
7. Lam HY, Mu XJ, Stutz AM, Tanzer A, Cayting PD, Snyder M, et al. Nucleotide-resolution analysis of structural variants using BreakSeq and a breakpoint library. Nat Biotechnol. 2010;28:47-55.
8. Clark MJ, Homer N, O'Connor BD, Chen Z, Eskin A, Lee H, et al. U87MG decoded: the genomic sequence of a cytogenetically aberrant human cancer cell line. PLoS Genet. 2010;6:e1000832.
9. Marschall T, Costa IG, Canzar S, Bauer M, Klau GW, Schliep A, et al. CLEVER: clique-enumerating variant finder. Bioinformatics. 2012;28:2875-82.
10. Abyzov A, Urban AE, Snyder M, Gerstein M. CNVnator: an approach to discover, genotype, and characterize typical and atypical CNVs from family and population genome sequencing. Genome Res. 2011;21:974-84.
11. Boeva V, Popova T, Bleakley K, Chiche P, Cappo J, Schleiermacher G, et al. Control-FREEC: a tool for assessing copy number and allelic

content using next-generation sequencing data. Bioinformatics. 2012;28:423-5.
12. Wang J, Mullighan CG, Easton J, Roberts S, Heatley SL, Ma J, et al. CREST maps somatic structural variation in cancer genomes with base-pair resolution. Nat Methods. 2011;8:652-4.
13. Rausch T, Zichner T, Schlattl A, Stutz AM, Benes V, Korbel JO. DELLY: structural variant discovery by integrated paired-end and split-read analysis. Bioinformatics. 2012;28:i333-i9.
14. Ebler J, Schonhuth A, Marschall T. Genotyping inversions and tandem duplications. Bioinformatics. 2017;33:4015-23.
15. Dayama G, Emery SB, Kidd JM, Mills RE. The genomic landscape of polymorphic human nuclear mitochondrial insertions. Nucleic Acids Res. 2014;42:12640-9.
16. Zhu M, Need AC, Han Y, Ge D, Maia JM, Zhu Q, et al. Using ERDS to infer copy-number variants in high-coverage genomes. Am J Hum Genet. 2012;91:408-21.
17. Li H. FermiKit: assembly-based variant calling for Illumina resequencing data. Bioinformatics. 2015;31:3694-6.
18. Michaelson JJ, Sebat J. forestSV: structural variant discovery through statistical learning. Nat Methods. 2012;9:819-21.
19. Sindi SS, Onal S, Peng LC, Wu HT, Raphael BJ. An integrative probabilistic model for identification of structural variation in sequencing data. Genome Biol. 2012;13:R22.
20. Handsaker RE, Korn JM, Nemesh J, McCarroll SA. Discovery and genotyping of genome structural polymorphism by sequencing on a population scale. Nat Genet. 2011;43:269-76.
21. Cameron DL, Schroder J, Penington JS, Do H, Molania R, Dobrovic A, et al. GRIDSS: sensitive and specific genomic rearrangement detection using positional de Bruijn graph assembly. Genome Res. 2017;27:2050-60.
22. Baheti S, Tang X, O'Brien DR, Chia N, Roberts LR, Nelson H, et al. HGT-ID: an efficient and sensitive workflow to detect human-viral insertion sites using next-generation sequencing data. BMC

Bioinformatics. 2018;19:271.
23. Quinlan AR, Clark RA, Sokolova S, Leibowitz ML, Zhang Y, Hurles ME, et al. Genome-wide mapping and assembly of structural variant breakpoints in the mouse genome. Genome Res. 2010;20:623-35.
24. Dharanipragada P, Vogeti S, Parekh N. iCopyDAV: Integrated platform for copy number variations-Detection, annotation and visualization. PLoS One. 2018;13:e0195334.
25. Ratan A, Olson TL, Loughran TP, Jr., Miller W. Identification of indels in next-generation sequencing data. BMC Bioinformatics. 2015;16:42.
26. Qi J, Zhao F. inGAP-sv: a novel scheme to identify and visualize structural variation from paired end mapping data. Nucleic Acids Res. 2011;39:W567-75.
27. Jiang C, Chen C, Huang Z, Liu R, Verdier J. ITIS, a bioinformatics tool for accurate identification of transposon insertion sites using next-generation sequencing data. BMC Bioinformatics. 2015;16:72.
28. Zhuang J, Weng Z. Local sequence assembly reveals a high-resolution profile of somatic structural variations in 97 cancer genomes. Nucleic Acids Res. 2015;43:8146-56.
29. Layer RM, Chiang C, Quinlan AR, Hall IM. LUMPY: a probabilistic framework for structural variant discovery. Genome Biol. 2014;15:R84.
30. Chen X, Schulz-Trieglaff O, Shaw R, Barnes B, Schlesinger F, Kallberg M, et al. Manta: rapid detection of structural variants and indels for germline and cancer sequencing applications. Bioinformatics. 2016;32:1220-2.
31. Wu Y, Tian L, Pirastu M, Stambolian D, Li H. MATCHCLIP: locate precise breakpoints for copy number variation using CIGAR string by matching soft clipped reads. Front Genet. 2013;4:157.
32. Yang L, Luquette LJ, Gehlenborg N, Xi R, Haseley PS, Hsieh CH, et al. Diverse mechanisms of somatic structural variations in human cancer genomes. Cell. 2013;153:919-29.
33. Gardner EJ, Lam VK, Harris DN, Chuang NT, Scott EC, Pittard WS, et al. The Mobile Element Locator Tool (MELT): population-scale

mobile element discovery and biology. Genome Res. 2017.

34. Mohiyuddin M, Mu JC, Li J, Bani Asadi N, Gerstein MB, Abyzov A, et al. MetaSV: an accurate and integrative structural-variant caller for next generation sequencing. Bioinformatics. 2015;31:2741-4.

35. Rizk G, Gouin A, Chikhi R, Lemaitre C. MindTheGap: integrated detection and assembly of short and long insertions. Bioinformatics. 2014;30:3451-7.

36. Thung DT, de Ligt J, Vissers LE, Steehouwer M, Kroon M, de Vries P, et al. Mobster: accurate detection of mobile element insertions in next generation sequencing data. Genome Biol. 2014;15:488.

37. Yau C. OncoSNP-SEQ: a statistical approach for the identification of somatic copy number alterations from next-generation sequencing of cancer genomes. Bioinformatics. 2013;29:2482-4.

38. Kavak P, Lin YY, Numanagic I, Asghari H, Gungor T, Alkan C, et al. Discovery and genotyping of novel sequence insertions in many sequenced individuals. Bioinformatics. 2017;33:i161-i9.

39. English AC, Salerno WJ, Reid JG. PBHoney: identifying genomic variants via long-read discordance and interrupted mapping. BMC Bioinformatics. 2014;15:180.

40. de Araujo Lima L, Wang K. PennCNV in whole-genome sequencing data. BMC Bioinformatics. 2017;18:383.

41. Ye K, Schulz MH, Long Q, Apweiler R, Ning Z. Pindel: a pattern growth approach to detect break points of large deletions and medium sized insertions from paired-end short reads. Bioinformatics. 2009;25:2865-71.

42. Kehr B, Melsted P, Halldorsson BV. PopIns: population-scale detection of novel sequence insertions. Bioinformatics. 2016;32:961-7.

43. Jiang Y, Wang Y, Brudno M. PRISM: pair-read informed split-read mapping for base-pair level detection of insertion, deletion and structural variants. Bioinformatics. 2012;28:2576-83.

44. Bickhart DM, Hutchison JL, Xu L, Schnabel RD, Taylor JF, Reecy JM, et al. RAPTR-SV: a hybrid method for the detection of structural variants. Bioinformatics. 2015;31:2084-90.

45. Miller CA, Hampton O, Coarfa C, Milosavljevic A. ReadDepth: a parallel R package for detecting copy number alterations from short sequencing reads. PLoS One. 2011;6:e16327.
46. Keane TM, Wong K, Adams DJ. RetroSeq: transposable element discovery from next-generation sequencing data. Bioinformatics. 2013;29:389-90.
47. Sedlazeck FJ, Rescheneder P, Smolka M, Fang H, Nattestad M, von Haeseler A, et al. Accurate detection of complex structural variations using single-molecule sequencing. Nat Methods. 2018.
48. Schroder J, Hsu A, Boyle SE, Macintyre G, Cmero M, Tothill RW, et al. Socrates: identification of genomic rearrangements in tumour genomes by re-aligning soft clipped reads. Bioinformatics. 2014.
49. Hart SN, Sarangi V, Moore R, Baheti S, Bhavsar JD, Couch FJ, et al. SoftSearch: integration of multiple sequence features to identify breakpoints of structural variations. PLoS One. 2013;8:e83356.
50. Bartenhagen C, Dugas M. Robust and exact structural variation detection with paired-end and soft-clipped alignments: SoftSV compared with eight algorithms. Brief Bioinform. 2016;17:51-62.
51. Kim J, Kim S, Nam H, Kim S, Lee D. SoloDel: a probabilistic model for detecting low-frequent somatic deletions from unmatched sequencing data. Bioinformatics. 2015;31:3105-13.
52. Zhang Z, Wang J, Luo J, Ding X, Zhong J, Wang J, et al. Sprites: detection of deletions from sequencing data by re-aligning split reads. Bioinformatics. 2016;32:1788-96.
53. Antaki D, Brandler WM, Sebat J. SV2: Accurate Structural Variation Genotyping and De Novo Mutation Detection from Whole Genomes. Bioinformatics. 2017.
54. Wala JA, Bandopadhayay P, Greenwald NF, O'Rourke R, Sharpe T, Stewart C, et al. SvABA: genome-wide detection of structural variants and indels by local assembly. Genome Res. 2018;28:581-91.
55. Zeitouni B, Boeva V, Janoueix-Lerosey I, Loeillet S, Legoix-ne P, Nicolas A, et al. SVDetect: a tool to identify genomic structural variations from paired-end and mate-pair sequencing data.

Bioinformatics. 2010;26:1895-6.
56. Zhao X, Emery SB, Myers B, Kidd JM, Mills RE. Resolving complex structural genomic rearrangements using a randomized approach. Genome Biol. 2016;17:126.
57. Yang R, Chen L, Newman S, Gandhi K, Doho G, Moreno CS, et al. Integrated analysis of whole-genome paired-end and mate-pair sequencing data for identifying genomic structural variations in multiple myeloma. Cancer Inform. 2014;13:49-53.
58. Zhang J, Wang J, Wu Y. An improved approach for accurate and efficient calling of structural variations with low-coverage sequence data. BMC Bioinformatics. 2012;13 Suppl 6:S6.
59. Wu J, Lee WP, Ward A, Walker JA, Konkel MK, Batzer MA, et al. Tangram: a comprehensive toolbox for mobile element insertion detection. BMC Genomics. 2014;15:795.
60. Lee E, Iskow R, Yang L, Gokcumen O, Haseley P, Luquette LJ, 3rd, et al. Landscape of somatic retrotransposition in human cancers. Science. 2012;337:967-71.
61. Zhuang J, Wang J, Theurkauf W, Weng Z. TEMP: a computational method for analyzing transposable element polymorphism in populations. Nucleic Acids Res. 2014;42:6826-38.
62. Eisfeldt J, Vezzi F, Olason P, Nilsson D, Lindstrand A. TIDDIT, an efficient and comprehensive structural variant caller for massive parallel sequencing data. F1000Res. 2017;6:664.
63. Gillet-Markowska A, Richard H, Fischer G, Lafontaine I. Ulysses: accurate detection of low-frequency structural variations in large insert-size sequencing libraries. Bioinformatics. 2015;31:801-8.
64. Hormozdiari F, Alkan C, Eichler EE, Sahinalp SC. Combinatorial algorithms for structural variation detection in high-throughput sequenced genomes. Genome Res. 2009;19:1270-8.
65. Wang Q, Jia P, Zhao Z. VERSE: a novel approach to detect virus integration in host genomes through reference genome customization. Genome Med. 2015;7:2.
66. Chen Y, Yao H, Thompson EJ, Tannir NM, Weinstein JN, Su X.

VirusSeq: software to identify viruses and their integration sites using next-generation sequencing of human cancer tissue. Bioinformatics. 2013;29:266-7.

67. Kronenberg ZN, Osborne EJ, Cone KR, Kennedy BJ, Domyan ET, Shapiro MD, et al. Wham: Identifying Structural Variants of Biological Consequence. PLoS Comput Biol. 2015;11:e1004572.

68. David M, Mustafa H, Brudno M. Detecting Alu insertions from high-throughput sequencing data. Nucleic Acids Res. 2013;41:e169.

69. Trappe K, Emde AK, Ehrlich HC, Reinert K. Gustaf: Detecting and correctly classifying SVs in the NGS twilight zone. Bioinformatics. 2014;30:3484-90.

70. Linheiro RS, Bergman CM. Whole genome resequencing reveals natural target site preferences of transposable elements in Drosophila melanogaster. PLoS One. 2012;7:e30008.

71. Korbel JO, Abyzov A, Mu XJ, Carriero N, Cayting P, Zhang Z, et al. PEMer: a computational framework with simulation-based error models for inferring genomic structural variants from massive paired-end sequencing data. Genome Biol. 2009;10:R23.

72. Escaramis G, Tornador C, Bassaganyas L, Rabionet R, Tubio JM, Martinez-Fundichely A, et al. PeSV-Fisher: identification of somatic and non-somatic structural variants using next generation sequencing data. PLoS One. 2013;8:e63377.

73. Robb SM, Lu L, Valencia E, Burnette JM, 3rd, Okumoto Y, Wessler SR, et al. The use of RelocaTE and unassembled short reads to produce high-resolution snapshots of transposable element generated diversity in rice. G3 (Bethesda). 2013;3:949-57.

74. Yoon S, Xuan Z, Makarov V, Ye K, Sebat J. Sensitive and accurate detection of copy number variants using read depth of coverage. Genome Res. 2009;19:1586-92.

75. Platzer A, Nizhynska V, Long Q. TE-Locate: A Tool to Locate and Group Transposable Element Occurrences Using Paired-End Next-Generation Sequencing Data. Biology (Basel). 2012;1:395-410.

76. Gilly A, Etcheverry M, Madoui MA, Guy J, Quadrana L, Alberti A, et

al. TE-Tracker: systematic identification of transposition events through whole-genome resequencing. BMC Bioinformatics. 2014;15:377.

77. Sudmant PH, Rausch T, Gardner EJ, Handsaker RE, Abyzov A, Huddleston J, et al. An integrated map of structural variation in 2,504 human genomes. Nature. 2015;526:75-81.
