# Supplementary material for: Comprehensive evaluation of structural variation detection algorithms for whole genome sequencing
Source: Genome Biol. 2019 Jun 3;20:117. doi: 10.1186/s13059-019-1720-5 (PMC6547561; doi:10.1186/s13059-019-1720-5)
Supplement: Supplementary file 2 — Figure S13. Effect of read length, read coverage, and insert size on recall and precision for various SV algorithms. (PDF 2174 kb) [file 13059_2019_1720_MOESM2_ESM.pdf]

Figure S13. Effect of read length, read coverage, and insert size on recall and precision for various SV detection algorithms.

S13-A: INSs called with BASIL-ANISE; **S13-B**: DELs called with BreakDancer; **S13-C**: INSs called with BreakDancer; **S13-D**: INVs called with BreakDancer; **S13-E**: DELs called with CLEVER; **S13-F**: DELs called with CNVnator; **S13-G**: DUPs called with CNVnator; **S13-H**: DELs called with DELLY; **S13-I**: DUPs called with DELLY; **S13-J:** INVs called with DELLY; **S13-K**: DELs called with forestSV; **S13-L**: DUPs called with forestSV; **S13-M**: DELs called with inGAP-sv; **S13-N**: DUPs called with inGAP-sv; **S13-O**: INSs called with inGAP-sv; **S13-P**: INVs called with inGAP-sv; **S13-Q**: DELs called with Lumpy; **S13-R**: DUPs called with Lumpy; **S13-S**: INVs called with Lumpy; **S13-T**: DELs called with Manta; **S13-U**: DUPs called with Manta; **S13-V**: INSs called with Manta; **S13-W**: INVs called with Manta; **S13-X**: DELs called with Meerkat; **S13-Y**: DUPs called with Meerkat; **S13-Z**: INVs called with Meerkat; **S13-AA**: DELs called with MetaSV; **S13-AB**: DUPs called with MetaSV; **S13-AC**: INVs called with MetaSV; **S13-AD**: DELs called with Pindel; **S13-AE**: DUPs called with Pindel; **S13-AF**: INVs called with Pindel; **S13-AG**: INSs called with PopIns; **S13-AH**: DELs called with PRISM; **S13-AI**: INVs called with PRISM; **S13-AJ**: DELs called with RAPTR; **S13-AK**: DUPs called with RAPTR; **S13-AL**: DELs called with Socrates; **S13-AM**: DELs called with SoftSV; **S13-AN**: DUPs called with SoftSV; **S13-AO**: INVs called with SoftSV; **S13-AP**: DELs called with SVseq2; **S13-AQ**: INSs called with SVseq2; **S13-AR**: DELs called with Ulysses; **S13-AS**: DUPs called with Ulysses; **S13-AT**: INVs called with Ulysses; **S13-AU**: DELs called with 1-2-3-SV; **S13-AV**: INVs called with 1-2-3-SV.

DELs, DUPs, INSs or INVs were called using the indicated algorithms with data with different scales of read length, insert size, or read coverage. The call set was filtered with the minimum number of reads supporting the called SVs, indicated with the scales on the x-axis. A respective set of the filtered SVs was used for measuring the recall and precision values, indicated with the left or the right scales on the y-axis. Recall and precision percentages are represented with blue and red symbols/lines, respectively. (**a**) The results of the simulated data with different read lengths (100 bp (R-100), 125 bp (R-125), and 150 bp (R-150)) are indicated with circles, triangles, and asterisks, respectively. (**b**) The results of the simulated data with different insert sizes (400 bp (I-400), 500 bp (I-500), and 600 bp (I-600)) are indicated with circles, triangles, and asterisks, respectively. (**c**) The results of the simulated data with different read coverage (10x, 20x, 30x, and 60x) are indicated with circles, triangles, asterisks, and squares, respectively. (**d**) Results of the real data with different read coverage. Note that the scales for recall and precision in (c) and (d) are different.

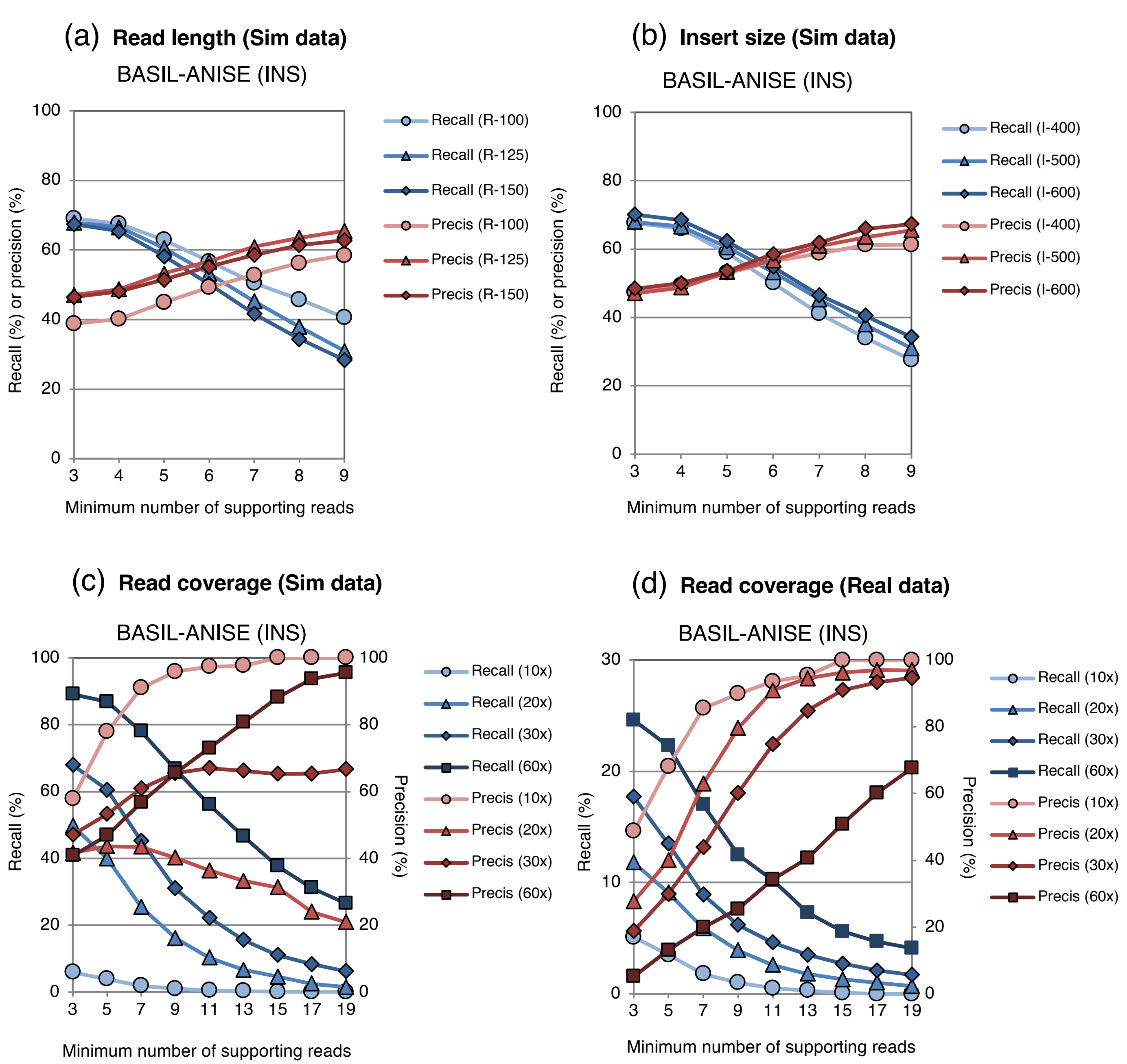

Figure S13-A. Results for INS calling using BASIL-ANISE. INSs were called using BASIL-ANISE with data with different scales of read length, insert size, or read coverage. The call set was filtered with the minimum number of reads supporting the called SVs, indicated with the scales on the x-axis. The respective set of the filtered SVs was used for measuring the recall and precision values, which are indicated with the left or the right scales on the y-axis. Recall and precision percentages are represented with blue and red symbols/lines, respectively. (**a**) The results of the simulated data with different read lengths, 100 bp (R-100), 125 bp (R-125), and 150 bp (R-150), are indicated with circles, triangles, and asterisks, respectively. (**b**) The results of the simulated data with different insert sizes, 400 bp (I-400), 500 bp (I-500), and 600 bp (I-600), are indicated with circles, triangles, and asterisks, respectively. (**c**) The results of the simulated data with different read coverage, 10x, 20x, 30x, and 60x, are indicated with circles, triangles, asterisks, and squares, respectively. (**d**) The results of the real data with different read coverage. It should be noted that the scales of the recall and precision in (c) and (d) are different.

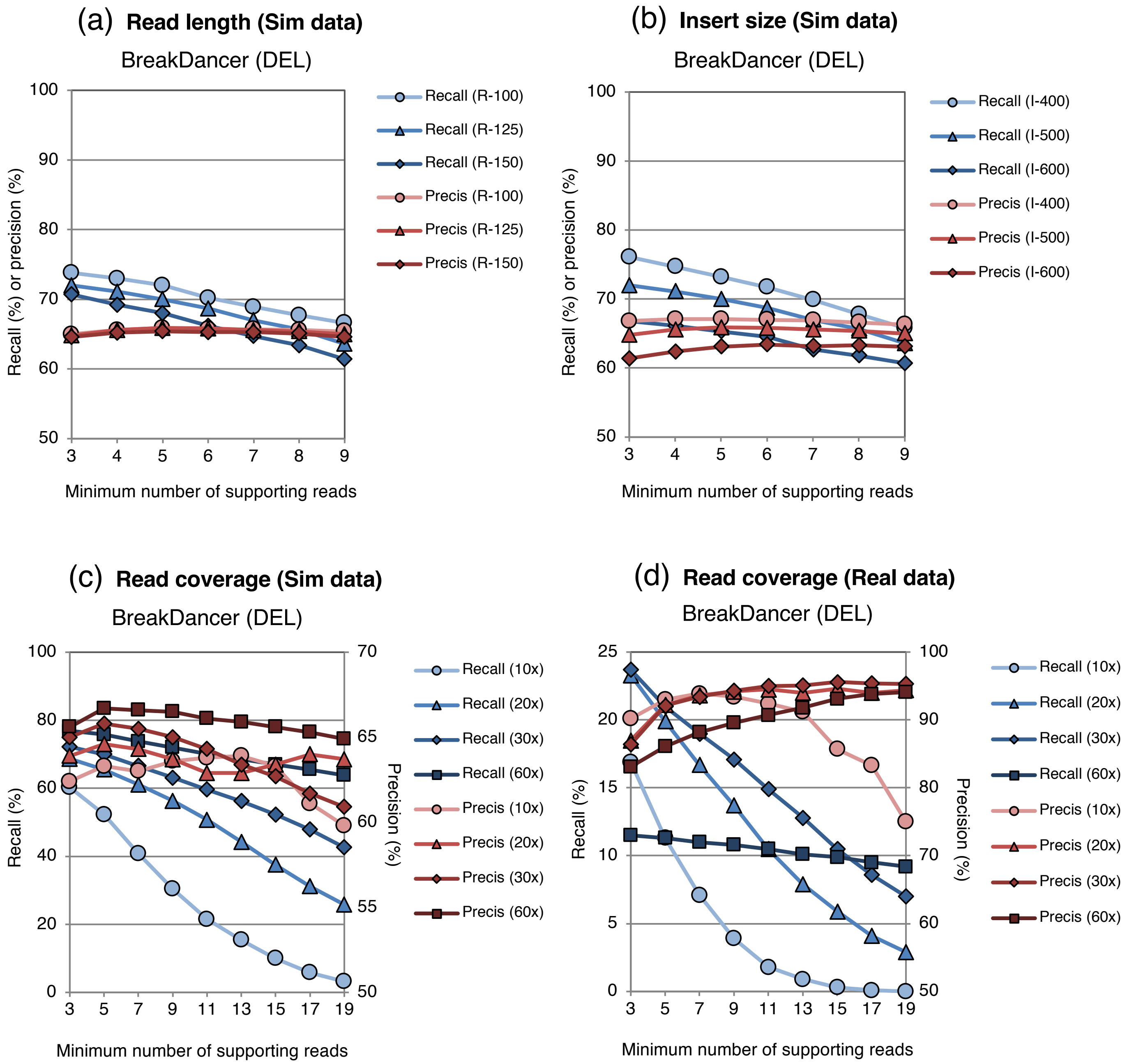

Figure S13-B. Results for DEL calling using BreakDancer. DELs were called using BreakDancer with the simulated data with different scales of read length (**a**), insert size (**b**), or read coverage (**c**) or with the real data with different scales of read coverage (**d**). It should be noted that the scales of the recall and precision were different in (c) and (d). The data are represented as in Figure S13-A.

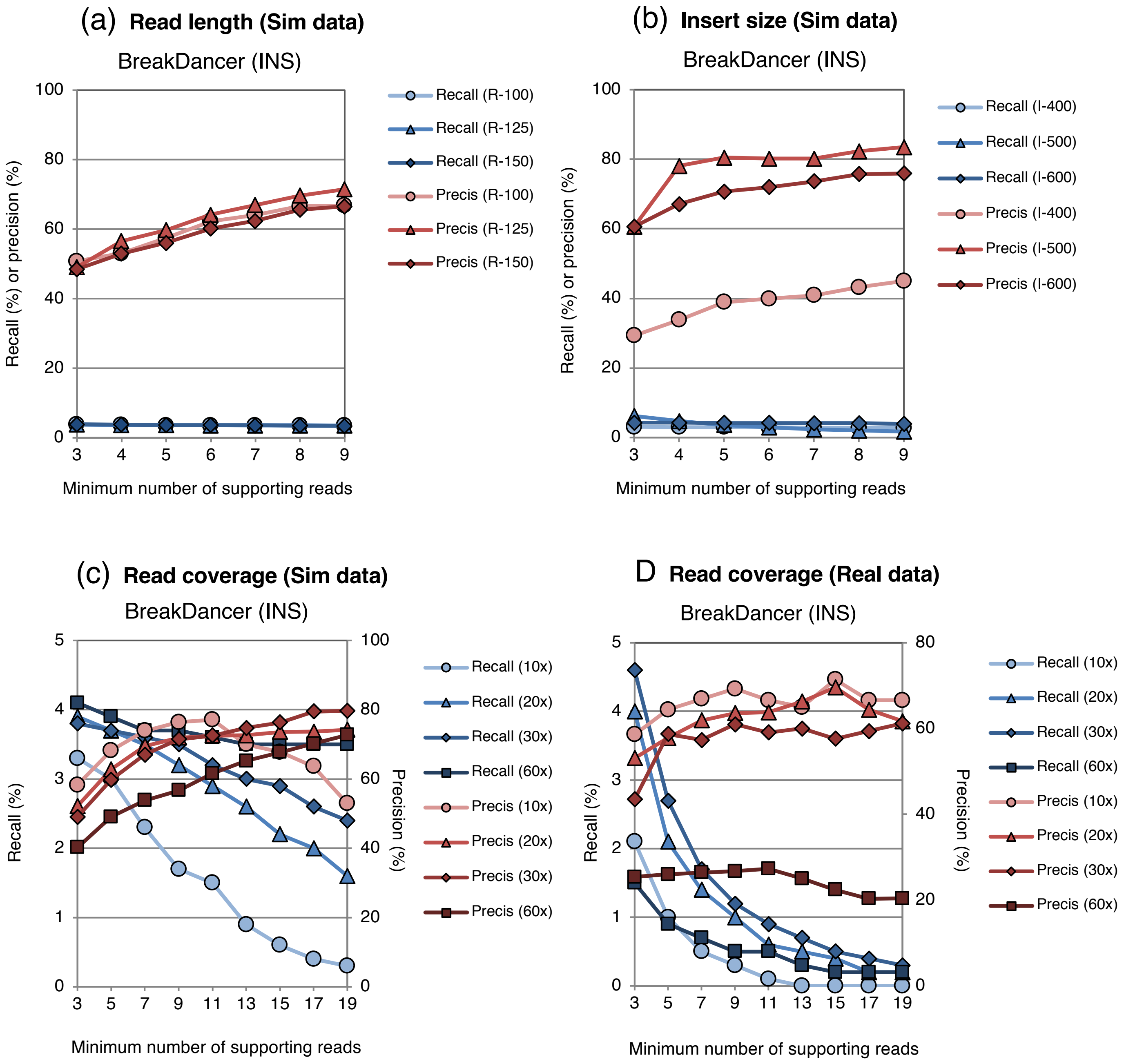

Figure S13-C. Results for INS calling using BreakDancer. INSs were called using BreakDancer with the simulated data with different scales of read length (**a**), insert size (**b**), or read coverage (**c**) or with the real data with different scales of read coverage (**d**). It should be noted that the scales of the recall and precision were different in (c) and (d). The data are represented as in Figure S13-A.

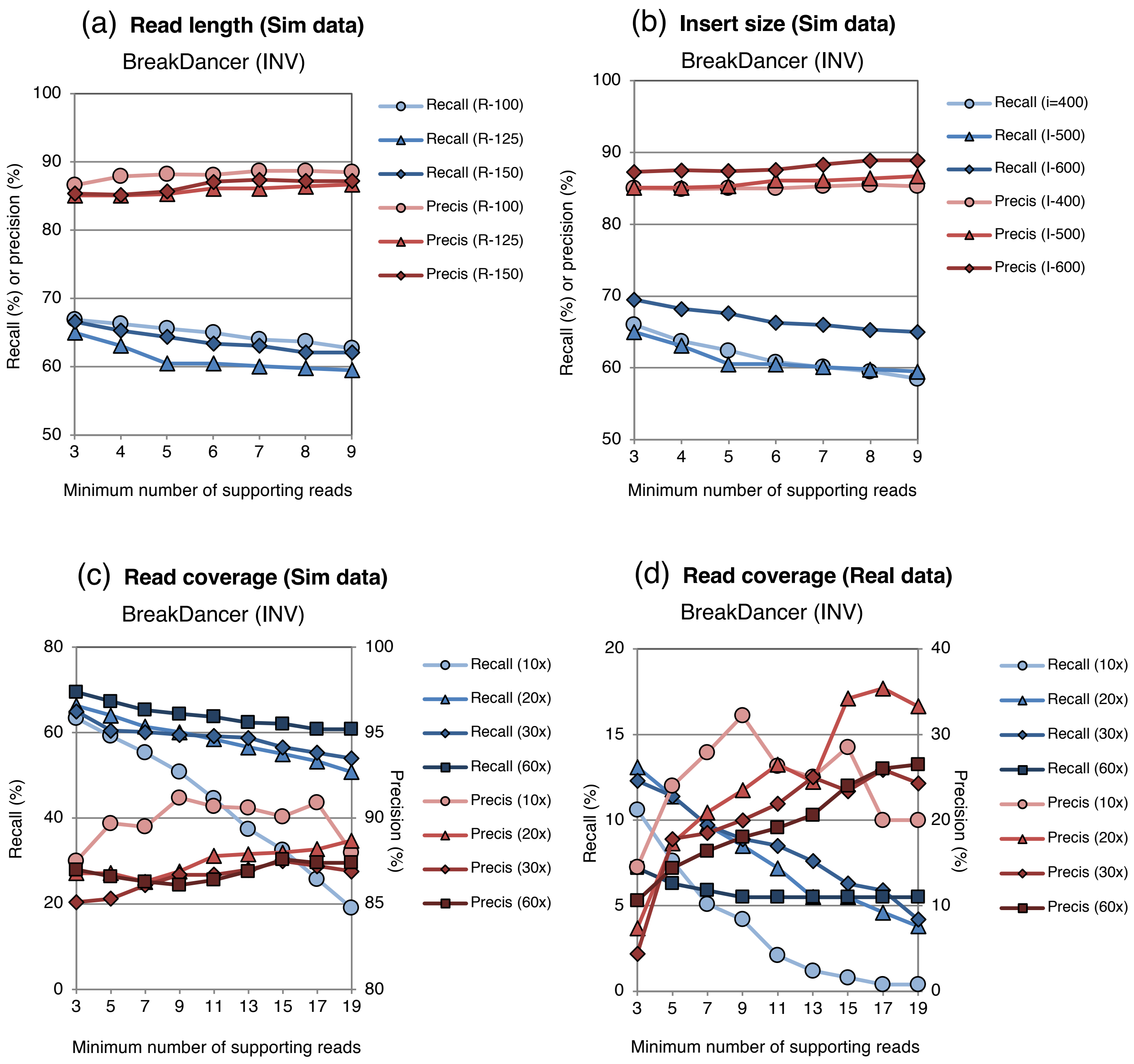

Figure S13-D. Results for INV calling using BreakDancer. INVs were called using BreakDancer with the simulated data with different scales of read length (**a**), insert size (**b**), or read coverage (**c**) or with the real data with different scales of read coverage (**d**). It should be noted that the scales of the recall and precision were different in (c) and (d). The data are represented as in Figure S13-A.

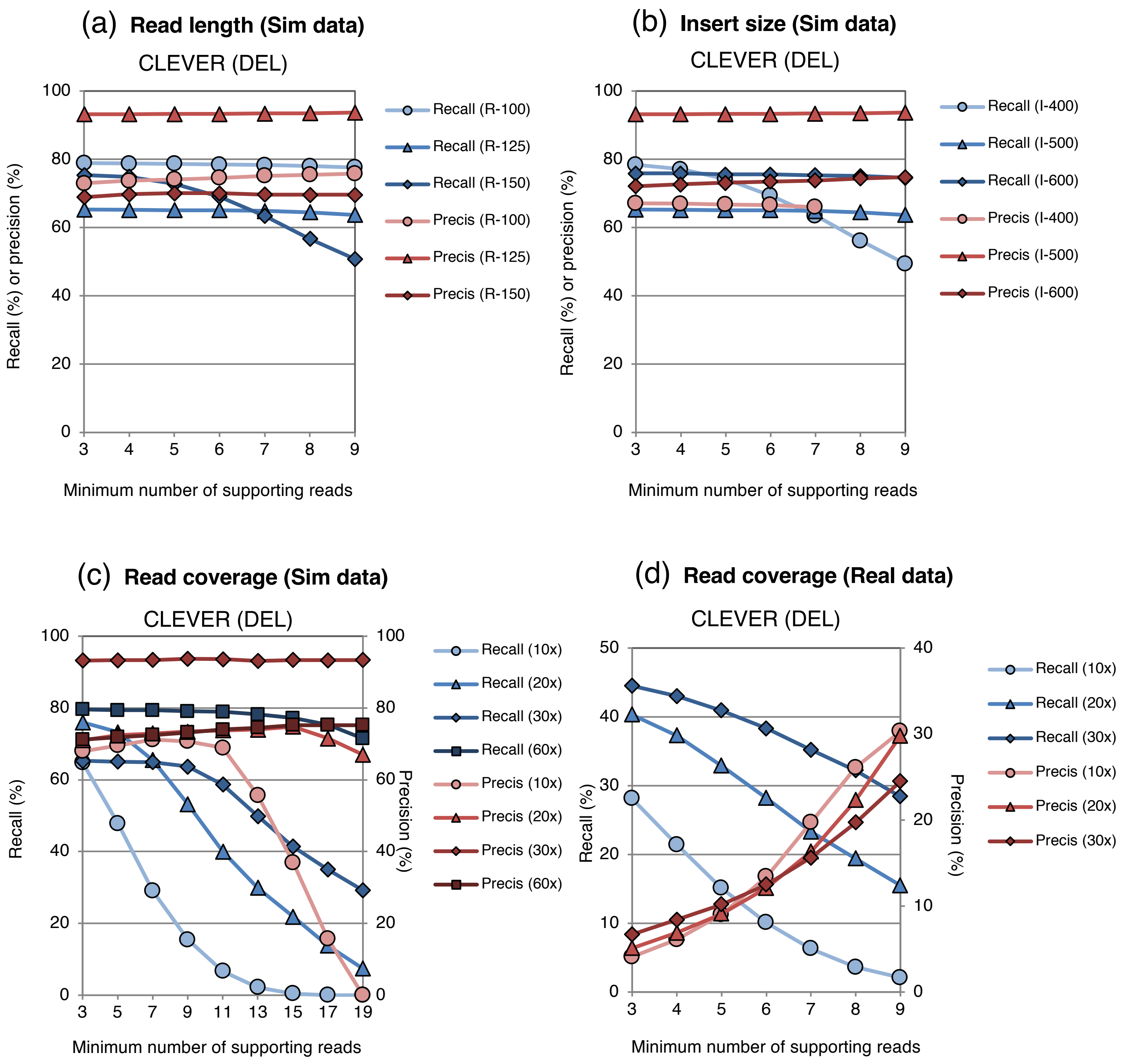

Figure S13-E. Results for DEL calling using CLEVER. DELs were called using CLEVER with the simulated data with different scales of read length (**a**), insert size (**b**), or read coverage (**c**) or with the real data with different scales of read coverage (**d**). It should be noted that the scales of the recall and precision were different in (c) and (d). The data are represented as in Figure S13-A.

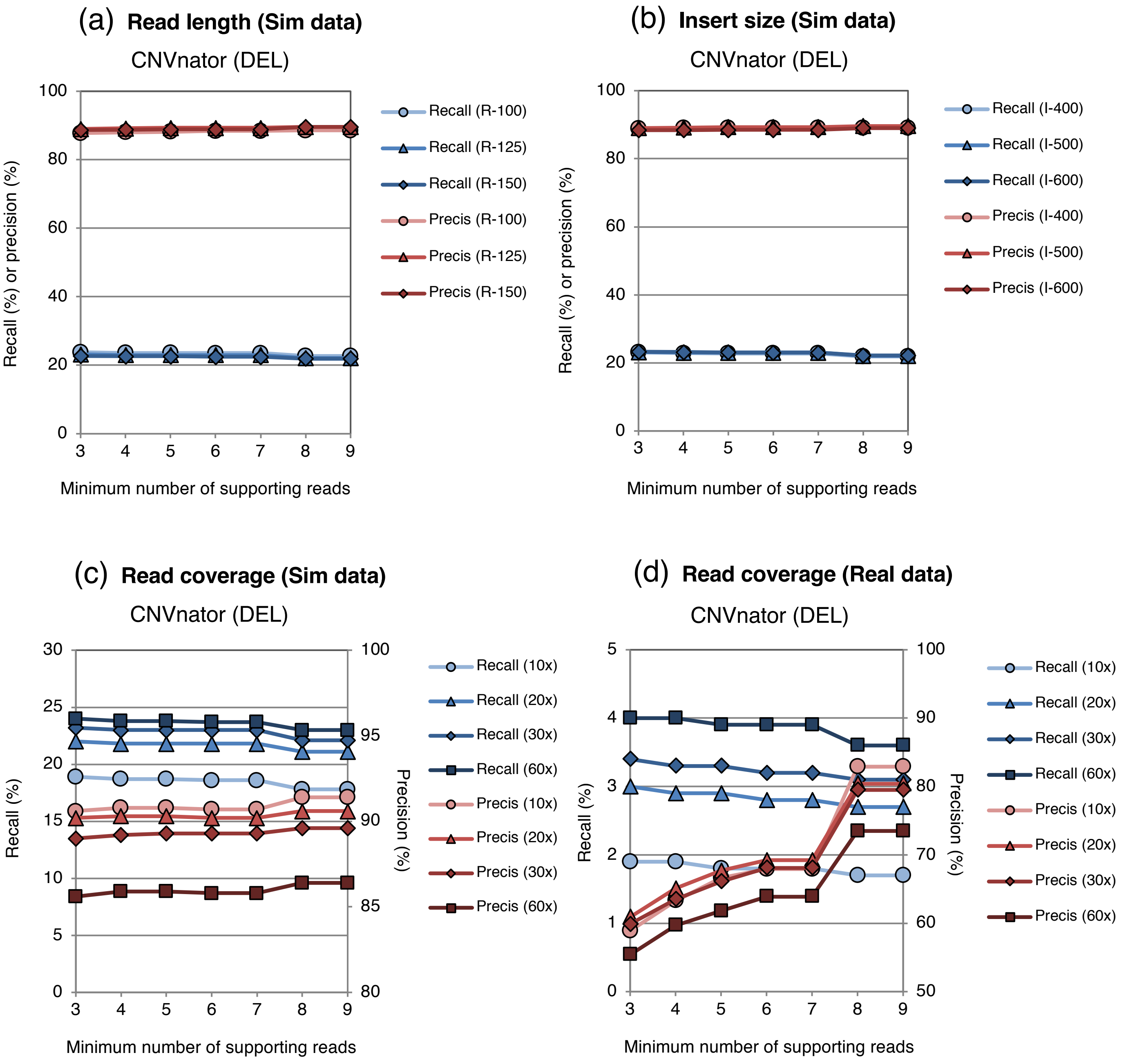

Figure S13-F. Results for DEL calling using CNVnator. DELs were called using CNVnator with the simulated data with different scales of read length (**a**), insert size (**b**), or read coverage (**c**) or with the real data with different scales of read coverage (**d**). It should be noted that the scales of the recall and precision were different in (c) and (d). The data are represented as in Figure S13-A.

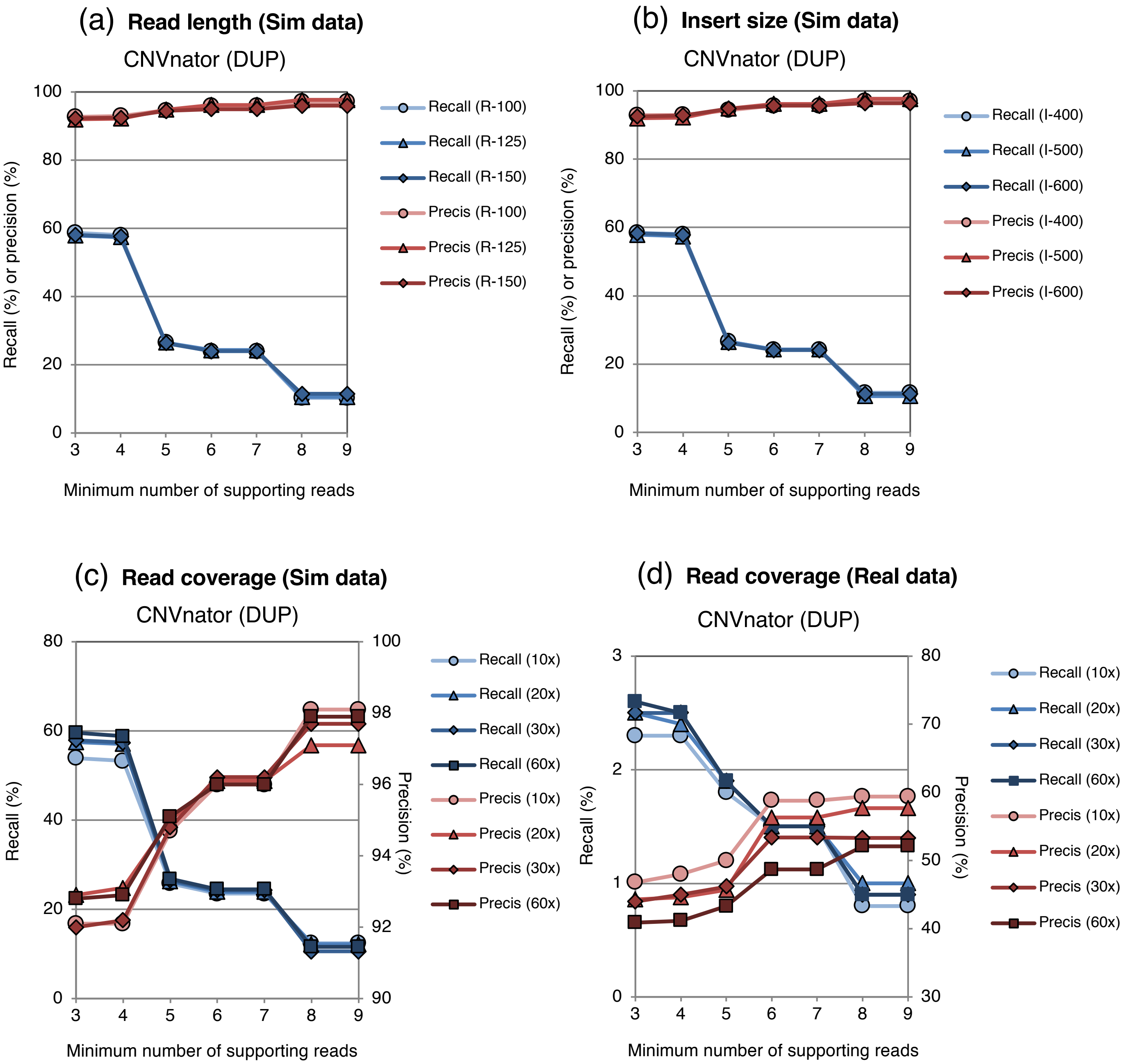

Figure S13-G. Results for DUP calling using CNVnator. DUPs were called using CNVnator with the simulated data with different scales of read length (**a**), insert size (**b**), or read coverage (**c**) or with the real data with different scales of read coverage (**d**). It should be noted that the scales of the recall and precision were different in (c) and (d). The data are represented as in Figure S13-A.

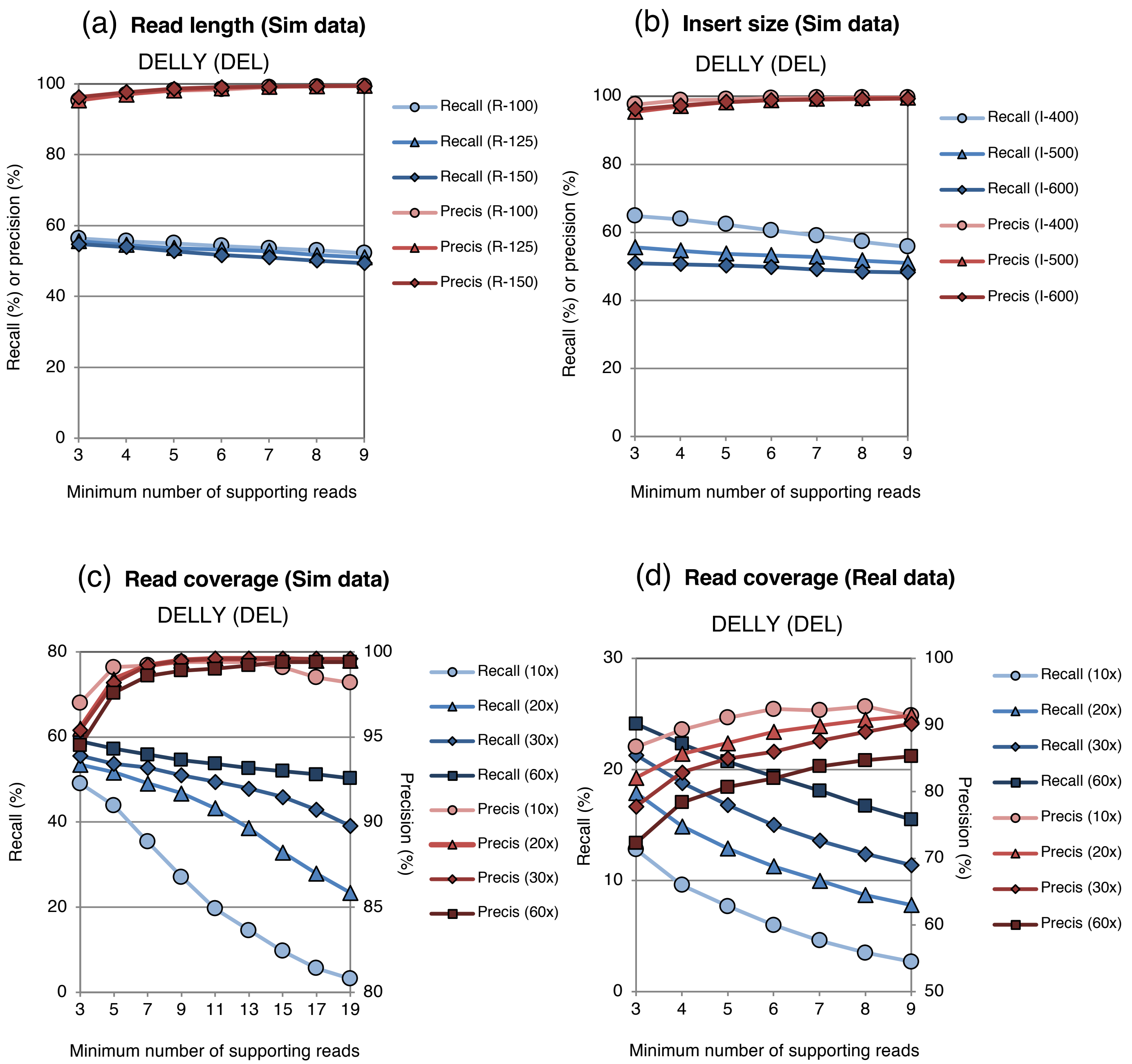

Figure S13-H. Results for DEL calling using DELLY. DELs were called using DELLY with the simulated data with different scales of read length (**a**), insert size (**b**), or read coverage (**c**) or with the real data with different scales of read coverage (**d**). It should be noted that the scales of the recall and precision were different in (c) and (d). The data are represented as in Figure S13-A.

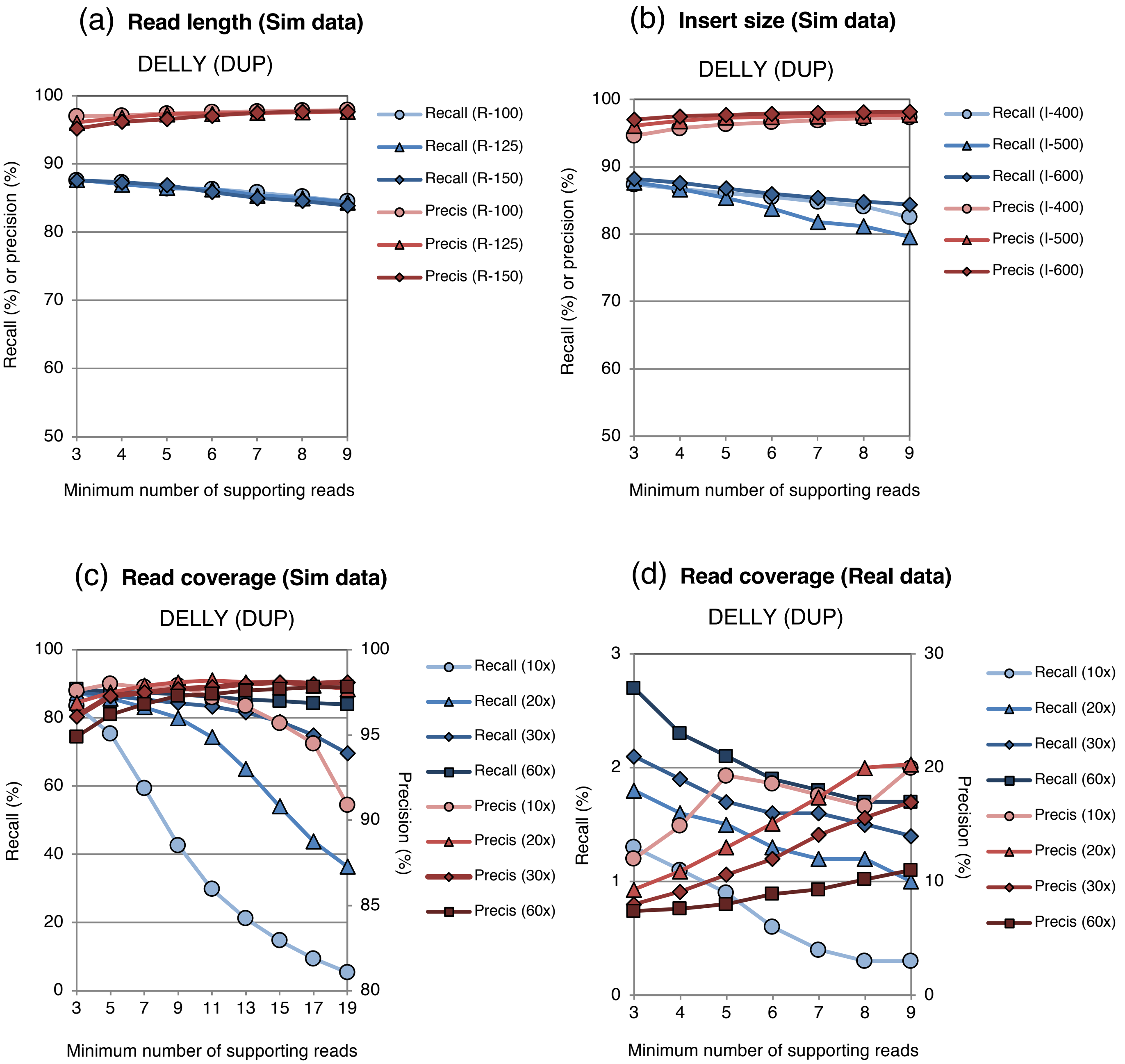

Figure S13-I. Results for DUP calling using DELLY. DUPs were called using DELLY with the simulated data with different scales of read length (**a**), insert size (**b**), or read coverage (**c**) or with the real data with different scales of read coverage (**d**). It should be noted that the scales of the recall and precision were different in (c) and (d). The data are represented as in Figure S13-A.

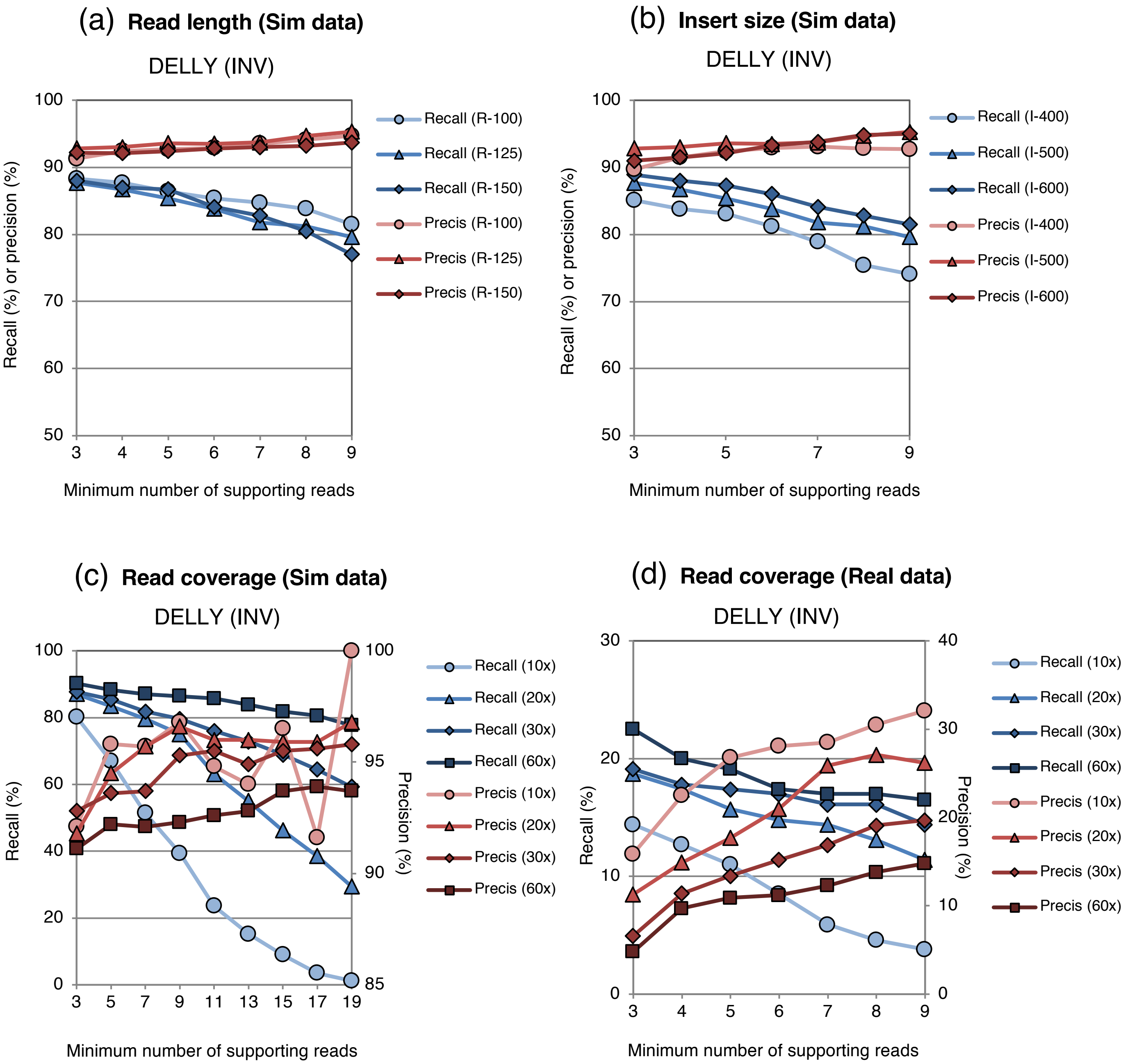

Figure S13-J. Results for INV calling using DELLY. INVs were called using DELLY with the simulated data with different scales of read length (**a**), insert size (**b**), or read coverage (**c**) or with the real data with different scales of read coverage (**d**). It should be noted that the scales of the recall and precision were different in (c) and (d). The data are represented as in Figure S13-A.

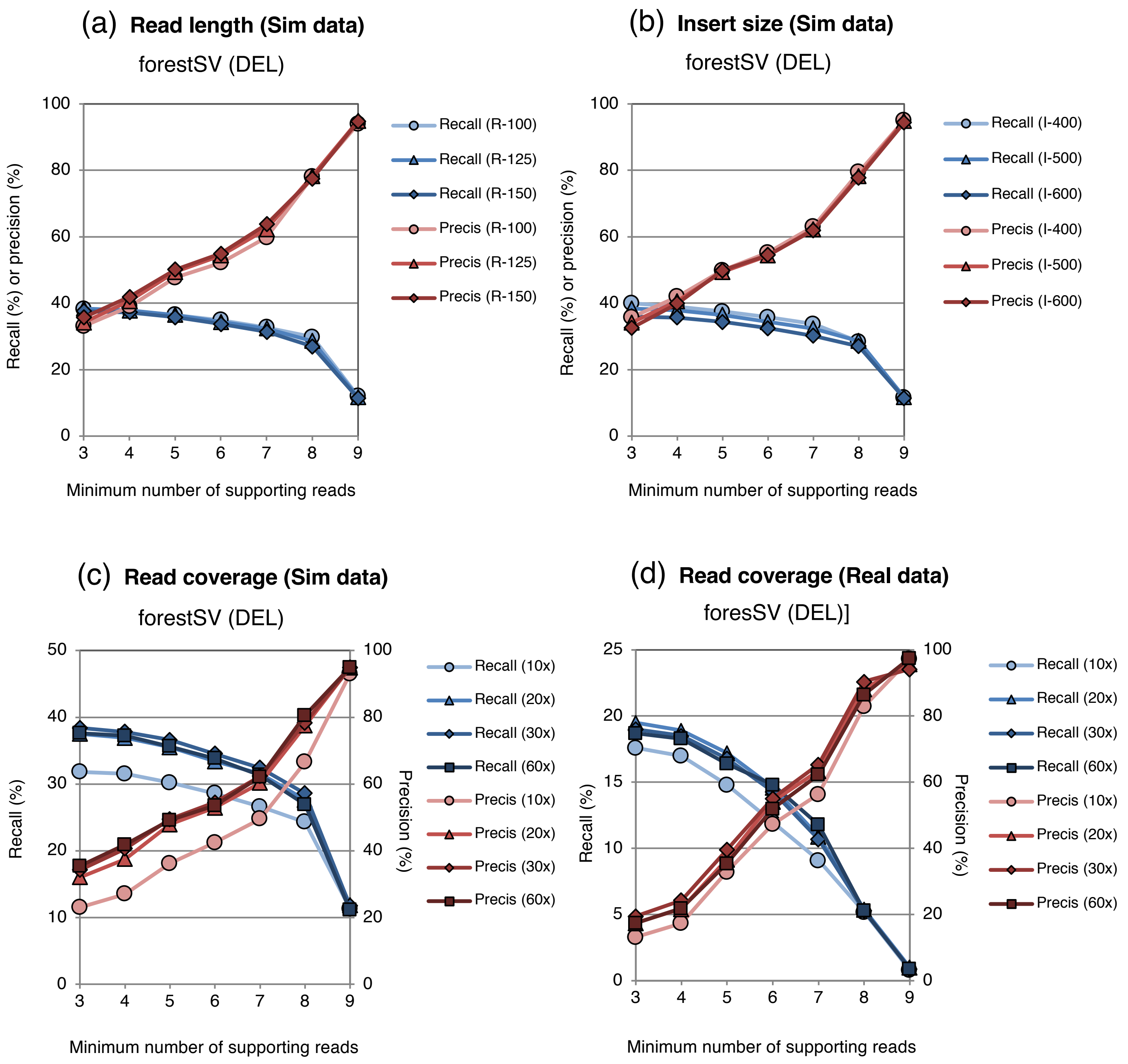

Figure S13-K. Results for DEL calling using forestSV. DELs were called using forestSV with the simulated data with different scales of read length (**a**), insert size (**b**), or read coverage (**c**) or with the real data with different scales of read coverage (**d**). It should be noted that the scales of the recall and precision were different in (c) and (d). The data are represented as in Figure S13-A.

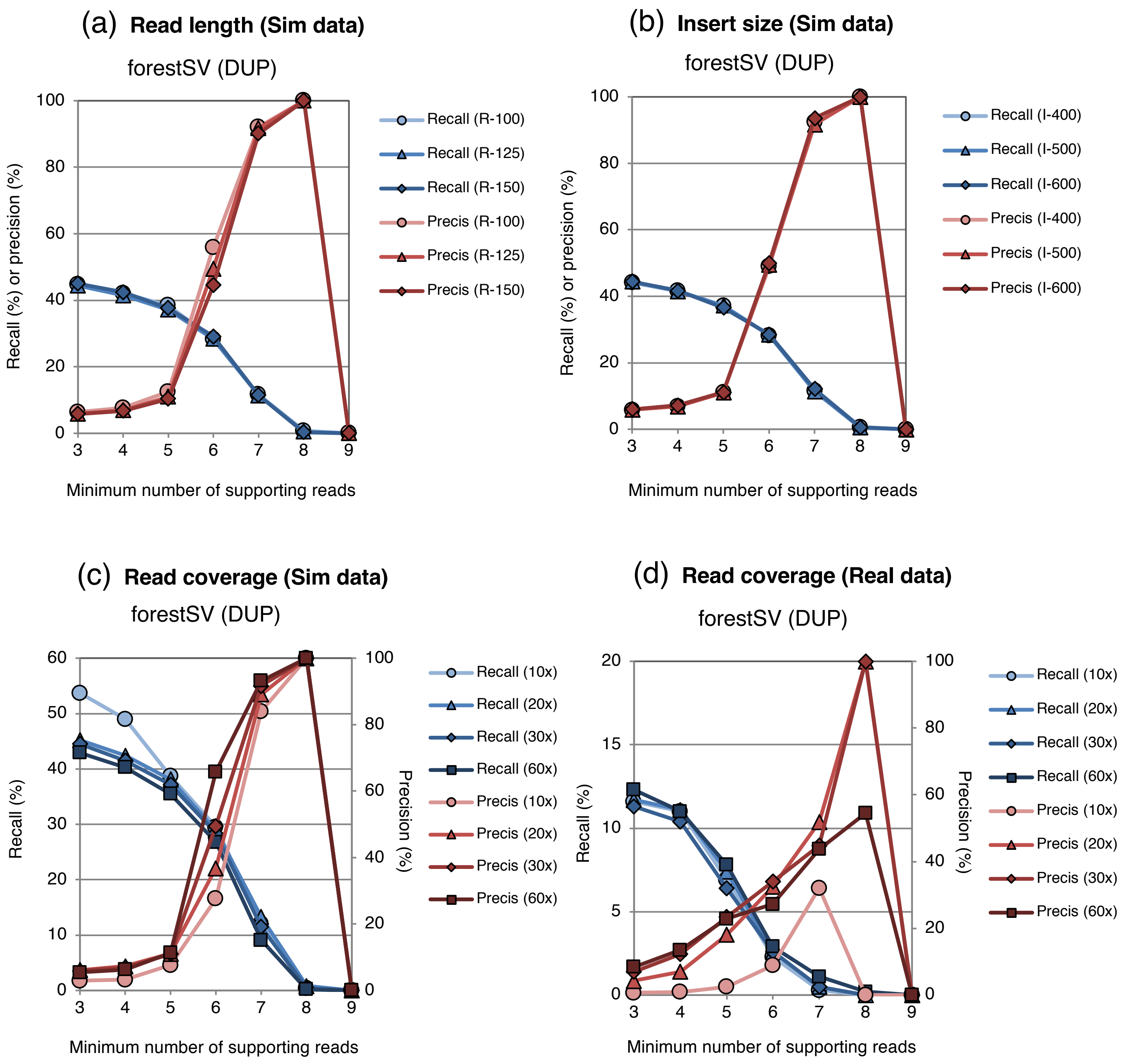

Figure S13-L. Results for DUP calling using forestSV. DUPs were called using forestSV with the simulated data with different scales of read length (**a**), insert size (**b**), or read coverage (**c**) or with the real data with different scales of read coverage (**d**). It should be noted that the scales of the recall and precision were different in (c) and (d). The data are represented as in Figure S13-A.

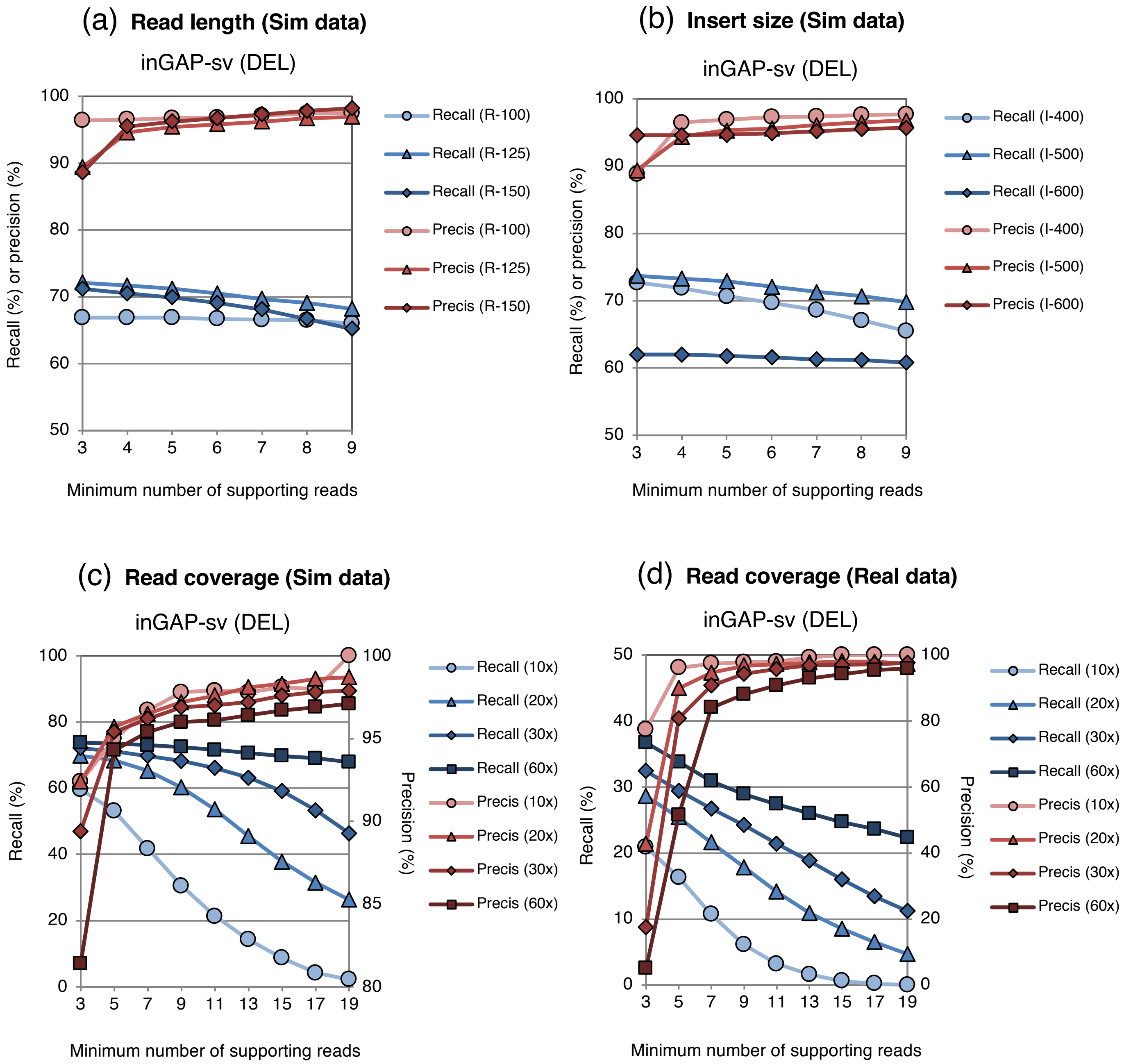

Figure S13-M. Results for DEL calling using inGAP-sv. DELs were called using inGAP-sv with the simulated data with different scales of read length (**a**), insert size (**b**), or read coverage (**c**) or with the real data with different scales of read coverage (**d**). It should be noted that the scales of the recall and precision were different in (c) and (d). The data are represented as in Figure S13-A.

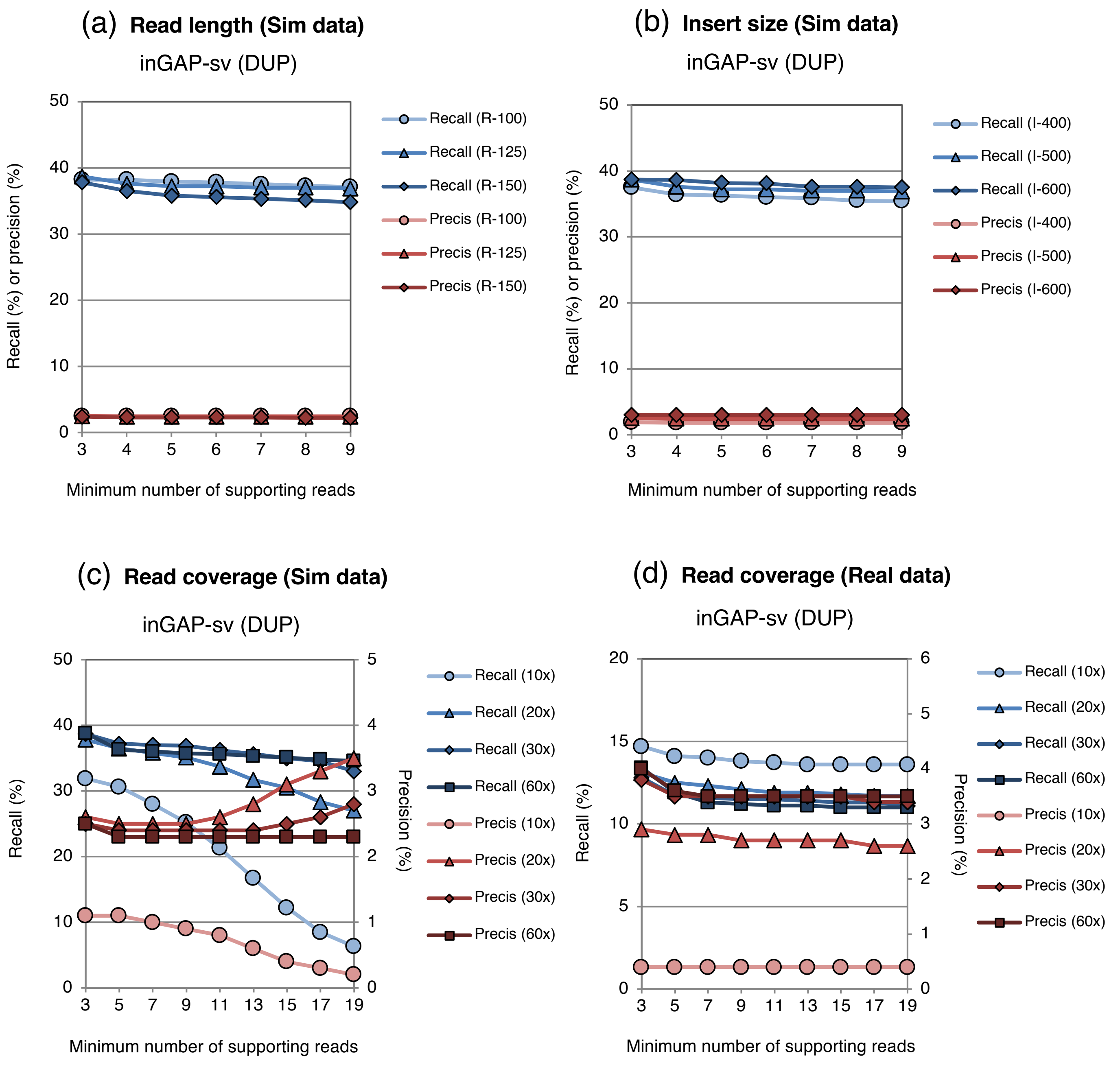

Figure S13-N. Results for DUP calling using inGAP-sv. DUPs were called using inGAP-sv with the simulated data with different scales of read length (**a**), insert size (**b**), or read coverage (**c**) or with the real data with different scales of read coverage (**d**). It should be noted that the scales of the recall and precision were different in (c) and (d). The data are represented as in Figure S13-A.

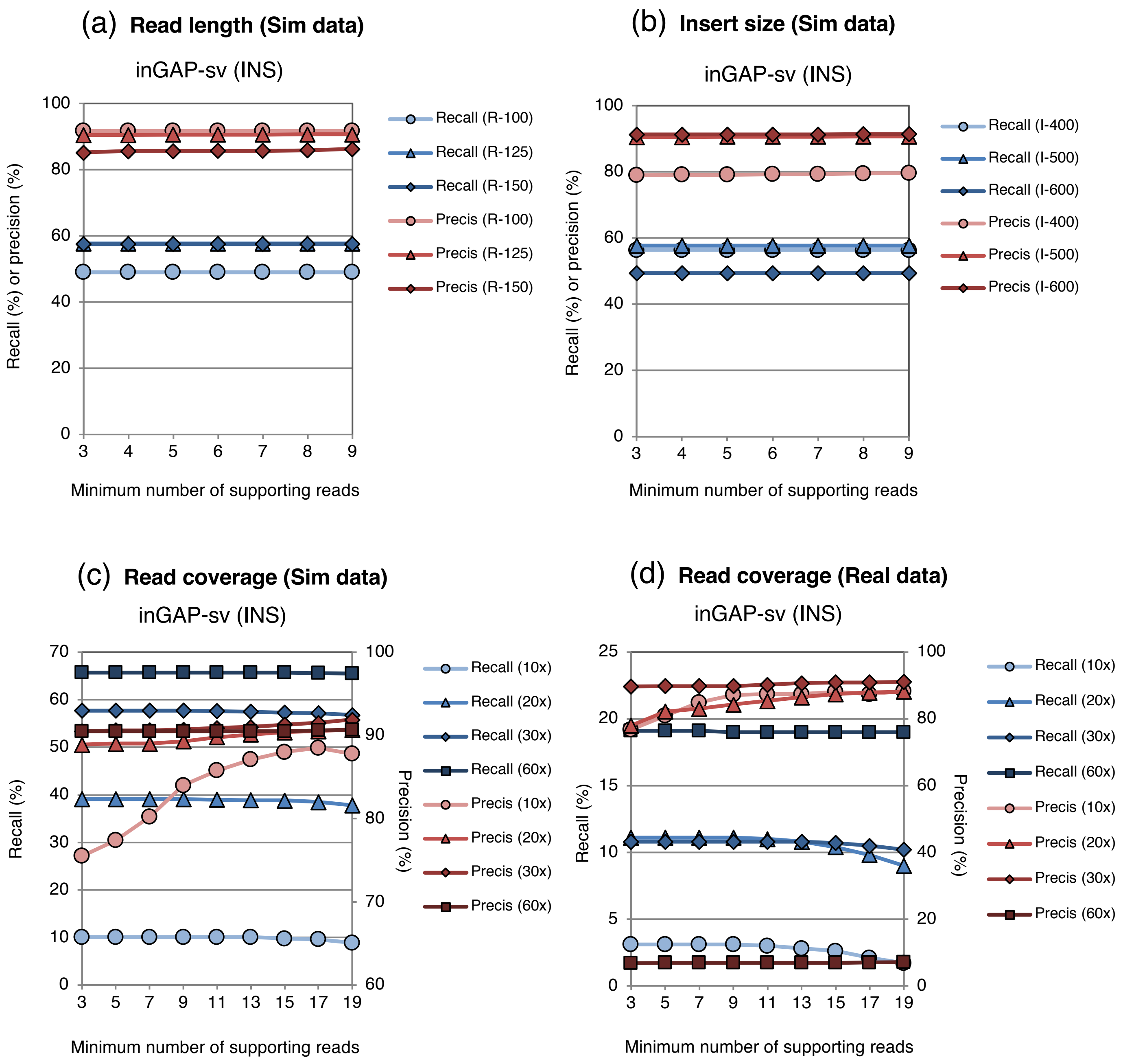

Figure S13-O. Results for INS calling using inGAP-sv. INSs were called using inGAP-sv with the simulated data with different scales of read length (**a**), insert size (**b**), or read coverage (**c**) or with the real data with different scales of read coverage (**d**). It should be noted that the scales of the recall and precision were different in (c) and (d). The data are represented as in Figure S13-A.

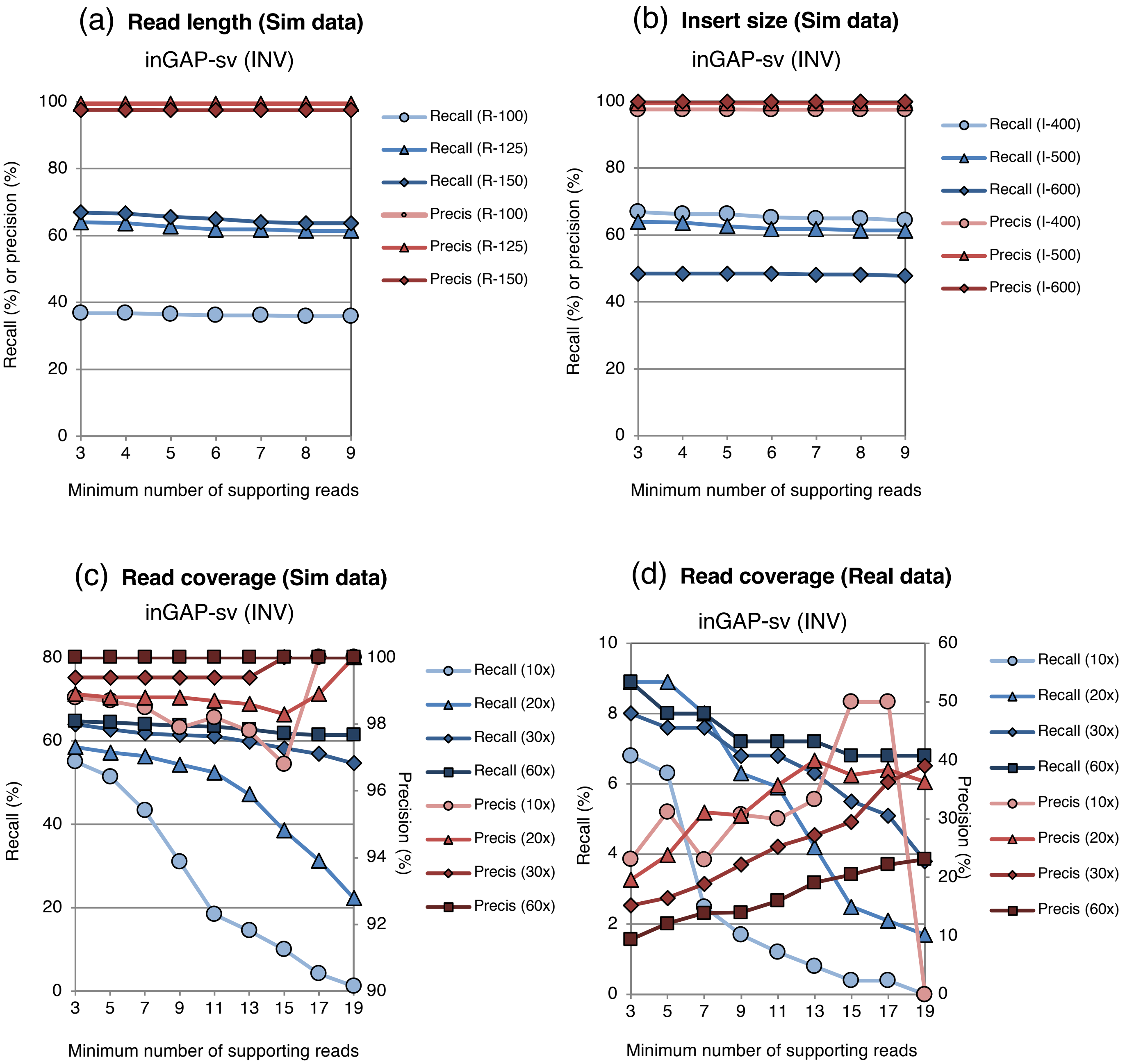

Figure S13-P. Results for INV calling using inGAP-sv. INVs were called using inGAP-sv with the simulated data with different scales of read length (**a**), insert size (**b**), or read coverage (**c**) or with the real data with different scales of read coverage (**d**). It should be noted that the scales of the recall and precision were different in (c) and (d). The data are represented as in Figure S13-A.

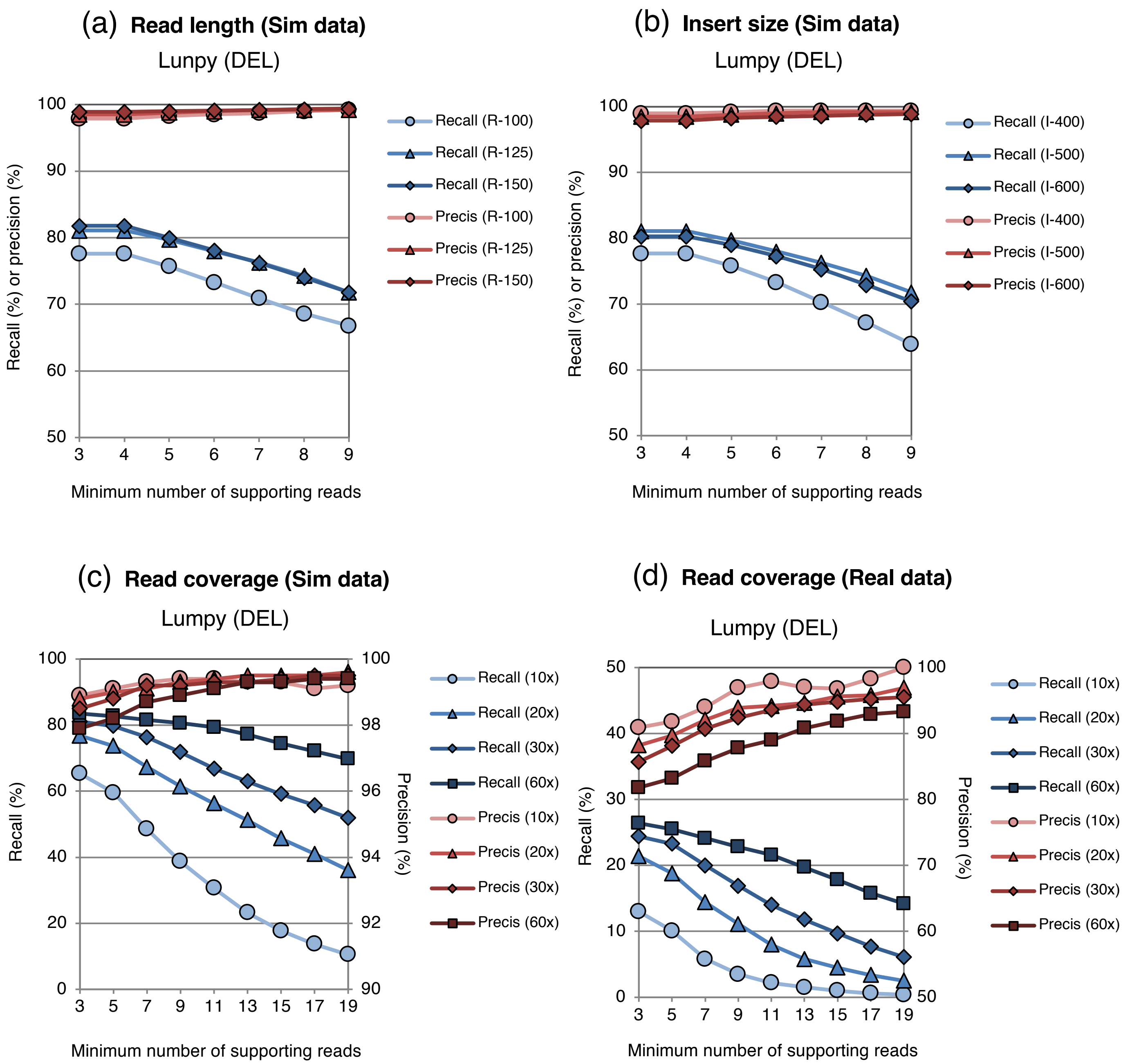

Figure S13-Q. Results for DEL calling using Lumpy. DELs were called using Lumpy with the simulated data with different scales of read length (**a**), insert size (**b**), or read coverage (**c**) or with the real data with different scales of read coverage (**d**). It should be noted that the scales of the recall and precision were different in (c) and (d). The data are represented as in Figure S13-A.

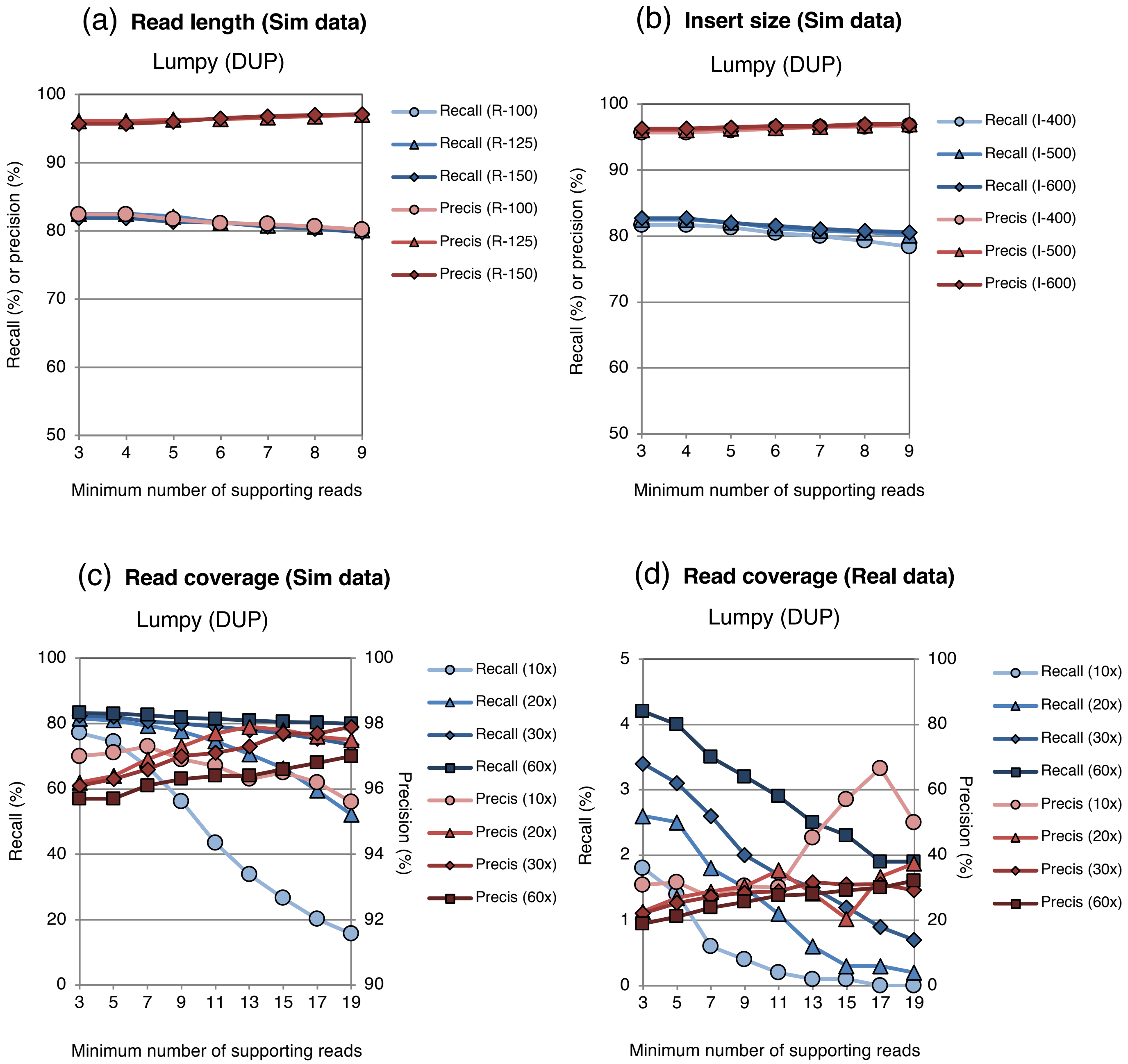

Figure S13-R. Results for DUP calling using Lumpy. DUPs were called using Lumpy with the simulated data with different scales of read length (**a**), insert size (**b**), or read coverage (**c**) or with the real data with different scales of read coverage (**d**). It should be noted that the scales of the recall and precision were different in (c) and (d). The data are represented as in Figure S13-A.

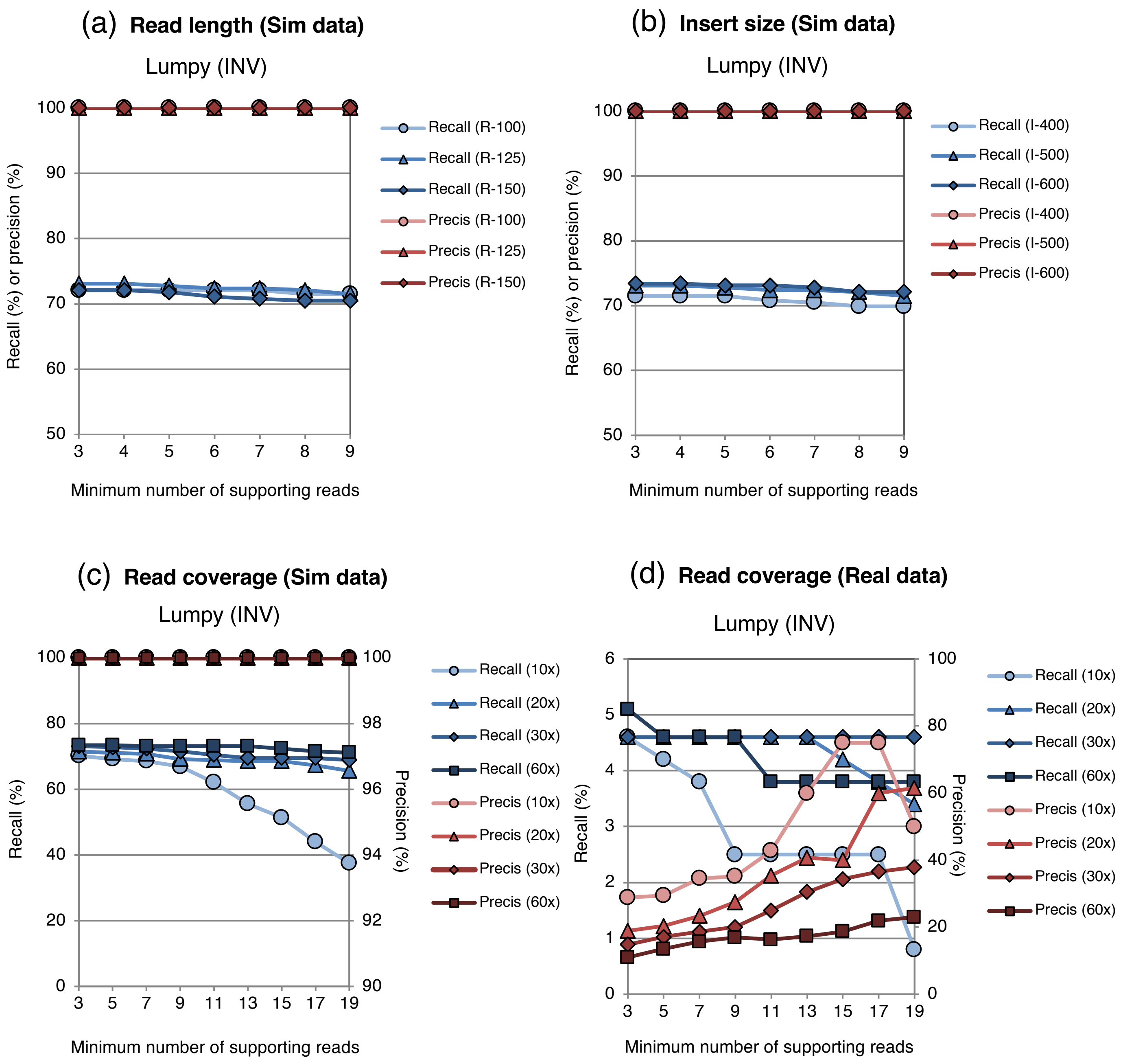

Figure S13-S. Results for INV calling using Lumpy. INVs were called using Lumpy with the simulated data with different scales of read length (**a**), insert size (**b**), or read coverage (**c**) or with the real data with different scales of read coverage (**d**). It should be noted that the scales of the recall and precision were different in (c) and (d). The data are represented as in Figure S13-A.

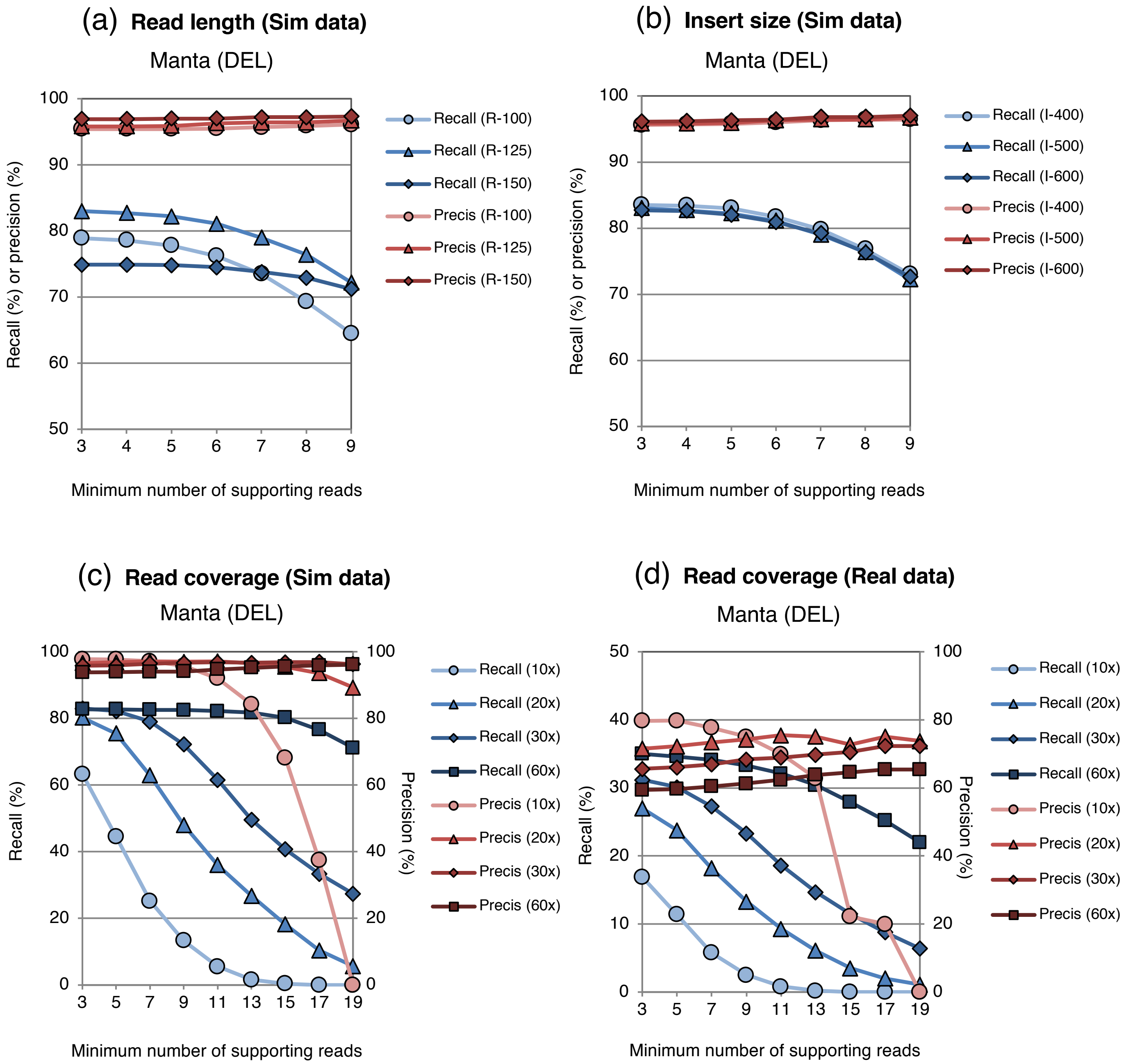

Figure S13-T. Results for DEL calling using Manta. DELs were called using Manta with the simulated data with different scales of read length (**a**), insert size (**b**), or read coverage (**c**) or with the real data with different scales of read coverage (**d**). It should be noted that the scales of the recall and precision were different in (c) and (d). The data are represented as in Figure S13-A.

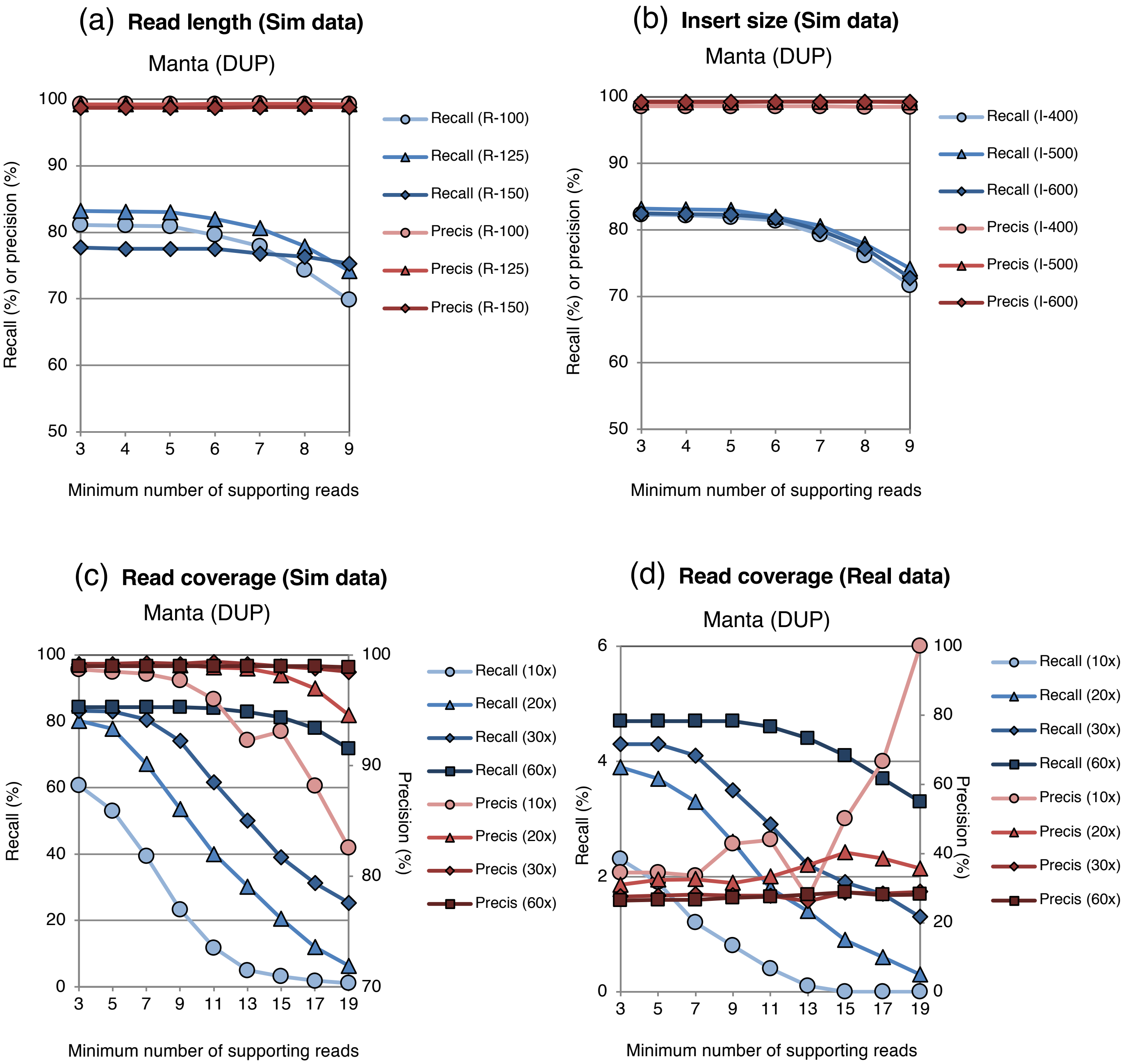

Figure S13-U. Results for DUP calling using Manta. DUPs were called using Manta with the simulated data with different scales of read length (**a**), insert size (**b**), or read coverage (**c**) or with the real data with different scales of read coverage (**d**). It should be noted that the scales of the recall and precision were different in (c) and (d). The data are represented as in Figure S13-A.

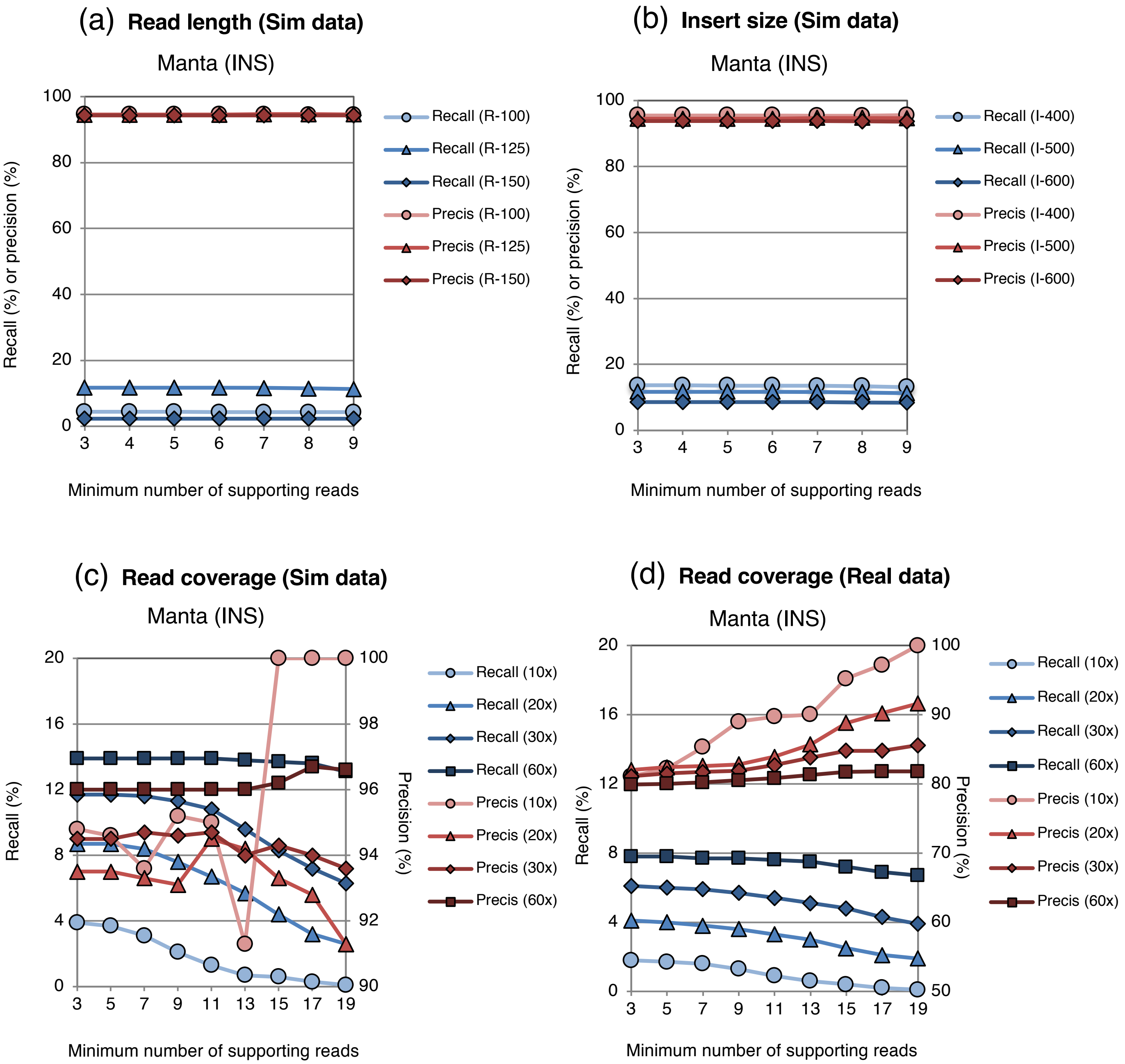

Figure S13-V. Results for INS calling using Manta. INSs were called using Manta with the simulated data with different scales of read length (**a**), insert size (**b**), or read coverage (**c**) or with the real data with different scales of read coverage (**d**). It should be noted that the scales of the recall and precision were different in (c) and (d). The data are represented as in Figure S13-A.

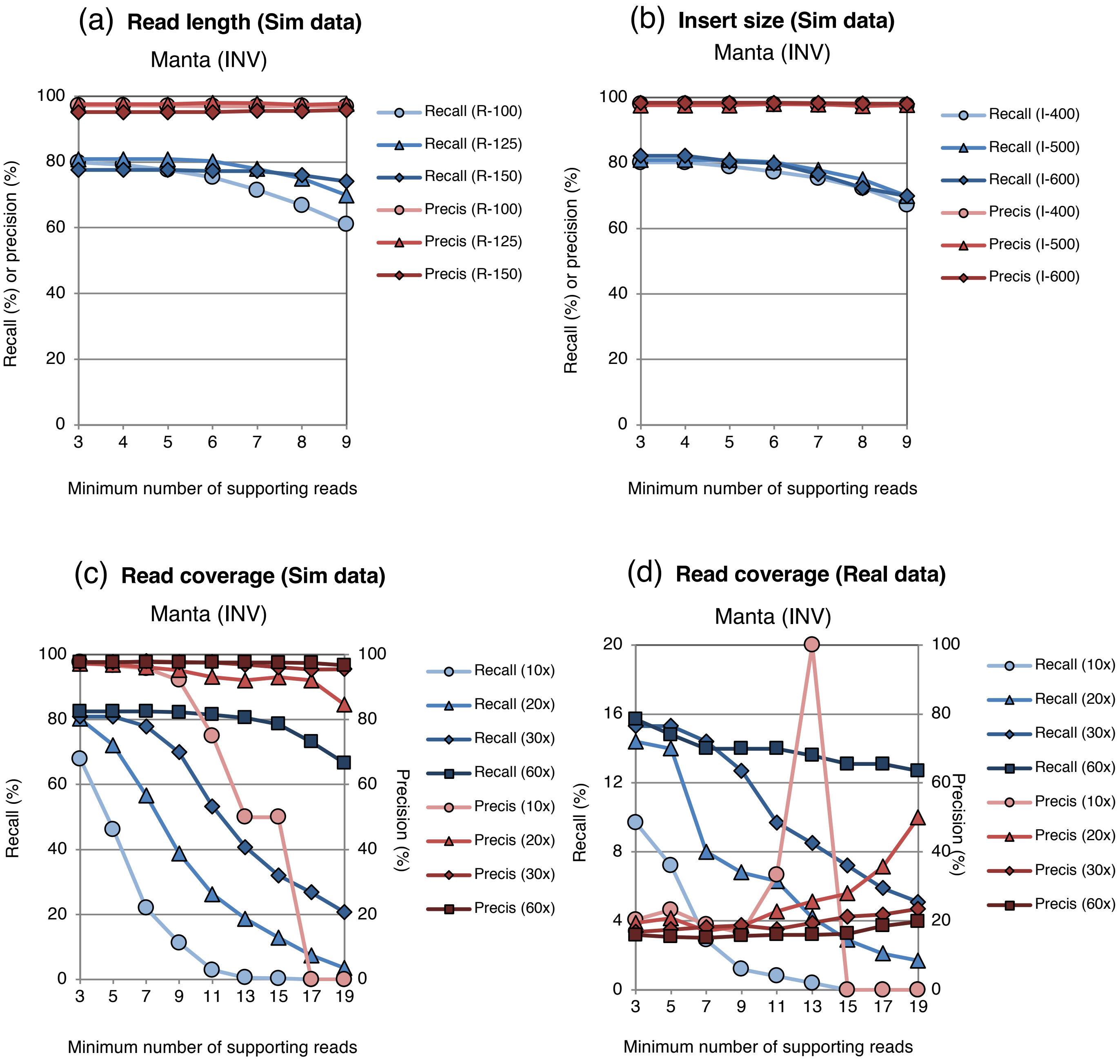

Figure S13-W. Results for INV calling using Manta. INVs were called using Manta with the simulated data with different scales of read length (**a**), insert size (**b**), or read coverage (**c**) or with the real data with different scales of read coverage (**d**). It should be noted that the scales of the recall and precision were different in (c) and (d). The data are represented as in Figure S13-A.

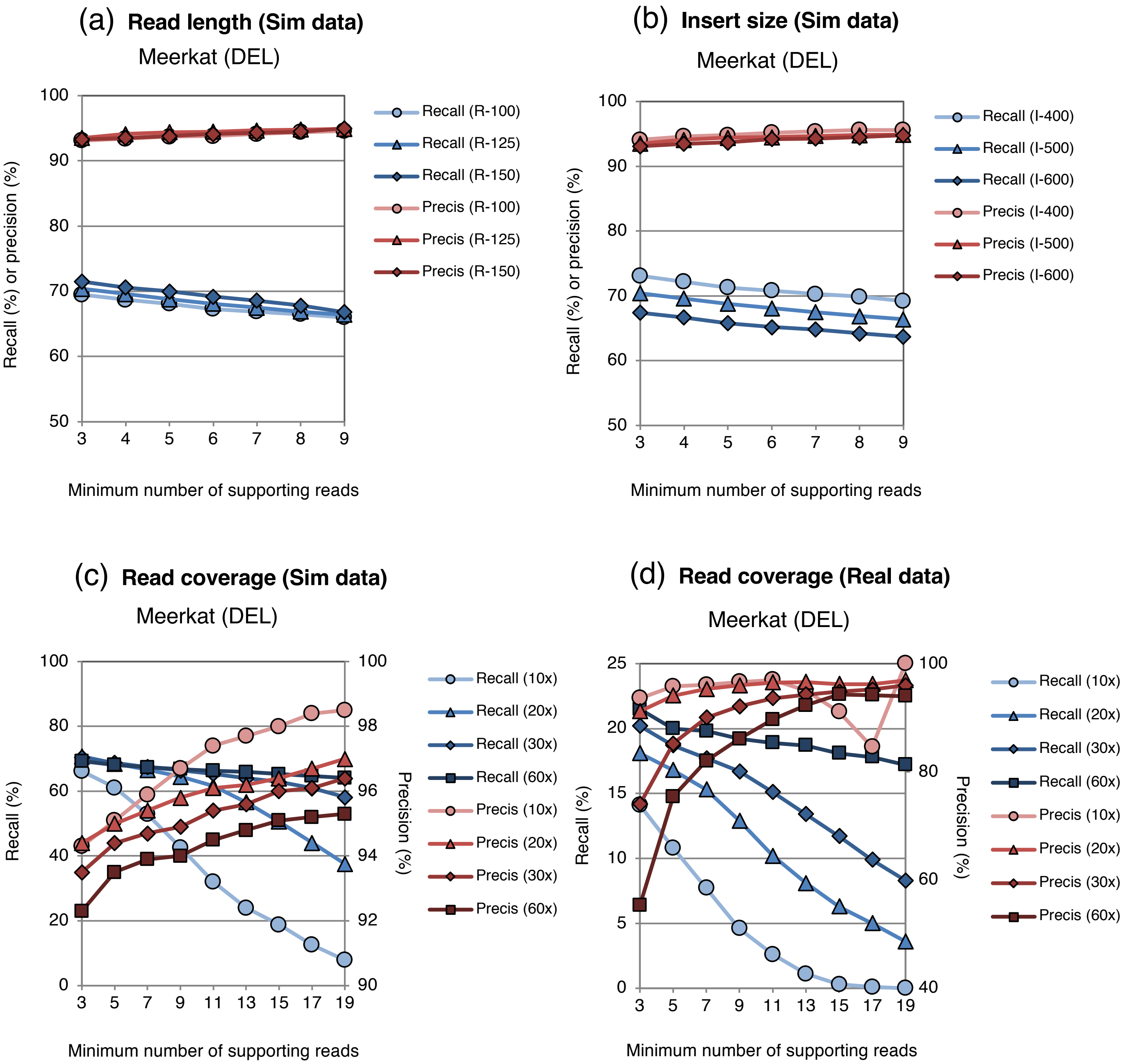

Figure S13-X. Results for DEL calling using Meerkat. DELs were called using Meerkat with the simulated data with different scales of read length (**a**), insert size (**b**), or read coverage (**c**) or with the real data with different scales of read coverage (**d**). It should be noted that the scales of the recall and precision were different in (c) and (d). The data are represented as in Figure S13-A.

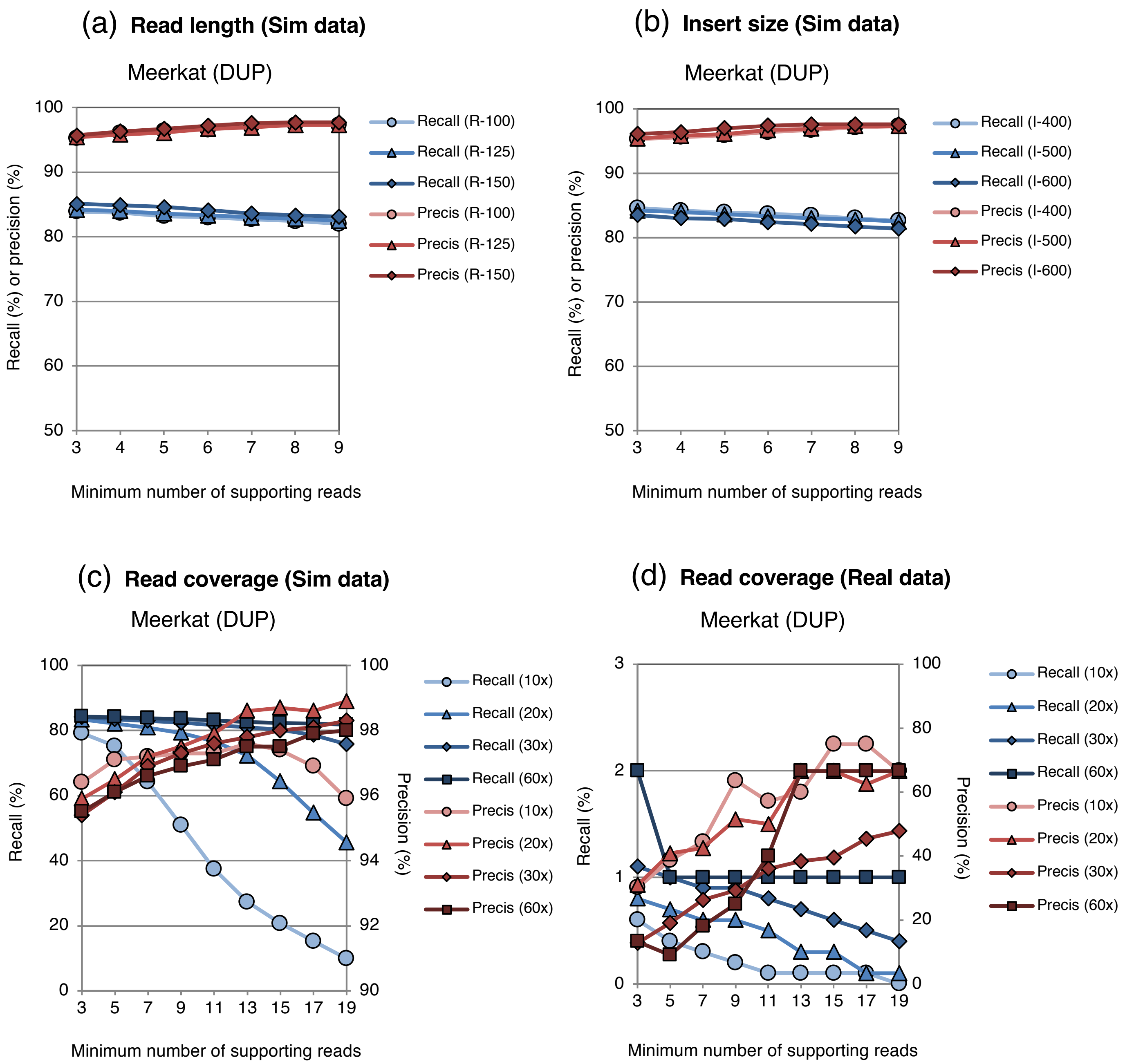

Figure S13-Y. Results for DUP calling using Meerkat. DUPs were called using Meerkat with the simulated data with different scales of read length (**a**), insert size (**b**), or read coverage (**c**) or with the real data with different scales of read coverage (**d**). It should be noted that the scales of the recall and precision were different in (c) and (d). The data are represented as in Figure S13-A.

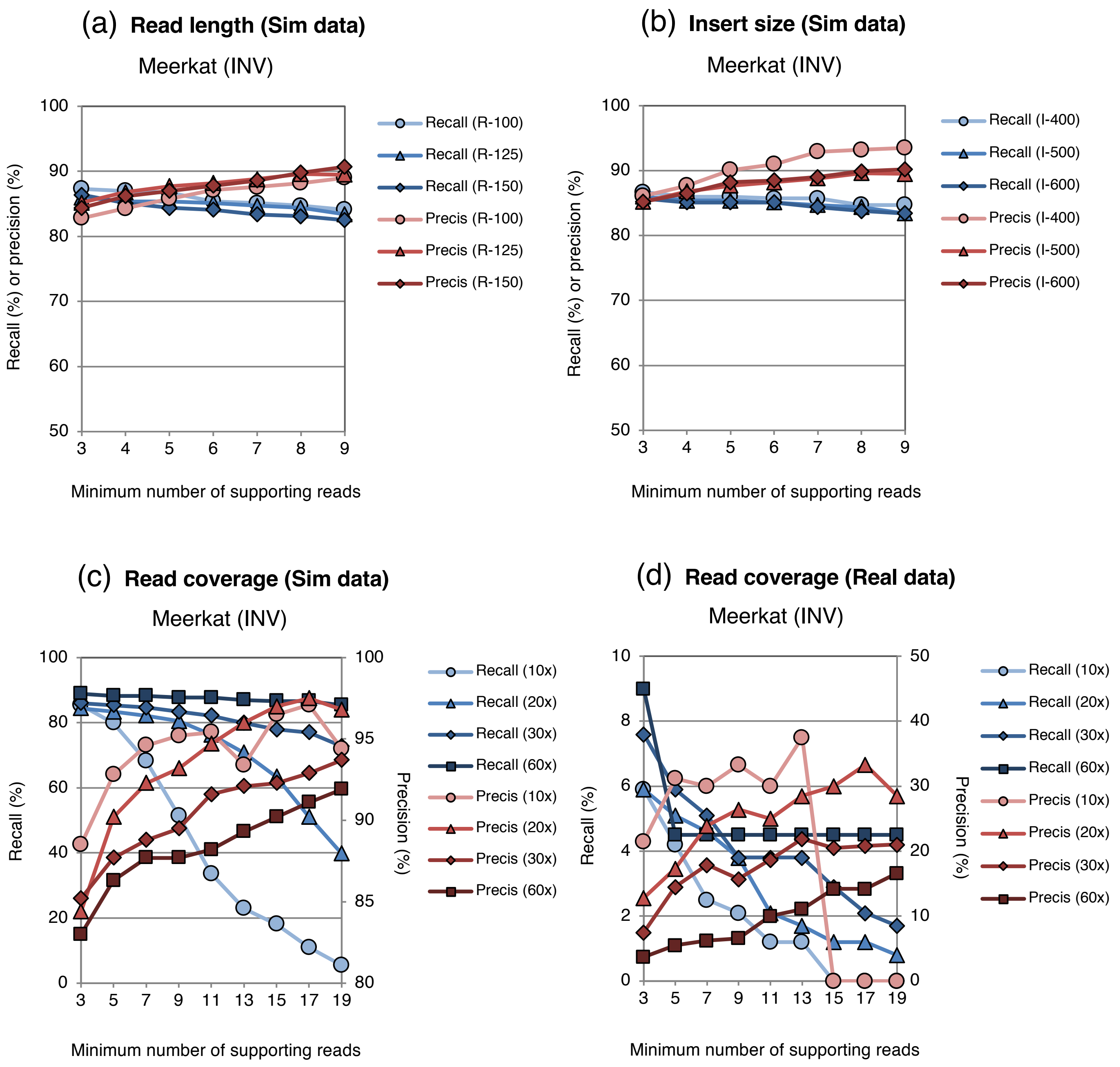

Figure S13-Z. Results for INV calling using Meerkat. INVs were called using Meerkat with the simulated data with different scales of read length (**a**), insert size (**b**), or read coverage (**c**) or with the real data with different scales of read coverage (**d**). It should be noted that the scales of the recall and precision were different in (c) and (d). The data are represented as in Figure S13-A.

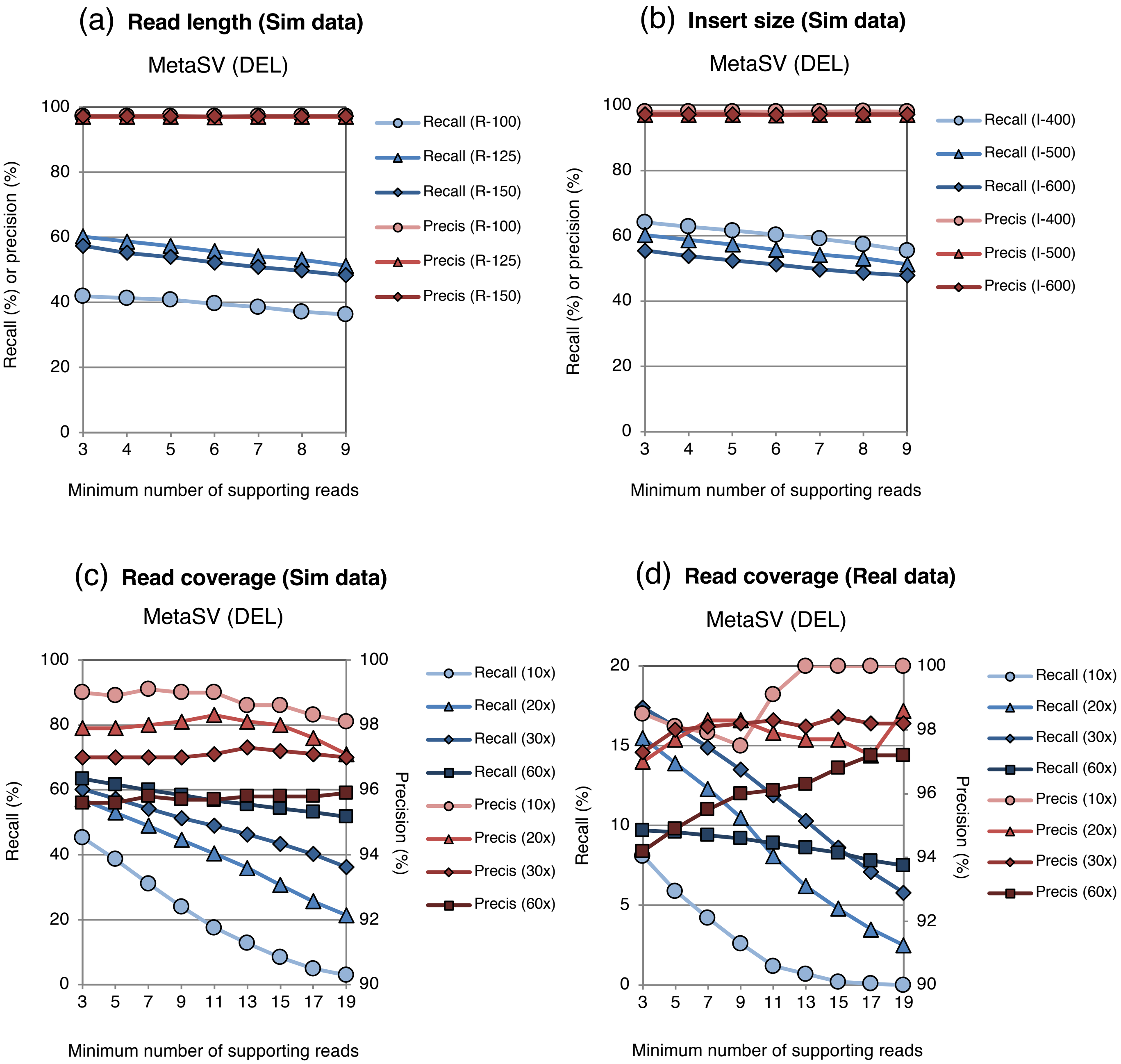

Figure S13-AA. Results for DEL calling using MetaSV. DELs were called using MetaSV with the simulated data with different scales of read length (**a**), insert size (**b**), or read coverage (**c**) or with the real data with different scales of read coverage (**d**). It should be noted that the scales of the recall and precision were different in (c) and (d). The data are represented as in Figure S13-A.

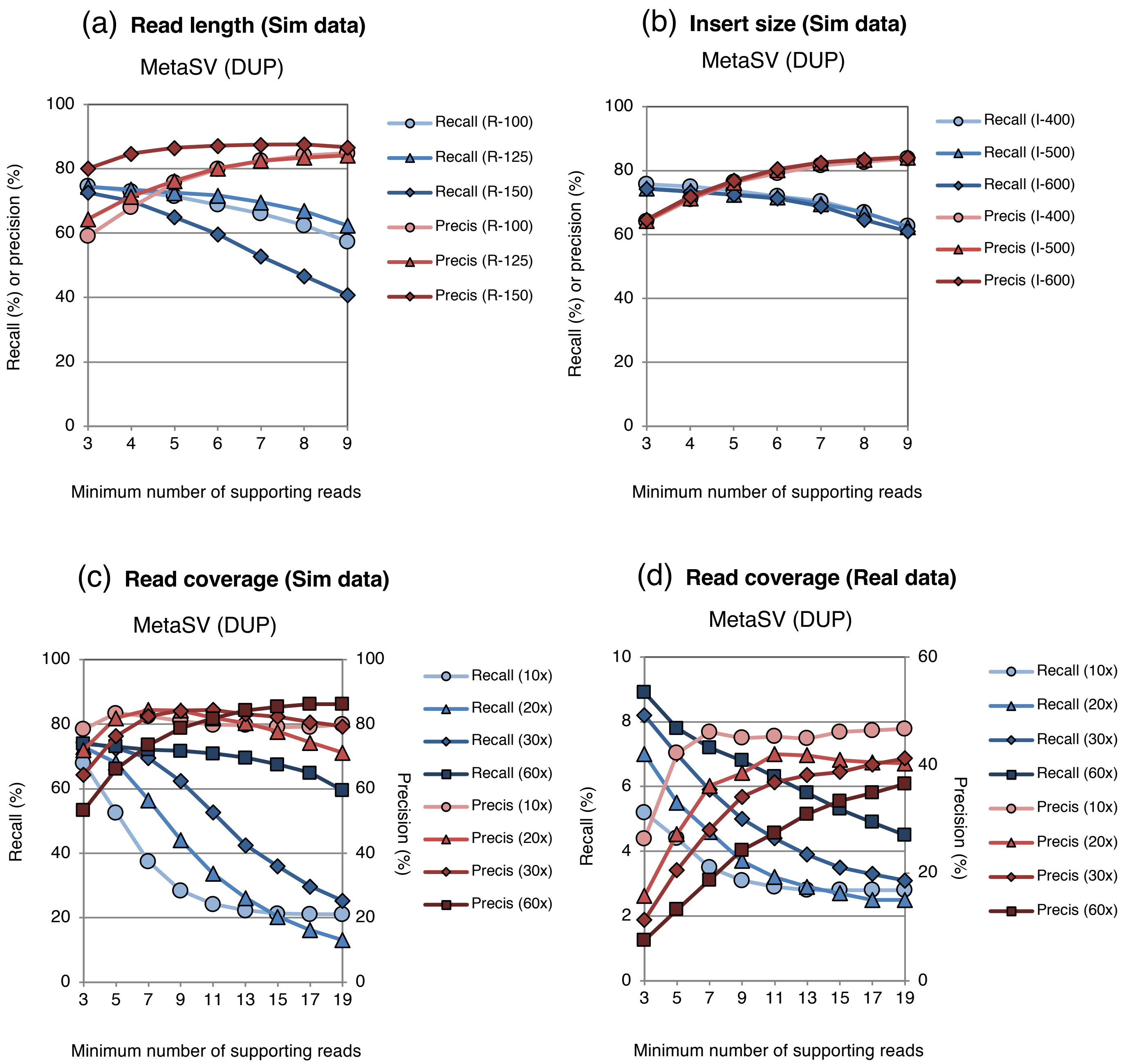

Figure S13-AB. Results for DUP calling using MetaSV. DUPs were called using MetaSV with the simulated data with different scales of read length (**a**), insert size (**b**), or read coverage (**c**) or with the real data with different scales of read coverage (**d**). It should be noted that the scales of the recall and precision were different in (c) and (d). The data are represented as in Figure S13-A.

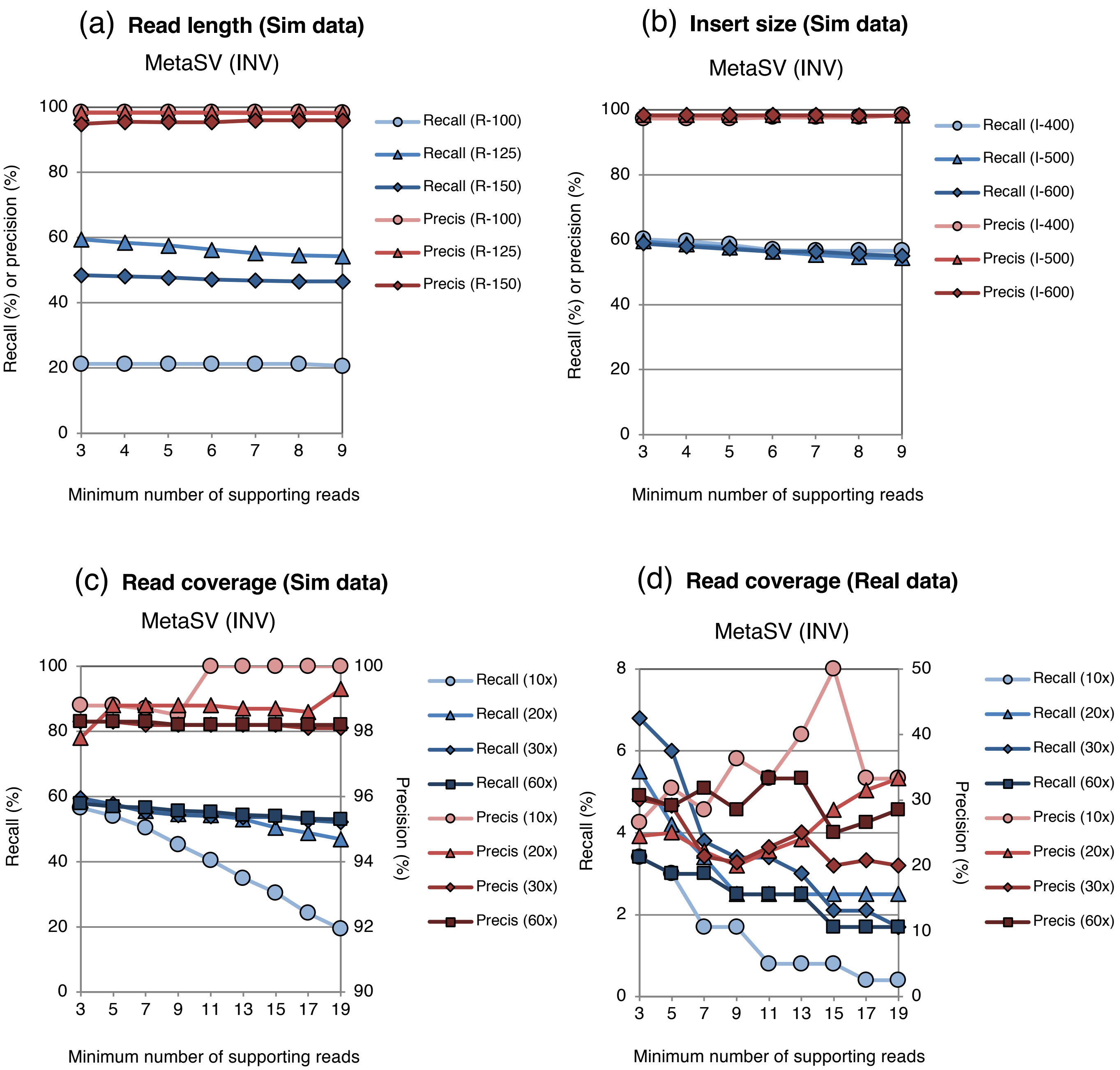

Figure S13-AC. Results for INV calling using MetaSV. INVs were called using MetaSV with the simulated data with different scales of read length (**a**), insert size (**b**), or read coverage (**c**) or with the real data with different scales of read coverage (**d**). It should be noted that the scales of the recall and precision were different in (c) and (d). The data are represented as in Figure S13-A.

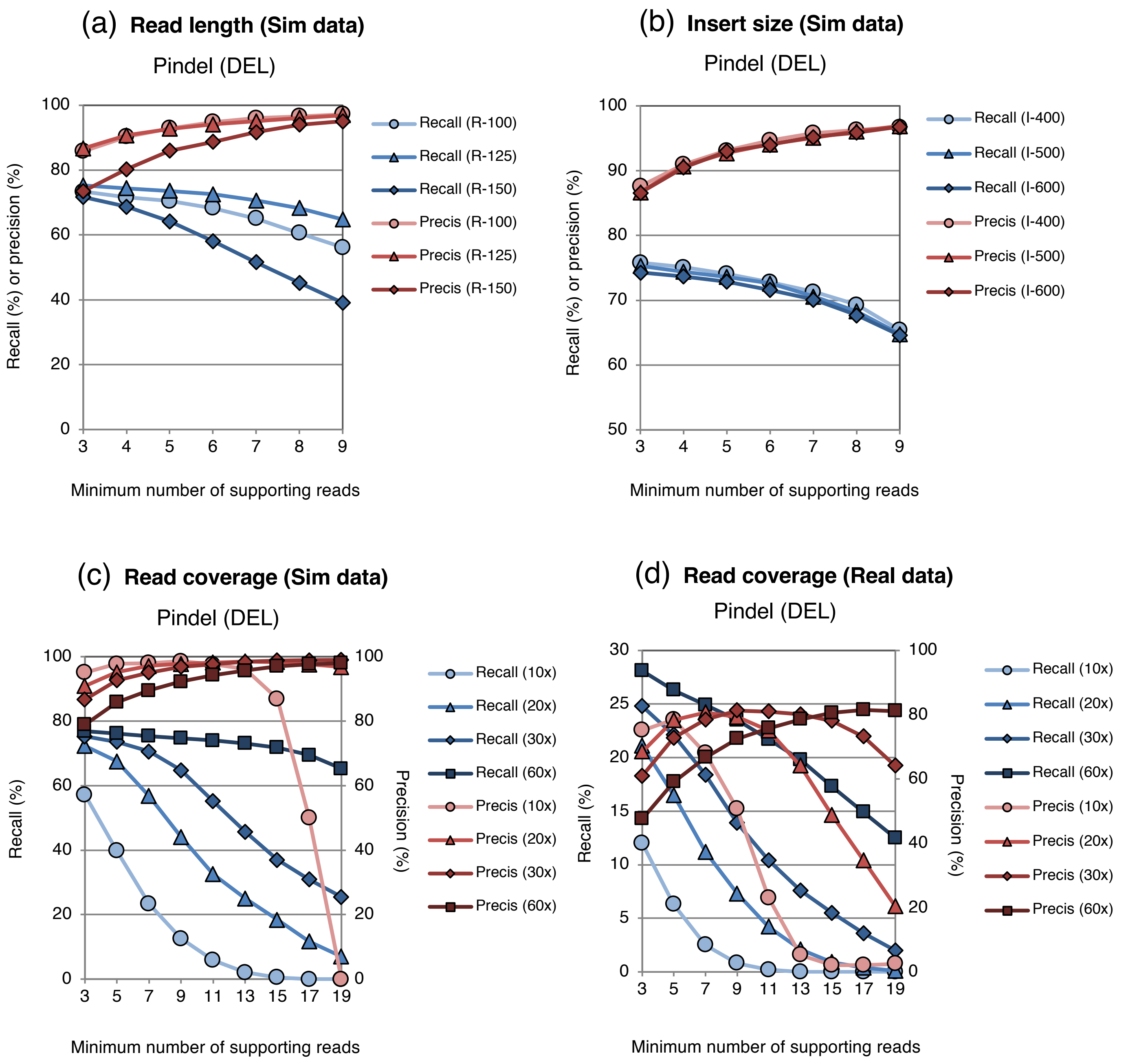

Figure S13-AD. Results for DEL calling using Pindel. DELs were called using Pindel with the simulated data with different scales of read length (**a**), insert size (**b**), or read coverage (**c**) or with the real data with different scales of read coverage (**d**). It should be noted that the scales of the recall and precision were different in (c) and (d). The data are represented as in Figure S13-A.

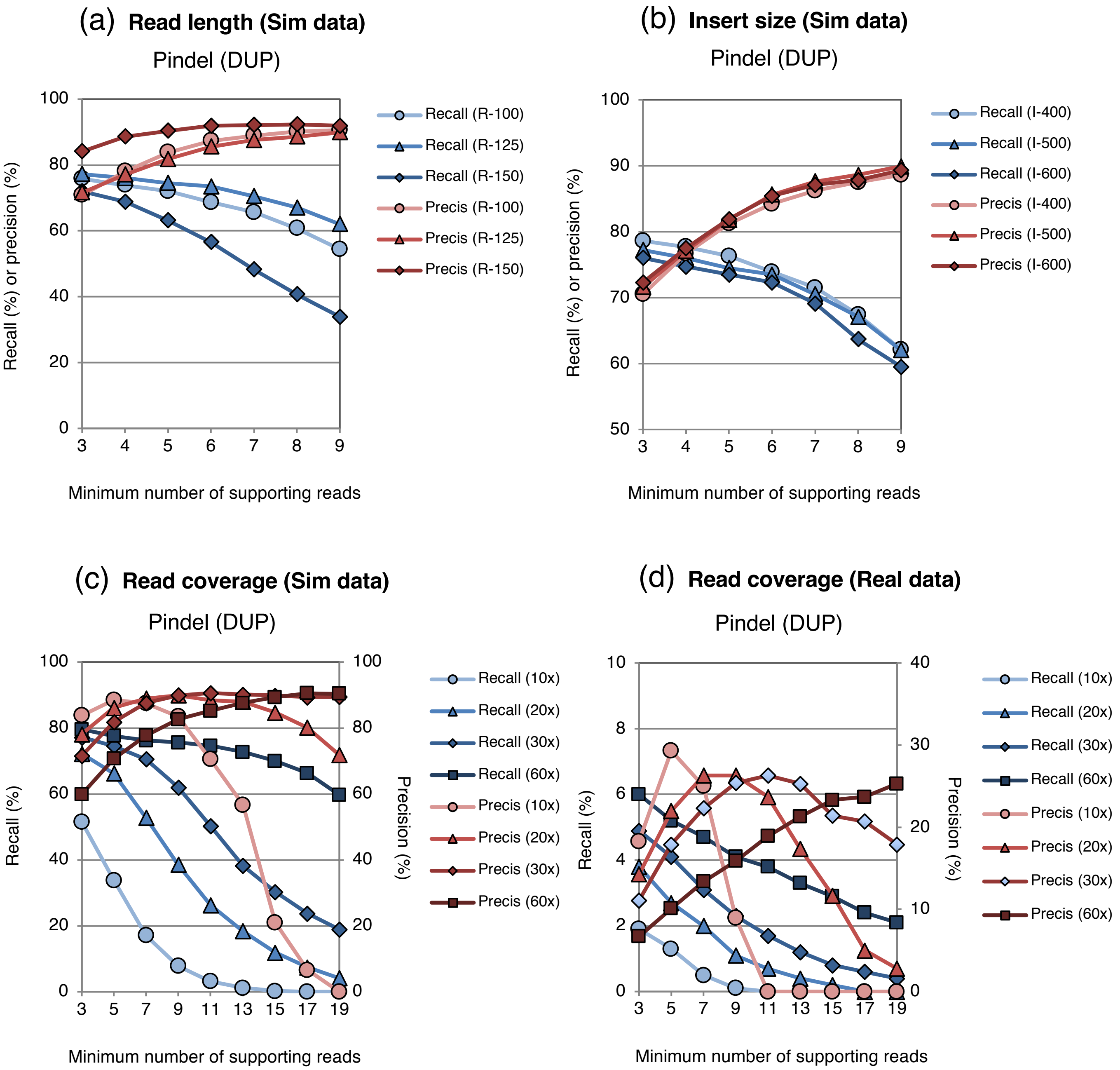

Figure S13-AE. Results for DUP calling using Pindel. DUPs were called using Pindel with the simulated data with different scales of read length (**a**), insert size (**b**), or read coverage (**c**) or with the real data with different scales of read coverage (**d**). It should be noted that the scales of the recall and precision were different in (c) and (d). The data are represented as in Figure S13-A.

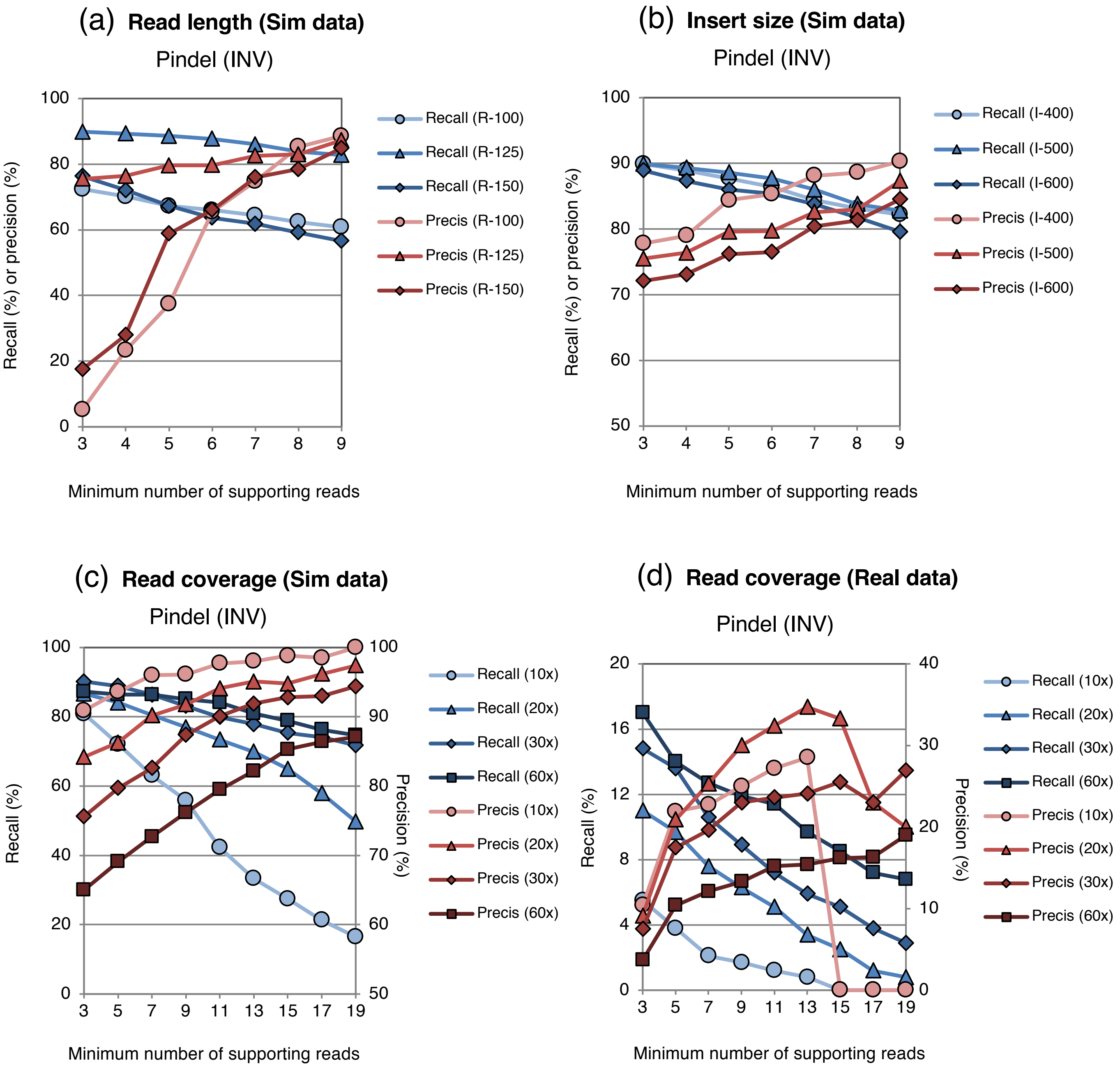

Figure S13-AF. Results for INV calling using Pindel. INVs were called using Pindel with the simulated data with different scales of read length (**a**), insert size (**b**), or read coverage (**c**) or with the real data with different scales of read coverage (**d**). It should be noted that the scales of the recall and precision were different in (c) and (d). The data are represented as in Figure S13-A.

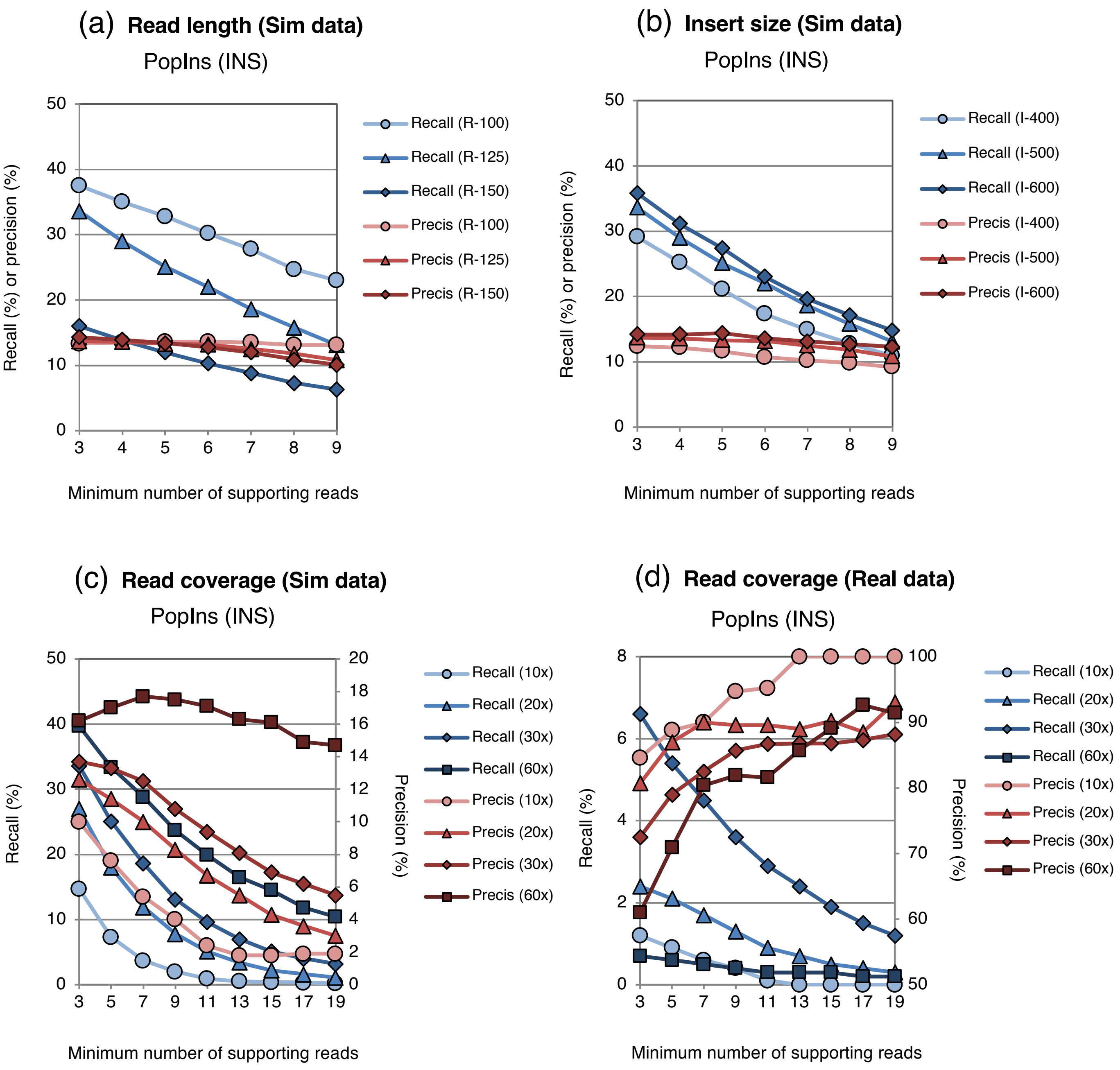

Figure S13-AG. Results for INS calling using PopIns. INSs were called using Popins with the simulated data with different scales of read length (**a**), insert size (**b**), or read coverage (**c**) or with the real data with different scales of read coverage (**d**). It should be noted that the scales of the recall and precision were different in (c) and (d). The data are represented as in Figure S13-A.

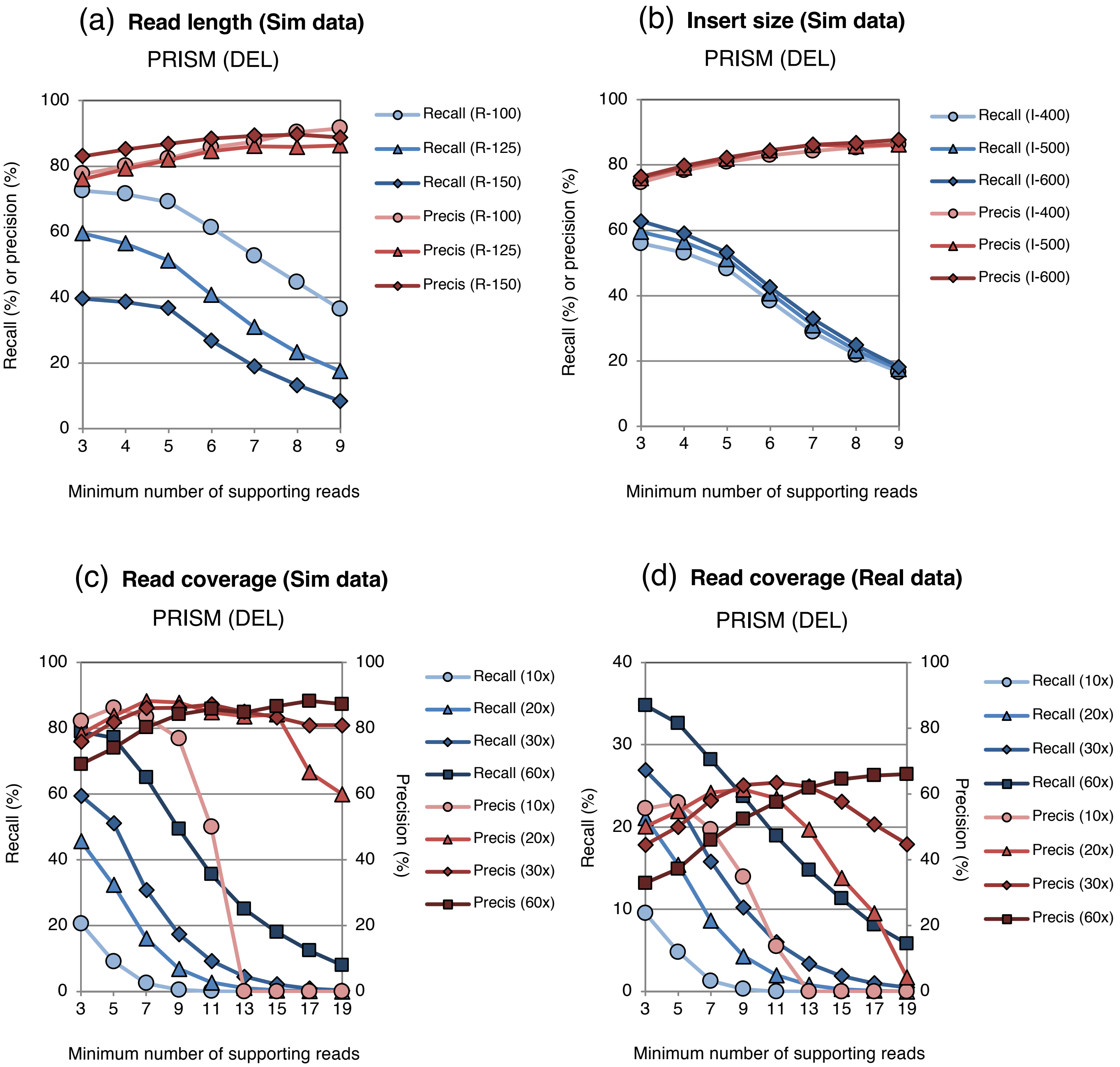

Figure S13-AH. Results for DEL calling using PRISM. DELs were called using PRISM with the simulated data with different scales of read length (**a**), insert size (**b**), or read coverage (**c**) or with the real data with different scales of read coverage (**d**). It should be noted that the scales of the recall and precision were different in (c) and (d). The data are represented as in Figure S13-A.

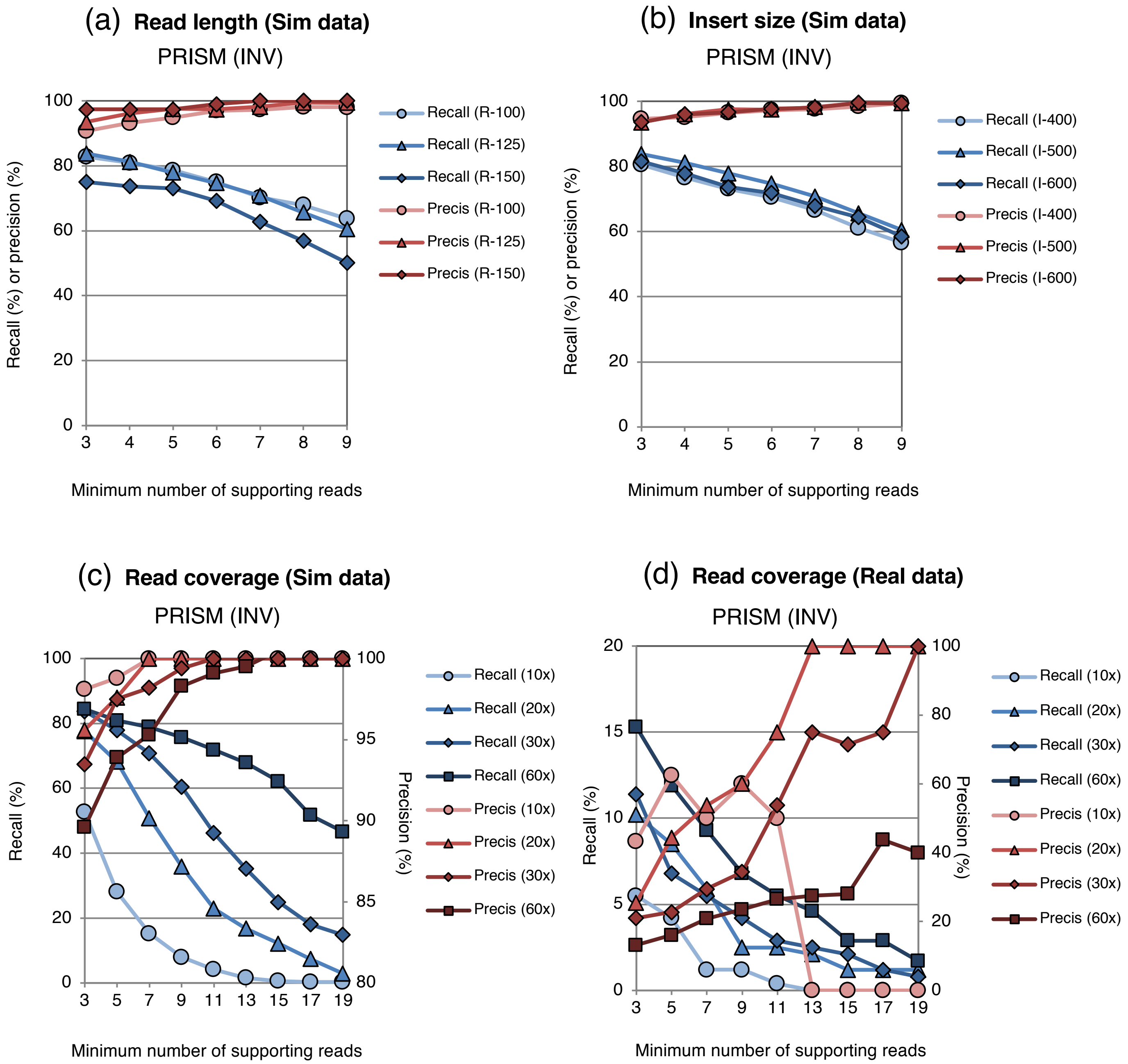

Figure S13-AI. Results for INV calling using PRISM. INVs were called using PRISM with the simulated data with different scales of read length (**a**), insert size (**b**), or read coverage (**c**) or with the real data with different scales of read coverage (**d**). It should be noted that the scales of the recall and precision were different in (c) and (d). The data are represented as in Figure S13-A.

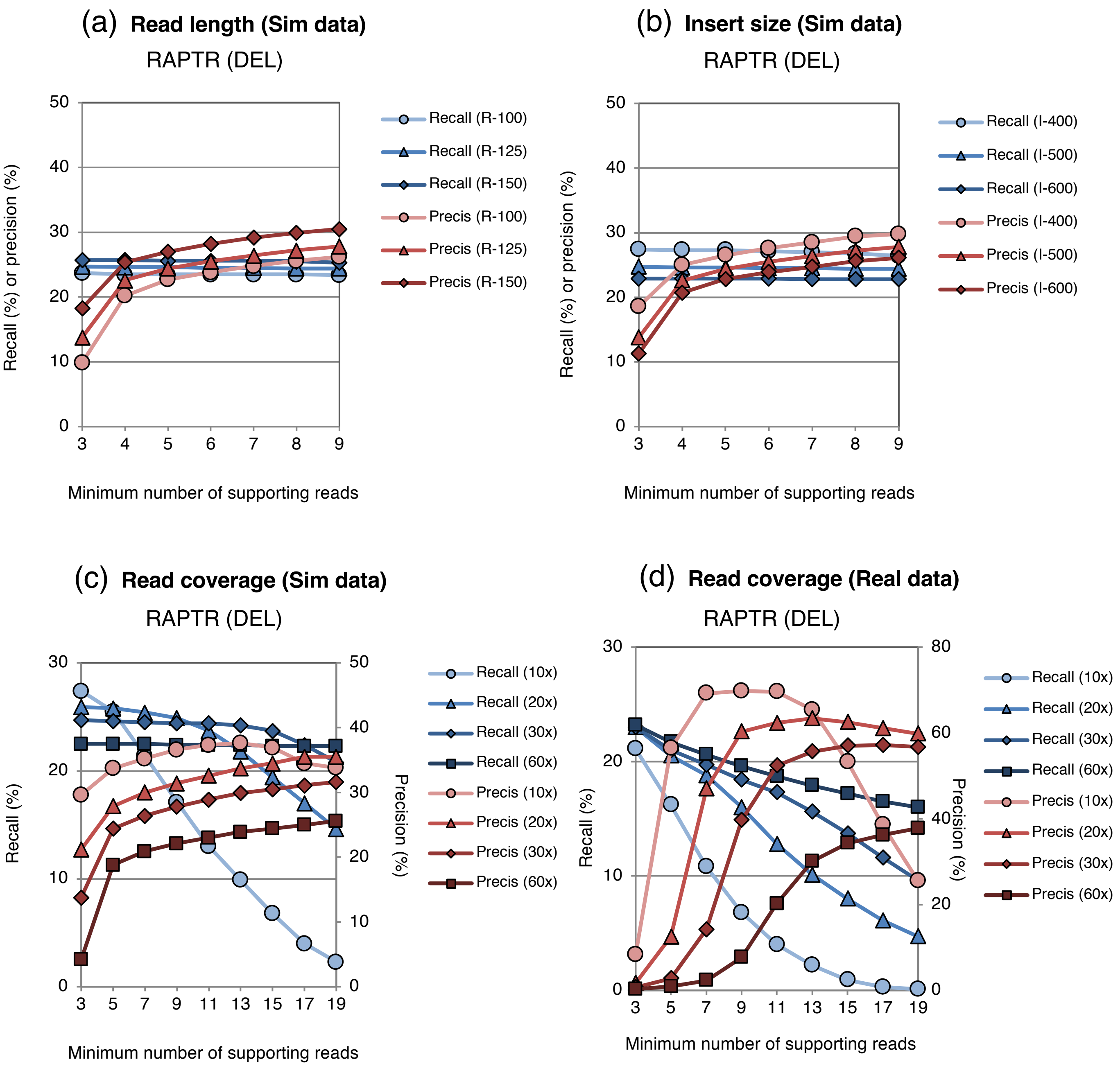

Figure S13-AJ. Results for DEL calling using RAPTR. DELs were called using RAPTR with the simulated data with different scales of read length (**a**), insert size (**b**), or read coverage (**c**) or with the real data with different scales of read coverage (**d**). It should be noted that the scales of the recall and precision were different in (c) and (d). The data are represented as in Figure S13-A.

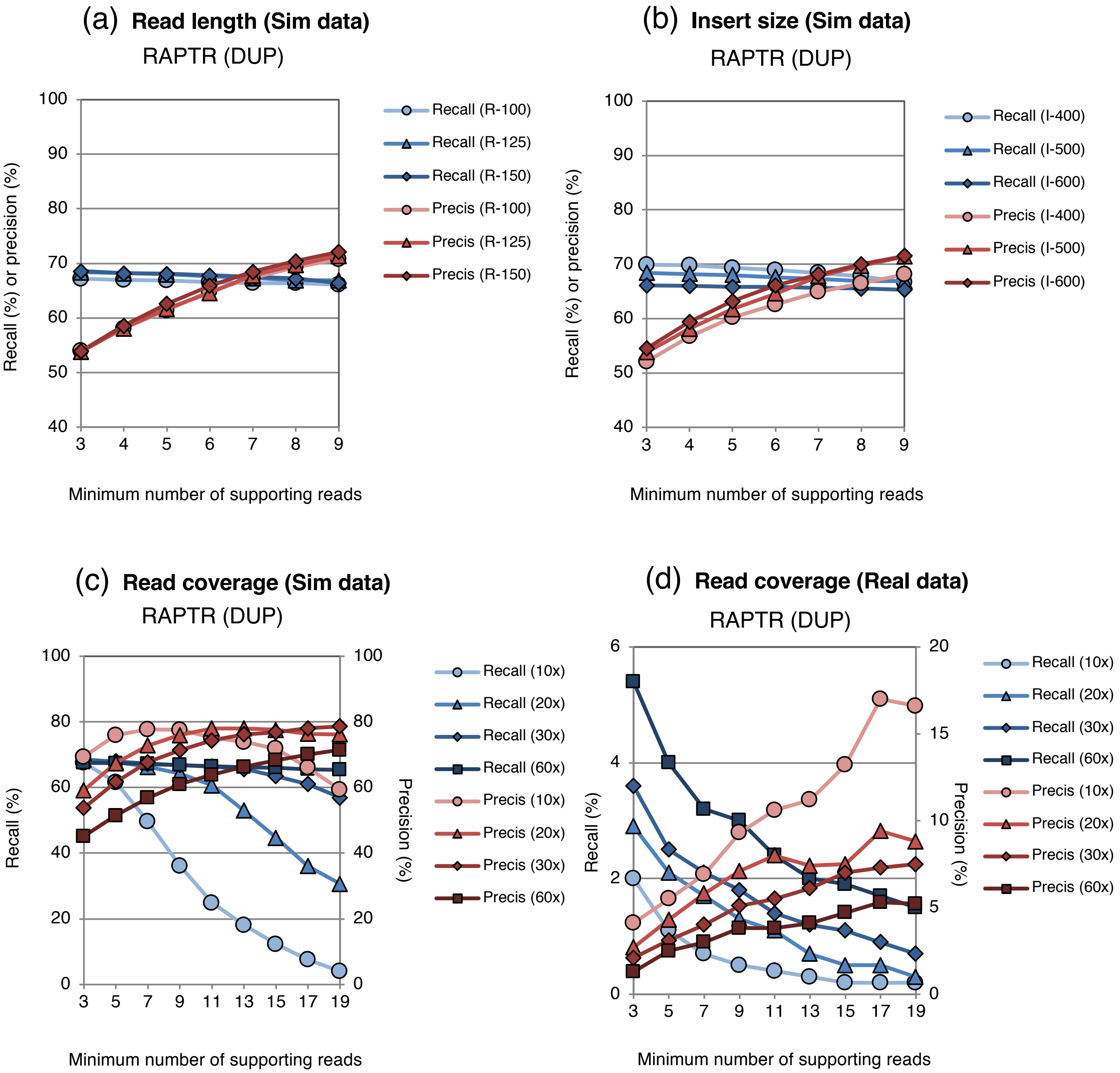

Figure S13-AK. Results for DUP calling using RAPTR. DUPs were called using RAPTR with the simulated data with different scales of read length (**a**), insert size (**b**), or read coverage (**c**) or with the real data with different scales of read coverage (**d**). It should be noted that the scales of the recall and precision were different in (c) and (d). The data are represented as in Figure S13-A.

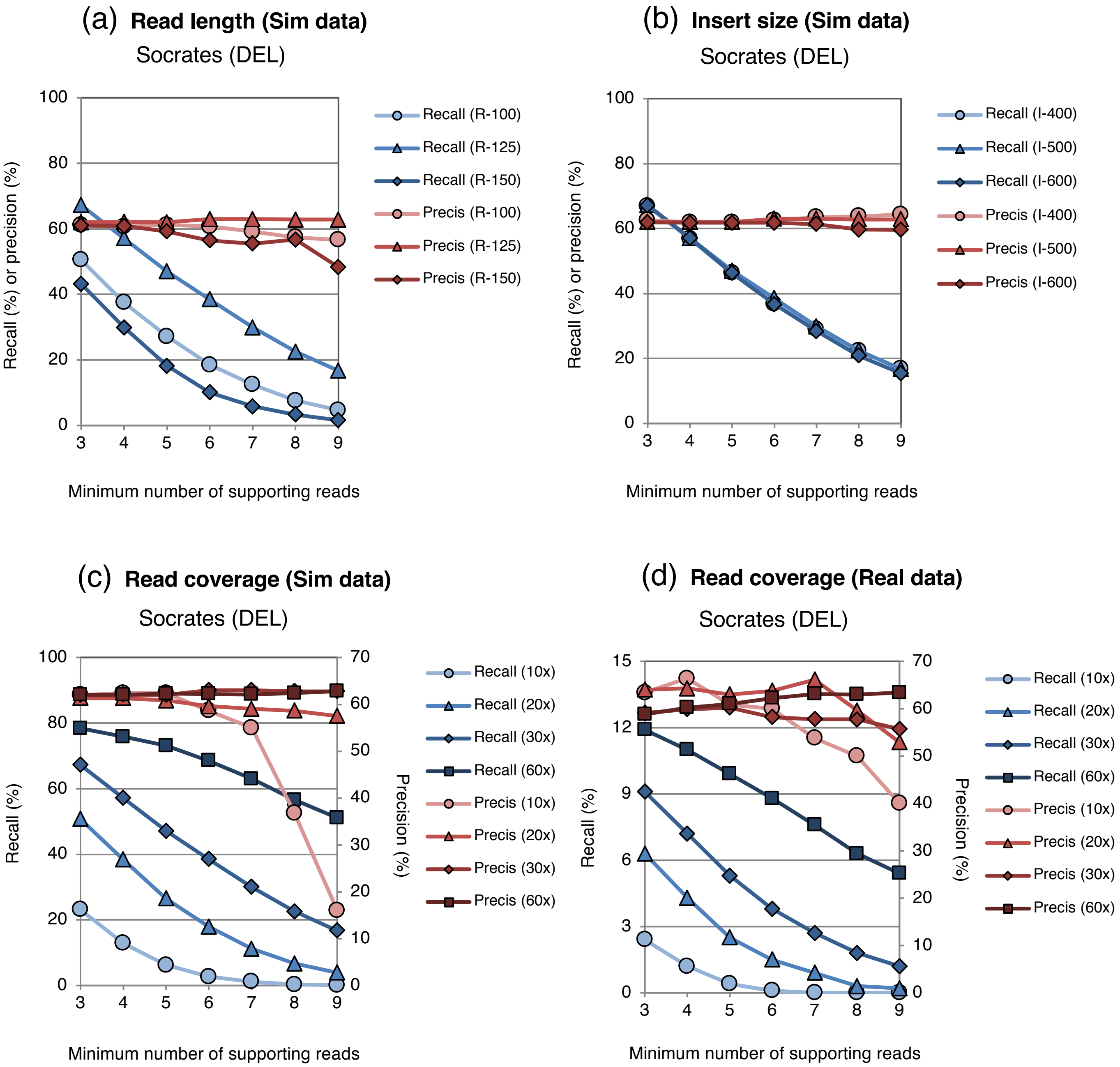

Figure S13-AL. Results for DEL calling using Socrates. DELs were called using Socrates with the simulated data with different scales of read length (**a**), insert size (**b**), or read coverage (**c**) or with the real data with different scales of read coverage (**d**). It should be noted that the scales of the recall and precision were different in (c) and (d). The data are represented as in Figure S13-A.

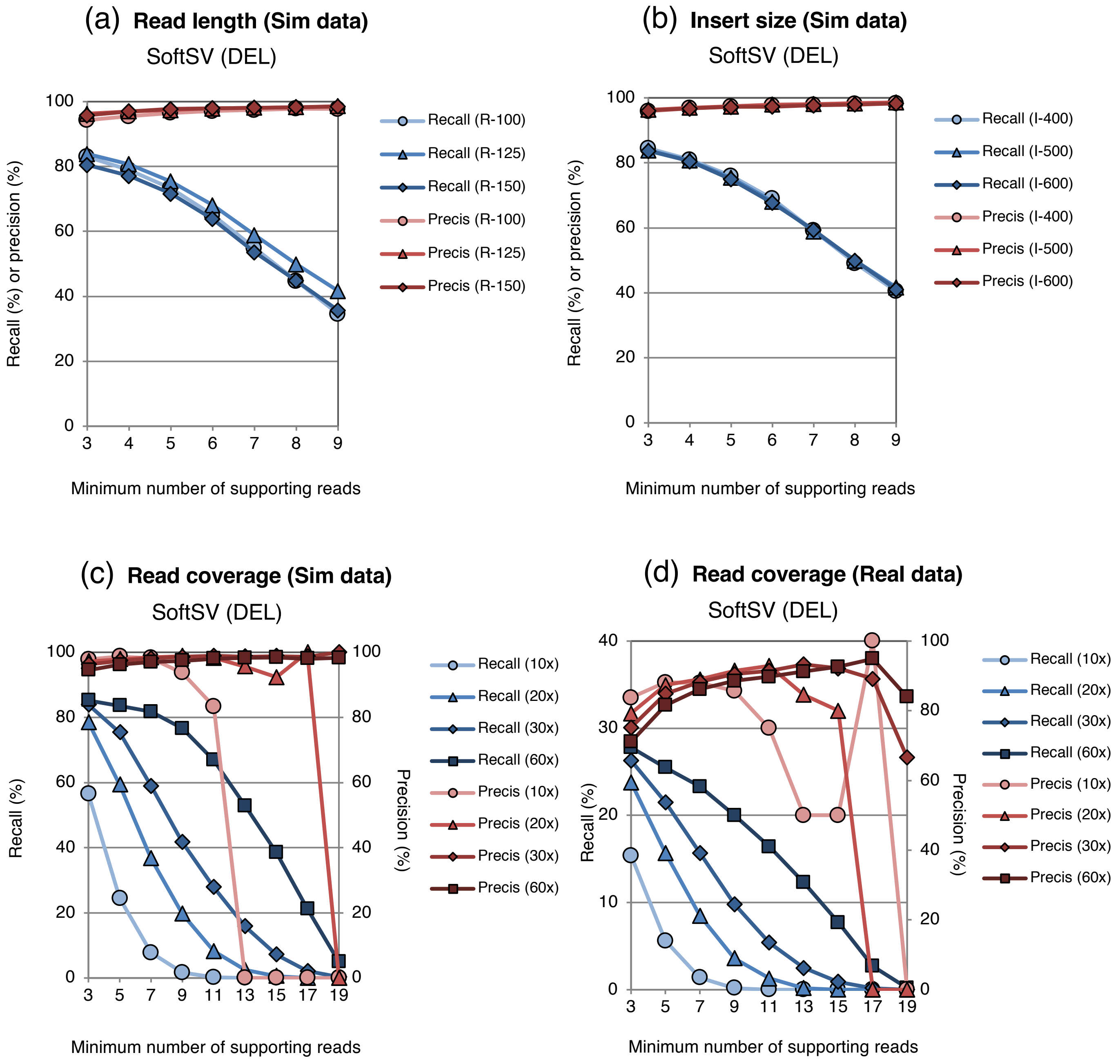

Figure S13-AM. Results for DEL calling using SoftSV. DELs were called using SoftSV with the simulated data with different scales of read length (**a**), insert size (**b**), or read coverage (**c**) or with the real data with different scales of read coverage (**d**). It should be noted that the scales of the recall and precision were different in (c) and (d). The data are represented as in Figure S13-A.

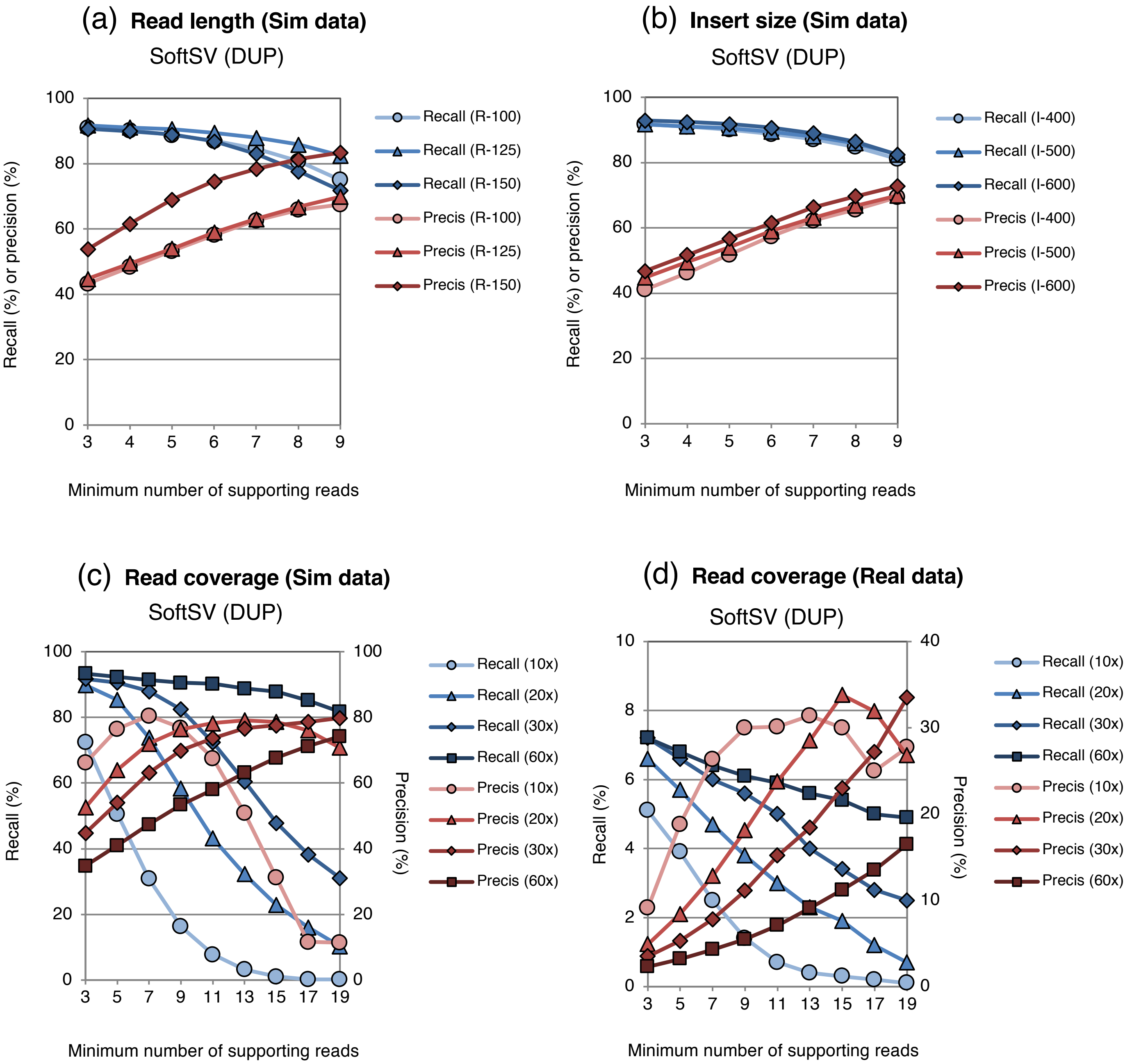

Figure S13-AN. Results for DUP calling using SoftSV. DUPs were called using SoftSV with the simulated data with different scales of read length (**a**), insert size (**b**), or read coverage (**c**) or with the real data with different scales of read coverage (**d**). It should be noted that the scales of the recall and precision were different in (c) and (d). The data are represented as in Figure S13-A.

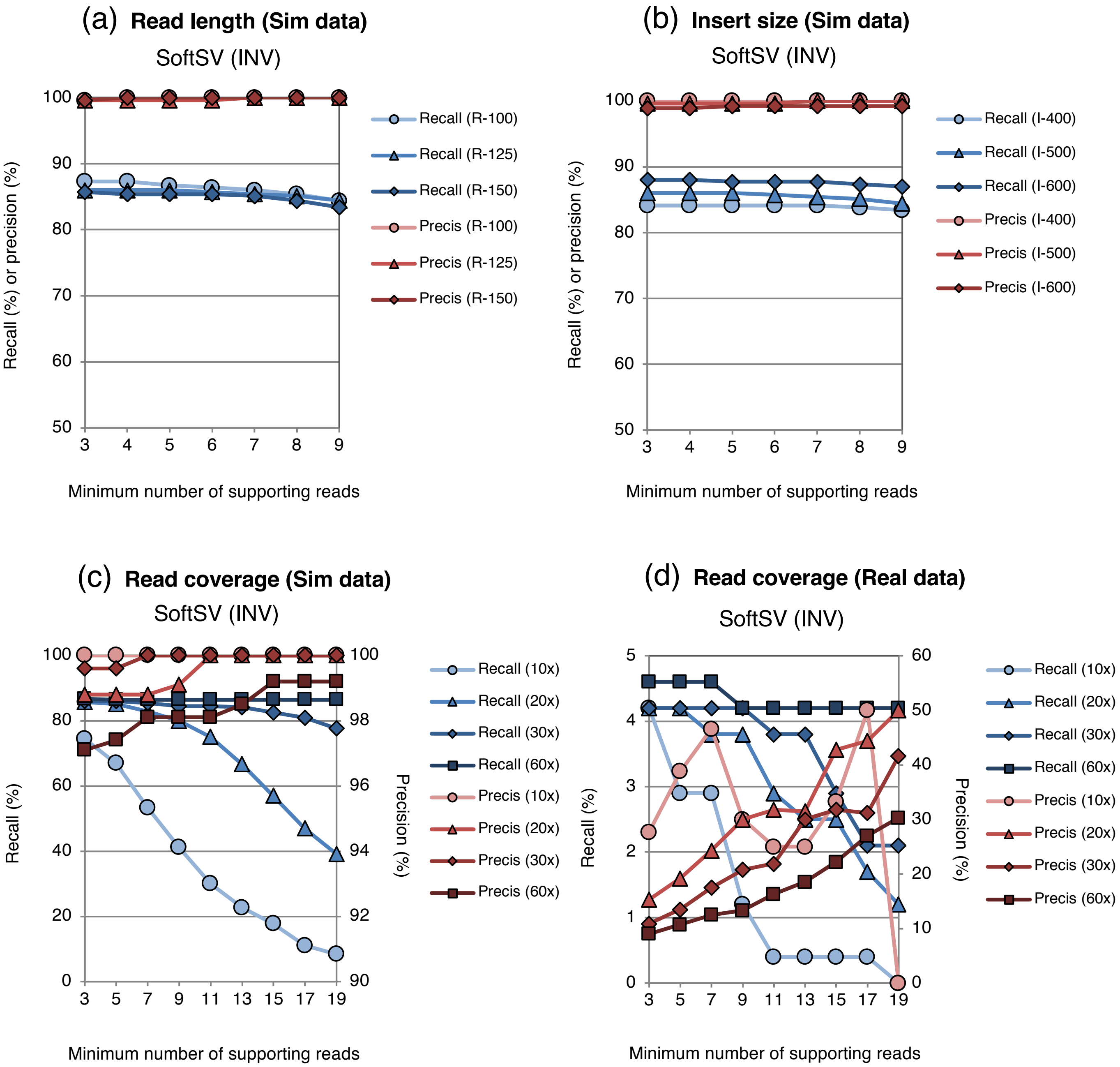

Figure S13-AO. Results for INV calling using SoftSV. INVs were called using SoftSV with the simulated data with different scales of read length (**a**), insert size (**b**), or read coverage (**c**) or with the real data with different scales of read coverage (**d**). It should be noted that the scales of the recall and precision were different in (c) and (d). The data are represented as in Figure S13-A.

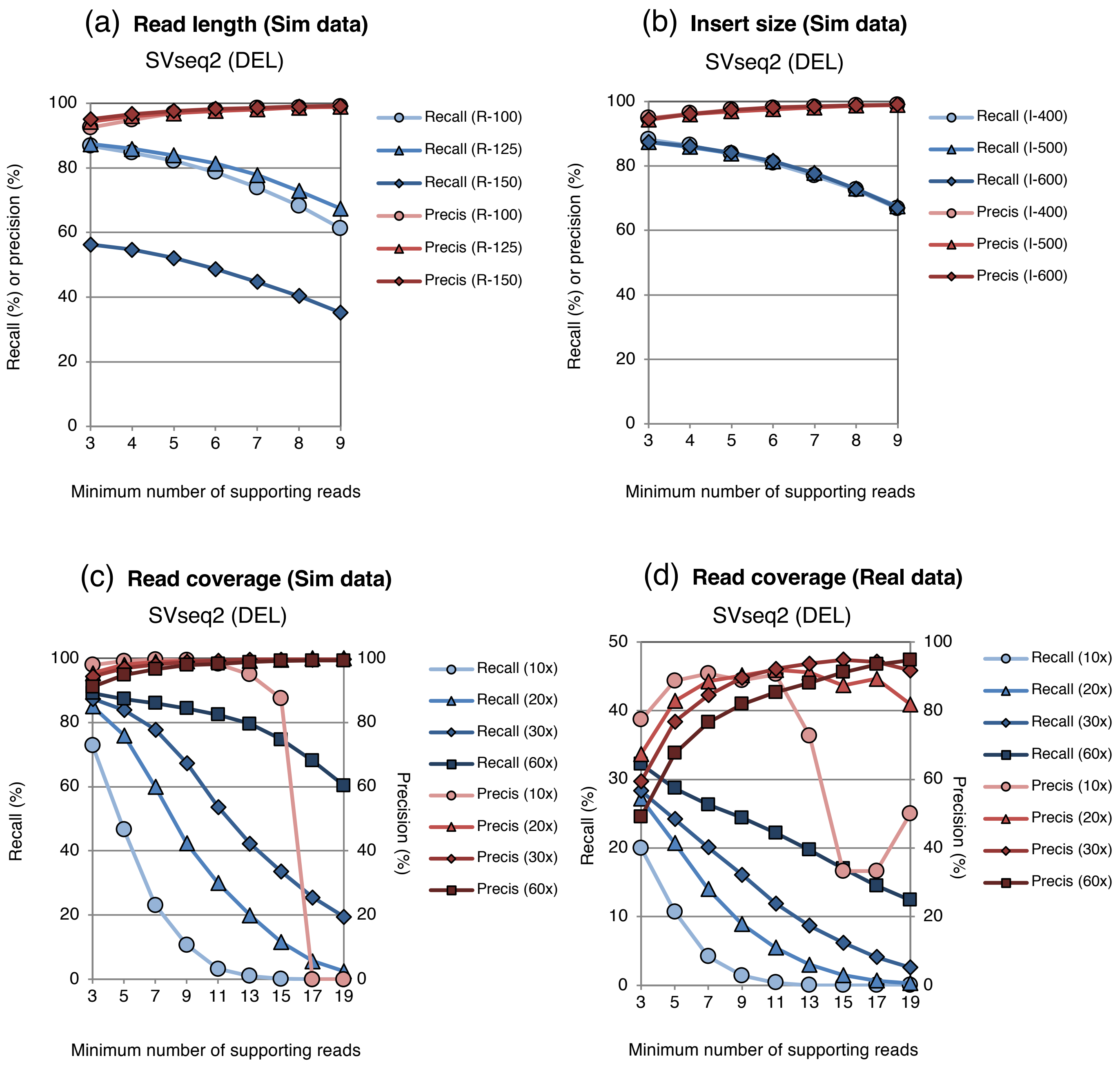

Figure S13-AP. Results for DEL calling using SVseq2. DELs were called using SVseq2 with the simulated data with different scales of read length (**a**), insert size (**b**), or read coverage (**c**) or with the real data with different scales of read coverage (**d**). It should be noted that the scales of the recall and precision were different in (c) and (d). The data are represented as in Figure S13-A.

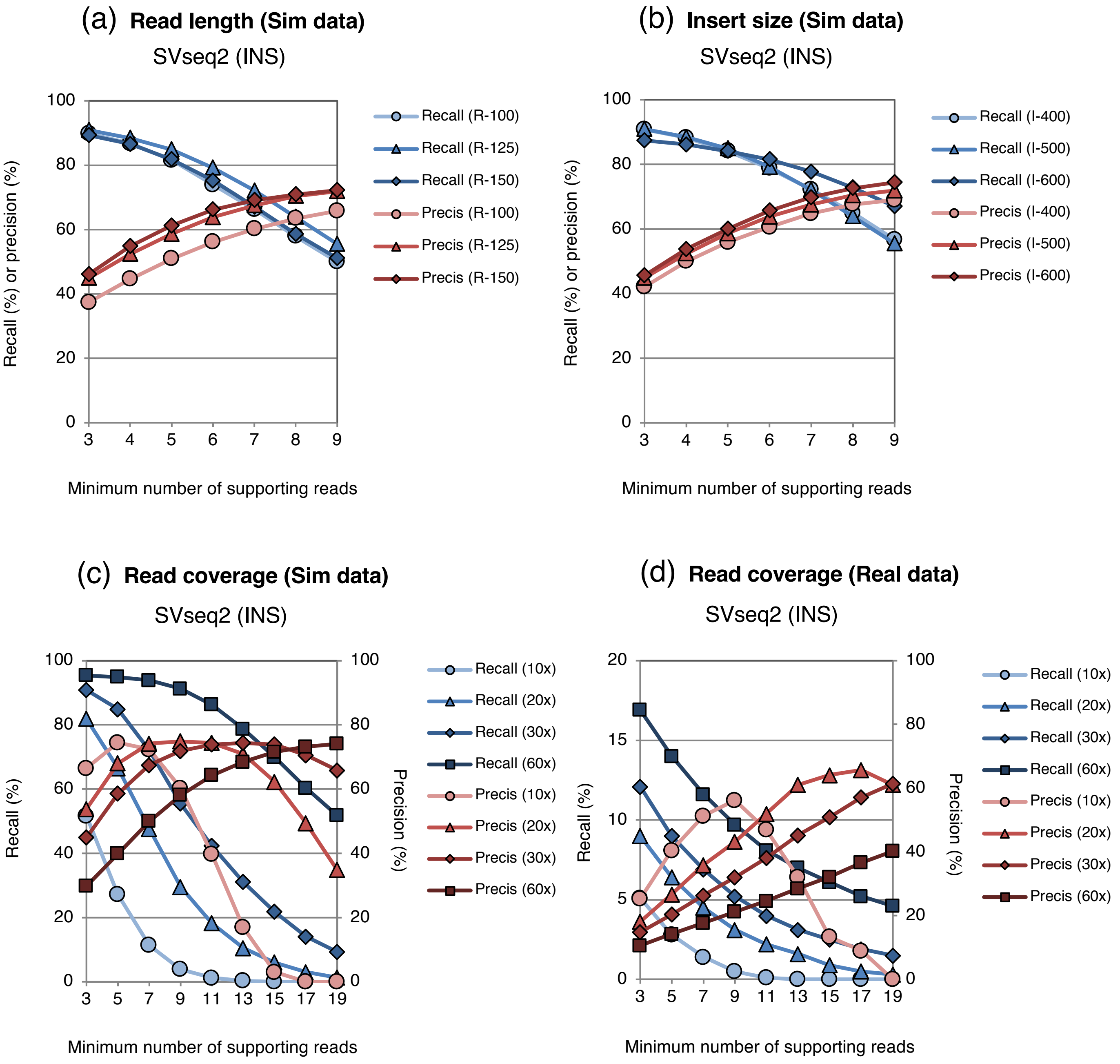

Figure S13-AQ. Results for INS calling using SVseq2. INSs were called using SVseq2 with the simulated data with different scales of read length (**a**), insert size (**b**), or read coverage (**c**) or with the real data with different scales of read coverage (**d**). It should be noted that the scales of the recall and precision were different in (c) and (d). The data are represented as in Figure S13-A.

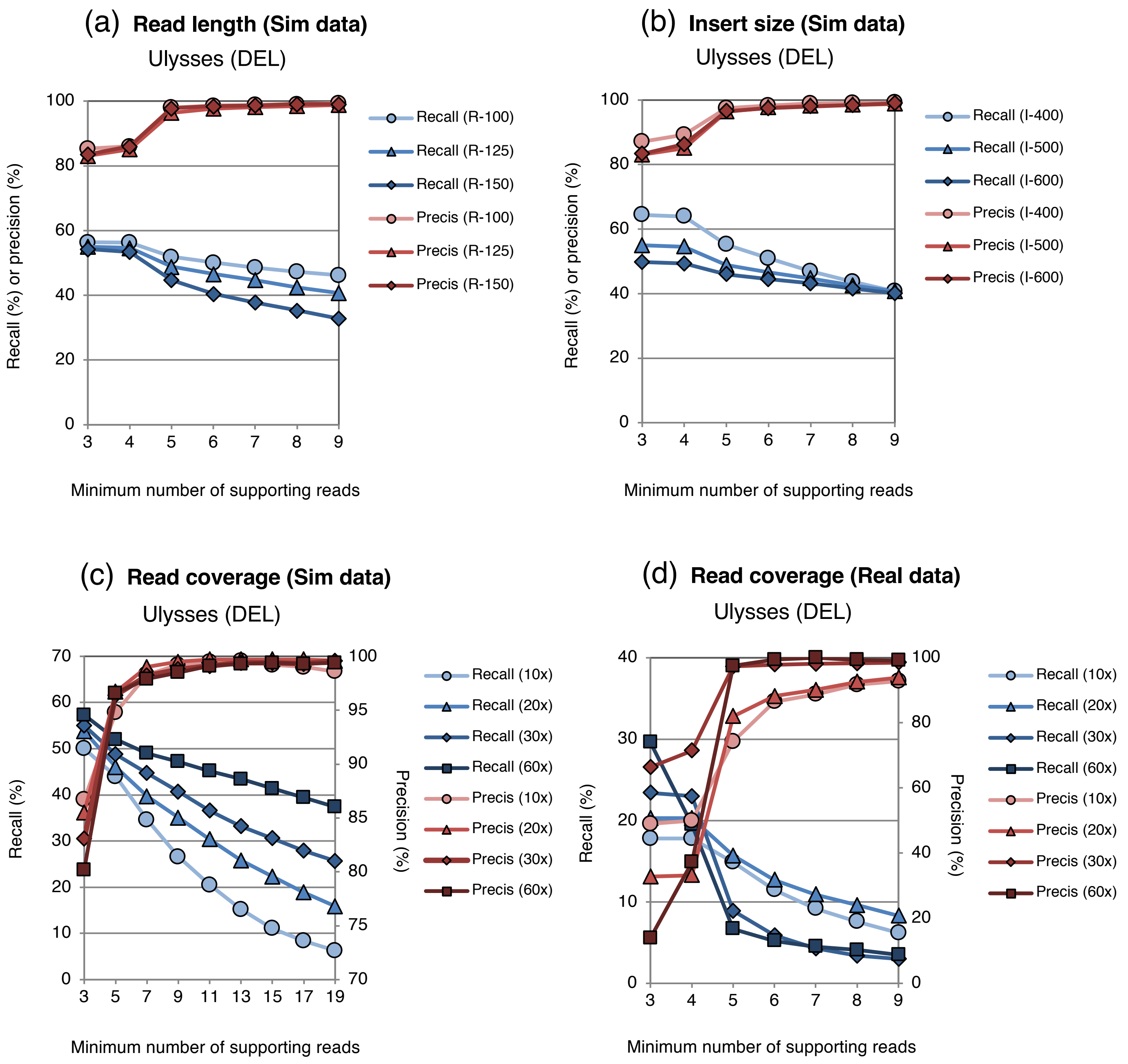

Figure S13-AR. Results for DEL calling using Ulysses. DELs were called using Ulysses with the simulated data with different scales of read length (**a**), insert size (**b**), or read coverage (**c**) or with the real data with different scales of read coverage (**d**). It should be noted that the scales of the recall and precision were different in (c) and (d). The data are represented as in Figure S13-A.

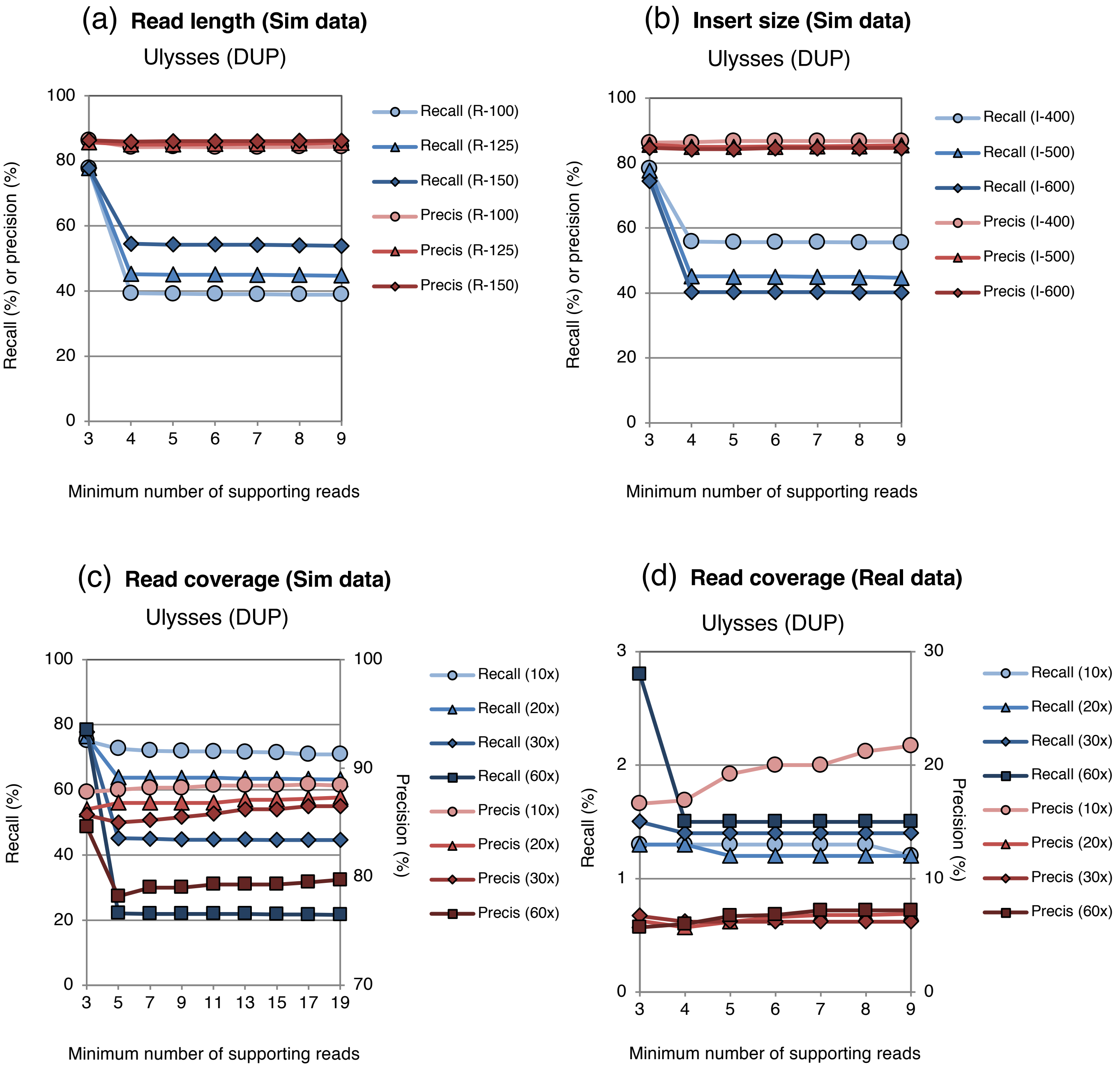

Figure S13-AS. Results for DUP calling using Ulysses. DUPs were called using Ulysses with the simulated data with different scales of read length (**a**), insert size (**b**), or read coverage (**c**) or with the real data with different scales of read coverage (**d**). It should be noted that the scales of the recall and precision were different in (c) and (d). The data are represented as in Figure S13-A.

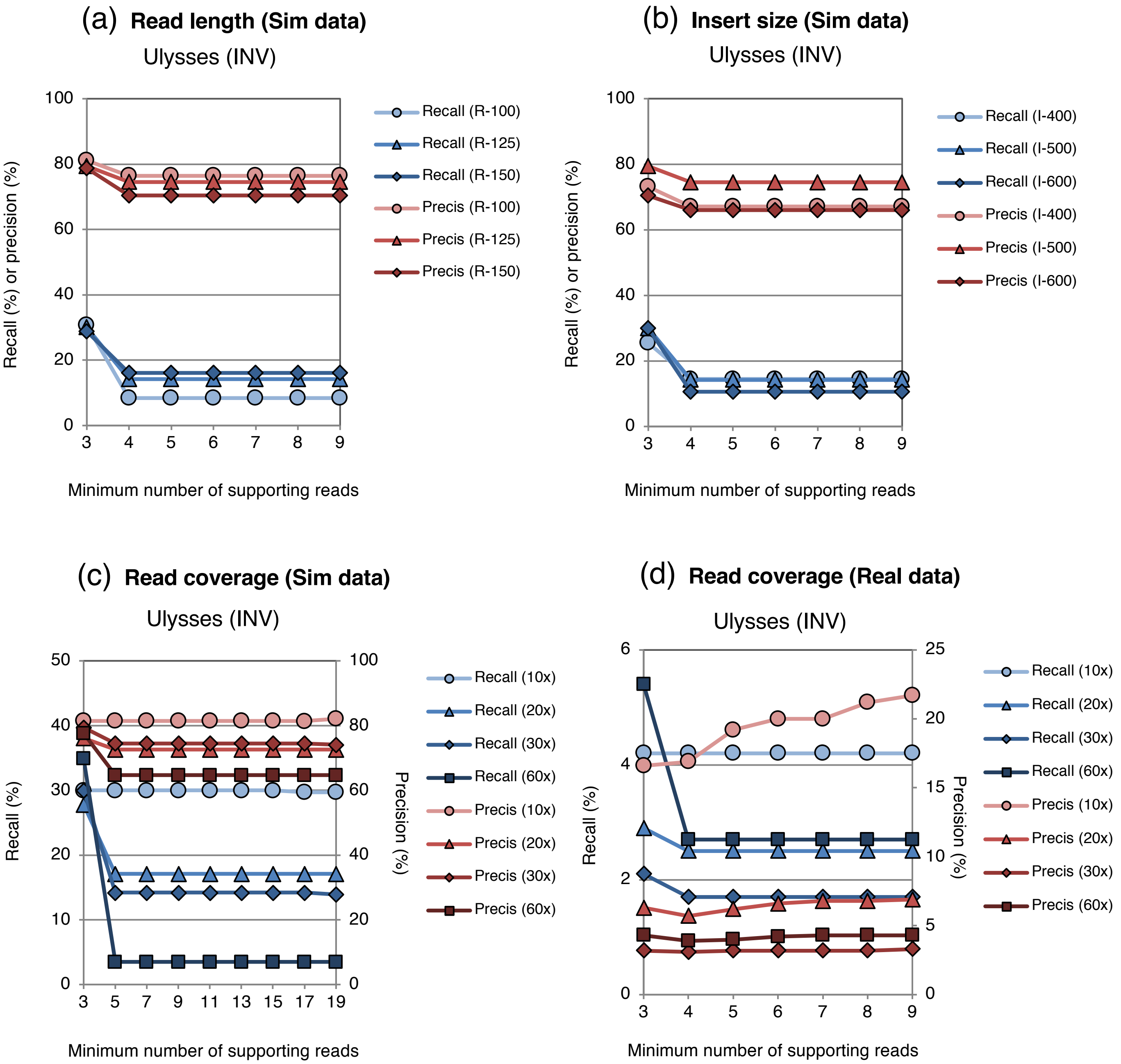

Figure S13-AT. Results for INV calling using Ulysses. INVs were called using Ulysses with the simulated data with different scales of read length (**a**), insert size (**b**), or read coverage (**c**) or with the real data with different scales of read coverage (**d**). It should be noted that the scales of the recall and precision were different in (c) and (d). The data are represented as in Figure S13-A.

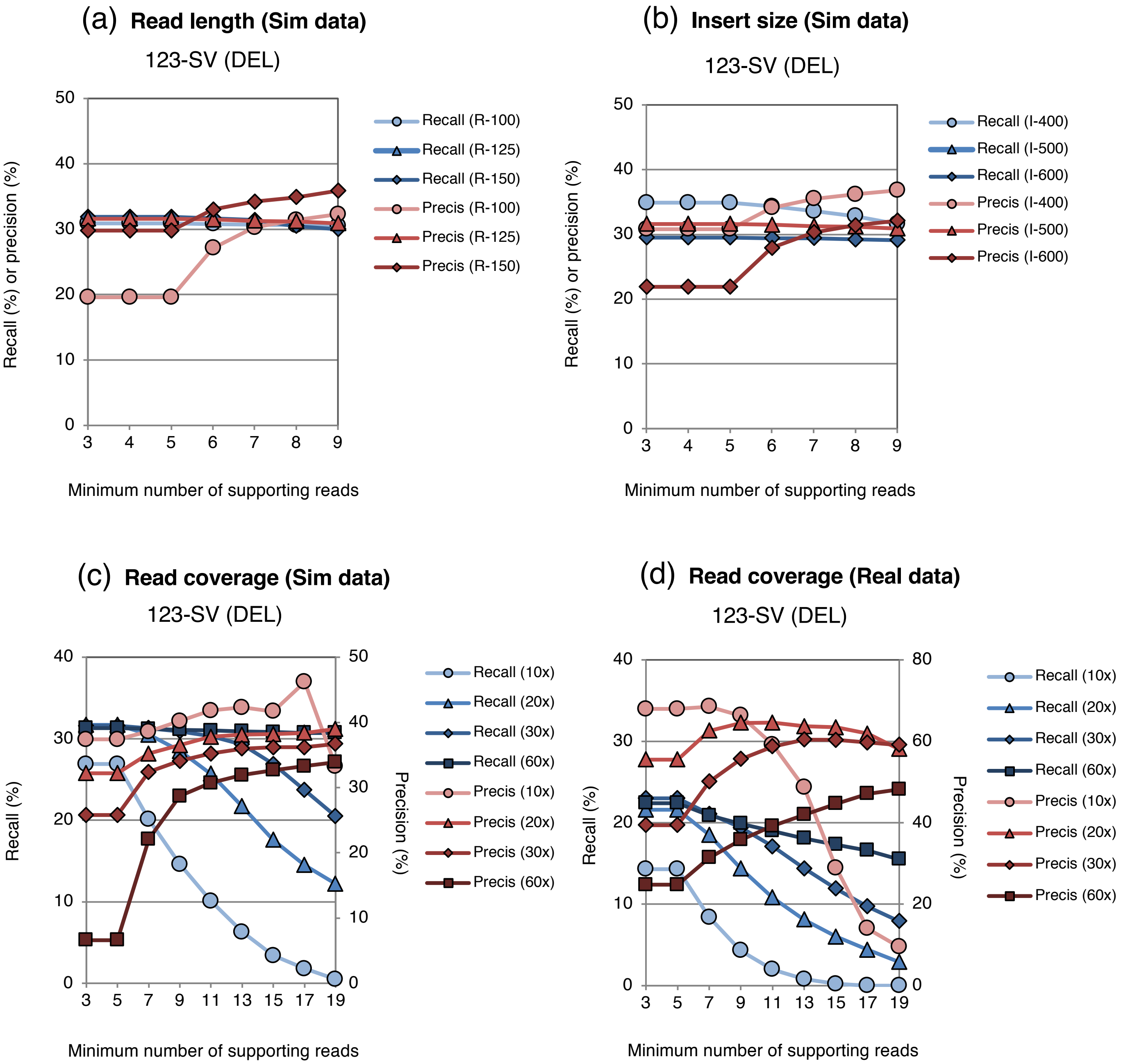

Figure S13-AU. Results for DEL calling using 1-2-3-SV. DELs were called using 1-2-3-SV with the simulated data with different scales of read length (**a**), insert size (**b**), or read coverage (**c**) or with the real data with different scales of read coverage (**d**). It should be noted that the scales of the recall and precision were different in (c) and (d). The data are represented as in Figure S13-A.

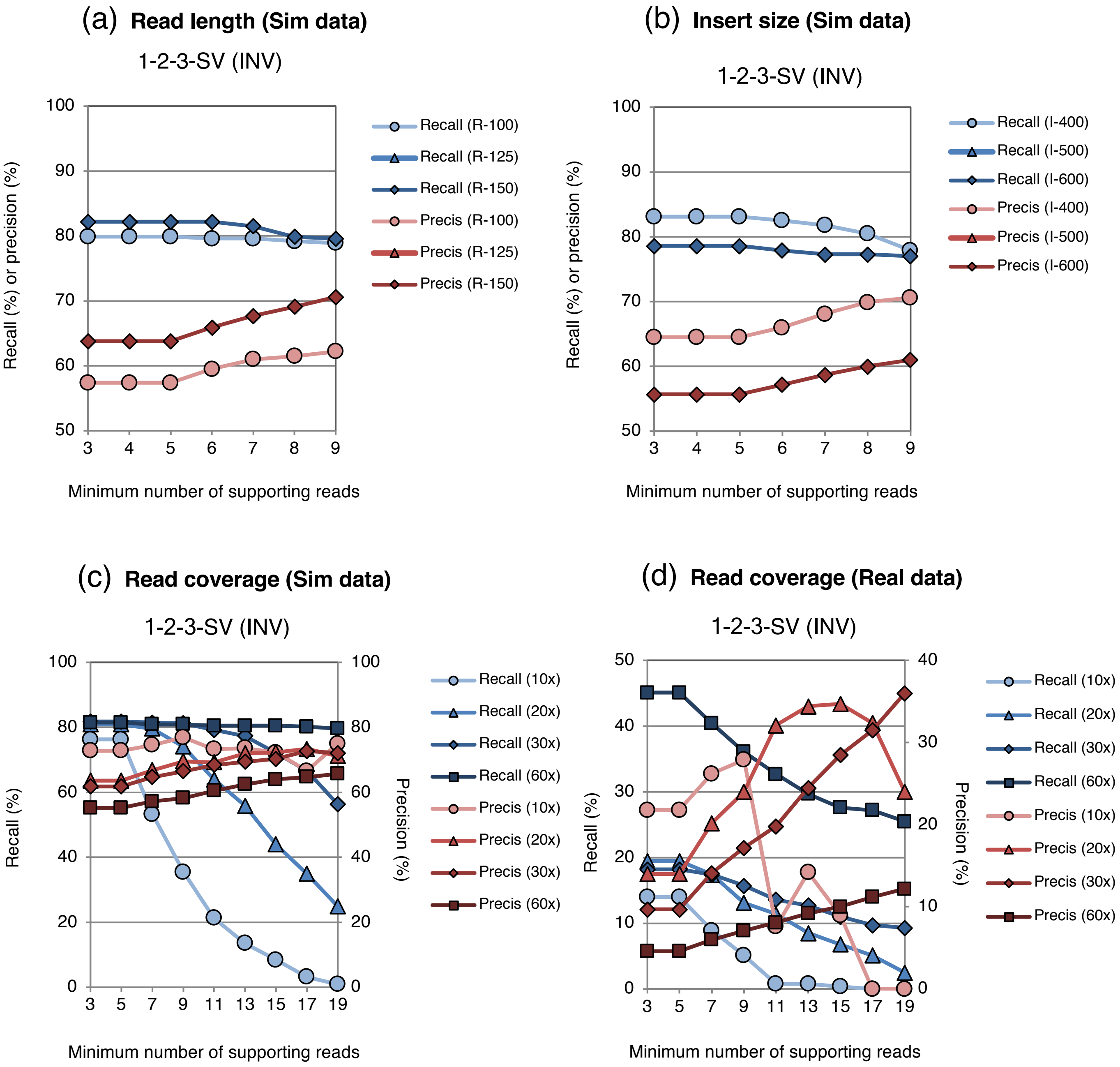

Figure S13-AV. Results for INV calling using 1-2-3-SV. INVs were called using 1-2-3-SV with the simulated data with different scales of read length (**a**), insert size (**b**), or read coverage (**c**) or with the real data with different scales of read coverage (**d**). It should be noted that the scales of the recall and precision were different in (c) and (d). The data are represented as in Figure S13-A.
