# Supplementary material for: Comprehensive evaluation of structural variation detection algorithms for whole genome sequencing
Source: Genome Biol. 2019 Jun 3;20:117. doi: 10.1186/s13059-019-1720-5 (PMC6547561; doi:10.1186/s13059-019-1720-5)
Supplement: Supplementary file 4 — Supplementary methods. SV calling processes for 69 SV detection algorithms used in this study. (PDF 430 kb) [file 13059_2019_1720_MOESM4_ESM.pdf]

Supplementary Methods

SV calling processes for 69 SV detection algorithms used in this study

1. SV calling

Examples of the commands and options used for the 69 algorithms used in this study are described below. For several specific algorithms, the procedures for the preparation of input files and the definition of reads supporting SVs are explained. Unless otherwise stated, the input data (NA78.bam) are the alignment data that was generated with the bwa 'mem' command using the hs37d5 reference and the NA12878 WGS data. The hs37d5 reference (hs37d5.fa) contains 41.8 Mb decoy sequences comprising of 61 sequences. The NA12878 WGS data comprises 100 bp paired-end reads with a 320 bp insert size (SD: 80 bp) and with 30× coverage. For several algorithms, to run the algorithm in parallel to shorten the runtime, the reference and the alignment files were split for individual chromosomes. In the split bam file for a single chromosome, when the alignment of a mate read was assigned to other chromosomes, the mate read was considered as an unmapped read and the bam data, including the flag field, were modified. All algorithms and the commands are assumed to be installed under the $HOME/tool directory and to be set in PATH. The final output file for each algorithm was converted to a vcf format compatible with our evaluation scripts. This vcf format included a READS tag in the information field, which represented the number of reads supporting the called SV allele (RSS). For several algorithms, including read depth-based algorithms whose output files do not provide RSS, other values such as scores were converted to different scales and were used to mimic RSS. For INSs, when the output file did not contain the information for SV size, the size was assigned as 0 with a SVLEN tag in the information field. For genotyping-executable algorithms, the vcf file included the GT tag to specify genotypes (0/1 for heterozygous SVs, 1/1 for homozygous SVs, or ./. for unclear genotypes).

1.1. 1-2-3-SV

Run 1-2-3-SV:

The input bam file was split for individual chromosomes. The split bam file and an analysis name were specified in the 123SV.conf file, which was included in the package. The deletion, insertion, and inversion command of the 123SV2.pl script were sequentially executed for each chromosome with the config file to detect DELs, INSs, and INVs, respectively.

Convert output file:

The output files (*_deletion_* for DEL, *_insertion_* for INS, and *_inversion_* for INV) for each SV type and each chromosome were merged and converted to the vcf format. The first and the second breakpoints were determined with the mean values of the values indicated at the second and the third columns and the fifth and sixth columns, respectively. The smaller site of either the first or the second breakpoint was assigned to the breakpoint in the vcf file. The absolute value of the distance between the first and the second breakpoints was assigned to the SV size. The SV sizes for INS were fixed to 0. The values indicated at the ninth column were assigned to RSS. Finally the converted vcf files for each SV type were merged to a single vcf file.

Commands used:

[1] Estimate insert size

```
123SV1.pl 1
```

[2] Call SVs

```
123SV2.pl deletion
123SV2.pl insertion
123SV2.pl inversion
```

1.2. AS-GENESENG

Preparation of input files:

The input bam and reference fasta files were split for individual chromosomes. The split reference chromosome fasta file was further split with the createwindowsforwholechrosome.py command for output to 'Window' directory. The split bam chromosome file was sorted by read name with samtools (http://samtools.sorceforge.net). Mappability and GC content files were prepared with the commands, print_oligo.pl, bwa_nhits.pl, createwindowsforwholechrosome.py, and genCovariates.py, as described in the authors' guideline. SNP data from the input bam file was generated with samtools and the output was converted to an AS-GENSENG-compatible format. Taking as an example the data for chromosome1, the output files generated were Window/window.chrom1.bed, chr1.sn.bam, chr1.mappability.txt, and chr1.gc.txt, and chr1.snp.txt.

Run GENSENG:

For the data for each chromosome, extraction of allele-specific reads, generation of read count data, and run of the GENSENG command were sequentially executed.

Convert output file:

Output files (*_segment.dat) generated for each chromosome were merged and converted to a vcf file. The SV type was designated as DEL when the copy number indicated in the fifth column was less than 2. Otherwise, the SV type was designated as DUP. For DELs, when the copy numbers were 0 and 1, the genotypes were assigned to ‘1/1’ and ‘0/1’ in the vcf file, respectively. For DUPs, when the copy numbers were ≧ 4 and 3, the genotypes were assigned to ‘1/1’ and ‘0/1’ in the vcf file, respectively. The scores indicated in the seventh column were used to represent provisional numbers of RSS. When the scores were 0–1, 1.1–5, 5.1–10, 10.1–20, 20.1–30, and > 30.1, RSSs were assigned as 3, 4, 5, 6, 7, and 8, respectively. The sv types and RSSs were indicated in the information field of a vcf file with SVTYPE and READS tags, respectively.

Commands used:

As an example for chromosome 1

[1] Preparation of Covariates, Mappability, and GC content files

```
$HOME/tool/AS-GENSENG1.0.2/InHouseScripts/2CovariatesGeneration/print_oligo.pl hs37d5.chr1.fa
bwa aln hs37d5.chr1.fa chr1.fq > chr1.fq.bwa.sai
bwa samse hs37d5.chr1.fa chr1.fq.bwa.sai chr1.fq > chr1.fq.bwa.sam
bwa_nhits.pl 17 chr1.fq.bwa.sam
python
$HOME/tool/AS-GENSENG1.0.2/InHouseScripts/2CovariatesGeneration/createwindowsforwholechrosome.py 500 300 hs37d5.chr1.fa.fai OUT
python
$HOME/tool/AS-GENSENG1.0.2/InHouseScripts/2CovariatesGeneration/genCovariates.py OUT/window.chrom1.bed chr1.fq.bwa.samX0.txt >
covariates_chr1.txt
cat covariates_chr1.txt | sed 1d | cut -f2-5,7 > chr1.mappability.txt
cat covariates_chr1.txt | sed 1d | cut -f2-4,6,8 > chr1.gc.txt
```

[1] Extract allele specific reads

```
samtools sort –n NA78.chr1.bam –o NA78.chr1.rn.bam
R
>library(asSeq)
```

```
>extractAsReads(a="NA78.chr1.rn.bam",b="impute-snp.txt",c="OUT2/$out
prefix")
```

[3] Generate read count data

```
bash
$HOME/tool/AS-GENSENG1.0.2/InHouseScripts/3DataGeneration/WGS/allele
specificreadcounts_bedtools.sh NA78.chr1.bam PAIREND
OUT/window.chrom1.bed chr1.mappability.txt chr1.gc.txt OUT.chr$chr
known.txt hg19_gap.chr1.txt OUT2/$outprefix.chr1_hap1.bam
OUT2/$outprefix.chr1_hap2.bam
```

[4] Call SVs

```
GENSENG config.chr1.txt
```

1.3. BASIL-ANISE

Run BASIL and ANISE:

The basil and anise commands were sequentially executed to find INS breakpoints and generate INS sequences, respectively.

Convert output file:

An output vcf file was converted to a vcf format compatible with our evaluation script. RSS was indicated with the mean value of OEALEFT and OEARIGHT.

Commands used:

[1] Find breakpoints

```
basil -ir hs37d5.fa -im NA78.bam -ov $out_prefix.vcf
--filter-max-coverage 105 --filter-min-aln-quality 3
```

[2] Generate insertion sequences

```
anise -ir hs37d5.fa -im NA78.bam -iv $out_prefix.vcf -of
$out_prefix.INS.fa --num-threads 6 --read-mapping-error-rate 2
--overlapper-max-error-rate 3
```

1.4. BatVI

Run BatVI:

A viral sequence dataset was created by merging the HBVall.fa file provided by the developer and the 669 viral sequences (http://www.nvbi.nim.gov/genome/viruses) used to create the Sim-VEI data. The virus and the hs37d5 (hs37.chr17 for the simulated data) reference fasta files

were indexed with batmis, blast, and bwa. The index files for batmis were constructed with both the build_index and bwtformatdb commands. The indexed references and the input fastq files were specified in batviconfig.txt and filelist.txt files in the working directory.

Convert output file:

The number of the split reads supporting the VEI sites, indicated at the 8th column of the output file (final_hits.txt) was assigned to RSS in a converted vcf file. The ranges of split reads corresponding to 2, 3–5, 6–10, 11–20, 21–30, 31–50, 51–80, and >80 were converted to 2, 3, 4, 5, 6, 7, 8 and 9 of RSSs, respectively. The sites supported by only one split read were filtered out.

Commands used:

```
call_integrations.sh . -t 4 --log batvi.log —filterdup
```

The 6th line of the analysecontigx.sh script was changed to 'echo $1/readsx/*.fa | xargs $DIR/cluster.pl - | $DIR/merge_msa.pl > $1/$Path.rc.txt' to avoid an error involved in a 'Argument list too long' problem.

1.5. BICseq2

Run BICseq2:

Normalization of the aligned data and CNV call were conducted with the data split for individual chromosomes. Read position files for each chromosome, which indicate the first positions of aligned reads, were generated with an in-house script and the bam files, where hard-clipped alignments were omitted. The generated read position files and downloaded mappability files (hg19CRG.100mer.chr*.txt) were specified in a configuration file for the normalization.

Convert output file:

The CNV size was determined with the distance between the breakpoints indicated at the second and the third columns of the output file. The type of CNV was defined as DEL and DUP when the log2 ratio of the read depth, indicated at the seventh column of the output file, was ≤ —0.5 and ≥ 0.3, respectively. The sites with —0.5~0.3 of the log2 ratio were filtered out. The genotypes of DEL were assigned to '0/1' and '1/1' when the log2 ratio was ≥ —2 and < —2, respectively. The genotypes of DUP were assigned to '0/1' and '1/1' when the log2 ratio was ≤ 0.8 and > 0.8, respectively. RSSs were assigned as 3, 4, 5, 6, 7, 8, and 9 when —$\log_{10}$ values of the p-values indicated at the eight column were ≤ 1, 1.1—2, 2.1—5, 5.1—10, 10.1—30, 30.1—60, and > 60, respectively.

Commands used:

[1] Normalize potential bases

```
NBICseq-norm.pl -l 100 -s 320 --tmp tmp config.norm.txt norm.out
```

The first two lines of config.norm.txt

```
ChromName faFile  MapFile readPosFile binFileNorm
1 hs37d5.chr1.fa  hg19CRG.100mer.chr1.txt          NA78.chr1.seq
NA78.chr1.norm.bin
```

[2] Detect CNVs

```
NBICseq-seg.pl --bootstrap config.seq.txt NA78.cnv.txt
```

The first two lines of config.norm.txt

```
ChromName binFileNorm
1 NA78.chr1.norm.bin
```

1.6. BreakDancer

Run BreakDancer:

After creating configuration files with bam2cfg.pl, breakdancer-max was executed for each chromosome.

Convert output file:

Output files (*.out) generated for each chromosome were merged and converted to a vcf format. The data corresponding to 'Pos1' and 'num_Reads' in the output file were assigned to the positions and RSSs in the vcf format, respectively. When 'Type' was ITX in the output file, the sv type was assigned to DEL for SVs > 0 in size and to INS for SVs < 0 in size.

Commands used:

[1] Create configuration file

```
$HOME/tool/breakdancer/perl/bam2cfg.pl $bam > config.txt
```

[2] Call SVs

```
breakdancer-max -y 20 -x 200 -r 3 -m 10000000 config.txt > $out_prefix.out
```

1.7. BreakSeek

Preparation of input files:

Input bam files were sorted by read name and converted to sam with samtools.

Run BreakSeek:

After splitting the input sam file for individual chromosomes, breakseek.py was executed with

options: -m 320 (average insert size) -s 80 (standard deviation of insert size) -q 100 (read length).

Convert output file:

Output files (*_INDEL_list.txt) generated for each chromosome were merged and converted to a vcf format. RSS was represented with a mean value of the largest number indicated in the Lbreak column and the largest number indicated in the Rbreak column.

Commands used:

[1] Convert the input bam file to a sam file sorted by read name

```
samtools sort –n NA78.bam | samtools view - > NA78.sn.sam
```

[2] Call SVs

```
python breakseek.py -f NA78.sn.sam -r hs37d5.fa -o ./ -m 320 -s 80 -q
100
```

1.8. BreakSeq2

Run BreakSeq2:

The run_breakseq2.py script was executed by specifying the option --bplib_gff with the breakseq2_bplib_20150129.gff and breakseq2_bplib_20150129.ins files accompanied with the BreakSeq2 package.

Convert output file:

An output vcf file was reformated to the vcf format compatible with our evaluation script. Because the original output file did not contain the RSS information, a fixed number (i.e., 7) of RSS was added to the vcf file. The variants with no 'PASS' filter in the original file were filtered out.

Commands used:

```
run_breakseq2.py --bams NA78.bam --sample $out_prefix --reference
hs37d5.fa --bplib_gff breakseq2_bplib_20150129.gff --bwa $bwa_dir/bwa
--samtools $samtools_dir/samtools --work WORK --nthreads 4
```

1.9. Breakway

Preparation of input files:

To generate a Dtranslocations file, the dtranslocations command from the dnaa package v.0.1.2 (http://dnaa.sourceforge.net) was executed with the input bam file split into each chromosome and options: '-i 10 -b 50 -l 250 -L 800'.

Run Breakway:

The breakway.run.pl script was executed with the input bam and Dtranslocations files and options: '--mindist 250 --maxdist 800 --mincs 25 --maxcs 80 --interval 10 --strand OPPOSITE --ordering 12 --mean 20 --stdev 2'.

Convert output file:

The start breakpoint of a called SV was assigned with a mean value of the start and end positions indicated at the first column of the output file (*.out), and the end breakpoint was assigned with a mean value of the start and end positions indicated at the second column of the output file. The size of the called SV was determined with the distance between the start and end breakpoints. The number at the fourth column in the output file was assigned to RSS.

Commands used:

[1] Preprocess the alignment data

```
$HOME/dnaa-0.1.2/dtranslocations/dtranslocations -i 10 -b 50 -l 200 -L 800 NA78.bam > dtranslocations.out
```

[2] Call SVs

```
breakway.run.pl --dtransfile dtranslocations.out --bamfile NA78.bam --fai hs37d5.fa.fai --bwfolder ~/tool/breakway.0.7.1 --filehandle breakway --mindist 200 --maxdist 800 --mincs 25 --maxcs 80 --interval 10 --strand OPPOSITE --ordering 12 --mean 30 --stdev 6 > $out_prefix.out
```

1.10. CLEVER

Preparation of input files:

The input bam file was sorted by read name with samtools. The CLEVER lib directory was assigned to the LD_LIBRARY_PATH variable.

Run CLEVER:

The clever command was run with the input sorted bam and the reference fasta files, and the output file (predictions.raw.txt) was processed with the postprocess-predictions command with options: '-d 3 -i 3 --vcf --stddev 125 500'.

Convert output file:

The output vcf file (predictions.vcf) was reformatted by assigning the last number indicated at the last column as RSS.

Commands used:

```
export LD_LIBRARY_PATH=$HOME/tool/clever-v2/lib:$LD_LIBRARY_PATH
```

```
clever NA78.sn.bam (read name-sorted bam) hs37d5.fa $out_prefix -T 6
```

(which generates predictions.raw.txt file in $out_prefix directory)

```
postprocess-predictions -d 3 -i 3 --vcf --stddev 80
$out_prefix/predictions.raw.txt 320 > $out_prefix/predictions.vcf
```

1.11. CNVnator

Run CNVnator:

Five consecutive steps: (1) extract read mapping, (2) generate histgram, (3) calculate statistics, (4) Partition read depth signals, and (5) CNV calling, were conducted described below.

Convert and filter output file:

The called CNVs were filtered with the following criteria: (1) when the normalized read depth (the fourth column of the output file (*.out)) was between 0.9 and 1.1, (2) the smallest e-value of the four e-values (the fifth to the eighth columns of the output file) was larger than 1e-7, (3) when the q0 values (fraction of reads mapped with q0 quality, indicated at the last column of the output file) of DUPs was larger than 0.7, (4) when the CNV size was larger than 15 Mb, (5) when the DEL size was smaller than 3 Kb, and (6) when the called CNV was overlapped with gap regions of the reference. For DELs, the q0 values were converted to a provisional number of RSS as follows: the q0 values divided into six ranges (0–0.025, 0.026–0.5, 0.51–0.8, 0.81–0.85, 0.86–0.9, and >0.9) were assigned as 12, 10, 7, 5, 4, and 3 of RSSs, respectively. When the DELs with the lowest q0 range were larger than 500 Kb, the RSS was assigned as 14. For DUPs, the normalized read depth values were converted to a provisional number of RSS as follows: the normalized read depth values divided into five ranges (1.1–1.39, 1.4–1.59, 1.6–1.79, 1.8–1.99, and ≥ 2.0) were assigned as 3, 4, 5, 7, and 10 of RSSs. However, when the q0 value of DUPs was < 0.01, the RSS was assigned as 2 (when and the DUP was > 2 Mb) or 3 (when and the DUP was 1–2 Mb). The normalized read depth values were also reflected to genotypes. The read depth values < 0.25 and 0.25–1.0 were converted to genotypes ‘1/1’ and ‘0/1’ for DELs, the values 1.85–2.2 and 1.35–1.65 were to ‘1/1’ and ‘0/1’ for DUPs, respectively, and the other values out of the ranges were to ‘./.’.

Commands used:

$chr_list = ‘1 2 3 4 5 6 7 8 9 10 11 12 13 14 15 16 17 18 19 20 21 22 X’

[1] Set environmental variables

```
export ROOTSYS=$HOME/tool/root-v5.26-bin
export PATH=$ROOTSYS/bin:$PATH
```

```
export LD_LIBRARY_PATH=$ROOTSYS/lib:$LD_LIBRARY_PATH
```

[2] Extract read mapping

```
cnvnator -root out.root -chrom $chr_list -tree NA78.bam –unique
```

[3] Generate histogram

```
cnvnator -root out.root -chrom $chr_list -his 1000 -d $ref_dir
```

$ref_dir: a directory containing fasta files (chr#.fa) corresponding to $chr_list of hs37d5.fa

[4] Calculate statistics

```
cnvnator -root out.root -chrom $chr_list -stat 1000
```

[5] Partition read depth signal

```
cnvnator -root out.root -chrom $chr_list -partition 1000
```

[6] Call CNVs

```
cnvnator -root out.root -chrom $chr_list -call 1000
```

1.12. Control-FREEC

Preparation of input files:

The SNP file (hg19_snp142.SingleDiNucl.1based.txt) and mappability file (out100m2_hg19.gem) were downloaded from the Control-FREEC website, and the chromosome names in the mappability file were changed to the ones compatible with the GRCh37 format. The mappability file was converted to the bed format according to the instruction in the Control-FREEC website. The chr-fasta files were generated by separating the reference fasta by each chromosome, and the chr-length file, a list of chromosome names and the corresponding sequence length, was also generated.

Run Control-FREEC:

The required files including the input bam, SNP, mappability, chromosome length, and split reference files were specified in a configure file (config.txt). The SNP bed file was specified to the makePileup option in the BAF section of the configure file, and the control section of the configure file were omitted. The specified options in the configure file were as follows:

```
[general]
chrLenFile=$chr_len_file
chrFiles=$chr_fasta_dir/
gemMappabilityFile=$mappability_file
ploidy=2
step=1000
```

```
window=50000
outputDir = ./
sex=XX
breakPointType=4
noisyData=FLASE
breakPointThreshold=1.5
maxThreads=4
[sample]
mateFile=NA78.pileup
inputFormat=pileup
mateOrientation=FR
[BAF]
SNPfile=$HOME/tool/Control-FREEC/hg19_snp142.SingleDiNucl.1based.rena
me.txt
fastaFile=hs37d5.fa
makePileup=$HOME/tool/Control-FREEC/hg19_snp142.SingleDiNucl.1based.r
ename.txt
minimalCoveragePerPosition=5
```

Convert and filter output file:

The SV type was assigned to 'DEL' and 'DUP' when the string indicated at the fourth column of the output file (*_CNVs) was 'loss' and 'gain', respectively. When the string was 'normal', the called site was filtered. The values indicated at the sixth column of the output file (percentage of uncertainty of the predicted genotype) were converted to a provisional number of RSS as follows: the values divided into seven ranges (< 0, 0.01–0.99, 1.0–1.99, 2.0–2.99, 3.0–4.99, 5.0–9.99, ≥ 10) were assigned as 3, 4, 5, 6, 7, 8, and 10 of RSSs, respectively. The genotype data indicated at the sixth column were converted to genotypes '1/1', '0/1', or './.' in the vcf file.

Commands used:

```
freec -conf config.txt
```

1.13. CREST

Run CREST:

Soft-clipped reads in the input bam file were extracted with the extractSClip.pl script. After the

blatserver (gfServer) was started with a 2bit file of the reference fasta, the CREST.pl script was executed with the cover file of the extracted soft-clipped reads and with options: '-l 125 --hetro_factor 0.4'.

Convert output file:

The SV size was determined with the distance between the start breakpoint (the value indicated at the second column) and the end breakpoint (the value indicated at the sixth column) in the output file (*.predSV.txt). When the SV type indicated at the ninth column of the output file was 'ITX' or 'CTX', the site was filtered. The RSS value was determined with the mean value of the numbers of the left and right soft-clipped reads, which were indicated at the fourth and eight columns of the output file, respectively.

Commands used:

```
export PERL5LIB=$PERL5LIB:$HOME/tool/CREST
```

[1] Extract soft-clipped read positions

```
extractSClip.pl -i NA78.bam --ref_genome hs37d5.fa
```

(which generates a hs37d5.cover file)

[2] Call SVs

```
faToTwoBit hs37d5.fa hs37d5.2bit
gfServer start localhost 8000 hs37d5.2bit
```

(execute on a separate terminal window)

```
CREST.pl -f hs37d5.cover -d NA78.bam --ref_genome hs37d5.fa -p $out_prefix -l 100 -t hs37d5.2bit --blatserver localhost --blatport 8000 --2bitdir / --hetero_factor 0.4
```

1.14. DELLY

Run DELLY:

The delly command was executed for each type of SVs, DEL, DUP, or INV.

Convert output file:

The output vcf files for DEL, DUP, and INV were merged, and the variants with no 'PASS' filter were filtered out. The number of paired-end support specified with the 'PE' tag was used for RSS. The genotype data was represented with the GT tag.

Commands used:

```
delly -g hs37d5.fa -o $out_prefix.del.bcf -t DEL NA78.bam -x $HOME/tool/delly2/excludeTemplates/human.hs37d.excl.tsv
```

```
delly -g hs37d5.fa -o $out_prefix.dup.bcf -t DUP NA78.bam -x $HOME/tool/delly2/excludeTemplates/human.hs37d.excl.tsv
delly -g hs37d5.fa -o $out_prefix.inv.bcf -t INV NA78.bam -x $HOME/tool/delly2/excludeTemplates/human.hs37d.excl.tsv
```

1.15. DIGTYPER

Run DIGTYPER:

The step to align split reads in the input bam file to the reference was omitted because the bam file generated with 'bwa mem' contained hard-clipped reads, which caused an error when executed with the align_split_reads.sh script. The DIGTYPER command was run with the bam file for the Sim-A data and with the --only_reversed_reads option. A variations-file as another input file was generated using the DUP and INV variants called with DELLY.

Convert output file:

The genotyping call results with '0/0' and '1/.' were converted to './.' and '0/1', respectively, and represented in a vcf file with the GT tag.

Commands used:

```
DIGTYPER Sim-A.bam variations-file -I 500 -s 100 -r 125 -o Sim-A.vcf --only_reversed_reads
```

1.16. DINUMT

Run DINUMT:

The dinumt.pl script was executed with a refNumt mask file included in the package and with options: '--min_reads_cluster=1 --len_cluster_include=800 --len_cluster_link=1600 --max_read_cov=150'.

Convert output file:

A fixed RSS value (i.e., 10) was used for the vcf format conversion because the information about reads supporting the called SVs was not available in the output vcf file.

Commands used:

```
dinumt.pl --mask_filename=$HOME/tool/dinumt/refNumts.rename.bed --input_filename=NA78.bam --reference= hs37d5.fa --output_filename=$out_prefix.vcf --min_reads_cluster=1 --prefix=NUMT --len_cluster_include=800 --len_cluster_link=1600 --max_read_cov=150
```

1.17. ERDS

Prepare input files:

An SNV vcf file was generated with GATK 3.5.0 HaplotypeCaller. The input bam and SNV vcf files were split for individual chromosomes.

Run ERDS:

The erds_pipeline.pl script was executed for each split bam and vcf file.

Convert output file:

RSS values were assigned as 3 and 5 when the INPRESICE and the PRECISE tags are indicated in the output vcf file, respectively. For DELs, the genotypes were assigned as '0/1' and '1/1' when the copy numbers indicated at the 10th column of the output vcf file were 1 and 0, respectively. For DUPs, the genotypes were assigned as '0/1' and '1/1' when the copy numbers were < 2 and > 2, respectively.

Commands used:

```
# As an example for chromosome 1
erds_pipeline.pl -b NA78.chr1.bam -r hs37d5.chr1.fa -v NA78.SNV.chr1.vcf
-o chr1 --sd b37 --large 1000000 --small 50
```

1.18. FermiKit

Run FermiKit:

The fermi2.pl script was executed with a concatenated fastq file of the input paired-end fastq files and with options: 'unitig -s82m -l125', followed by running make. The run-calling command was executed with a mag.gz file generated from the previous step.

Convert output file:

When the SV type specified in the output vcf file was 'COMPLEX', the site was filtered. The values specified with the 'MINTIPQ' tag were converted to a provisional number of RSS as follows: the MINTIPQ values divided into five ranges (0–1.0, 1.1–5.0, 5.1–10.0, 10.1–15.0, and 15.1–20.0) were assigned as 3, 5, 7, 10, and 15 of RSSs, respectively.

Commands used:

[1] Assemble reads

```
fermi2.pl unitig -s3100m –t8 –l100 -p$out_prefix NA78.fastq >
$out_prefix.mak
make -f $out_prefix.mak –j 8
```

(which generates $out_prefix.mag.gz)

[2] Call SVs

```
run-calling -o$out_prefix –t8 $ref_bwa $out_prefix.mag.gz
```

($ref_bwa is a prefix name of the hs37d5.fa reference index files generated with bwa index)

1.19. forestSV

Run forestSV:

The forestSV command was executed for each chromosome by specifying an information file and a training dataset (rf_1KG_ILMN_BWA_HG19_v1.Rdata).

Convert output file:

The output files (*_calls.txt) generated for each chromosome were merged. The scores indicated at the fifth column of the merged output file were converted to a provisional number of RSS as follows: the scores divided into nine ranges (0–0.2, 0.21–0.3, 0.31–0.4, 0.41–0.5, 0.51–0.6, 0.61–0.7, 0.81–0.9, and > 0.9) were assigned as 2, 3, 4, 5, 6, 7, 8, 9, and 10 of RSSs, respectively.

Commands used:

As an example for chromosome 1

[1] Create info files for each chromosome

The content of chr1.info.txt

```
chr  filename  chrlength bas
1    $out_prefix.chr1/$out_prefix.chr1.bam 249250621
```

[2] Call SVs

```
forestSV --infofile= chr1.info.txt --basename=$out_prefix.chr1
--forest=rf_1KG_ILMN_BWA_HG19_v1.Rdata
```

1.20. GASVpro

Run GASVpro:

The GASVPro-HQ.sh script included in the package was copied to a working directory, and edited the header lines to specify the input file and optional parameters. When the job stacked, the bam files split into each chromosome were processed.

Convert output file:

The start and end breakpoints were assigned with the mean values of the left and right breakpoints, which were indicated at the third and fifth columns of the output file (*.GASVPro.clusters.pruned.clusters), respectively. The SV size was determined with the

distance between the start and end breakpoints. When the SV type in the output file was 'IR', 'I+', or 'I–', the SV type was converted to 'INV', and those with 'TR' or 'TN' were removed. The number indicated at the sixth column of the output file was assigned to RSS.

Commands used:

[1] Preprocess

```
java -Xms8192m -Xmx8192m -jar $HOME/tool/gasv/bin/BAMToGASV.jar
NA78.bam
```

[2] Call SVs

```
java -Xms8192m -Xmx8192m -jar $HOME/tool/gasv/bin/GASV.jar --outputdir .
--lmin 200 --lmax 800 --numChrom 86 NA78.bam
```

1.21. GenomeSTRiP

Run GenomeSTRiP:

The SVPreprocess and SVDiscover commands were conducted with the default parameter file (genstrip_parameters.txt). The SVDiscover command was executed with the options -minimumSize=30 and -maximu,Size 2000000.

Convert output file:

The value specified with the 'GSNPAIRS' tag in the output vcf file (*.dels.vcf) was assigned to RSS. The genotype data was represented with the GT tag.

Commands used:

```
export SV_DIR=$HOME/tool/svtoolkit
classpath="${SV_DIR}/lib/SVToolkit.jar:${SV_DIR}/lib/gatk/GenomeAnal
ysisTK.jar:${SV_DIR}/lib/gatk/Queue.jar"
```

[1] Create reference dict file

```
java -jar $HOME/tool/picard-tools-1.119/CreateSequenceDictionary.jar
R=hs37d5.fa O= hs37d5.dict
```

[2] Preprocess

```
java -Xmx4g -cp ${classpath} org.broadinstitute.gatk.queue.QCommandLine
-S ${SV_DIR}/qscript/SVPreprocess.q -S ${SV_DIR}/qscript/SVQScript.q
-cp ${classpath} -gatk ${SV_DIR}/lib/gatk/GenomeAnalysisTK.jar
-configFile ${SV_DIR}/conf/genstrip_parameters.txt -R hs37d5.fa -I
NA78.bam -md out_meta -jobLogDir logDir -jobRunner Shell -gatkJobRunner
Shell -run
```

[2] Call SVs

```
java -Xmx4g -cp ${classpath} org.broadinstitute.gatk.queue.QCommandLine -S ${SV_DIR}/qscript/SVDiscovery.q -S ${SV_DIR}/qscript/SVQScript.q -cp ${classpath} -gatk ${SV_DIR}/lib/gatk/GenomeAnalysisTK.jar -configFile ${SV_DIR}/conf/genstrip_parameters.txt -R hs37d5.fa -I NA78.bam -md out_meta -runDirectory SV_run -jobLogDir SV_run/logs -O SV_run/svdiscovery.dels.vcf -genderMapFile gendermap.txt -jobRunner Shell -gatkJobRunner Shell -minimumSize 30 -maximumSize 2000000 -debug true -run
```

1.22. GRIDSS

Run GRIDSS:

The GRIDSS CallVariants pipeline was run with the gridss.sh script, in which the input bam file, the reference fasta file, and the other options had been specified. The reference hs37d5.fa was indexed with the 'bwa index' command, and a .dict file was created with picard. The maximum and minimum fragment sizes were specified with the mean insert size + the insert SD * 3 and 125, respectively.

Convert output file:

The output vcf file (NA78.sv.vcf) was annotated with the simple-event-annotation.R script, which was included in the example folder of the GRIDSS package. The annotated vcf file was converted to a vcf file compatible to our study. Duplicated variants and translocations (ITX) were removed. Variants with 'LOW_QUAL;NO_ASSEMBLY' in the 7th column of the annotated vcf file were filtered out. INSs < 30 bp were filtered out. RSSs were assigned based on the quality values, which were indicated the 6th column of the annotated vcf file. The quality values were divided into ten ranges (< 10, 11–50, 51–100, 101–300, 301–500, 501–700, 701–1000, 1001–1500, 1501–2000, > 2000), and the corresponding ranges were assigned as 2, 3, 4, 5, 6, 7, 8, 9, 10, and 12 of RSSs, respectively.

Commands used:

```
./grids.sh
# The content of grids.sh file
INPUT=NA78.bam
BLACKLIST=$HOME/tool/gridss-1.5.0/example/wgEncodeDacMapabilityConsensusExcludable.bed
```

```
REFERENCE=hs37d5.fa
OUTPUT=${INPUT/.bam/.sv.vcf}
ASSEMBLY=${OUTPUT/.sv.vcf/.gridss.assembly.bam}
GRIDSS_JAR=$HOME/tool/gridss-1.5.0/gridss-1.5.0-jar-with-dependencies
.jar
.
.
java -ea -Xmx31g \
-Dsamjdk.create_index=true \
-Dsamjdk.use_async_io_read_samtools=true \
-Dsamjdk.use_async_io_write_samtools=true \
-Dsamjdk.use_async_io_write_tribble=true \
-Dgridss.gridss.output_to_temp_file=true \
-cp $GRIDSS_JAR gridss.CallVariants \
TMP_DIR=./tmp \
WORKING_DIR=. \
REFERENCE_SEQUENCE="$REFERENCE" \
INPUT="$INPUT" \
OUTPUT="$OUTPUT" \
ASSEMBLY="$ASSEMBLY" \
BLACKLIST="$BLACKLIST" \
INPUT_MAX_FRAGMENT_SIZE=560 \
INPUT_MIN_FRAGMENT_SIZE=125 \
THREADS=4 \
2>&1 | tee -a gridss.$HOSTNAME.$$.log
```

1.23. HGT-ID

Run HGT-ID:

The genomic reference datasets and the viral data comprising of 8,481 sequences were automatically downloaded from the NCBI and UCSC sites with the setup.sh script. These reference datasets were specified in the config.txt file.

Convert output file:

The scores indicated at the last column of the output file (output.txt) were assigned to RSS:

score ranges corresponding to < -1, -1–0, 0.1–2, 2.1–5, 5.1–10, 10.1–20, 20.1–50, >50 were converted as 2, 3, 4, 5, 6, 7, 8, and 9 of RSSs.

Commands used:

```
hgt.pl -c config.txt -b NA78.bam -b -d
```

1.24. hydra-sv

Run hydra-sv:

The ‘hydra-multi.sh’ script was run with the option -p 100, specifying the maximum allowable read depth.

Convert output file:

The start breakpoint was assigned with the mean values of the first breakpoints, which were indicated at the second and third columns of the output file (*.sv.final). The end breakpoint was assigned with the mean values of the second breakpoints, which were indicated at the fifth and sixth columns of the output file. The SV size was determined with the distance between the start and end breakpoints. The SV type was assigned to ‘INS’ and ‘DEL’ when the SV size was smaller than 10 and greater than 9, respectively. The number indicated at the seventh column of the output file was assigned to RSS.

Commands used:

```
hydra-multi.sh run -t 4 -p 100 -o $out_prefix.stab.txt stab.txt
# The content of stab.txt file
$out_prefix  NA78.bam
```

1.25. iCopyDAV

Run iCopyDAV:

The calOptBinSize, prepareData, pretreatment, runSegmentation, and callCNV commands were sequentially executed for each chromosome with the input bam files and the reference-associated files, including the mappability, GC-content, and genome-length files, provided by the developer.

Convert output file:

The CNV type was specified as DEL and DUP when the fourth column of the output file (*_tmv_pCNVR.bed) was 0 and 1, respectively. The RSS was fixed to 3 because the information for RSS was not available.

Commands used:

As an example for chromosome 1

```
calOptBinSize -c chr1.config.txt -I NA78.chr1.bam
```

The content of configure file (chr1.config.txt)

```
minSize=100
genomeSize=249250621
percCNLoss=0.05
percCNGain=0.05
fdr=0.01
overDispersion=3
ploidy=2
prepareData -m $mappability.chr1.dat.txt -g $gc.chr1.dat.txt --genome_file $genlen.chr1.txt -o $out_prefix.chr1 --win 1000
pretreatment -i NA78.chr1.bam -o $out_prefix.chr1 -z $out_prefix.chr1_1000.bin --mapfile $out_prefix.chr1_1000.map --gcfile $out_prefix.chr1_1000.gc
runSegmentation -o $out_prefix.chr1 -t
callCNV -o $out_prefix.chr1 -z $out_prefix.chr1_1000.bin --hg19
```

1.26. indelMINER

Run indelMINER:

The ‘indelminer’ command was executed for each chromosome with a configure file, specifying the read group ID, minimum insert size, maximum insert size, and average coverage. The minimum and the maximum insert size was specified with the value of [mean insert size – insert size SD * 3], and the maximum insert size was specified with the value of [mean insert size + insert size SD * 3].

Convert output file:

The output vcf files for each chromosome were merged, and the values specifying with the ‘NS’ tag were assigned to RSS.

Commands used:

As an example for chromosome 1

```
indelminer hs37d5.chr1.fa -i chr1.config.txt sample=NA78.chr1.bam -e 3 > $out_prefix.chr1.vcf
```

The content of configure file (chr1.config.txt)

```
IL   NA78   200 800
RC   1 30
```

1.27. inGAP-sv

Run inGAP-sv:

The ‘inGAP.jar SVP’ command was executed for each chromosome with the option -SE 3 -PE 3 -SIZE 1000000, specifying minimum number of supporting single-end and paired-end reads, and maximum SV size, respectively.

Convert output file:

The output files (*.out) for each chromosome were merged, and the values specifying with the ‘num’ tag were assigned to RSS. INSs were filtered out when the quality score, indicated at the third column of the output file, was < 90.

Commands used:

```
samtools view NA78.bam > NA78.sam ↩
java -mx4000m -jar ~/tool/inGAP_3_1_1/inGAP.jar SVP -SE 3 -PE 3 -SIZE
1000000 -r hs37d5.fa -i NA78.sam -o $out_prefix.out ↩
```

1.28. ITIS

Run ITIS:

The ‘it is.pl’ script was run with the input fastq files of paired-end reads and a fasta file of mobile elements of ALU, L1, SVA, or HERVK and with the options ‘-l 500 -e Y’. The mobile element sequence files were substituted with those included in the MELT package.

Convert and filter output file:

The output files (*.filtered.bed) for ALU, L1, SVA, and HERVK were merged, and the sites with ‘NB=N’ were filtered out. The first number specified with ‘SR’ tag was assigned to RSS.

Commands used:

```
itis.pl -g hs37d5.fa -t ALU.fa -N ALU -l 500 -e Y -c 10,3,3 -1 NA78_1.fq
-2 NA78 _2.fq ↩
itis.pl -g hs37d5.fa -t LINE1.fa -N LINE1 -l 500 -e Y -c 10,3,3 -1 NA78_1.fq
-2 NA78 _2.fq ↩
itis.pl -g hs37d5.fa -t SVA.fa -N SVA -l 500 -e Y -c 10,3,3 -1 NA78_1.fq
-2 NA78 _2.fq ↩
itis.pl -g hs37d5.fa -t HERVK.fa -N HERVK -l 500 -e Y -c 10,3,3 -1 NA78_1.fq
```

```
-2 NA78 _2.fq
```

1.29. laSV

Run laSV:

The 'run_laSV.sh' script was run with the input fastq files of paired-end reads and with the options '-f 500 -l 125 -k 55 -R Ref'. The 'Ref' directory specified with the -R option contained reference-associated files of reference fasta, 2bit index, bwa index, repeat masker bed, and chromInfo (a list of chr-name and its length).

Convert output file:

The SV size was determined with the distance between the first and the second positions, which were indicated at the second and the fifth columns of the output vcf file (*.SVs.vcf). The values specified with the 'BKSUP' tag were assigned to RSS.

Commands used:

```
run_laSV.sh -i $read_prefix -D $HOME/tool/laSV-k63 -R $ref_dir -G hs37d5
-f 320 -l 100 -k 55 -t 8
# $read_prefix is a prefix of the input read fastq files
# $ref_dir is a directory containing hs37d5.fa and its associated files (see above)
```

1.30. Lumpy

Run Lumpy:

Discordant paired read alignments and split read alignments were extracted with samtools and the extractSplitReads_BwaMem command to generate out.discordant.sort.bam and out.sr.sort.bam files. An out.histo file was generated with samtools and the 'pairend_distro.py' script specified with the options '-r 150 -X 4 -N 10000'. The 'lumpy' command was executed with the options: '-mw 4 -tt 0.0 -pe bam_file:out.discordant.sort.bam,histo_file:out.histo,mean:500,stdev:130,read_length:125,min_non_overlap:150,discordant_z:4,back_distance:20,weight:1,id:1,min_mapping_threshold:20 -sr bam_file:out.sr.sort.bam,back_distance:20,weight:1,id:2,min_mapping_threshold:20'. To genotype the called SVs, svtyper (https://github.com/hall-lab/svtyper) was executed with the out.sr.sort.bam and the output vcf files.

Convert and filter output file:

The sites with 'BND' specified with the 'SVTYPE' tag in the output file were filtered out. The values specified with the 'RU' tag were assigned to RSS. The genotype data was represented

with the GT tag.

Commands used:

[1] Preprocess

```
samtools view -uF 0x0002 NA78.bam | samtools view -uF 0x100 - | samtools view -uF 0x0004 - | samtools view -uF 0x0008 - | samtools view -bF 0x0400 - | samtools sort - -o NA78.discordant.sort.bam
samtools view -h NA78.bam | $HOME/tool/lumpy/scripts/extractSplitReads_BwaMem -i stdin | samtools view -Sb - | samtools sort - -o NA78.sr.sort.bam
samtools view NA78.bam | tail -n+100000 | python $HOME/tool/lumpy/scripts/pairend_distro.py -r 100 -X 4 -N 10000 -o NA78.histo
```

[2] Call SVs

```
lumpy -mw 4 -tt 0.0 -pe bam_file:NA78.discordant.sort.bam,histo_file:NA78.histo,mean:320,stdev:80,read_length:100,min_non_overlap:150,discordant_z:4,back_distance:20,weight:1,id:1,min_mapping_threshold:20 -sr bam_file:NA78.sr.sort.bam,back_distance:20,weight:1,id:2,min_mapping_threshold:20
```

1.31. Manta

Run Manta:

The ‘configManta.py’ script was run with the input bam and reference fasta files, and the ‘runWorkflow.py’ script was run to execute the entire workflow.

Convert output file:

The number of split reads supporting an alt allele, specified with the ‘SR’ tag in the output file (*.diploidSV.vcf), was assigned to RSS. When the number of split reads supporting an alt allele was 0, the number of paired-end reads supporting an alt allele, specified with the ‘PR’ tag, was used for RSS. The genotype data was represented with the GT tag.

Commands used:

[1] Configuration

```
configManta.py --bam NA78.bam --referenceFasta hs37d5.fa --runDir ./
```

[2] Call SVs

```
./runWorkflow.py -m local -j 6 -g 100
```

1.32. MATCHCLIP

Run MATCHCLIP:

The matchclip command from the matchclip2 package (https://github.com/yhwu/matchclip2) was executed with the input bam and the reference fasta files.

Convert output file:

The sum of the numbers of the paired-end reads and split reads supporting both the breakpoints, which are indicated at the 9th and the 11th columns of the output file, was assigned to RSS.

Commands used:

```
matchclips -b NA78.bam -f hs37d5.fa -t 3 -o NA78.out
```

1.33. Meerkat

Run Meerkat:

The input bam file generated with 'bwa mem' was modified to mimic the one generated with 'bwa aln' by adding the XA, X0, X1, and XT tags and by removing hard-clipped alignments. The modified bam file was preprocesses with the 'pre_process.pl' script and the options '-k 200 -f 0'. The 'meerkat.pl' script was run with the options '-u 1 -a 0 -p 3 -o 1 -q 2 -z 1000000'. To call SVs, the 'mechanism.pl' script was run with a repeat mask file, which was obtained from the UCSC Genome Browser site (https://genome.ucsc.edu).

Convert output file:

The SV types prefixed with 'del', 'ins', 'tandem', and 'inv' at the first column of the output file (*.variants) were converted to DEL, INS, DUP, and INV, respectively, and those with a 'transl' prefix were filtered out. For INSs, the chromosome, position, and size were assigned with those indicated at the 10th, 11th, and 13th columns of the output file, and the position was given with the mean value of 11th and 12th columns when the INS size was larger than 11. For the other types of SVs, the chromosome, position, and size were assigned with those at the sixth, seventh, and ninth columns of the output file. The values (or mean values) indicated at the fourth column were assigned to RSS.

Commands used:

[1] Add XA/X0/X1/XT tags to the input bam

```
add_X0_tag_bwa-mem-bam.pl NA78.bam | samtools view –Sbh - -o NA78.addXA.bam
# add_X0_tag_bwa-mem-bam.pl is our in-house script.
samtools index NA78.addXA.bam
```

[2] Preprocess

```
pre_process.pl -b NA78.addXA.bam -I $bwa_index -A hs37d5.fa.fai -k 200 -f 0 -l 0 -t 6 –W $path_to_BWA
# $bwa_index: prefix of bwa index files of hs37d5.fa
# $path_to_BWA: path to a directory containing bwa executable
```

[3] Call SVs

```
meerkat.pl -b NA78.addXA.bam -u 1 -a 0 -p 3 -o 1 -q 2 -z 1000000 -l 0 -t 6 -F $ref_dir -B $path_to_blastall –W $path_to_BWA
# $ref_dir: a directory containing hs37d5.fa and directory.index files
# $path_to_blastall: path to a directory containing blastall executable
```

1.34. MELT (MELT-mei and MELT-numt)

Prepare input file:

A reference zip file for MELT-numt was created according to the following procedure. A reference fasta file of the entire human mitochondrial genome sequence was indexed with samtools and Bowtie2-build. A bed file containing reported human NUMTs was created. Analogous to the zip files of mobile elements, included in the MELT package, a MT_MELT.zip file containing the bed, fasta, and its index files was created and was specified with the -t option of MELT.

Run MELT:

The input bam file was preprocesses with the 'Preprocess' command. MELT-mei or MELT-numt was conducted using the 'Single' command with the options '-r 125 -e 500 -d 40000000 -c 30'. This step was performed with the annotation file (hg19.genes.bed) included in the MELT package and a zip file for each type of the transposons (ALU_MELT.zip, L1_MELT.zip, SVA_MELT.zip, HERVK_MELT.zip, or ALU_MELT.zip) or the mitochondrial genome (MT_MELT.zip).

Convert output file:

For MEIs, the output files for each transposon type (*.final_comp.vcf) were merged, and the mean value of the values specified with the 'LP' and 'RP' tags was assigned to RSS. The

genotype data was represented with the GT tag.

Commands used:

[1] Preprocess

```
java -Xmx2G -jar $HOME/tool/MELTv2.0.1/MELT.jar Preprocess NA78.bam hs37d5.fa
```

[2] Call SVs

```
java -jar $HOME/tool/MELTv2.0.1/MELT.jar Single -l NA78.bam -h hs37d5.fa -n $HOME/tool/MELTv2.0.1/add_bed_files/hg19.genes.rename.bed -t $HOME/tool/MELTv2.0.1/me_refs/ALU_MELT.zip -w . -r 100 -e 320 -d 40000000 -c 30
java -jar $HOME/tool/MELTv2.0.1/MELT.jar Single -l NA78.bam -h hs37d5.fa -n $HOME/tool/MELTv2.0.1/add_bed_files/hg19.genes.rename.bed -t $HOME/tool/MELTv2.0.1/me_refs/LINE1_MELT.zip -w . -r 100 -e 320 -d 40000000 -c 30
java -jar $HOME/tool/MELTv2.0.1/MELT.jar Single -l NA78.bam -h hs37d5.fa -n $HOME/tool/MELTv2.0.1/add_bed_files/hg19.genes.rename.bed -t $HOME/tool/MELTv2.0.1/me_refs/SVA_MELT.zip -w . -r 100 -e 320 -d 40000000 -c 30
java -jar $HOME/tool/MELTv2.0.1/MELT.jar Single -l NA78.bam -h hs37d5.fa -n $HOME/tool/MELTv2.0.1/add_bed_files/hg19.genes.rename.bed -t $HOME/tool/MELTv2.0.1/me_refs/HERVK_MELT.zip -w . -r 100 -e 320 -d 40000000 -c 30
```

For NUMT call, MT_MELT.zip file was specified with –t option

1.35. MetaSV

Run MetaSV;

The ‘run_metasv.py’ script was run with the options ‘--filter_gaps --gaps hs37.gap.bed --isize_mean 500 --isize_sd 50 --min_support_ins 3 --disable_assembly’. The gap.bed file was obtained from the UCSC Genome Browser site. For this run, the output files from BreakDancer, Pindel, and CNVnator were used with the options ‘--breakdancer_native, --pindel_native, and --cnvnator_native’, respectively.

Convert output file:

When ‘CNV’ specified in the output file (variants.vcf) has a negative SV size, the SV type was

assigned to 'DEL', and when 'CNV' has a positive size, it was assigned to 'DUP'. The values specified with the 'BD_SUPPORTING_READ_PAIRS' tag were assigned to RSS. When the values specified with the 'BD_SUPPORTING_READ_PAIRS' tag was 0, the values specified with the 'PD_UNIQ_READ_SUPP' or 'PD_READ_SUPP' tag were used for RSS.

Commands used:

```
python run_metasv.py --reference hs37d5.fa --bam NA78.bam --filter_gaps --gaps $gap_file --outdir results --sample $out_prefix --num_threads 2 --isize_mean 320 --isize_sd 80 --min_support_ins 3 --spades $spades_dir --age $age_dir --pindel_native $pindel_out --breakdancer_native $breakdancer_out --breakseq_native $breakseq_out --cnvnator_native $cnvnator_out --disable_assembly
# $gap_file: a bed file annotationg the reference gap regions
# $spades_dir: path to a directory containing spades executable
# $age_dir: path to a directory containing age_align executable
# $pindel_out: Pindel output files with _D, _TD, _INV, and _SI suffixs
# $breakdancer_out: a BreakDancer output file
# $cnvnator_out: a CNVnator output file
# $breakseq_out: a BreakSeq2 gff output file
```

1.36. MindTheGap

Run MindTheGap:

The insertion sites were detected with the 'mindthegap find' command and fastq read files, followed by assembling insertion sequences at the detected breakpoints with the 'mindthegap fill' command.

Convert output file:

The information of called SVs, including chromosome, position, and INS length, was obtained from the headers of the output fasta file. RSS was fixed to 15 because the RSS information was not available.

Commands used:

[1] Call SVs

```
MindTheGap find -in $read_list -ref hs37d5.fa -out $out_prefix -nb-cores 4
```

(which generates a $out_prefix.breakpoints file)

$read_list: a list file describing the input fastq files

[2] Assemble INS sequences

```
MindTheGap fill -in $read_list -bkpt $out_prefix.breakpoints -out
$out_prefix -nb-cores 4
```

1.37. Mobster (Mobstre-mei, Mobster-numt, and Mobster-vei)

Prepare input files:

To enable Mobster to call NUMTs (Mobster-numt), a reference fasta file of the entire human mitochondrial genome sequence with a header containing a prefix name (e.g., '>numt-') was converted to dat format file with MosaicBuild, and a jumping database was created with the dat file and the MosaicJump command. These files were specified to the 'MOBIOME_MAPPING_CMD' line in a Mobster.properties file. For Mobster-vei, the procedure for preparing input files was the same as that described in Mobster-numt, except that the mitochondrial sequence was replaced with the 669 virus sequences (http://www.nvbi.nim.gov/genome/viruses) used for the creation of the Sim-VEI data.

The input bam file generated with 'bwa mem' was modified to mimic the one generated with 'bwa aln' by adding the XA, X0, X1, and XT tags and by removing hard-clipped alignments. Although a bam file generated with 'bwa mem' can be used in recent versions of Mobster, 'bwa aln'-mimicked bam files were used to call variants because 'bwa aln'-mimicked ones gave a slightly higher recall than those with 'bwa mem'.

Run Mobster:

MEIs were detected with the Mobster.jar file, the Mobster.properties configuration file, and the input modified bam files. For NUMTs and VEIs (Mobster-numt and Mobster-vei), the command was conducted with the Mobster.properties file specified for the NUMT- and VEI-specific reference files, as described above.

Convert output file and filtering:

The length of the called insertion sequences was determined with the mean value of the values indicated at the ninth and tenth columns of the output file (*_predictions.txt) when both the values indicated at the ninth and tenth columns were not 'NA'. When either value of the ninth and the tenth columns was 'NA', the value of the other column was assigned to the INS length. When the INS length was less than 150, the site was filtered out. The numbers of supported reads indicated at the 8th columns was assigned to RSS. When at least two of the four values at the 9th to 12th columns were 'NA' and the last four columns were -1 or 'unknown', the site was

filtered out only for MEIs and VEIs.

Commands used:

[1] Edit Mobster.properties file

```
# The edited lines in the file
IN_FILE=NA78.bam
OUT_FILE=$out_prefix
READ_LENGTH=100
SAMPLENAME=NA78
MAPPING_TOOL=bwa ('unspecified' when using bam generated with bwa mem)
MOBIOME_MAPPING_CMD=MosaikBuild -q (FASTQ) -st illumina -out (DAT_FILE)
-quiet && MosaikAligner -in (DAT_FILE) -out (OUT_FILE) -ia
$HOME/tool/Mobster-0.2.4.1/resources/mobiome/54_mobiles_inclHERVK.da
t -hs 9 -mmp 0.1 -act 20 -j $HOME/tool
/Mobster-0.2.4.1/resources/mobiome/54_mobiles_inclHERVK_hs9 -p 2
-annpe $HOME/tool
/Mobster-0.2.4.1/resources/MOSAIK/2.1.26.pe.100.0065.ann -annse
$HOME/tool /Mobster-0.2.4.1/resources/MOSAIK/2.1.26.se.100.005.ann –
quiet
```

(For NUMT and VEI calls, the NUMT- and VEI-specific reference files were specified with the –ia and –j options in the MOBIOME_MAPPING_CMD line)

[2] Call SVs

```
java -Xmx32G -jar $HOME/tool/Mobster-0.2.4.1/
MobileInsertions-0.2.4.jar -properties Mobster.properties -in NA78.bam
-out $out_prefix -sn NA78
```

1.38. OncoSNP-seq

Prepare input files:

The input bam file was split for individual chromosomes, and pileup files for each bam file was generated with the 'samtools mpileup' command. SNP bed files for each chromosome were generated with the process_pileup.pl script and with the pileup files.

Run OncoSNP-seq:

CNVs were called with the run_oncoseq.sh script and the input SNP bed file and with the hgTables_b37.txt and tumourStates.txt files, which were included in the OncoSNP-seq package.

Convert output file:

The output files for each chromosome were merged and converted to the vcf format.

The CNV length was determined with the distance between the start and end positions of the called CNV. When the copy number indicated at the fourth column was lower than 2, the SV type was assigned to 'DEL'. When the copy number was larger than 2, the SV type was assigned to 'DUP'. The log likelihood values indicated at the seventh column were divided into five ranges (< –10000, –10000 to –1001, –1000 to –401, –400 to –101, > –101), and the CNVs contained in these ranges were assigned as 3, 5, 7, 9, and 11 of RSSs, respectively.

Commands used:

As an example for chromosome 1

[1] Prepare input file

```
samtools mpileup –f hs37d5.chr1.fa NA78.chr1.bam > NA78.chr1.pileup ↵
$HOME/tool/oncosnpseq-master/scripts/process_pileup.pl --infile
NA78.chr1.pileup --outfile chr1/NA78.chr1.oncosnp.txt
--snpfile $SNP.bed ↵
```

$SNP.bed: a bed file of the hg19 reference SNPs

[2] Call SVs

```
run_oncoseq.sh $MCR_dir --infile chr1/NA78.chr1.oncosnp.txt
--samplename NA78.chr1 --seqtype illumina --outdir chr1 --hgtable
$HOME/tool/oncosnpseq-master/config/hgTables_b37.txt
--tumourstatetable
$HOME/tool/oncosnpseq-master/config/tumourStates.txt ↵
```

$MCR_dir: MATLAB installed directory

1.39. Pamir

Run Pamir:

Paired-end read fastq files were extracted from the bam files split into each chromosome with the picard SamToFastq command. Insertions were called with the pamir.py script with an input gzipped fastq file, in which the two read fastq files were merged with concatenated order of each mate and all the reads were adjusted to equal length. The final fastq files were compressed using the gzip utility. The command was conducted with the option --mrsfast-threads 10.

Convert output file:

The RSSs were assigned with the values indicated with the 'Support' tag in the output file

(insertions_setcover.vcf).

Commands used:

As an example for chromosome 1

```
pamir.py -p $out_prefix -r hs37d5.chr1.fa --files fastq=NA78.chr1.fq.gz
--num-worker 6 --mrsfast-threads 10 (--resume)
```

NA78.chr1.fq.gz: an input gzip fastq file in which read pairs are concatenated by read name and all the read lengths are identical.

1.40. PBHoney (PBHoney-NGM)

Prepare input files:

The PacBio long read data with 10× coverage in fastq format was aligned to the reference using blasr and the Honey.py pie command with the default parameters. For PBHoney-NGM, the read data was aligned with the NGM-LR aligner (v0.2.6b, https://github.com/philres/ngmlr) with the --no-smallinv option. The output sam file was converted to sorted bam with samtools.

Run PBHoney:

SVs were called with the long reads-aligned bam file successively with the Honey.py tails and spots commands. To adapt for the low depth data, the -b, -E, and -e options for the tail and spots commands were specified with 2. For PBHoney-NGM, SVs were called with the bam file generated with NGM-LR directly with the Honey.py spots command with the custom options provided by Dr. Aaron from the Pacific Biosciences of California, Inc. (see below). The output spots file was converted to the vcf file with RSS values, which were substituted with the values with specified the szCount tag in the info field of the spots file. The genotype data was represented with the GT tag. For PBHoney-NGM, sites with < 0.2 of the rate of RSS, which was calculated by dividing RSS with the value specified with the coverage tag, were filtered out.

Commands used:

[1] Set environment variables

```
source $HOME/tool/PBSuite_15.8.24/setup.sh
```

Edit environment variables in setup.sh

[2] Align reads with blasr

```
Honey.py pie -n 10 -o $out_prefix.sam --temp ./tmp NA78.pacbio.fq
hs37d5.fa
samtools view –Sbh $out_prefix.sam | samtools sort - -o $out_prefix.bam
samtools index $out_prefix.bam
```

[3] Cluster mapped soft-clipped tails

```
Honey.py tails -o $out_prefix.hon.tails $out_prefix.bam
```

[4] Call SVs

```
Honey.py spots -o $out_prefix.hon.spot --reference hs37d5.fa $out_prefix.bam
```

[For PBHoney-NGM]

[1] Set environment variables

```
source $HOME/tool/PBSuite_15.8.24/setup.sh
```

[2] Align with NGM-LR

```
ngmlr -r hs37d5.fa -q NA78.pacbio.fq -t 10 -o $out_prefix.sam --no-smallinv
samtools view -Sbh $out_prefix.sam | samtools sort - -o $out_prefix.bam
samtools index $out_prefix.bam
```

[3] Call DELs

```
Honey.py spots -q 10 -m 10 -i 20 -e 1 -E 1 --spanMax 100000 --consensus None -o $out_prefix.DEL --reference hs37d5.fa $out_prefix.bam
```

[4] Call INSs

```
Honey.py spots -q 10 -m 70 -i 20 -e 2 -E 2 --spanMax 10000 --consensus None -o $out_prefix.INS --reference hs37d5.fa $out_prefix.bam
```

1.41. pbsv

Prepare input files:

To generate alignment data, the PacBio long reads were aligned to the reference with the NGM-LR aligner (v0.2.6b).

Run pbsv:

SVs were called with pbsv contained in the SMRT Link package (v5.0.1), a free software, from the Pacific Biosciences of California, Inc.

Convert output file:

RSS was fixed to 3 because the output vcf file had no information regarding SV-supporting reads.

Commands used:

[1] Align with NGM-LR

See the PBHoney-NGM commands

[2] Call SVs

```
pbsv call hs37d5.fa $out_prefix.bam $out_prefix.bed
```

1.42. PennCNV-Seq

Run PennCNV-Seq:

The bed and frequency files required for PennCNV-Seq were downloaded by executing the download_and_format_database.sh script, which generated a 'reference' folder containing the pre-required files. The frequency file was converted to a pfb file. Bugs found in several lines of penncnv-seq_example.sh and convert_map2signal.pl were fixed, and the penncnv-seq_example.sh script was executed with the input bam and reference files.

Convert output file:

Variants with < 1 Kb were filtered out because of low quality. The type of SV was assigned to DEL and DUP when the copy number indicated with the 'cn' tag in the output file was < 2 and > 2, respectively. The genotype of DEL was '0/1' and '1/1' when the copy number was 1 and 0, respectively. The genotype of DUP was '0/1' and '1/1' when the copy number was 3 and > 3, respectively. RSS was fixed to 3 because the output vcf file had no information regarding SV-supporting reads.

Commands used:

[1] Set PATH to PennCNV executables

```
export PATH=$HOME/tool/PennCNV-1.0.4:$PATH
```

[2] Download pre-required files

```
download_and_format_database.sh hg19 1 0
```

[3] Call CNVs

```
penncnv-seq_example.sh $HOME/tool/PennCNV-Seq
$HOME/tool/PennCNV-Seq/reference hg19 EAS hs37d5.fa NA78.bam
```

1.43. Pindel

Run Pindel:

Configure files for each chromosome were generated, where the input bam file for each chromosome and the mean insert size (i.e., 500) were specified. The 'pindel' command was executed for each chromosome with the configure and ploidy files.

Convert output file:

The output files (*_D for DEL, *_TD for DUP, *_SI for INS, and *_INV for INV) for each

chromosome were merged and converted to the vcf format. The first value specified with the 'BP' tag was assigned to the breakpoint position. The value specified with the 'Supports' tag was assigned to RSS. To obtain genotyping information, the output files were converted to a vcf file with the pindel2vcf executable.

Commands used:

As an example for chromosome 1

```
pindel -f hs37d5.chr1.fa -i $config_file -o $out_prefix.chr1 -Y $ploidy_file -c 1 -x 2 -M 3 -v 100 -d 50 -E 0.92 -w 10 --MIN_DD_MAP_DISTANCE 3000 -g
```

The content of $config_file

```
NA78.chr1.bam 320 $out_prefix.chr1
```

$ploidy_file: list of chromosome names and the corresponding ploidy

1.44. PopIns

Run PopIns:

The popins commands were executed with the input bam files split for individual chromosomes. The assembled contig fasta files were indexed with the 'bwa index' and 'samtool index' commands, and unmapped reads were subsequently mapped to the contigs with the 'popins contigmap' command. Next, the insertion breakpoints of the contigs were determined with the popins place' command. Finally, the called SVs were genotyped with the popins genotype command.

Convert output file:

The output genotyped files (insertions.GT.vcf) for each chromosome were merged and converted to the vcf format. The value given with the 'length_' prefix indicated at the fifth column was assigned to the INS length. The value specified with the 'RP' tag at the eighth column was assigned to RSS. The genotype data was represented with the GT tag.

Commands used:

As an example for chromosome 1

[1] Assemble unmapped reads

```
popins assemble -t 6 -d chr1 chr1/NA78.chr1.bam
```

(which generates a contigs.fa file in the chr1 directory)

```
samtools faidx chr1/contigs.fa
bwa index –a is –p chr1/contigs.fa chr1/contigs.fa
```

[2] Map unmapped reads to assembled contigs

```
popins contigmap -d chr1 –t 6 -m 20000000000 chr1/contigs.fa
```

(which generates a locations.txt file)

[3] Find INS breakpoints

```
popins place -l locations.txt -b $bam_lst -r 100 -e 560 contigs.fa
hs37d5.chr1.fa
```

(which generates non_ref_new.bam and insertions.vcf files)

The content of $bam_lst

```
$out_prefix.chr1.bam
```

[4] Genotype INSs

```
popins genotype -i 1000 hs37d5.chr1.fa $out_prefix.chr1.bam contigs.fa
non_ref_new.bam insertions.vcf
```

1.45. PRISM

Run PRISM;

The input bam was split for individual chromosomes, and the bam file was sorted by read name followed by converting to the sam format. The run_PRISM.sh script was run for each chromosome with the chromosome-split sam file.

Convert output file:

The output files (del_10_50, del_50_100, del_100_1000, and del_1000_plus for DEL, ins_10_50 and ins_50_100 for INS, dup for DUP, and inv for INV) for each chromosome were merged and converted to the vcf format. The values indicated at the second, fourth, and sixth columns were assigned to the breakpoint, size, and RSS of the called SV.

Commands used:

As an example for chromosome 1

[1] Prepare the input sam file

```
export PRISM_PATH=$HOME/tool/PRISM_1_1_6
samtools sort –n NA78.chr1.bam | samtools view - -o NA78.chr1.sn.sam
```

[2] Call SVs

```
run_PRISM.sh -m 320 -e 80 -p 3 -l 100 -r hs37d5.chr1.fa -i NA78.chr1.sn.sam
-I chr1-input -O chr1-output
```

1.46. RAPTR

Run RAPTR:

The input bam file and the reference fasta file were split for individual chromosomes. The split reference fasta files were index with the 'mrsfast -index' command from the mrsfast aligner. The split bam files were preprocessed with the 'RAPTR-SV.jar preprocess' command and the indexed reference files. To call SVs the 'RAPTR-SV.jar cluster' command was executed for each chromosome with the preprocessed output file (*.flat) and the gap bed file.

Convert output file:

The output files (*.raptr.deletions for DEL, *.raptr.insertions for INS, and *. raptr.tand for DUP) for each chromosome were merged and converted to the vcf format. The first breakpoint was determined with the mean value of the values indicated at the second and the third columns, and the second breakpoint was with the mean value of the fourth and the fifth columns. The first breakpoint was assigned to the breakpoint in the vcf file. The distance between the first and the second breakpoints was assigned to the SV size. The value at the seventh column was assigned to RSS.

Commands used:

As an example for chromosome 1

[1] Index reference fasta with mrsfast

```
mrfast --index hs37d5.chr1.fa --ws 15
```

[2] Preprocess the input bam

```
java -jar $HOME/tool/RAPTR-SV-master-2/store/RAPTR-SV.jar preprocess -i
NA78.chr1.bam -r hs37d5.chr1.fa -o $out_prefix.chr1 -t 4
```

(which generates a $out_prefix.chr1.flat file)

[3] Cluster and call SVs

```
java -jar $HOME/tool/RAPTR-SV-master-2/store/RAPTR-SV.jar cluster -s
$out_prefix.chr1.flat -g $gap_bed -o $out_prefix.chr1 -t 4 -i 3
```

$gap_bed: a bed file indicating the start, end, and size of gap regions present in the reference.

1.47. readDepth

Prepare input files:

Annotation files (gcWinds and mappability files) were obtained at https://xfer.genome.wustl.edu/gxfer1/project/cancer-genomics/readDepth/index.html. The input bam file was split for individual chromosomes, and they were converted to bed files.

Run readDepth:

An R script describing a set of commands was executed with a parameter file (params) for each input bed file.

Convert output file:

The output files (alts.dat) for each chromosome were merged and converted to the vcf format. The distance between the start and end position was assigned to the SV size. When the copy number indicated at the last column was smaller than 1, the SV type was assigned to DEL. When the copy number was larger than or equal to 1, the SV type was assigned to DUP. For DELs, the copy number values were divided into six ranges (≤ 0.01, 0.02–0.05, 0.06–0.1, 0.11–0.2, 0.21–0.5, and 0.51–0.9), and the sites contained in these ranges were assigned as 8, 7, 6, 5, 4, and 3 of provisional RSSs, respectively. DELs with > 0.9 of copy number were filtered out. For DUPs, the copy number values were divided into six ranges (< 1.5 1.5–1.9, 2.0–2.4, 2.5–2.9, 3.0–3.9, and ≥ 4.0), and the sites contained in these ranges were assigned as 3, 4, 5, 6, 7, and 8 of provisional RSSs, respectively. DUPs with < 1.1 of copy number were filtered out. The copy number values were also reflected to genotypes in the vcf file. For DELs, when the copy numbers were < 0.5 and 0.5–1.75, the genotypes were assigned as '1/1' and '0/1', respectively. For DUPs, when the copy numbers were > 3.5 and 2.5–3.5, the genotypes were assigned as '1/1' and '0/1', respectively.

Commands used:

As an example for chromosome 1

[1] Construct data structure

```
mkdir chr1 chr1/reads chr1/output chr1/annotations chr1/annotations/gcWinds chr1/annotations/mapability
```

copy entrypoints, gcWinds, and mapability files to the annotations, gcWinds, and mapability folders, respectively

[2] Convert the input bam to bed

```
samtools view -F 4 NA78.chr.bam | awk 'OFS="¥t"{print $3,$4-1,$4}' > chr1/reads/NA78.chr1.bed
```

[3] Call SVs

```
Rscript readDepth.R
```

The content of readDepth.R

```
library("readDepth")
rdo = new("rdObject")
```

```
rdo = readDepth(rdo)
rdo = rd.mapCorrect(rdo, minMapability=0.75)
rdo = rd.gcCorrect(rdo)
segs = rd.cnSegments(rdo, minWidth=3)
writeSegs(segs)
writeAlts(segs,rdo)
writeThresholds(rdo)
# The content of param file
readLength 100
fdr  0.01
overDispersion 3
gcWindowSize 100
percCNGain 0.1
percCNLoss 0.1
chunkSize  5e6
maxCores 4
readCores  4
verbose  TRUE
```

1.48. RetroSeq

Run RetroSeq:

Reference TE bed files, describing the locations of each type of transposable elements in the reference, were obtained from the MELT MEI detection tool package. The 'retroseq.pl -discover' command was executed with a list file (TE_list.txt), which indicated the TE bed file and the corresponding TE type. The 'retroseq.pl -call' command was conducted with the output file from the discover phase.

Convert output file:

The value specified with the 'FL' tag at the last column of the output file (*.PE.vcf) was assigned to provisional RSSs. The INS size was fixed to 0 because the information for the size of the insertion sequence lacked in the output file. The genotype data was represented with the GT tag.

Commands used:

[1] Discover MEIs

```
retroseq.pl -discover -bam NA78.bam -refTEs TE_list.txt -output
$out_prefix.out -q 20
```

[2] Call MEIs

```
retroseq.pl -call -bam NA78.bam -input $out_prefix.out -ref hs37d5.fa
-output $out_prefix.vcf -hets -filter TE_list.txt -reads 3 -q 20
```

1.49. Sniffles

Prepare input files:

The PacBio long read data with 10× coverage were aligned with the NGM-LR aligner (v0.2.6b) with the --no-smallinv option. The output sam file was converted to sorted bam with samtools.

Run Sniffles:

SVs were called with the bam file and with the options '-s 2 -q 5 -t 3 --genotype'.

Convert output file:

The last value separated with ':' in the tenth column of the output vcf file was assigned to RSS. The genotype data (the first string separated with ':' in the tenth column) was represented with the GT tag.

Commands used:

[1] Align with NGM-LR

See the PBHoney-NGM commands

[2] Call SVs

```
sniffles -m $out_prefix.bam -s 2 -q 5 -t 4 --genotype -v $out_prefix.vcf
```

1.50. Socrates

Run Socrates:

The 'Socrates all' command was executed with the option --percent-id=97 and with the Bowtie2 indexed database and the input bam file.

Convert output file:

The start and end breakpoints were obtained from the values at the first and the 13th columns of the output file (results_Socrates_paired_*). The start breakpoint was assigned to the breakpoint in the vcf file. The distance between the start and end breakpoints was assigned to the SV size. When the SV size was larger than or equal to 30 bp, the called SV was assigned to DEL. When the SV size was smaller than 30 bp, the called SV was assigned as INS. The value at the seventh column was assigned to RSS.

Commands used:

[1] Index the reference with Bowtie2

```
bowtie2-build hs37d5.fa hs37d5.fa
```

[2] Call SVs

```
Socrates all -t 4 --bowtie2_threads 4 --bowtie2_db hs37d5.fa -p 97
--jvm_memory 11g NA78.bam
```

1.51. SoftSearch

Run SoftSearch:

The input bam file and the reference fasta file were split for individual chromosomes. The SoftSearch.pl script was run for each input bam and reference files with the options '-r 3 -m 3'.

Convert output file:

The output files for each chromosome were merged. The string specified with the 'EVENT' tag at the eighth column was assigned to the SV type. When the SV type strings were 'TDUP' and 'NOV_INS', they were converted to DUP and INS, respectively. The second sites of duplicated SV sites were removed because SV events were always represented in duplicate with two reciprocal breakpoints in the POS and ALT fields in the output file. When the SV type was 'CTX' and the chromosome names indicated at the first and the fifth columns were same, the SV type was assigned to INS. The value specified with the 'ISIZE' tag was assigned to the SV size. A stretch of values at the last column were assigned to RSS for CTX (INS), DEL, INS, INV, NOV_INS, and TDUP.

Commands used:

As an example for chromosome 1

```
export PERL5LIB=$PERL5LIB:$HOME/tool/Softsearch-2.4/lib
SoftSearch.pl -b NA78.bam -f hs37d5.fa -o $out_prefix.vcf -r 3 -m 3 -c
1
```

1.52. SoftSV

Run SoftSV:

The input bam file was split for individual chromosomes. The SoftSV command was executed for each split bam file.

Convert output file:

The output files (deletions.txt and deletions_small.txt for DEL, tandems.txt and

tandems_small.txt for DUP, inversions.txt and inversions_small.txt for INV, and insertion_small.txt for INS) for each type of SV were merged and converted to the vcf format. The value at the sixth column was assigned to RSS. Finally the vcf files for each chromosome were merged.

Commands used:

As an example for chromosome 1

```
SoftSV -i NA78.chr1.bam -r 1 -o chr1
```

1.53. SoloDel

Run SoloDel:

The input bam file and the reference fasta file were split for individual chromosomes. SoloDel.jar was executed for each split input file with the output files of BreakDancer as a deletion list.

Convert output file:

The output files (*.somatic.call) for each chromosome were merged and converted to the vcf format. The SV type was fixed to DEL, and the value at the fifth column was assigned to RSS.

Commands used:

As an example for chromosome 1

```
java -jar $HOME/tool/SoloDel-1.0.0/SoloDel.jar -R hs37d5.chr1.fa -g hg19 -c 3 -f $fastahack_path -t $blat_path -l 100 -i 420 -D NA78.chr1.bam -d $ref_var -o $out_prefix.chr1.dBD -w $work_dir
```

$fastahack_path: path to fastahack executable

$blat_path: path to blat executable

$ref_var: an output file of BreakDancer

$work_dir: the current working directory

1.54. Sprites

Run Sprites:

The sprites command was executed for each input file with the default option.

Convert output file:

The start and end breakpoints of the called SVs were determined by taking the means of the values indicating at the 2nd/3rd and 5th/6th columns in the output file, respectively. The length of SVs was assigned with the distance between the start and end breakpoints. RSSs were fixed

to 3 because the output file had no information regarding SV-supporting reads.

Commands used:

```
sprites -r hs37d5.fa -o $out_prefix.calls NA78.bam
```

1.55. SV^2

Prepare input files:

A vcf file of the Sim-A SVs detected with Manta was used for an input SV vcf file, in which INS and INV variants had been deleted and the END tag had been added in the INFO field. A vcf file of the Sim-A SNVs detected with GATK 3.5.0 HaplotypeCaller was used for an input vcf file. The SNV vcf file was compressed with bgzip and indexed with tabix. The SNV ped file was generated with plink2.

Run SV^2:

DELs and DUPs detected with Manta were genotyped with the sv2 command and the prepared input files including the sorted Sim-A bam file.

Convert output file:

The genotyping call results with '0/0' were converted to './.' and represented in a vcf file with the GT tag.

Commands used:

```
sv2 -hg19 hs37d5.fa
sv2 -i Sim-A.bam -v Manta.Sim-A.vcf -snv GATK.Sim-A.SNV.vcf.gz -p
GATK.Sim-A.SNV.ped
```

1.56. SvABA

Run SvABA:

The svaba command was executed with the input bam and with the --germline option.

Convert output file:

The SV size was represented with the value specified with the SPAN tag of the eighth column of the output file (*.svaba.unfiltered.sv.vcf). The SV type was classified according to the ALT field of the output file. When there are two lines with the same chromosome and coordinate, we refer the ALT fields in the first and the second line ALT1 and ALT2, respectively. When ALT1 starts with 'N[' and ALT2 ends with ']N', the SV type is assigned to DEL, where N is any nucleotide. L. When ALT1 ends with ']N' and ALT2 starts with 'N[', the SV type is assigned to DUL. When both ALT1 and ALT2 end with '[N', the SV type is assigned to INV. INS was

assigned when the SV size was -1. The RSS value was fixed to 2 because the information for RSS was not available.

Commands used:

```
svaba run -t NA78.bam -G hs37d5.fa -a NA78 -p 4 --germline
```

1.57. SVDetect

Run SVDetect:

The input bam file was preprocessed with the script, BAM_preprocessingPairs.pl. The command 'SVDetect linking filtering' was executed with a configure file (sample.sv.conf) included in the SVDetect package.

Convert output file:

The output file (*.links.filtered) was converted to a tabulated-text format with the 'SVDetect links2SV' command. The converted sv file (*.links.filtered.sv.txt) was further converted to a vcf format. When the SV type indicated at the second column of the sv file was 'INSERTION' or 'INS_FRAGMT', the SV type was assigned to INS. When the SV type of the sv file was 'DUPLICATION', 'SMALL_DUPLI' or 'LARGE_DUPLI', the SV type was assigned to DUP. When the SV type of the sv file has a 'INV' prefix, the SV type was assigned to INV. When the SV type of the sv file was 'UNDEFINED' or 'TRANSLOC', the corresponding site was removed. The start breakpoint was assigned with the middle position of the range indicated at the fifth column of the sv file. The score indicated at the 13 column of the sv file were divided into five ranges (0.85–0.89, 0.90–0.94, 0.95–0.97, 0.98–0.99, 1.0), and the scores contained in these ranges were assigned as 3, 5, 7, 10, and 15 of RSSs, respectively.

Commands used:

[1] Preprocess

```
BAM_preprocessingPairs.pl NA78.bam
```

(which generates NA78.ab.bam)

[2] Call SVs

```
SVDetect linking filtering -conf $conf_file
```

The content of $conf_file

```
read1_length=100
read2_length=100
mates_file=NA78.ab.bam
num_threads=4
```

```
cmap_file=$chr_len_file
mu_length=320
sigma_length=80
split_link_file=1
split_mate_file=1
sv_type = intra
```

$chr_len_file: indicating sequential number, name, and length for each chromosome (e.g., 1st line: 1 1 249250621)

[3] Convert to bed file

```
SVDetect links2SV -conf $conf_file
```

1.58. SVelter

Run SVelter:

SV calling consisting of six processes was conducted with the svelter.py script with the default setting.

Convert output file:

The SV length was determined with the distance between the start and end positions. RSS was assigned as 3, 4, 5, 6, 7, 8, and 9 when the scores indicated at the sixth column of the output file were 0, 1–19, 20–39, 40–59, 60–79, 80–99, 100, respectively.

Commands used:

[1] Set up

```
svelter.py Setup --reference hs37d5.fa --workdir ./ --support
$HOME/tool/svelter/Support/GRCh37 --ref-index
$HOME/tool/svelter/Support/ref-index/GRCh37
```

[2] Build null models

```
svelter.py NullModel --sample NA78.bam --workdir ./
```

[3] Search for breakpoints

```
svelter.py BPSearch --sample NA78.bam --workdir ./
```

[4] Cluster breakpoints

```
svelter.py BPIntegrate --sample NA78.bam --workdir ./
```

[5] Resolve complex SVs

```
svelter.py SVPredict --sample NA78.bam --workdir ./ --bp-file
bp_files.NA78.bam/NA78.txt
```

[6] Write output in vcf format

```
svelter.py SVIntegrate --workdir ./ --prefix NA78 --input-path bp_files.NA78.bam
```

1.59. SVfinder

Run SVfinder:

The input bam file was converted to a sam file with samtools, and the chromosome name was converted to the hg19-compatible name (e.g., X -> chrX). The SVfinder.py script was run with the annotation file (hg19.ucsc.gene.txt) included in the package.

Convert output file:

When the SV type indicated at the first column of the output file was 'Interchromosomal_translocation', the called site was filtered out. When the SV type was 'Intrachromosomal_translocation' and the chromosome names indicated at the third and the eighth columns were same, the SV type was assigned to INS. The start breakpoint was determined with the mean value of the values indicated at the fourth and fifth columns, and the end breakpoint was with the mean value of the eighth and ninth columns. The start breakpoint was assigned to the breakpoint of the called SV in the vcf file. The distance between the start and end breakpoints was assigned to the SV size. The value of the first column was assigned to RSS.

Commands used:

[1] Preprocess

```
rename_sam.pl NA78.bam > NA78.rn.sam
```

rename_sam.pl is our in-house script that adds 'chr' prefix to the reference names in a bam file

[2] Call SVs

```
SVfinder.py -i NA78.rn.sam -o $out_prefix -n 3 -r 100 -g $HOME/tool/SVfinder-master/annotation/hg19.ucsc.gene.txt
```

1.60. SVseq2

Run SVseq2:

The input bam file and the reference fasta file were split for individual chromosomes. For DEL calling, the SVseq2_2 command was executed for each split input file. For INS calling, the 'SVseq2_2 -insertion' command was executed for each chromosome.

Convert output file:

The output files (*.DEL.out and *.INS.out) for each chromosome were merged and converted to the vcf format. The information for chromosome, breakpoint, and SV size (breakpoint distance) was obtained from the space-separated header line for each called SV. The number of the flanking regions around the called breakpoints, which were represented in the output files, was assigned to RSS.

Commands used:

As an example for chromosome 1

[1] Call DELs

```
SVseq2_2 -r hs37d5.chr1.fa -c 1 -b NA78.chr1.bam --o $out_prefix.chr1.DEL.out --is 320 80
```

[2] Call INSs

```
SVseq2_2 -insertion -c 1 -b NA78.chr1.bam --o $out_prefix.chr1.INS.out
```

1.61. Tangram (Tangram-mei, Tangram-numt, Tangram-vei)

Prepare input files:

<Tangram-mei> A reference fasta file was created by combining the human chromosome sequences (e.g., hs37.chr17.fasta) with the mobile element sequences (moblist_19Feb2010_sequence_length60.fa included in the package) with a header containing a prefix name '>moblist_. This combined reference was converted dat file with MosaicBuild. Read files converted to a mbk file was aligned with MosaicAligner and the dat file and by specifying the prefix name 'moblist_' to the -sref option. The resulting bam file was sorted by coordinate and used as the input file.

<Tangram-numt> The procedure for preparing input files was the same as that described in Tangram-mei, except that the mobile element sequences were replaced with the mitochondrial sequence with a header containing a prefix name '>mit_'.

<Tangram-vei> The procedure for preparing input files was the same as that described in Tangram-numt, except that the mitochondrial sequence was replaced with the 669 virus sequences used for the creation of the Sim-VEI data.

Run Tangram:

The entire processes after the Mosaik alignment were conducted with the input bam and the reference fasta files, which had been split for individual chromosomes. The reference was indexed with the tangram_index command with the target fasta file of the mobile elements,

mitochondrial sequence, or viral sequences, specified with the -sp option. The input bam file was scanned with the tangram_scan command, and the insertion events were detected with the tangram_detect command. The insertion detection step was conducted with the output files (lib_table.dat, hist.dat, and indexed reference files) from the previous steps and with the options '-gt -mq 10 -smq 10 -srf 3'. Finally the output vcf file was filtered with the tangram_filter.pl script, which eliminates the insertion sites corresponding to the mobile element sequences (mitochondrial or viral sequences) defined in the reference.

Convert output file:

The mean value of the values specified with the 'SR5' and 'SR3' tags in the output vcf file was assigned to RSS. The SV size was fixed to 0 because the information for the size of the insertion sequences lacked in the output file. The genotype data was represented with the GT tag.

Commands used:

[1] Align reads with Mosaik

```
MosaikBuild -fr hs37d5.fa -oa hs37d5.dat -sn "Homo sapiens" -ga GRCh37
MosaikJump -ia hs37d5.dat -out hs37d5 -hs 15
MosaikBuild -q NA78_1.fq -q2 NA78_2.fq -st illumina -ln $out_prefix -mfl 320 -out $out_prefix.mbk
MosaikAligner -in $out_prefi.mbk -ia hs37d5.dat -a all -sref $special_ref_prefix -j hs37d5 -out $out_prefix -p 10 -annpe $HOME/tool/MOSAIK-2.2.3-source/networkFile/2.1.78.pe.ann -annse $HOME/tool/MOSAIK-2.2.3-source/networkFile/2.1.78.se.ann
```

$special_ref_prefix: a prefix name of the headers of combined reference sequences (e.g., 'moblist' for the MEI reference [moblist_19Feb2010_sequence_length60.fa] included in the Tangram package

```
samtools sort $out_prefix.bam -@ 2 -o $out_prefix.sort.bam
samtools index $out_prefix.sort.bam
```

[2] Index reference (as an example for chromosome 1)

```
tangram_index -ref hs37d5.chr1.fa -out hs37d5.chr1.index -sp $sref
```

$sref: a fasta file of MEI, NUMT, or VEI reference sequences

[3] Scan bam file (as an example for chromosome 1)

```
tangram_scan -in $bam_lst -dir chr1
```

(which generates lib_table.dat and hist.dat files in the out directory)

$bam_list: a file listing the full path of a chromosome-split file (e.g., NA78.chr1.bam) of the bam file generated at [1]

[4] Call SVs (as an example for chromosome 1)

```
tangram_detect -lb chr1/lib_table.dat -ht chr1/hist.dat -in $bam_lst
-ref hs37d5.chr1.index -gt -mq 10 -smq 10 -srf 3 -rg 1 -p 4 -out $out_prefix
```

[5] Filter called SVs

```
tangram_filter.pl --vcf $out_prefix.vcf --msk $mask_list
```

$mask_list: a file listing bed files that were annotated for ALU, L1, SVA, and HERVK (or NUMT, VEI) in the hs37d5 reference

The content of $mask_list

```
ALU  400  hs37_rmsk_Alu.bed
L1 400  hs37_rmsk_L1.bed
SV 400  hs37_rmsk_SVA.bed
HE 400  hs37_rmsk_HERV.bed
```

1.62. TEA

Run TEA:

The command './tea_run [prefix of the input bam] tea ra' was conducted with the options '-c hg19 -f -K'.

Convert output file:

The breakpoint, size, and RSS of the called SVs were assigned with the values indicated at the seventh, the fifth, and 12th columns of the output file (*.germline), respectively.

Commands used:

```
export
LD_LIBRARY_PATH=$HOME/tool/TEA-master/preprocess/lib:$LD_LIBRARY_PA
cp $HOME/tool/TEA-master/tea.run ./
cp $HOME/tool/TEA-master/conf_file ./
./tea.run NA78 tea ra -c hg19 -f -K -p 4
```

1.63. TEMP

Run TEMP:

A hs37_rmsk.bed file of the TE annotation and a TE.fasta file of the TE consensus sequences

were obtained from the UCSC site and the MELT package, respectively. The lines 132 and 133 of the script were modified because our bam file was not recognized as a bam generated with ‘bwa mem’. The shebang of all the perl scripts in the package was converted to ‘#!/usr/bin/perl’.

Convert output file:

The INS lengths were estimated with the distance between the start and end positions, and the RSSs were assigned with the number of ‘VariantSupport’ indicated at the 7th column of the output file.

Commands used:

```
TEMP_Insertion.sh -i NA78.bam -s $HOME/tool//TEMP/scripts -m 3 -r $TE.fasta -t $rmsk.bed -c 4
```

$TE.fasta: a fasta file of transposable elements

$rmsk.bed: a bed file of annotated TE for the GRCh37 reference

lines 132 and 133 in TEMP_Insertion.sh were changed to 'perl $BINDIR/pickUniqPairFastq_MEM.pl $i.unpair.sam $i.unpair.uniq $SCORE' and 'perl $BINDIR/pickUniqPos_MEM.pl $i.unpair.sam $SCORE > $i.unpair.uniq.bed', respectively.

1.64. TIDDIT

Run TIDDIT:

The command ‘./TIDDIT --sv -b [the input bam file] was conducted with the default options.

Convert output file:

The RSSs were assigned with the values indicated with the ‘LTE’ tag in the info field of the TIDDIT output vcf file. When the LTE tag was not specified in the output file, RSS was set to 10. The SV length (SVLEN) was determined by subtracting the first breakpoint (POS) from the second breakpoint (END). The sites without the PASS filter were filtered out. An SV type of TDUP was converted to DUP whereas the calls with an SV type of IDUP and BND were removed. INS calls were also removed because many of them seem to be wrongly annotated but should be rather annotated as DELs.

Commands used:

```
TIDDIT --sv -b NA78.bam –o $out_prefix
```

1.65. Ulysses

Run Ulysses:

The input bam file was split for individual chromosomes. The ReadBam.py script was run for

each split bam with the -p, -ststs, and -out options to specify output files. The output parameter file contained a description, 'vcf=False', it was changed to 'vcf=True'. The Ulysses.py script was run with the parameter file and by specifying DEL, DUP, and INV with the -typesv option to sequentially call DEL, DUP, and INV.

Convert output file:

The output files (*.DEL.vcf, *.DUP.vcf, and *.INV.vcf) for each chromosome were merged and converted to the vcf format compatible with our evaluation script. When the SV type specified with the 'SVTYPE' tag was 'RT' or 'NRT', the site was filtered out. The mean value of the absolute values specified with the 'SVM' and 'SVMI' tags was assigned to the SV size. A negative log-value of the p-value specified with the 'PVAL' tag was added with 3, and the value was assigned to a provisional RSS.

Commands used:

As an example for chromosome 1

[1] Preprocess

```
ReadBAM.py -out $out_prefix.chr1 -stats NA78.chr1.stats.txt -p ulysses_params.chr1 NA78.chr1.bam
```

(which generates NA78.chr1.stats.txt and ulysses_params.chr1 files)

The input NA78.chr1.bam file should be placed in the working directory.

The generated ulysses_params.chr1 file was modified to change 'vcf=False' to 'vcf=True'.

[2] Call DELs

```
Ulysses.py -p ulysses_params.chr1 -typesv DEL
```

[3] Call DUPs

```
Ulysses.py -p ulysses_params.chr1 -typesv DUP
```

[4] Call INVs

```
Ulysses.py -p ulysses_params.chr1 -typesv INV
```

1.66. VariationHunter

Prepare input files:

The input read files were divided into each 500,000 read pair set, and they were aligned to the reference with the mrFast aligner (http://mrfast.sourceforge.net) with the --discordant-vh option. The multiple output *DIVET.vh files were merged and filtered. The resulting filtered DIVET file was split into each chromosome and sorted by coordinate.

Run VariationHunter:

The clustering step was conducted for each chromosome with the VH command and with a set of files of chromosome length, gap bed, and a satellite-repeatmasker bed. The selection step was conducted with the multiInd_SetCover command.

Convert output file:

The output files (*_select.out) for each chromosome were merged and converted to the vcf format. When the string specified with the 'SVtype:' prefix was 'D', 'I', and 'V', the SV type was assigned to DEL, INS, and INV, respectively. For INS, the mean value of the values indicated at the second to the fifth columns was assigned to the breakpoint. For DEL and INV, the start breakpoint was determined with the mean value of the values at the second and the third columns, and the end breakpoint was determined with the mean value of the values at the forth and the fifth columns. The first breakpoint and the distance between the first and end breakpoints were assigned to the breakpoint and the size of the called SV. The value specified with the 'sup:' prefix was assigned to RSS.

Commands used:

[1] Align reads with mrFast

```
mrfast --search hs37d5.fa --pe --discordant-vh --seq1 $sub_read1 --seq2 $sub_read2 --min 200 --max 600 -o $out_prefix.$div_num
```

$sub_read1, $sub_read2: fastq files containing 500,000 read pairs

$div_num: the sequential number of the divided read set

The generated multiple $out_prefix.$div_num_DIVET.vh files were merged to generate NA78_DIVET.vh.

[2] Filter alignment file

```
cat NA78_DIVET.vh | awk '{if ($11<=6 && ($10=="I" || ($10=="D" && $3-$7>-500000 && $3-$7<500000) || ($10=="V" && $3-$7>-10000000 && $3-$7<10000000))) print}' > NA78_DIVET.filt.vh
```

NA78_DIVET.filt.vh file was sorted by coordinate and split into each chromosome.

As an example for chromosome 1

[3] Create maximal cluster

```
$HOME/tool/VariationHunter/clustering/VH -c $chr1.len.txt -i $HOME/tool/VariationHunter/clustering/initInfo -l $chr1.lib -r $chr1.mask.bed -g $chr1.gap.bed -o $out_prefix.chr1.out -t $out_prefix.chr1
```

The content of $chr1.lib

```
1
lib-1 $out_prefix.chr1 DIVET.filt.chr1.vh 200 900 100
```
$chr1.len.txt: a file indicating the name and the length of chromosome 1

$chr1.mask.bed: a repeat-annotated bed file generated with the RepeatMasker output

$chr1.gap.bed: a bed file annotated for the gap regions of the GRCh37 chr1 reference

[4] Call SVs

```
$HOME/tool/VariationHunter/selection/multiInd_SetCover -l $chr1.lib -r $out_prefix.chr1 -c $out_prefic.chr1.out -t 1000 -o $out_prefic.chr1_select.out
```

1.67. VirusFinder

Run VirusFinder:

The detect_virus.pl script was modified because it was optimized for an older version of Trinity. The lines 254 to 274 of the script was replaced with the line "$trinity_script --seqType fa --max_memory 10G --single blat_out_candidate_singlelane.fa --min_contig_length $min_contig_length --output trinity_output --CPU $thread_no;". In addition, the following three lines to remove extra header items of the Trinity output file (Trinity.fasta) were added at the line 286 of the script. The rename_trinity_fasta.pl script renames the header of a fasta file so that leaves only the first two items of a space-separated header.

<Added lines>

"system ("rename_trinity_fasta.pl trinity_output/Trinity.fasta > trinity_output/Trinity.rn.fasta");"

"system ("rm trinity_output/Trinity.fasta");"

"system ("mv trinity_output/Trinity.rn.fasta trinity_output/Trinity.fasta");"

The 669 virus sequences used for the creation of the Sim-VEI data were used as the virus reference, and indexed with blast. The reference genome was also indexed with blast and bowtie2. These indexed files and the input bam file were specified in the config.txt file that was included in the package. To detect VEIs the VirusFinder.pl script was run with the config.txt file and the virus reference fasta file.

Convert output file:

The value indicated at the seventh column of the output file (results-virus-loci.txt) was assigned to RSS. When the RSS string was separated with '+', the former value was used for RSS, but the latter value was used for RSS when the former value was smaller than 3. The SV size was

fixed to 0 because the information for the size of the insertion sequences lacked in the output file.

Commands used:

```
VirusFinder.pl -c $config -v $virus_ref
# $virus_ref: a fata file of virus reference sequences
# Edited lines in $config file
alignment_file =NA78.bam
thread_n=6
```

1.68. VirusSeq

Run VirusSeq:

The reference genome files (hg19.fa and hg19Virus.fa), viral sequence file (gibVirus.fa), and the other associated files provided by the developer were used. The viral sequence file contained 25,525 viral sequences. The input paired-end fastq files were aligned to the reference containing the hg19 genome and the viral sequences with the Mosaik aligner provided by the developer.

Convert output file:

The number of the discordant paired-end reads supporting the viral integration sites, indicated at the 3rd column of the output file was assigned to RSS. The ranges of discordant read pairs corresponding to 0–2, 3–4, 5–7, 8–10, 11–15, 16–20, 21–30, 31–40, and >40 were converted to 2, 3, 4, 5, 6, 7, 8, 9, and 10 of RSSs, respectively.

Commands used:

The reference file (hg19Virus.fa) was indexed with MosaikBuild and MosaikJump to generate hg19Virus.dat and hg19Virus.JumpDb_*. The read fastq files (NA78_1.fq and NA78_2.fq) were index with MosaikBuild to generate NA78.mbk, NA78_1.mbk, and NA78_2.mbk.

```
MosaikAligner -in NA78.mb -ia hg19Virus.dat -j hg19Virus.JumpDb -out NA78.hybrid.out -p 8 -hs 15 -mmp 0.06 -mmal -minp 0.5 -act 25 -mhp 100 -m unique -a all -km -pm
Spanner --scan –infile NA78.hybrid.out –outdir Spanner_out
Spanner --build –infile NA78.hybrid.out –outdir Spanner_out -f Spanner_out/MSK.stats -a Spanner_anchor_hg19Virus.txt -t
MosaikAligner -in NA78_1.mbk -ia hg19Virus.dat -j hg19Virus.JumpDb -out NA78 SE1.out -p 8 -hs 15 -mmp 0.06 -mmal -minp 0.5 -act 25 -m unique -km -pm
```

```
MosaikAligner -in NA78_2.mbk -ia hg19Virus.dat -j hg19Virus.JumpDb -out
NA78 SE2.out -p 8 -hs 15 -mmp 0.06 -mmal -minp 0.5 -act 25 -m unique -km
-pm
mkdir mosaik_tmp
export MOSAIK_TMP=./ mosaik_tmp
MosaikSort -in NA78.SE1.out -out NA78.SE1.sort.out -u
MosaikSort -in NA78.SE2.out -out NA78.SE2.sort.out -u
MosaikMerge -in NA78.SE1.sort.out -in NA78.SE2.sort.out -out
NA78.SE12.sort.out
MosaikText -in NA78.SE12.sort.out -axt NA78.SE12.axt
# change the working directory to Spanner_out
Spanner_cross_converter.pl hg19Virus_refGene_RIS.txt ../NA78.SE12.axt
NA78.CrossRoad
VirusSeq_Integration.pl NA78.CrossRoad hg19Virus_refGene_RIS.txt hg19 320
80 100 ../NA78.integration-sites.txt
```

1.69. WHAM

Run WHAM:

SVs were called with the whamg executable.

Convert output file:

RSSs were assigned with the values specified with the A tag in the output file. Sites with < 3 RSSs were filtered out. SVs with > 2 Mb or < 50 bp (< 30 bp for DELs) were filtered out. SVs were also filtered out when the value specified with the CW tag, which indicates the contribution of supporting evidence each to SV type, in the output file was < 0.2 for the corresponding SV type or when the CW value for BND was > 0.2.

Commands used:

```
whamg -f NA78.bam -a hs37d5.fa -x 4 -c
1,2,3,4,5,6,7,8,9,10,11,12,13,14,15,16,17,18,19,20,21,22,X,Y >
NA78.vcf
```

2. SV filtering

When the sizes of the called SVs were < 30 bp or > 2 Mb, they were filtered out. However, for

the SVs called using CNVnator, those < 15 Mb in length were allowed. Several SV detection algorithms, including CNVnator, often call SVs overlapping with gap regions of the reference. These SV sites were filtered when the length of the overlapped region between a CNV and a gap was larger than 30% of the CNV or the gap. A bed file of the reference gap regions was obtained from the UCSC Genome Browser site. When evaluating the performance of algorithms or merging call SV call sets, SVs with a smaller RSS value than a specified minimum RSS were filtered out.

3. Generation of simulated data with simulation tools

3.1 VarSim

The simulated diploid genomes for the Sim-A simulated data was generated with the hs37d5.fa reference genome using the VarSim simulator. VarSim introduced a total of 8,314 SVs, including 3,530 DELs, 1,656 DUPs, 2,819 INSs, and 309 INVs, with sizes ranged from 30 bp to 1 Mb, in addition to SNPs and short indels corresponding to 0.1% and 0.02% of the genome size, into simulated paternal and maternal haploid genomes, with approximately 3:2 of heterozygous and homozygous variants (Table S3 in Additional file 1). The accompanying ART simulator generated simulated reads consisted of 125 bp of paired-end reads with 30× coverage and with 500 bp insert size with 100 bp standard deviation with the paternal and maternal simulated genomes.

Commands used:

```
python varsim.py --vc_in_vcf $HOME/tool/VarSim/All.vcf --sv_insert_seq
$HOME/tool/VarSim/insert_seq.txt --sv_dgv
$HOME/tool/VarSim/GRCh37_hg19_supportingvariants_2013-07-23.txt
--reference hs37d5.fa --id Sim-A --read_length 125 --mean_fragment_size
500 --sd_fragment_size 100 --vc_num_snp 2700000 --vc_num_ins 270000
--vc_num_del 270000 --sv_num_ins 3000 --sv_num_del 4000 --sv_num_dup
2000 --sv_num_inv 500 --sv_percent_novel 0.2 --vc_percent_novel 0.01
--vc_min_length_lim 0 --vc_max_length_lim 29 --sv_min_length_lim 30
--sv_max_length_lim 1000000 --nlanes 1 --total_coverage 30
--simulator_executable
$HOME/tool/art_bin_ChocolateCherryCake/art_illumina --out_dir out
```

```
--log_dir log --simulator art --profile_1
$HOME/tool/art_bin_ChocolateCherryCake/Illumina_profiles/HiSeq2500L1
25R1.txt --profile_2
$HOME/tool/art_bin_ChocolateCherryCake/Illumina_profiles/HiSeq2500L1
25R2.txt
```

3.2 ART

Simulated paired-end reads for the Sim-MEI, Sim-VEI, Sim-NUMT, and Sim-A derivative data were generated with the simulated diploid genomes (`$simulated_genome_fasta`) using the ART simulator.

Commands used:

```
art_illumina -sam -i $simulated_genome_fasta -l 125 -f 15 -m 500 -s 100
-o $out_prefix -p -1
$HOME/tool/art_bin_ChocolateCherryCake/Illumina_profiles/HiSeq2500L1
25R1.txt -2
$HOME/tool/art_bin_ChocolateCherryCake/Illumina_profiles/HiSeq2500L1
25R2.txt
```

3.3 PBSIM

Simulated PacBio reads were generated with the Sim-A simulated diploid genomes and with the real PacBio reads (the PacBio-data1) as sample-fastq using the PBSIM simulator.

Commands used:

```
pbsim --data-type CLR --depth 5.0 --length-max 100000 --prefix
Sim-A-PacBio --sample-fastq $PacBio-data1-fastq
$Sim-A-diploid-genome-fasta
```
